# RV-Typer: A Web Server for Typing of *Rhinoviruses* Using Alignment-Free Approach

**DOI:** 10.1371/journal.pone.0149350

**Published:** 2016-02-12

**Authors:** Pandurang S. Kolekar, Vaishali P. Waman, Mohan M. Kale, Urmila Kulkarni-Kale

**Affiliations:** 1 Bioinformatics Centre, Savitribai Phule Pune University (formerly University of Pune), Pune, 411 007, India; 2 Department of Statistics, Savitribai Phule Pune University (formerly University of Pune), Pune, 411 007, India; University of Malaya, MALAYSIA

## Abstract

*Rhinoviruses* (RV) are increasingly being reported to cause mild to severe infections of respiratory tract in humans. RV are antigenically the most diverse species of the genus *Enterovirus* and family *Picornaviridae*. There are three species of RV (RV-A, -B and -C), with 80, 32 and 55 serotypes/types, respectively. Antigenic variation is the main limiting factor for development of a cross-protective vaccine against RV.Serotyping of *Rhinoviruses* is carried out using cross-neutralization assays in cell culture. However, these assays become laborious and time-consuming for the large number of strains. Alternatively, serotyping of RV is carried out by alignment-based phylogeny of both protein and nucleotide sequences of VP1. However, serotyping of RV based on alignment-based phylogeny is a multi-step process, which needs to be repeated every time a new isolate is sequenced. In view of the growing need for serotyping of RV, an alignment-free method based on “return time distribution” (RTD) of amino acid residues in VP1 protein has been developed and implemented in the form of a web server titled RV-Typer. RV-Typer accepts nucleotide or protein sequences as an input and computes return times of di-peptides (*k* = 2) to assign serotypes. The RV-Typer performs with 100% sensitivity and specificity. It is significantly faster than alignment-based methods. The web server is available at http://bioinfo.net.in/RV-Typer/home.html.

## Introduction

*Rhinoviruses* (RV) are the most frequently infecting human pathogens causing common cold infections. RV are the principle agents of acute respiratory tract illness and are increasingly being associated with more severe diseases such as acute otitis media, pneumonia, recurrent whizzing, asthma and bronchiolitis [1–3].

The RV belong to the genus *Enterovirus* of the family *Picornaviridae* and there are three species such as RV-A, -B and -C. They are small, non-enveloped, single-stranded RNA viruses containing a copy of positive sense genome (~7200 nt). The viral capsid comprises of 60 protomers, each of which contains four viral proteins (VPs), designated as VP1 to VP4. Within *Picornaviridae*, RV represents serologically the most diverse group. Currently, there are 80, 32 and 55 serotypes/types of RV-A, -B and -C respectively [4,5]. RV-C is known to cause majority of asthma attacks in children, as compared to RV-A and -B [3]. Moreover, RV-C has become a global public health concern due to its association with lower respiratory tract illnesses in children [2].

High antigenic diversity observed amongst RV is attributed to high mutation rate caused by the low fidelity of RNA-dependent RNA polymerase, which lacks proof-reading activity. The serotype diversity, especially in RV-A and RV-C, was also attributed to intra- or inter-species recombination events, mainly within 5’-UTR and non-structural genes [6]. New and emerging serotypes of RV have been reported and which need to be serotyped [7]. The serotypes of RV are determined using two approaches viz., cross neutralization assays [8,9] and phylogenetic analysis [10]. The experimental approach based on neutralization assays in cell culture is laborious and time-consuming and hence is impractical for serotyping of a large number of RV strains. The computational approach involves phylogenetic analysis of VP1. It is the largest of the four capsid proteins encoded by *Rhinoviruses* and is the most widely used phylogenetic marker for RV [10–14]. Use of both, protein as well as nucleotide sequences of VP1 for serotyping of RV has been well-documented [10,13,14]. Though the RV type assignments have also been carried out based on VP4/VP2 region, its shorter length and greater sequence conservation (as compared to VP1) limits its usage as a typing marker using both, alignment-based phylogeny [10] as well as alignment-free typing using RTD method.

Serotyping of viruses using the alignment-based phylogenetic analysis, however, is a multi-step process, which needs to be repeated every time a new isolate of RV is sequenced. Furthermore, the uncertainty and computational intensity associated with large-scale alignment-based phylogeny analyses are also known [15]. In view of this, alignment-free approaches provide a cost-effective and robust solution to sequence-based serotyping of RV and would be useful to understand their intriguing antigenic diversity. An alignment-free method based on return time distribution (RTD) has been developed in house and applied for serotyping of *Mumps*, *Dengue* and *West Nile viruses* using genomic sequences [16–20]. In this study RTD-based alignment-free approach has been extended for serotyping of RV using VP1 protein sequences and has been implemented in the form of web server for the serotyping of RV.

## Materials and Methods

### Data sets

The reference data set (S1 Table) consisting of a total of 432 sequences of VP1 protein of serotypes of RV-A (238), -B (83) and -C (111) was curated and compiled from the GenPept database at National Center for Biotechnology Information (NCBI) [21]. The information on RV serotypes available at the *Picornaviridae* study group of International Committee on Taxonomy of Viruses was used [4].

In order to assess the performance of web server, true positive (TP) and negative (TN) data sets were compiled such that there is no overlap with the reference data set. The TP data set (S2 Table) consists of 218 VP1 protein sequences of known serotypes/types of RV-A, -B and–C. The TN data set consists of 7101 protein sequences (S3 Table), which includes non-VP1 protein sequences of *Rhinoviruses*; and VP1 and non-VP1 protein sequences of other species of the family *Picornaviridae*. The TP and TN data sets were used to calculate sensitivity and specificity of RTD-based method proposed for the serotyping of RV. The data sets are also available from “Data sets” page of the web server.

### Methodology

The RTD-based alignment-free method was originally developed for molecular phylogeny and its applications for the genotyping of viruses using nucleotide sequences were demonstrated [16–20]. In this study RTD-based method was suitably modified for the use of protein sequences as an input for the first time and applied for the phylogenetic reconstruction as well as serotyping of RV using VP1 protein. The computations of return time are performed for a word or k-mer, the value of which may vary from 1 to n. For the chosen value of *k*, the RTDs for each of the k-mers are computed and summarized using statistical parameters viz., mean (*μ*) and standard deviation (*σ*) for each of the sequences. Since there are 20^*k*^ possible k-mers for chosen value of *k* and the RTD of each k-mer has two parameters (*μ* and *σ*), each protein sequence is represented as a numeric vector of size 2*20^*k*^. In case of absence of RTD of any k-mer in protein sequence, its *μ* and *σ* were assigned to zero. A sample computation for *μ* and *σ* for RTDs at *k* = 1 is given in S1 File.

The Euclidean distance measure reported earlier [19] was used to compute the pairwise distances between protein sequences using the respective numeric vectors of parameters of RTD. The distance matrix thus obtained was used as an input to Neighbor joining (NJ) method to derive distance-based phylogenetic tree using the Neighbor program in PHYLIP package [22].

Optimisation of size of k-mer is one of the most important aspects of this method. The optimum size of k-mer was determined based on the accuracy of the phylogenetic tree reconstructed for the reference data set by varying value of *k* from 1 to 3. The NJ tree generated using alignment-based molecular phylogeny analysis of the reference data set is used as the reference tree to assess the accurancy of the RTD-based tree and thereby to optimise value of *k*. The value of *k*, for which the resultant phylogenetic tree showed accurate classification of RV species and their respective serotypes, was selected to be optimum for RTD based serotyping of RV. The RTD of reference data set of RV at the optimum value of *k* is subsequently used as the knowledgebase at the backend of the server. The distance cut-offs for various serotypes of each of the three RV species are derived and used for subsequent typing of individual RV sequences, submitted as query.

The serotyping methodology of a query sequence of RV using RTD-based method involves following steps; (1) calculating the RTDs and parameters of k-mers for input query sequence(s) of VP1 protein at optimum value of *k*, (2) computing the Euclidean distance of RTD-based numeric vector(s) of query sequence(s) from pre-computed numeric vector(s) of RV serotypes in reference data set at optimum value of *k*, (3) assign the serotype(s) of the closest reference serotype(s) to query sequence(s) based on distance proximity and pre-computed distance cut-off values.

### Implementation and availability of web server

The RTD-based methodology for serotyping of RV described above is implemented in the form of a web server namely RV-Typer, using Apache, PHP, CGI architecture and is made available online at http://bioinfo.net.in/RV-Typer/home.html.

## Results and Discussion

The wide antigenic diversity among serotypes of *Rhinoviruses* has posed a challenge in development of cross-serotype *Rhinovirus* vaccine [23]. The emergence of new lineages among *Rhinoviruses* has been proposed [6,7], which continues to be a cause of concern in design and development of vaccines against RV. The epidemiological surveillance and monitoring of circulating serotypes of RV in a population would be essential to prioritize vaccine candidate(s). Thus, identification of serotypes of circulating strains is critical in management of RV.

The return-time distribution (RTD) based alignment-free method was originally developed for phylogenetic analysis using nucleotide sequence data and its applications for geno- and serotyping of viruses such as *Mumps*, *Dengue*, *West Nil*e *viruses* were demonstrated successfully [16–20]. The method has been successfully modified to provide protein sequence data as an input for VP1 protein based serotyping of RV. The optimization of k-mer size for RTD-based serotyping of RV, description of the RTD-based web server, RV-Typer and its validation using true positive and negative data sets, is described.

### Optimization of k-mer size for serotyping of *Rhinoviruses*

**Fig 1:**
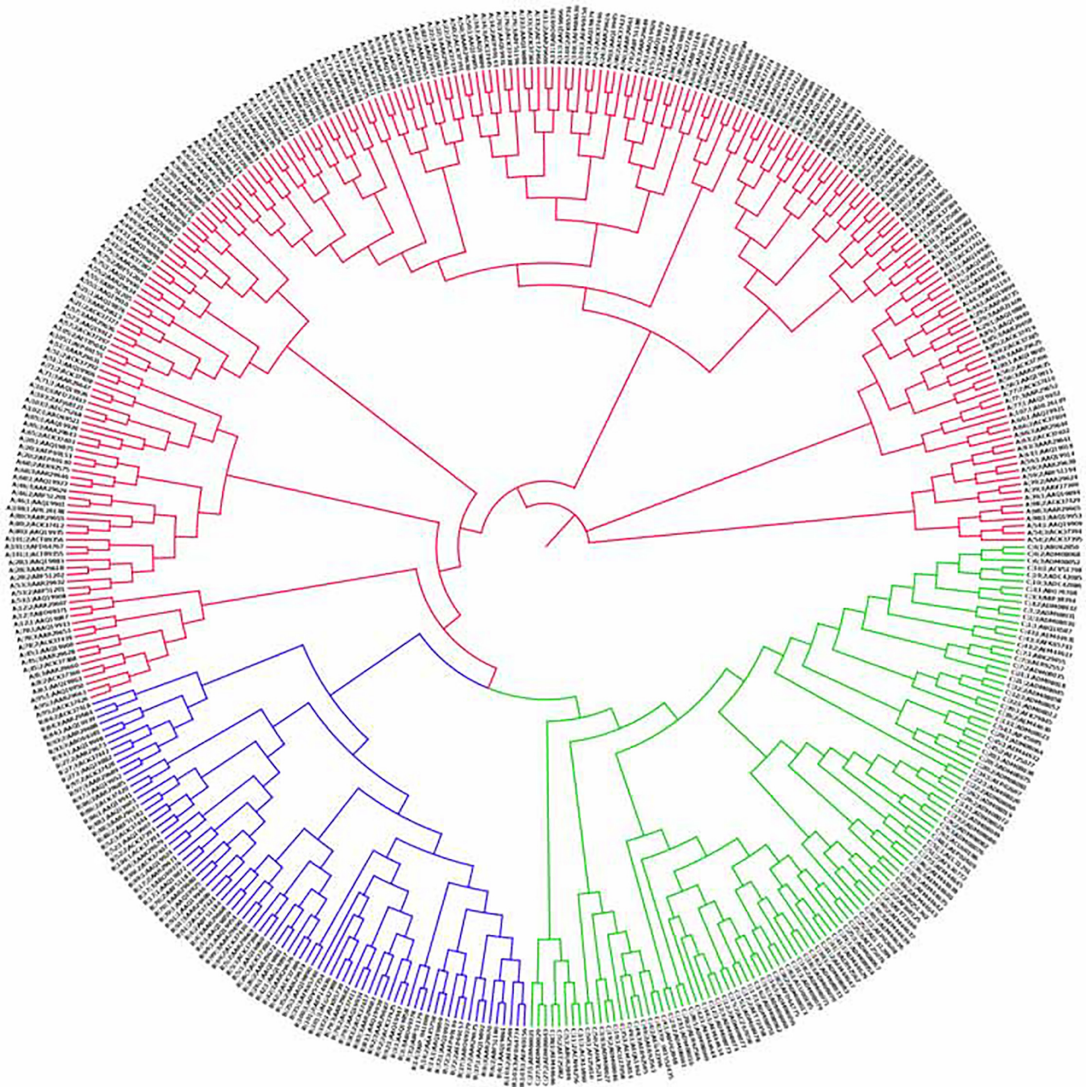
RTD-based phylogenetic tree of *Rhinoviruses* using VP1 reference data set at *k* = 2. The branches are color coded as following, *Rhinovirus A* (red), *Rhinovirus B* (blue) and *Rhinovirus C* (green). The tip labels are divided in 4 parts by ‘|’ characters indicating species, serotype, serial number of that serotype and GenPept accession number respectively. Note: The RTD-based phylogenetic tree for the reference and the true positive sequences of RV types is provided as S3 Fig.

The reference data set was subjected to RTD-based phylogenetic reconstruction at varying values of *k* as described in the method section. It was found that at *k* = 2 (i.e. RTDs of dipeptides) all the strains of RV in reference data set were accurately classified in clusters pertaining to all the three species and their respective serotypes. The phylogenetic tree obtained at *k* = 2 is shown in Fig 1. The RTDs of dipeptides have been shown to have information content for classification of protein sequences.

The phylogenetic grouping of all strains and serotypes obtained by the alignment-free RTD-based method (Fig 1) was found to be consistent with the alignment-based NJ tree (S1 Fig). All strains were correctly clustered into three distinct genetic groups corresponding to species RV-A, -B, and -C, at *k* = 2 (Fig 1). These results obtained using VP1 protein sequence are also in corroboration with the known taxonomic information of RV species and hence prove the applicability of RTD method for serotyping of RV species. Thus, RTD-based numeric vectors of reference serotypes obtained at *k* = 2 were used to calculate the species-specific distance cut-offs to predict the serotype(s) of query sequence(s).

### Description of the web server

The sequence submission form of RV-Typer accepts either genomic or protein sequence(s) consisting of VP1 region as an input. Users can submit sequences either by pasting them in text area or by uploading a text file. Upon submission of the sequence(s), a Perl script at the backend of server is executed, which extracts the VP1 protein sequence(s) by performing either BLASTP or BLASTX [24] search of input sequences (protein or nucleotide) against reference data set of VP1 protein. The BLAST search serves the purpose of extracting exact region(s) of VP1 protein sequences regardless of input data (genomic/proteomic). In the second phase, the Perl script proceeds with RTD-based serotyping of VP1 protein sequence(s) using the methodology described above. After successful completion of the job, the server provides an output consisting of the header of the input sequence(s) and predicted serotype using RTD-based method. The server also provides a summary of BLAST similarity search of query sequence(s) against the reference data set, which includes % pairwise identity between query and the closest serotype in reference data set, e-value, start and end position etc. This data will help users to compare and crossvalidate the type assignments provided by RTD-based method and BLAST. If the distance of input sequence is beyond the pre-computed species-specific distance cut-offs, the RTD-based method does not predict serotype and displays an error message. The distance cut-offs are derived based on extent of variation of distances observed between RV species and serotypes. The “Example” page made available on the server provides guidelines on how to use the RV-Typer.

It should also be noted that new serotypes of RV are continuously being reported by ICTV Picornaviridae study group. The reference data set of RV-Typer will be updated to include newly added RV types and corresponding distance cut-offs will be revised accordingly.

### Validation of the RV-Typer server

The performance of RV-Typer was validated using receiver operating characteristic (ROC) analysis. The sequences in TP and TN data sets were given as an input to RV Typer to calculate the sensitivity and specificity of the serotype prediction. It was found that RV Typer has 100% sensitivity and specificity for serotype prediction of RV. During validation of the RV-Typer server, one of the strains in true positive data set namely, HRV-B70_p1052_sR2777_2008 [GenPept: AFD64776.1], which was annotated as RV-B serotype 70 in its GenPept record, was serotyped as RV-B serotype 17 by the RTD-based method. This serotype prediction was further investigated by alignment-based molecular phylogeny analysis (MPA) of VP1 sequences in reference and true positive data sets. The multiple sequence alignment derived using MUSCLE algorithm [25] was given as an input to kimura-2-parameter model [26] with 1000 bootstrap replicates and phylogenetic tree was reconstructed using Neighbor-joining [22] method as implemented in MEGA 6 package [27]. The resultant phylogenetic tree (S2 Fig) re-confirmed that the strain HRV-B70_p1052_sR2777_2008 [GenPept: AFD64776.1] clusters with the members of RV-B serotype 17. Similarly, the RTD-based phylogenetic tree generated by combining reference and true positive data sets of VP1, also supported the same observation (S3 Fig). These results not only help us to validate performance of the server but also demonstrate its use for curation of serotype annotation errors, in GenPept (or any other database of RV). Thus, RV-Typer provides a tool for large-scale annotation of RV serotypes.

It was found that alignment-based phylogeny of VP1 sequences in reference and true positive data sets took around ~15 minutes. The users have to repeat all the steps of phylogeny, even for serotyping of a newly added single VP1 query sequence, such as compilation of reference and query sequence(s) followed by their multiple sequence alignment and then phylogenetic inference. Whereas serotyping of a VP1 sequence using RTD-based method implemented in the RV-Typer just took about ~2 seconds. Thus, RV-Typer provides a faster yet accurate alternative for the serotyping of RV.

### Effect of recombination on RTD-based typing of *Rhinoviruses*

Classification or typing of recombinant sequences using phylogenetic methods is a challenging problem. Though occurrence of recombination in VP1 gene is known to be less frequent [6,12], the gene sequences of VP1, compiled as the references data set were tested for recombination using various methods in RDP4 package [28–35]. It was observed that none of the sequences in the reference data set were identified as recombinants by at least three methods (with p-value < 0.00001), which was used as a criteria to detect recombination. Further, in order to assess the efficacy of RV-Typer for typing potential RV recombinants, simulated data was generated and tested. The intra- and inter-type recombinants of VP1 gene at varying levels of proportions of major and minor parents were constructed with % sequence proportions of 90–10, 80–20, 70–30, 60–40 and 50–50, respectively. While generating the simulated data sets of intra- and inter-typic recombinants (100 sequences in each), equal representation of sequences (~33%) from RV-A, B and C species were retained. The simulated data sets are given in S2 File. These simulated sequences were used to benchmark performance of the RV-Typer. The results of serotype prediction are provided in S3 File, which is linked on the “Validation” page of the RV-Typer. The simulated recombinant sequences can also be downloaded from this page. It was observed that the RV-Typer assigned the type of the major parent in case of both, intra- and inter-typic recombinants having sequence proportions of 90–10 and 80–20 from major and minor parents, respectively. Whereas in case of most of the recombinants with proportions of 70–30, 60–40 and 50–50 from respective parents, RV-Typer did not assign any serotype and in a few cases it assigned serotype of minor parent. Only ~6% of simulated recombinant sequences were assigned with the type other than the types of their parents. Thus, for most of the simulated data of recombinants, RV-Typer didn’t assign a type and displayed a note accordingly. Furthermore, in case of type assignment using BLAST, the type of the major parent gets assigned to the simulated data of inter- and intra-typic recombinant strains based on the longest region of similarity shown as the best hit. Therefore, if the results obtained by RV-Typer and BLAST do not match, users are suggested to carry out recombination detection analysis prior to typing.

## Conclusions

The RV-Typer server is RTD-based alignment-free robust tool for the serotyping of RV with highest levels of accuracy, sensitivity and specificity. RV-Typer is the first typing server that implements RTD method and uses protein sequences as an input. It is developed with an objective to speed up the species and serotype identification of new isolates of RV, especially in case of unculturable RV-C strains/isolates. RV-Typer is expected to be useful in epidemiological surveillance and serotyping of RV.

## Supporting Information

S1 Fig Alignment-based Neighbor-joining phylogenetic tree of *Rhinoviruses* using VP1 reference data set. The branches are color coded as following, *Rhinovirus A* (red), *Rhinovirus B* (blue) and *Rhinovirus C* (green). The tip labels are divided in 4 parts by ‘|’ characters indicating species, serotype, serial number of that serotype and GenPept accession number respectively.
(TIF)

S2 Fig Alignment-based Neighbor-joining phylogenetic tree of *Rhinoviruses* using VP1 sequences in reference and true-positive data sets. The branches are color coded as following, *Rhinovirus A* (red), *Rhinovirus B* (blue) and *Rhinovirus C* (green). The tip labels are divided in 4 parts by ‘|’ characters indicating species, serotype, serial number of that serotype and GenPept accession number respectively. The tip label of sequences from true positive data set begins with ‘TP’.
(TIF)

S3 Fig The RTD-based phylogenetic tree of *Rhinoviruses* using VP1 protein sequences in reference and true positive data sets. The branches are color coded as following, *Rhinovirus A* (red), *Rhinovirus B* (blue) and *Rhinovirus C* (green). The tip labels are divided in 4 parts by ‘|’ characters indicating species, serotype, serial number of that serotype and GenPept accession number respectively. The tip label of sequences from true positive data set begins with ‘TP’.
(TIF)

S1 File A sample computation for *μ* and *σ* of return time distribution at *k* = 1. (PDF)

S2 File The simulated data sets of intra- and inter-typic recombinant sequences generated in this study. The sequences were simulated at varying levels of proportions of major and minor parents with % sequence contributions of 90–10, 80–20, 70–30, 60–40 and 50–50% from respective parents. A readme file indicating the details of the data sets is also provided. The data sets (.txt format) can be extracted using WinRAR archiver.
(RAR)

S3 File Typing of simulated data of recombinants using RV-Typer. The file contains results of typing obtained using RV-Typer for simulated data sets of intra-typic (RV-A, -B and–C) as well as of inter-typic recombinant sequences.
(PDF)

S1 Table The reference data set of 432 VP1 protein sequences of serotypes of *Rhinoviruses* (RV) and their GenPept accession numbers used in this study. (PDF)

S2 Table The true positive data set of 218 VP1 protein sequences of serotypes of Rhinoviruses (RV) and their GenPept accession numbers used in this study. (PDF)

S3 Table The true negative data set of 7101 protein sequences and their GenPept accession numbers used in this study. (PDF)
