# Supplementary figures and images for: RV-Typer: A Web Server for Typing of Rhinoviruses Using Alignment-Free Approach
Source: PLoS One. 2016 Feb 12;11(2):e0149350. doi: 10.1371/journal.pone.0149350 (PMC4752186; doi:10.1371/journal.pone.0149350)

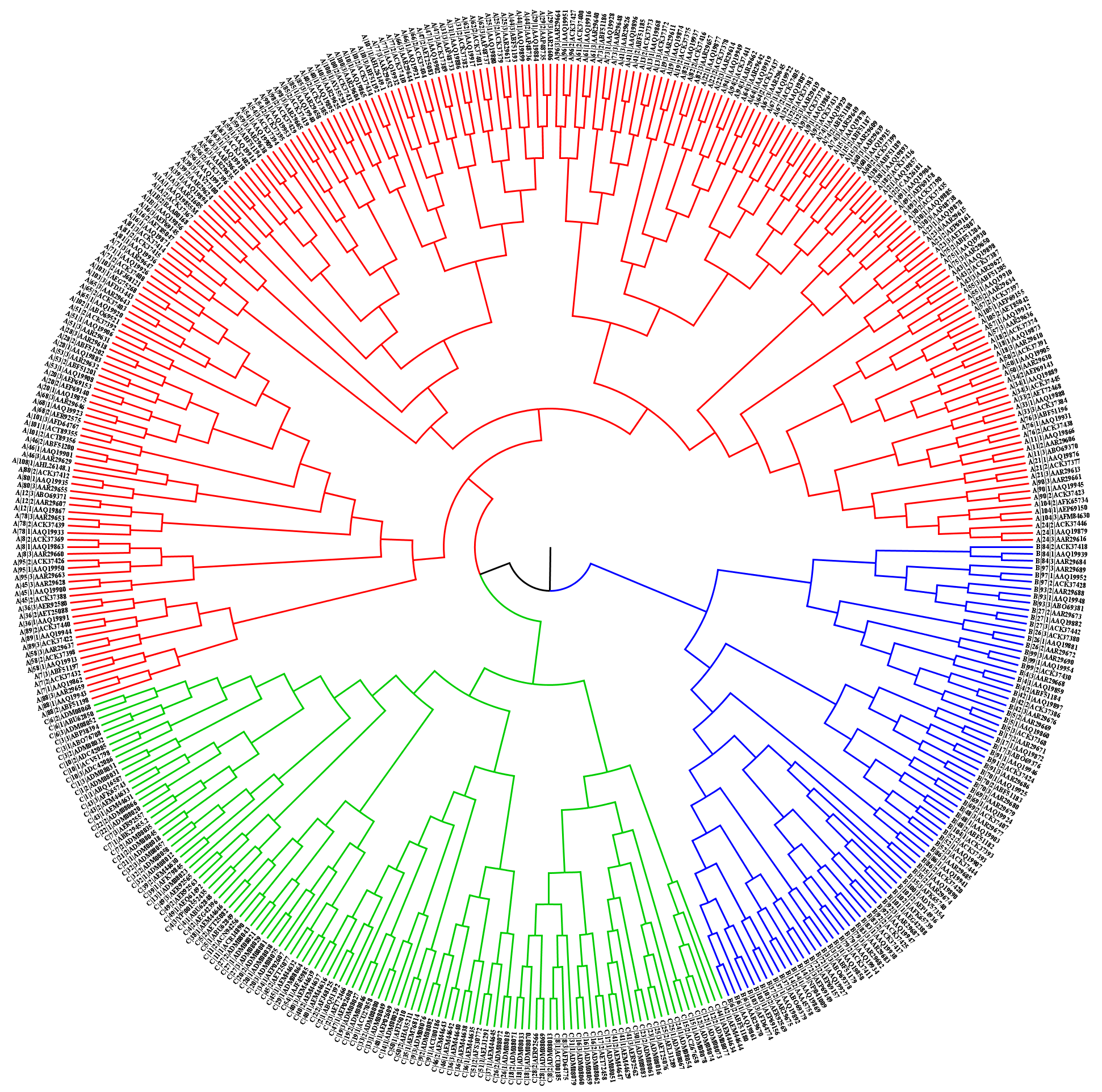

Supplement: S1 Fig — The branches are color coded as following, Rhinovirus A (red), Rhinovirus B (blue) and Rhinovirus C (green). The tip labels are divided in 4 parts by ‘|’ characters indicating species, serotype, serial number of that serotype and GenPept accession number respectively. (TIF) [file pone.0149350.s001.tif]

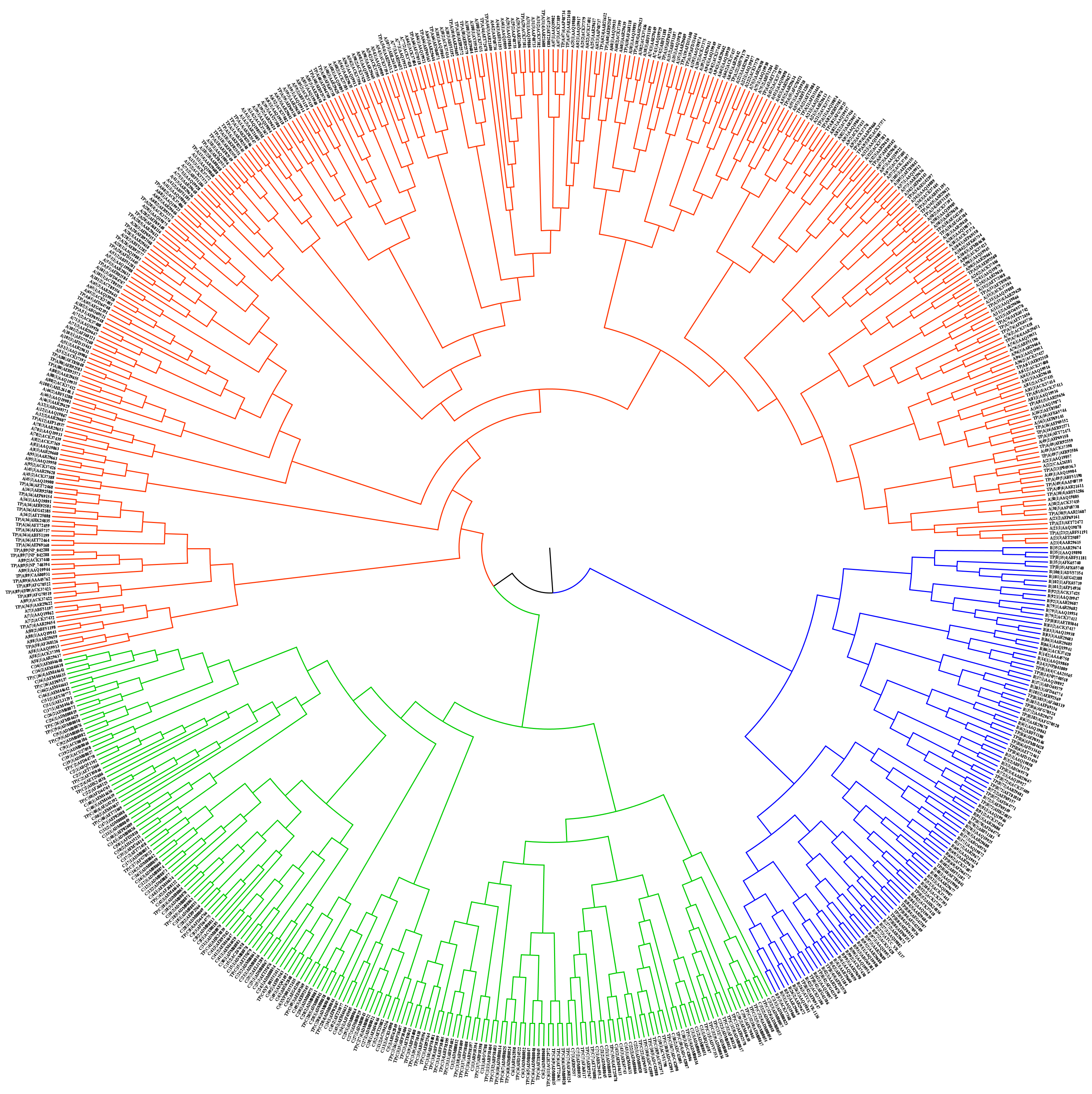

Supplement: S2 Fig — The branches are color coded as following, Rhinovirus A (red), Rhinovirus B (blue) and Rhinovirus C (green). The tip labels are divided in 4 parts by ‘|’ characters indicating species, serotype, serial number of that serotype and GenPept accession number respectively. The tip label of sequences from true positive data set begins with ‘TP’. (TIF) [file pone.0149350.s002.tif]

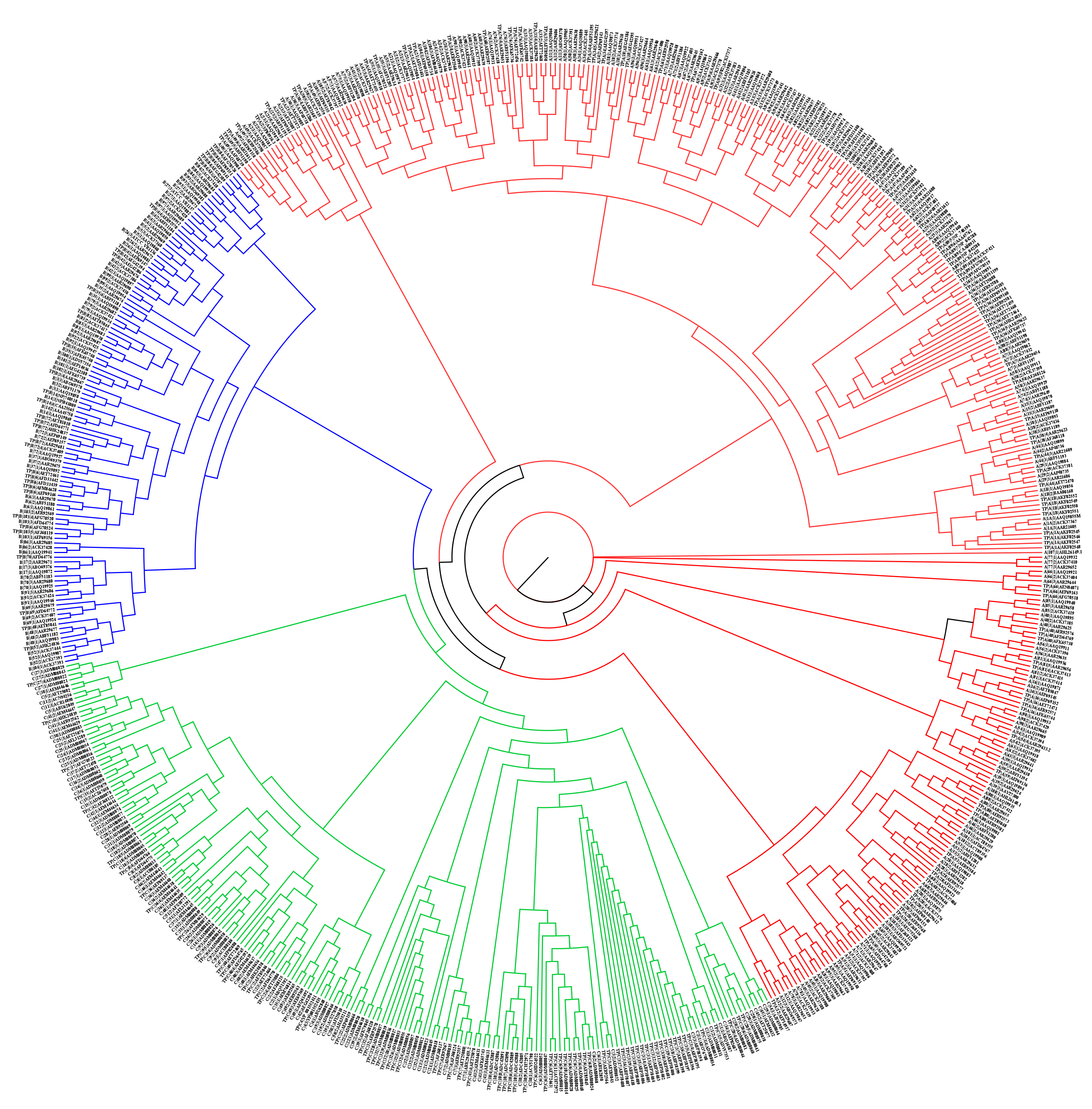

Supplement: S3 Fig — The branches are color coded as following, Rhinovirus A (red), Rhinovirus B (blue) and Rhinovirus C (green). The tip labels are divided in 4 parts by ‘|’ characters indicating species, serotype, serial number of that serotype and GenPept accession number respectively. The tip label of sequences from true positive data set begins with ‘TP’. (TIF) [file pone.0149350.s003.tif]
