# Supplementary material for: RV-Typer: A Web Server for Typing of Rhinoviruses Using Alignment-Free Approach
Source: PLoS One. 2016 Feb 12;11(2):e0149350. doi: 10.1371/journal.pone.0149350 (PMC4752186; doi:10.1371/journal.pone.0149350)
Supplement: S1 File — (PDF) [file pone.0149350.s004.pdf]

Supplementary file Text S1

RV-Typer: a web server for serotyping of *Rhinoviruses* using alignment-free approach

Pandurang Kolekar[1], Vaishali Waman[1], Mohan Kale[2] and Urmila Kulkarni-Kale[1§]

[1]Bioinformatics Centre, Savitribai Phule Pune University (formerly University of Pune), Pune 411 007, India.
[2]Department of Statistics, Savitribai Phule Pune University (formerly University of Pune), Pune 411 007, India.
[§]Corresponding author

Detailing of method to compute Return time distribution (RTD)

Sample protein sequence:

TLALAPAAPNPDKWNDFTAFFKVGAAACATTGTGAAYNCFYDGAACA

Computing RTD for ‘A’

TLALAPAAPNPDKWNDFTAFFKVGAAACATTGTGAAYNCFYDGAACA

1-1-0-10-5-0-0-1-5-0-7-0-1 **Return times for ‘A’**

RTD for ‘A’ in above sample sequence

Return time for A (X)	Frequency (F)
0	5
1	4
5	2
7	1
10	1

Parameters of RTD for ‘A’

μ(A) = 2.38 and σ(A) = 3.27

Similarly, we can compute μ and σ of RTDs of other amino acids for k = 1.

- At $k = 2$, there will be 400 k-mers (di-mers) of 20 amino acids, hence 400 RTDs can be computed using the same approach.

- So in general for other integer values/size of k, 20^k RTDs will be computed.
