# Supplementary material for: RV-Typer: A Web Server for Typing of Rhinoviruses Using Alignment-Free Approach
Source: PLoS One. 2016 Feb 12;11(2):e0149350. doi: 10.1371/journal.pone.0149350 (PMC4752186; doi:10.1371/journal.pone.0149350)
Supplement: S3 File — The file contains results of typing obtained using RV-Typer for simulated data sets of intra-typic (RV-A, -B and–C) as well as of inter-typic recombinant sequences. (PDF) [file pone.0149350.s006.pdf]

# RV-Typer: a web server for typing of *Rhinoviruses* using alignment-free approach

Pandurang S Kolekar[1], Vaishali P Waman[1], Mohan Kale[2] and Urmila Kulkarni-Kale [1*].

[1]Bioinformatics Centre, Savitribai Phule Pune University (formerly University of Pune), Pune 411 007, India.

[2]Department of Statistics, Savitribai Phule Pune University (formerly University of Pune), Pune 411 007, India.

[*]Corresponding author

## Supporting S3 File

**S4 Table:** RV-Typer based typing of simulated intra-typic (RV-A, B and C) recombinant sequences

| Proportion of major-minor parent types | Predicted type by RV-Typer | | | | Total |
|---|---|---|---|---|---|
| | Major type | Minor type | No type predicted | Other type | |
| 90-10 | 84 | 0 | 11 | 5 | 100 |
| 80-20 | 55 | 5 | 31 | 9 | 100 |
| 70-30 | 31 | 20 | 43 | 6 | 100 |
| 60-40 | 18 | 34 | 42 | 6 | 100 |
| 50-50 | 9 | 43 | 43 | 5 | 100 |

**S5 Table:** RV-Typer based typing of simulated inter-typic (RV-A & B, RV-A & C, RV-B & C) recombinant sequences

| Proportion of major-minor parent types | Predicted type by RV-Typer | | | | Total |
|---|---|---|---|---|---|
| | Major type | Minor type | No type predicted | Other type | |
| 90-10 | 81 | 0 | 10 | 9 | 100 |
| 80-20 | 41 | 0 | 51 | 8 | 100 |
| 70-30 | 13 | 0 | 76 | 11 | 100 |
| 60-40 | 12 | 0 | 86 | 2 | 100 |
| 50-50 | 3 | 2 | 95 | 0 | 100 |
