# Supplementary material for: RV-Typer: A Web Server for Typing of Rhinoviruses Using Alignment-Free Approach
Source: PLoS One. 2016 Feb 12;11(2):e0149350. doi: 10.1371/journal.pone.0149350 (PMC4752186; doi:10.1371/journal.pone.0149350)
Supplement: S1 Table — (PDF) [file pone.0149350.s007.pdf]

RV-Typer: a web server for serotyping of *Rhinoviruses* using alignment-free approach

Pandurang Kolekar[1], Vaishali Waman[1], Mohan Kale[2] and Urmila Kulkarni-Kale [1§].

[1]Bioinformatics Centre, Savitribai Phule Pune University (formerly University of Pune), Pune 411 007, India.
[2]Department of Statistics, Savitribai Phule Pune University (formerly University of Pune), Pune 411 007, India.
[§]Corresponding author

Table S1: The reference data set of 432 VP1 protein sequences of serotypes of *Rhinoviruses* (RV) and their GenPept accession numbers used in this study.

Sr. no.	RV-species	RV serotype	GenPept Accession Number	Reference
1.	RV-A	108	AHL26148.1	http://www.picornastudygroup.com/types/enterovirus/hrv-a.htm
2.	RV-A	107	AHL26149.1	http://www.picornastudygroup.com/types/enterovirus/hrv-a.htm
3.	RV-A	105	AEP69155.1	http://www.picornastudygroup.com/types/enterovirus/hrv-a.htm
4.	RV-A	105	AET85042.1	http://www.picornastudygroup.com/types/enterovirus/hrv-a.htm
5.	RV-A	104	AEP69150.1	http://www.picornastudygroup.com/types/enterovirus/hrv-a.htm
6.	RV-A	104	AFK65734.1	http://www.picornastudygroup.com/types/enterovirus/hrv-a.htm
7.	RV-A	104	AFM84630.1	http://www.picornastudygroup.com/types/enterovirus/hrv-a.htm
8.	RV-A	103	AEG75268.1	http://www.picornastudygroup.com/types/enterovirus/hrv-a.htm
9.	RV-A	103	AFJ68121.1	Benson et al., 2013
10.	RV-A	103	AFD33443.1	Benson et al., 2013
11.	RV-A	102	ABO69521.1	http://www.picornastudygroup.com/types/enterovirus/hrv-a.htm; de Vries et al., 2008
12.	RV-A	101	ACT89355.1	Rathe et al., 2010
13.	RV-A	101	ACT89356.1	Rathe et al., 2010
14.	RV-A	101	AFD64767.1	http://www.picornastudygroup.com/types/enterovirus/hrv-a.htm
15.	RV-A	100	AAQ19955.1	Ledford et al., 2004
16.	RV-A	100	ACK37431.1	Palmenberg et al., 2009
17.	RV-A	100	AAR29604.1	Laine et al., 2005
18.	RV-A	98	AAQ19953.1	Ledford et al., 2004
19.	RV-A	98	ACK37429.1	Palmenberg et al., 2009
20.	RV-A	98	AAR29665.1	Laine et al., 2005
21.	RV-A	96	AAQ19951.1	Ledford et al., 2004
22.	RV-A	96	ACK37427.1	Palmenberg et al., 2009
23.	RV-A	96	AAR29664.1	Laine et al., 2005
24.	RV-A	95	AAQ19950.1	Ledford et al., 2004
25.	RV-A	95	ACK37426.1	Palmenberg et al., 2009
26.	RV-A	95	AAR29663.1	Laine et al., 2005
27.	RV-A	94	AAQ19949.1	Ledford et al., 2004
28.	RV-A	94	ACK37441.1	Palmenberg et al., 2009
29.	RV-A	94	AAR29662.1	Laine et al., 2005
30.	RV-A	90	AAQ19945.1	Ledford et al., 2004
31.	RV-A	90	ACK37423.1	Palmenberg et al., 2009
32.	RV-A	90	AAR29661.1	Laine et al., 2005
33.	RV-A	89	AAQ19944.1	Ledford et al., 2004
34.	RV-A	89	ACK37440.1	Palmenberg et al., 2009
35.	RV-A	89	ACK37422.1	Palmenberg et al., 2009
36.	RV-A	88	AAQ19943.1	Ledford et al., 2004
37.	RV-A	88	ABF51198.1	Kistler et al., 2007

38.	RV-A	88	AAR29659.1	Laine et al., 2005
39.	RV-A	85	AAQ19940.1	Ledford et al., 2004
40.	RV-A	85	ACK37419.1	Palmenberg et al., 2009
41.	RV-A	85	AAR29658.1	Laine et al., 2005
42.	RV-A	82	AAQ19937.1	Ledford et al., 2004
43.	RV-A	82	ACK37416.1	Palmenberg et al., 2009
44.	RV-A	82	AAR29657.1	Laine et al., 2005
45.	RV-A	81	AAQ19936.1	Ledford et al., 2004
46.	RV-A	81	ACK37415.1	Palmenberg et al., 2009
47.	RV-A	81	ACK37414.1	Palmenberg et al., 2009
48.	RV-A	80	AAQ19935.1	Ledford et al., 2004
49.	RV-A	80	ACK37412.1	Palmenberg et al., 2009
50.	RV-A	80	AAR29655.1	Laine et al., 2005
51.	RV-A	78	AAQ19933.1	Ledford et al., 2004
52.	RV-A	78	ACK37439.1	Palmenberg et al., 2009
53.	RV-A	78	AAR29653.1	Laine et al., 2005
54.	RV-A	77	AAQ19932.1	Ledford et al., 2004
55.	RV-A	77	ACK37410.1	Palmenberg et al., 2009
56.	RV-A	77	AAR29652.1	Laine et al., 2005
57.	RV-A	76	AAQ19931.1	Ledford et al., 2004
58.	RV-A	76	ACK37438.1	Palmenberg et al., 2009
59.	RV-A	76	ABF51196.1	Kistler et al., 2007
60.	RV-A	75	AAQ19930.1	Ledford et al., 2004
61.	RV-A	75	ABF51204.1	Kistler et al., 2007
62.	RV-A	75	AAR29650.1	Laine et al., 2005
63.	RV-A	74	AAQ19929.1	Ledford et al., 2004
64.	RV-A	74	ABF51188.1	Kistler et al., 2007
65.	RV-A	74	AAR29649.1	Laine et al., 2005
66.	RV-A	73	AAQ19928.1	Ledford et al., 2004
67.	RV-A	73	ABF51186.1	Kistler et al., 2007
68.	RV-A	73	AAR29648.1	Laine et al., 2005
69.	RV-A	71	AAQ19926.1	Ledford et al., 2004
70.	RV-A	71	ACK37408.1	Palmenberg et al., 2009
71.	RV-A	71	AAR29647.1	Laine et al., 2005
72.	RV-A	68	AAQ19923.1	Ledford et al., 2004
73.	RV-A	68	AER92575	Benson et al., 2013
74.	RV-A	68	AAR29646.1	Laine et al., 2005
75.	RV-A	67	AAQ19922.1	Ledford et al., 2004
76.	RV-A	67	ACK37405.1	Palmenberg et al., 2009
77.	RV-A	67	AAR29645.1	Laine et al., 2005
78.	RV-A	66	AAQ19921.1	Ledford et al., 2004
79.	RV-A	66	ACK37404.1	Palmenberg et al., 2009
80.	RV-A	66	AAR29644.1	Laine et al., 2005
81.	RV-A	65	AAQ19920.1	Ledford et al., 2004
82.	RV-A	65	ACK37403.1	Palmenberg et al., 2009
83.	RV-A	65	AAR29643.1	Laine et al., 2005
84.	RV-A	64	AAQ19919.1	Ledford et al., 2004
85.	RV-A	64	ACK37437.1	Palmenberg et al., 2009
86.	RV-A	64	AAR29642.1	Laine et al., 2005
87.	RV-A	63	AAQ19918.1	Ledford et al., 2004
88.	RV-A	63	ACK37402	Palmenberg et al., 2009
89.	RV-A	63	AAR29641.1	Laine et al., 2005
90.	RV-A	62	AAQ19917.1	Ledford et al., 2004

91.	RV-A	62	ACK37401	Palmenberg et al., 2009
92.	RV-A	62	AAP48737.1	Vlasak et al.; 2003
93.	RV-A	61	AAQ19916.1	Ledford et al., 2004
94.	RV-A	61	ACK37400.1	Palmenberg et al., 2009
95.	RV-A	61	AAR29640.1	Laine et al., 2005
96.	RV-A	60	AAQ19915.1	Ledford et al., 2004
97.	RV-A	60	ACK37399.1	Palmenberg et al., 2009
98.	RV-A	60	AAR29639.1	Laine et al., 2005
99.	RV-A	59	AAQ19914.1	Ledford et al., 2004
100.	RV-A	59	ABF51194.1	Kistler et al 2003
101.	RV-A	59	AAR29638.1	Laine et al., 2005
102.	RV-A	58	AAQ19913.1	Ledford et al., 2004
103.	RV-A	58	ACK37398.1	Palmenberg et al., 2009
104.	RV-A	58	AAR29637.1	Laine et al., 2005
105.	RV-A	57	AAQ19912.1	Ledford et al., 2004
106.	RV-A	57	ACK37397.1	Palmenberg et al., 2009
107.	RV-A	57	AAR29636.1	Laine et al., 2005
108.	RV-A	56	AAQ19911.1	Ledford et al., 2004
109.	RV-A	56	ACK37396.1	Palmenberg et al., 2009
110.	RV-A	56	AAR29635.1	Laine et al., 2005
111.	RV-A	55	AAQ19910.1	Ledford et al., 2004
112.	RV-A	55	AAR29634.1	Laine et al., 2005
113.	RV-A	55	ABF51205.1	Kistler et al 2003
114.	RV-A	54	AAQ19909.1	Ledford et al., 2004
115.	RV-A	54	ACK37395.1	Palmenberg et al., 2009
116.	RV-A	54	ACK37394.1	Palmenberg et al., 2009
117.	RV-A	53	AAQ19908.1	Ledford et al., 2004
118.	RV-A	53	ABF51201.1	Kistler et al 2003
119.	RV-A	53	AAR29632.1	Laine et al., 2005
120.	RV-A	51	AAQ19906.1	Ledford et al., 2004
121.	RV-A	51	ACK37392.1	Palmenberg et al., 2009
122.	RV-A	51	AAR29631.1	Laine et al., 2005
123.	RV-A	50	AAQ19905.1	Ledford et al., 2004
124.	RV-A	50	ACK37391.1	Palmenberg et al., 2009
125.	RV-A	50	AAR29630.1	Laine et al., 2005
126.	RV-A	49	AAQ19904.1	Ledford et al., 2004
127.	RV-A	49	AEP69158.1	Benson et al., 2013
128.	RV-A	49	ACK37390.1	Palmenberg et al., 2009
129.	RV-A	47	AAQ19902.1	Ledford et al., 2004
130.	RV-A	47	AET25083.1	Benson et al., 2013
131.	RV-A	47	ACK37389.1	Palmenberg et al., 2009
132.	RV-A	46	AAQ19901.1	Ledford et al., 2004
133.	RV-A	46	ABF51200.1	Kistler et al 2003
134.	RV-A	46	AAR29629.1	Laine et al., 2005
135.	RV-A	45	AAQ19900.1	Ledford et al., 2004
136.	RV-A	45	ACK37388.1	Palmenberg et al., 2009
137.	RV-A	45	AAR29628.1	Laine et al., 2005
138.	RV-A	44	AAQ19899.1	Ledford et al., 2004
139.	RV-A	44	AAP48736.1	Benson et al., 2013
140.	RV-A	44	ABF51193.1	Kistler et al 2003
141.	RV-A	43	AAQ19898.1	Ledford et al., 2004
142.	RV-A	43	ACK37387.1	Palmenberg et al., 2009
143.	RV-A	43	AAR29627.1	Laine et al., 2005

144.	RV-A	41	AAQ19896.1	Ledford et al., 2004
145.	RV-A	41	ABF51185.1	Kistler et al 2003
146.	RV-A	41	AAR29626.1	Laine et al., 2005
147.	RV-A	40	AAQ19895.1	Ledford et al., 2004
148.	RV-A	40	ACK37385.1	Palmenberg et al., 2009
149.	RV-A	40	AAR29625.1	Laine et al., 2005
150.	RV-A	39	AAQ19894.1	Ledford et al., 2004
151.	RV-A	39	AAR29624.1	Laine et al., 2005
152.	RV-A	39	AAV27300.1	Harris and Racaniello, 2005
153.	RV-A	38	AAQ19893.1	Ledford et al., 2004
154.	RV-A	38	ACK37436.1	Palmenberg et al., 2009
155.	RV-A	38	ABF51189.1	Kistler et al 2003
156.	RV-A	36	AAQ19891.1	Ledford et al., 2004
157.	RV-A	36	AET25088.1	Benson et al., 2013
158.	RV-A	36	AER92580.1	Benson et al., 2013
159.	RV-A	34	AAQ19889.1	Ledford et al., 2004
160.	RV-A	34	AEP69143.1	Benson et al., 2013
161.	RV-A	34	ACK37445.1	Palmenberg et al., 2009
162.	RV-A	33	AAQ19888.1	Ledford et al., 2004
163.	RV-A	33	AET72468.1	Benson et al., 2013
164.	RV-A	33	ACK37384.1	Palmenberg et al., 2009
165.	RV-A	32	AAQ19887.1	Ledford et al., 2004
166.	RV-A	32	ACK37383.1	Palmenberg et al., 2009
167.	RV-A	32	AAR29619.1	Laine et al., 2005
168.	RV-A	31	AAQ19886.1	Ledford et al., 2004
169.	RV-A	31	ACK37382.1	Palmenberg et al., 2009
170.	RV-A	31	AAP48733.1	Benson et al., 2013
171.	RV-A	30	AAQ19885.1	Ledford et al., 2004
172.	RV-A	30	ACK37435.1	Palmenberg et al., 2009
173.	RV-A	30	AAP48738.1	Benson et al., 2013
174.	RV-A	29	AAQ19884.1	Ledford et al., 2004
175.	RV-A	29	AAP48735.1	Benson et al., 2013
176.	RV-A	29	AAR21606.1	Laine et al., 2005
177.	RV-A	28	AAQ19883.1	Ledford et al., 2004
178.	RV-A	28	ABF51202.1	Kistler et al 2003
179.	RV-A	28	AAR29618.1	Laine et al., 2005
180.	RV-A	25	AAQ19880.1	Ledford et al., 2004
181.	RV-A	25	ACK37379.1	Palmenberg et al., 2009
182.	RV-A	25	AAR29617.1	Laine et al., 2005
183.	RV-A	24	AAQ19879.1	Ledford et al., 2004
184.	RV-A	24	ACK37446.1	Palmenberg et al., 2009
185.	RV-A	24	AAR29616.1	Laine et al., 2005
186.	RV-A	23	AAQ19878.1	Ledford et al., 2004
187.	RV-A	23	AEP69161	Benson et al., 2013
188.	RV-A	23	AET25087	Benson et al., 2013
189.	RV-A	23	AAR29615.1	Laine et al., 2005
190.	RV-A	22	AAQ19877.1	Ledford et al., 2004
191.	RV-A	22	ACK37378.1	Palmenberg et al., 2009
192.	RV-A	22	AAR29614.1	Laine et al., 2005
193.	RV-A	21	AAQ19876.1	Ledford et al., 2004
194.	RV-A	21	ACK37377.1	Palmenberg et al., 2009
195.	RV-A	21	AAR29613.1	Laine et al., 2005
196.	RV-A	20	AAQ19875.1	Ledford et al., 2004

197.	RV-A	20	AEP69140.1	Benson et al., 2013
198.	RV-A	20	AEP69153.1	Benson et al., 2013
199.	RV-A	19	AAQ19874.1	Ledford et al., 2004
200.	RV-A	19	ACK37375.1	Palmenberg et al., 2009
201.	RV-A	19	AAR29611.1	Laine et al., 2005
202.	RV-A	18	AAQ19873.1	Ledford et al., 2004
203.	RV-A	18	ACK37374.1	Palmenberg et al., 2009
204.	RV-A	18	AAR29610.1	Laine et al., 2005
205.	RV-A	16	AAQ19871.1	Ledford et al., 2004
206.	RV-A	16	AET85047.1	Benson et al., 2013
207.	RV-A	16	AEP69145.1	Benson et al., 2013
208.	RV-A	15	AAQ19870.1	Ledford et al., 2004
209.	RV-A	15	ABF51187.1	Kistler et al 2003
210.	RV-A	15	AAR29609.1	Laine et al., 2005
211.	RV-A	13	AAQ19868.1	Ledford et al., 2004
212.	RV-A	13	ACK37373.1	Palmenberg et al., 2009
213.	RV-A	13	ACK37372.1	Palmenberg et al., 2009
214.	RV-A	12	AAQ19867.1	Ledford et al., 2004
215.	RV-A	12	AAR29607.1	Laine et al., 2005
216.	RV-A	12	ABO69371.1	Tapparel et al., 2007
217.	RV-A	11	AAQ19866.1	Ledford et al., 2004
218.	RV-A	11	AAR29606.1	Laine et al., 2005
219.	RV-A	11	ABO69370.1	Tapparel et al., 2007
220.	RV-A	10	AAQ19865.1	Ledford et al., 2004
221.	RV-A	10	ACK37434.1	Palmenberg et al., 2009
222.	RV-A	10	ABF51192.1	Kistler et al., 2003
223.	RV-A	9	AAQ19864.1	Ledford et al., 2004
224.	RV-A	9	ACK37433.1	Palmenberg et al., 2009
225.	RV-A	9	ACK37370.1	Palmenberg et al., 2009
226.	RV-A	8	AAQ19863.1	Ledford et al., 2004
227.	RV-A	8	ACK37369.1	Palmenberg et al., 2009
228.	RV-A	8	AAR29660.1	Laine 2005
229.	RV-A	7	AAQ19862.1	Ledford et al., 2004
230.	RV-A	7	ACK37432.1	Palmenberg et al., 2009
231.	RV-A	7	ABF51197.1	Kistler et al., 2003
232.	RV-A	2	AAQ19857.1	Ledford et al., 2004
233.	RV-A	2	CAA26181	Skern et al., 2005
234.	RV-A	1B	AAQ19856.1	Ledford et al., 2004
235.	RV-A	1B	BAA00168	Hughes et al., 1988
236.	RV-A	1A	AAQ19855.1	Ledford et al., 2004
237.	RV-A	1A	ACK37367.1	Palmenberg et al., 2009
238.	RV-A	1A	AAR21605.1	Laine et al., 2005
239.	RV-B	104	ACK37393.1	http://www.picornastudygroup.com/types/enterovirus/hrv-b.htm, Palmenberg et al., 2009
240.	RV-B	103	AEP69156.1	http://www.picornastudygroup.com/types/enterovirus/hrv-b.htm
241.	RV-B	103	AER92569	http://www.picornastudygroup.com/types/enterovirus/hrv-b.htm
242.	RV-B	103	AFD64774.1	http://www.picornastudygroup.com/types/enterovirus/hrv-b.htm
243.	RV-B	102	AFK65739.1	http://www.picornastudygroup.com/types/enterovirus/hrv-b.htm
244.	RV-B	101	AEG42388	http://www.picornastudygroup.com/types/enterovirus/hrv-b.htm
245.	RV-B	101	AEP14936.1	http://www.picornastudygroup.com/types/enterovirus/hrv-b.htm
246.	RV-B	100	ADV57354.1	http://www.picornastudygroup.com/types/enterovirus/hrv-b.htm; Linsuwanon et al., 2011
247.	RV-B	99	AAQ19954.1	Ledford et al., 2004
248.	RV-B	99	ACK37430.1	Palmenberg et al., 2009

249.	RV-B	99	AAR29690.1	Laine et al., 2005
250.	RV-B	97	AAQ19952.1	Ledford et al., 2004
251.	RV-B	97	ACK37428.1	Palmenberg et al., 2009
252.	RV-B	97	AAR29689.1	Laine et al., 2005
253.	RV-B	93	AAQ19948.1	Ledford et al., 2004
254.	RV-B	93	AAR29688.1	Laine et al., 2005
255.	RV-B	93	ABO69381.1	Tapparel et al., 2007
256.	RV-B	92	AAQ19947.1	Ledford et al., 2004
257.	RV-B	92	ACK37425.1	Palmenberg et al., 2009
258.	RV-B	92	AAR29687.1	Laine et al., 2005
259.	RV-B	91	AAQ19946.1	Ledford et al., 2004
260.	RV-B	91	ACK37424.1	Palmenberg et al., 2009
261.	RV-B	91	AAR29686.1	Laine et al., 2005
262.	RV-B	86	AAQ19941.1	Ledford et al., 2004
263.	RV-B	86	ACK37420.1	Palmenberg et al., 2009
264.	RV-B	86	AAR29685.1	Laine et al., 2005
265.	RV-B	84	AAQ19939.1	Ledford et al., 2004
266.	RV-B	84	ACK37418.1	Palmenberg et al., 2009
267.	RV-B	84	AAR29684.1	Laine et al., 2005
268.	RV-B	83	AAQ19938.1	Ledford et al., 2004
269.	RV-B	83	ACK37417.1	Palmenberg et al., 2009
270.	RV-B	83	AAR29683.1	Laine et al., 2005
271.	RV-B	79	AAQ19934.1	Ledford et al., 2004
272.	RV-B	79	ACK37411.1	Palmenberg et al., 2009
273.	RV-B	79	AAR29682.1	Laine et al., 2005
274.	RV-B	72	AAQ19927.1	Laine et al., 2005
275.	RV-B	72	AEP69157.1	Benson et al., 2013
276.	RV-B	72	AEP69149.1	Benson et al., 2013
277.	RV-B	70	AAQ19925.1	Laine et al., 2005
278.	RV-B	70	ABF51183.1	Kistler et al., 2003
279.	RV-B	70	AAR29680.1	Laine et al., 2005
280.	RV-B	69	AAQ19924.1	Ledford et al., 2004
281.	RV-B	69	ACK37407.1	Palmenberg et al., 2009
282.	RV-B	69	AAR29679.1	Laine et al., 2005
283.	RV-B	52	AAQ19907.1	Ledford et al., 2004
284.	RV-B	52	ACK37444.1	Palmenberg et al., 2009
285.	RV-B	52	AAR29678.1	Laine et al., 2005
286.	RV-B	48	AAQ19903.1	Ledford et al., 2004
287.	RV-B	48	ABF51182.1	Kistler et al ., 2003
288.	RV-B	48	AAR29677.1	Laine et al., 2005
289.	RV-B	42	AAQ19897.1	Ledford et al., 2004
290.	RV-B	42	ACK37386.1	Palmenberg et al., 2009
291.	RV-B	42	AAR29676.1	Laine et al., 2005
292.	RV-B	37	AAQ19892.1	Ledford et al., 2004
293.	RV-B	37	AAR29675.1	Laine et al., 2005
294.	RV-B	37	ABO69379.1	Ledford et al., 2004
295.	RV-B	35	AAQ19890.1	Ledford et al., 2004
296.	RV-B	35	AAR29674.1	Laine et al., 2005
297.	RV-B	35	AFK65740	Benson et al., 2013
298.	RV-B	27	AAQ19882.1	Ledford et al., 2004
299.	RV-B	27	AAR29673.1	Laine et al., 2005
300.	RV-B	27	ACK37442.1	Palmenberg et al., 2009
301.	RV-B	26	AAQ19881.1	Ledford et al., 2004

302.	RV-B	26	AAR29672.1	Laine et al., 2005
303.	RV-B	26	ACK37380.1	Palmenberg et al., 2009
304.	RV-B	17	AAQ19872.1	Ledford et al., 2004
305.	RV-B	17	AAR29671.1	Laine et al., 2005
306.	RV-B	17	ABO69376.1	Tapparel et al., 2007
307.	RV-B	14	AAQ19869.1	Ledford et al., 2004
308.	RV-B	14	AAA45758.1	Lee et al., 1993
309.	RV-B	14	NP_041009.1	Callahan et al., 1993
310.	RV-B	6	AAQ19861.1	Ledford et al., 2004
311.	RV-B	6	ABF51180.1	Kistler et al., 2003
312.	RV-B	6	AAR29670.1	Laine et al., 2005
313.	RV-B	5	AAQ19860.1	Ledford et al., 2004
314.	RV-B	5	AAR29669.1	Laine et al., 2005
315.	RV-B	5	ACK37368.1	Palmenberg et al., 2009
316.	RV-B	4	AAQ19859.1	Ledford et al., 2004
317.	RV-B	4	ABF51184.1	Kistler et al., 2003
318.	RV-B	4	AAR29668.1	Laine et al., 2005
319.	RV-B	3	AAQ19858.1	Ledford et al., 2004
320.	RV-B	3	ABF51179.1	Kistler et al., 2003
321.	RV-B	3	ABO69378.1	Tapparel et al., 2007
322.	RV-C	1	ABQ16587.1	http://www.picornastudygroup.com/types/enterovirus/hrv-c.htm; Kistler et al., 2007
323.	RV-C	1	ADM08031.1	McIntyre et al., 2010; Simmonds et al., 2010
324.	RV-C	1	ADM08030.1	McIntyre et al., 2010; Simmonds et al., 2010
325.	RV-C	2	ABQ51392.1	http://www.picornastudygroup.com/types/enterovirus/hrv-c.htm; Kistler et al., 2007
326.	RV-C	2	AFJ68125.1	Benson et al., 2013
327.	RV-C	2	AET72466.1	Benson et al., 2013
328.	RV-C	3	ABO76708.1	http://www.picornastudygroup.com/types/enterovirus/hrv-c.htm; Tapparel et al., 2007
329.	RV-C	3	ADM08032.1	McIntyre et al., 2010; Simmonds et al., 2010
330.	RV-C	3	ABP38394.1	http://www.picornastudygroup.com/types/enterovirus/hrv-c.htm; McErlean et al., 2007
331.	RV-C	4	ABU62848.1	http://www.picornastudygroup.com/types/enterovirus/hrv-c.htm; Lau et al., 2007
332.	RV-C	4	AEG42396.1	Benson et al., 2013
333.	RV-C	4	YP_001552435	Lau et al., 2007
334.	RV-C	5	ABU62849.1	http://www.picornastudygroup.com/types/enterovirus/hrv-c.htm; Lau et al., 2007
335.	RV-C	5	AET25082	
336.	RV-C	6	ABU62850.1	http://www.picornastudygroup.com/types/enterovirus/hrv-c.htm; Lau et al., 2007
337.	RV-C	6	ADM08068.1	McIntyre et al., 2010; Simmonds et al., 2010
338.	RV-C	6	ADM08052.1	McIntyre et al., 2010; Simmonds et al., 2010
339.	RV-C	7	ABK29455.2	http://www.picornastudygroup.com/types/enterovirus/hrv-c.htm; Lamson et al., 2006
340.	RV-C	7	ADM08035.1	McIntyre et al., 2010; Simmonds et al., 2010
341.	RV-C	7	AER92557.1	Benson et al., 2013
342.	RV-C	8	ACU00185.1	http://www.picornastudygroup.com/types/enterovirus/hrv-c.htm; Huang et al., 2009
343.	RV-C	8	ADM08013.1	McIntyre et al., 2010; Simmonds et al., 2010
344.	RV-C	8	AFD64775.1	Benson et al., 2013
345.	RV-C	9	ACU00186.1	http://www.picornastudygroup.com/types/enterovirus/hrv-c.htm; Huang et al., 2009

346.	RV-C	9	ADM08082.1	McIntyre et al., 2010; Simmonds et al., 2010
347.	RV-C	9	ADM08076.1	McIntyre et al., 2010; Simmonds et al., 2010
348.	RV-C	10	ACV51798.1	http://www.picornastudygroup.com/types/enterovirus/hrv-c.htm; Arden et al., 2010
349.	RV-C	10	ADC42085.1	http://www.picornastudygroup.com/types/enterovirus/hrv-c.htm; Arden et al., 2010
350.	RV-C	10	ADC42086.1	http://www.picornastudygroup.com/types/enterovirus/hrv-c.htm; Arden et al., 2010
351.	RV-C	11	ACR14890.1	http://www.picornastudygroup.com/types/enterovirus/hrv-c.htm; Tapparel et al., 2009
352.	RV-C	11	ACN94256.1	McIntyre et al., 2010; Simmonds et al., 2010
353.	RV-C	12	ADM08073.1	http://www.picornastudygroup.com/types/enterovirus/hrv-c.htm; McIntyre et al., 2010
354.	RV-C	12	ADM08077.1	McIntyre et al., 2010; Simmonds et al., 2010
355.	RV-C	12	ADM08074.1	McIntyre et al., 2010; Simmonds et al., 2010
356.	RV-C	13	ADM08023.1	http://www.picornastudygroup.com/types/enterovirus/hrv-c.htm; ; McIntyre et al., 2010
357.	RV-C	14	ADM08026.1	http://www.picornastudygroup.com/types/enterovirus/hrv-c.htm; ; McIntyre et al., 2010
358.	RV-C	15	ADM08078.1	http://www.picornastudygroup.com/types/enterovirus/hrv-c.htm; ; McIntyre et al., 2010
359.	RV-C	15	ACZ67658.1	http://www.picornastudygroup.com/types/enterovirus/hrv-c.htm
360.	RV-C	16	ADM08059.1	http://www.picornastudygroup.com/types/enterovirus/hrv-c.htm; ; McIntyre et al., 2010
361.	RV-C	16	ADM08062.1	McIntyre et al., 2010; Simmonds et al., 2010
362.	RV-C	16	ADM08060.1	McIntyre et al., 2010; Simmonds et al., 2010
363.	RV-C	17	ADM08051.1	http://www.picornastudygroup.com/types/enterovirus/hrv-c.htm; ; McIntyre et al., 2010
364.	RV-C	17	AET72458.1	Benson et al., 2013
365.	RV-C	18	ADM08033.1	http://www.picornastudygroup.com/types/enterovirus/hrv-c.htm; ; McIntyre et al., 2010
366.	RV-C	18	ADM08071.1	McIntyre et al., 2010; Simmonds et al., 2010
367.	RV-C	18	ADM08070.1	McIntyre et al., 2010; Simmonds et al., 2010
368.	RV-C	19	ACU27058.1	http://www.picornastudygroup.com/types/enterovirus/hrv-c.htm; Tapparel et al., 2009 (enterovirus)
369.	RV-C	19	ADM08046.1	McIntyre et al., 2010; Simmonds et al., 2010
370.	RV-C	19	ADM08027.1	McIntyre et al., 2010; Simmonds et al., 2010
371.	RV-C	20	ADM08038.1	http://www.picornastudygroup.com/types/enterovirus/hrv-c.htm ; McIntyre et al., 2010
372.	RV-C	20	ADM08081.1	McIntyre et al., 2010; Simmonds et al., 2010
373.	RV-C	20	ADM08075.1	McIntyre et al., 2010; Simmonds et al., 2010
374.	RV-C	21	ADM08018.1	http://www.picornastudygroup.com/types/enterovirus/hrv-c.htm ; McIntyre et al., 2010
375.	RV-C	21	ADM08045.1	McIntyre et al., 2010; Simmonds et al., 2010
376.	RV-C	22	ADM08020.1	http://www.picornastudygroup.com/types/enterovirus/hrv-c.htm ; McIntyre et al., 2010
377.	RV-C	22	ADM08066.1	McIntyre et al., 2010; Simmonds et al., 2010
378.	RV-C	23	ADM08016.1	http://www.picornastudygroup.com/types/enterovirus/hrv-c.htm ; McIntyre et al., 2010
379.	RV-C	23	ADM08061.1	McIntyre et al., 2010; Simmonds et al., 2010
380.	RV-C	24	ADM08054.1	http://www.picornastudygroup.com/types/enterovirus/hrv-c.htm ; McIntyre et al., 2010
381.	RV-C	25	ADM08067.1	http://www.picornastudygroup.com/types/enterovirus/hrv-c.htm ; McIntyre et al., 2010

382.	RV-C	25	AEL31289.1	http://www.picornastudygroup.com/types/enterovirus/hrv-c.htm
383.	RV-C	25	AET25076.1	Benson et al., 2013
384.	RV-C	26	ADM08019.1	http://www.picornastudygroup.com/types/enterovirus/hrv-c.htm ; McIntyre et al., 2010
385.	RV-C	26	ADM08072.1	McIntyre et al., 2010; Simmonds et al., 2010
386.	RV-C	27	ADM08021.1	http://www.picornastudygroup.com/types/enterovirus/hrv-c.htm ; McIntyre et al., 2010
387.	RV-C	27	ADM08043.1	McIntyre et al., 2010; Simmonds et al., 2010
388.	RV-C	27	ADM08029.1	McIntyre et al., 2010; Simmonds et al., 2010
389.	RV-C	28	ADM08069.1	http://www.picornastudygroup.com/types/enterovirus/hrv-c.htm; ; McIntyre et al., 2010
390.	RV-C	28	AER92566.1	Benson et al., 2013
391.	RV-C	29	ADM08064.1	http://www.picornastudygroup.com/types/enterovirus/hrv-c.htm; ; McIntyre et al., 2010
392.	RV-C	30	ADM08083.1	http://www.picornastudygroup.com/types/enterovirus/hrv-c.htm; ; McIntyre et al., 2010
393.	RV-C	31	ADM08079.1	http://www.picornastudygroup.com/types/enterovirus/hrv-c.htm; ; McIntyre et al., 2010
394.	RV-C	32	ADM08012.1	http://www.picornastudygroup.com/types/enterovirus/hrv-c.htm; ; McIntyre et al., 2010
395.	RV-C	32	ADM08058.1	McIntyre et al., 2010; Simmonds et al., 2010
396.	RV-C	32	ADM08057.1	McIntyre et al., 2010; Simmonds et al., 2010
397.	RV-C	33	ADM08049.1	http://www.picornastudygroup.com/types/enterovirus/hrv-c.htm; ; McIntyre et al., 2010
398.	RV-C	33	ADM08080.1	McIntyre et al., 2010; Simmonds et al., 2010
399.	RV-C	34	AEP02607.1	http://www.picornastudygroup.com/types/enterovirus/hrv-c.htm; Chidlow et al., 2012
400.	RV-C	35	AEM76814.1	http://www.picornastudygroup.com/types/enterovirus/hrv-c.htm
401.	RV-C	36	AEM44635.1	http://www.picornastudygroup.com/types/enterovirus/hrv-c.htm; Lysholm et al., 2012
402.	RV-C	36	AEM44638.1	http://www.picornastudygroup.com/types/enterovirus/hrv-c.htm
403.	RV-C	36	AEM44640.1	http://www.picornastudygroup.com/types/enterovirus/hrv-c.htm
404.	RV-C	37	AEM44645.1	http://www.picornastudygroup.com/types/enterovirus/hrv-c.htm
405.	RV-C	38	AEM44646.1	http://www.picornastudygroup.com/types/enterovirus/hrv-c.htm
406.	RV-C	39	AFK79845.1	http://www.picornastudygroup.com/types/enterovirus/hrv-c.htm
407.	RV-C	39	AEM44630.1	http://www.picornastudygroup.com/types/enterovirus/hrv-c.htm
408.	RV-C	40	AEM44636.1	http://www.picornastudygroup.com/types/enterovirus/hrv-c.htm
409.	RV-C	40	AEM44637.1	http://www.picornastudygroup.com/types/enterovirus/hrv-c.htm
410.	RV-C	40	AEM44639.1	http://www.picornastudygroup.com/types/enterovirus/hrv-c.htm
411.	RV-C	41	AEM44629.1	http://www.picornastudygroup.com/types/enterovirus/hrv-c.htm
412.	RV-C	41	AEM44647.1	http://www.picornastudygroup.com/types/enterovirus/hrv-c.htm
413.	RV-C	41	AER92562.1	Benson et al., 2013
414.	RV-C	42	AEM44644.1	http://www.picornastudygroup.com/types/enterovirus/hrv-c.htm
415.	RV-C	43	AEM44631.1	http://www.picornastudygroup.com/types/enterovirus/hrv-c.htm
416.	RV-C	43	AEM44633.1	http://www.picornastudygroup.com/types/enterovirus/hrv-c.htm
417.	RV-C	43	AFK65743.1	Benson et al., 2013
418.	RV-C	44	AEM44634.1	http://www.picornastudygroup.com/types/enterovirus/hrv-c.htm
419.	RV-C	45	AEM44632.1	http://www.picornastudygroup.com/types/enterovirus/hrv-c.htm
420.	RV-C	45	AET25077.1	Benson et al., 2013
421.	RV-C	46	AEM44642.1	http://www.picornastudygroup.com/types/enterovirus/hrv-c.htm
422.	RV-C	46	AEM44643.1	http://www.picornastudygroup.com/types/enterovirus/hrv-c.htm
423.	RV-C	47	AEP02608.1	http://www.picornastudygroup.com/types/enterovirus/hrv-c.htm
424.	RV-C	48	AEP02609.1	http://www.picornastudygroup.com/types/enterovirus/hrv-c.htm
425.	RV-C	49	AEQ61492.1	http://www.picornastudygroup.com/types/enterovirus/hrv-c.htm

426.	RV-C	49	AER92563.1	Benson et al., 2013
427.	RV-C	49	AER92565	Benson et al., 2013
428.	RV-C	50	AFI25010.1	http://www.picornastudygroup.com/types/enterovirus/hrv-c.htm
429.	RV-C	50	AHA35211	http://www.picornastudygroup.com/types/enterovirus/hrv-c.htm
430.	RV-C	51	AEL31291.1	http://www.picornastudygroup.com/types/enterovirus/hrv-c.htm
431.	RV-C	51	AFS30772.1	http://www.picornastudygroup.com/types/enterovirus/hrv-c.htm
432.	RV-C	54	AJF45985	http://www.picornastudygroup.com/types/enterovirus/hrv-c.htm
