# Supplementary material for: RV-Typer: A Web Server for Typing of Rhinoviruses Using Alignment-Free Approach
Source: PLoS One. 2016 Feb 12;11(2):e0149350. doi: 10.1371/journal.pone.0149350 (PMC4752186; doi:10.1371/journal.pone.0149350)
Supplement: S2 Table — (PDF) [file pone.0149350.s008.pdf]

RV-Typer: a web server for serotyping of *Rhinoviruses* using alignment-free approach

Pandurang Kolekar[1], Vaishali Waman[1], Mohan Kale[2] and Urmila Kulkarni-Kale [1§].

[1]Bioinformatics Centre, Savitribai Phule Pune University (formerly University of Pune), Pune 411 007, India.
[2]Department of Statistics, Savitribai Phule Pune University (formerly University of Pune), Pune 411 007, India.
[§]Corresponding author

Table S2: The true positive data set of 218 VP1 protein sequences of serotypes of *Rhinoviruses* (RV) and their GenPept accession numbers used in this study.

Sr. no.	Species	Serotype	GenPept Accession no.
1.	RV-A	1A	AKF02545
2.	RV-A	1A	AKF02546
3.	RV-A	1A	AKF02547
4.	RV-A	1A	AKF02548
5.	RV-A	1B	AKF02549
6.	RV-A	1B	AKF02550
7.	RV-A	1B	AKF02551
8.	RV-A	1B	AKF02552
9.	RV-A	10	AER92572
10.	RV-A	10	AER92579
11.	RV-A	12	AEP14937
12.	RV-A	15	AEP69138
13.	RV-A	16	AEP69152
14.	RV-A	16	AER92571
15.	RV-A	16	AET72471
16.	RV-A	16	AFK65744
17.	RV-A	18	AEG42384
18.	RV-A	18	AEG42395
19.	RV-A	19	AFD33440
20.	RV-A	19	AFD33444
21.	RV-A	20	AER92568
22.	RV-A	20	AFJ68116
23.	RV-A	21	AET25084
24.	RV-A	21	AFD33441
25.	RV-A	22	AHV79179
26.	RV-A	23	AET72472
27.	RV-A	24	AER92560
28.	RV-A	28	AER92577
29.	RV-A	28	AFD33445
30.	RV-A	29	ACK37381
31.	RV-A	33	AET85050
32.	RV-A	34	ABF51195
33.	RV-A	34	AEG42397
34.	RV-A	36	AEG42385

35.	RV-A	36	AEP69154
36.	RV-A	36	AEP69160
37.	RV-A	36	AER92581
38.	RV-A	36	AET72459
39.	RV-A	36	AET72460
40.	RV-A	36	AET72464
41.	RV-A	36	AFK65737
42.	RV-A	36	AHK24835
43.	RV-A	38	AFJ68118
44.	RV-A	40	AER92576
45.	RV-A	40	AFD64769
46.	RV-A	40	AFK65738
47.	RV-A	43	AET72455
48.	RV-A	44	AET72470
49.	RV-A	49	AER92559
50.	RV-A	51	AEP69148
51.	RV-A	53	AER92584
52.	RV-A	55	AFG70521
53.	RV-A	58	AFJ68126
54.	RV-A	59	AEP69136
55.	RV-A	60	AER92587
56.	RV-A	61	AER92558
57.	RV-A	65	AEG42391
58.	RV-A	65	AFD64768
59.	RV-A	66	AEN04071
60.	RV-A	66	AEP69163
61.	RV-A	66	AFG70518
62.	RV-A	67	AEP69162
63.	RV-A	76	AET72456
64.	RV-A	76	AFK65736
65.	RV-A	76	AFK65742
66.	RV-A	80	AER92573
67.	RV-A	80	AER92583
68.	RV-A	80	AET85048
69.	RV-A	82	AER92582
70.	RV-A	82	AFG70525
71.	RV-A	89	AFG70519
72.	RV-A	89	AFG70522
73.	RV-A	89	CAA00931
74.	RV-A	89	NP042288
75.	RV-A	10	AAR29605
76.	RV-A	13	AAR29608
77.	RV-A	20	ACK37376
78.	RV-A	20	AAR29612
79.	RV-A	21	AAQ19854

80.	RV-A	23	ABF51191
81.	RV-A	2	P04936.3
82.	RV-A	30	ABF51206
83.	RV-A	30	AAR21607
84.	RV-A	31	AAR21608
85.	RV-A	33	AAR29620
86.	RV-A	34	AAR29621
87.	RV-A	36	ABF51199
88.	RV-A	36	AAR29622
89.	RV-A	38	AAR29623
90.	RV-A	44	AAR21609
91.	RV-A	47	AAP48734
92.	RV-A	47	AAR21610
93.	RV-A	49	AAP48739
94.	RV-A	49	ABF51190
95.	RV-A	49	AAR21611
96.	RV-A	49	AER92586
97.	RV-A	54	AAR29633
98.	RV-A	62	AAR21612
99.	RV-A	68	ACK37406
100.	RV-A	76	AAR29651
101.	RV-A	7	AAR29654
102.	RV-A	81	ACK37413
103.	RV-A	81	AAR29656
104.	RV-A	89	ACK37421
105.	RV-A	89	NP_740394
106.	RV-A	89	AAA45762
107.	RV-A	89	NP_042288
108.	RV-A	9	AAR29666
109.	RV-A	9	ACK37371
110.	RV-B	103	AFG70520
111.	RV-B	103	AFJ68119
112.	RV-B	14	CAA25565
113.	RV-B	14	NP_740518
114.	RV-B	35	ABF51181
115.	RV-B	35	ACK37443
116.	RV-B	3	AAR29667
117.	RV-B	42	AEG42386
118.	RV-B	42	AEG42394
119.	RV-B	42	AEP69147
120.	RV-B	48	AET85041
121.	RV-B	4	AER92570
122.	RV-B	52	AHK24836
123.	RV-B	69	AFD64772
124.	RV-B	6	AEP69146

125.	RV-B	6	AET72461
126.	RV-B	6	AFD33439
127.	RV-B	6	AFD33442
128.	RV-B	6	AFG70524
129.	RV-B	6	AFM84628
130.	RV-B	70	AFD64776
131.	RV-B	72	ACK37409
132.	RV-B	72	AAR29681
133.	RV-B	72	AET81010
134.	RV-B	72	AFD64771
135.	RV-B	72	AHK24837
136.	RV-B	83	AET85044
137.	RV-B	84	AEG42387
138.	RV-B	84	AEG42389
139.	RV-B	84	AEP69141
140.	RV-B	84	AEP69151
141.	RV-B	84	AFG70526
142.	RV-C	1	ADM08034
143.	RV-C	1	ADV57353
144.	RV-C	2	AET85046
145.	RV-C	2	AET25086
146.	RV-C	2	AFD64770
147.	RV-C	2	AHK24838
148.	RV-C	3	ABP38395
149.	RV-C	3	ABP38396
150.	RV-C	3	ABP38397
151.	RV-C	3	ABP38398
152.	RV-C	3	ABP38399
153.	RV-C	3	ABP38400
154.	RV-C	3	ABP38401
155.	RV-C	3	ABP38402
156.	RV-C	3	ABP38403
157.	RV-C	3	ABP38404
158.	RV-C	3	ABP38405
159.	RV-C	3	ABP38406
160.	RV-C	3	ABP38407
161.	RV-C	3	ABP38408
162.	RV-C	3	ABP38409
163.	RV-C	3	ABP38410
164.	RV-C	3	AER92564
165.	RV-C	3	AET85043
166.	RV-C	4	YP_001552411
167.	RV-C	6	ACH72972
168.	RV-C	6	ADM08014
169.	RV-C	6	ADM08015

170.	RV-C	6	ADM08024
171.	RV-C	6	ADM08025
172.	RV-C	6	ADM08028
173.	RV-C	6	ADM08047
174.	RV-C	6	ADM08048
175.	RV-C	6	AET72463
176.	RV-C	6	AET85045
177.	RV-C	6	AHD24522
178.	RV-C	7	AER92567
179.	RV-C	7	AET25080
180.	RV-C	7	AFJ68117
181.	RV-C	7	AFJ68124
182.	RV-C	8	AFD64766
183.	RV-C	9	ADM08042
184.	RV-C	9	ADM08050
185.	RV-C	10	ACH72971
186.	RV-C	10	ADC42087
187.	RV-C	10	ADC42088
188.	RV-C	10	ADC42089
189.	RV-C	10	ADC42090
190.	RV-C	10	ADC42091
191.	RV-C	15	AET25079
192.	RV-C	17	AFG70523
193.	RV-C	18	ADM08063
194.	RV-C	18	ADM08065
195.	RV-C	20	ADM08040
196.	RV-C	20	ADM08041
197.	RV-C	20	ADM08044
198.	RV-C	22	AEP69159
199.	RV-C	26	AFM84629
200.	RV-C	27	ADM08022
201.	RV-C	32	ADM08017
202.	RV-C	32	ADM08036
203.	RV-C	32	ADM08037
204.	RV-C	32	ADM08039
205.	RV-C	32	ADM08053
206.	RV-C	32	ADM08055
207.	RV-C	32	ADM08056
208.	RV-C	32	AER92578
209.	RV-C	32	AFJ68120
210.	RV-C	36	AEM44641
211.	RV-C	36	AEP69137
212.	RV-C	40	AEG42392
213.	RV-C	40	AET72469
214.	RV-C	40	AFD64765

215.	RV-C	41	AHK24839
216.	RV-C	42	AFJ68122
217.	RV-C	43	AET25078
218.	RV-C	49	AEE69369.1
