# Supplementary material for: RV-Typer: A Web Server for Typing of Rhinoviruses Using Alignment-Free Approach
Source: PLoS One. 2016 Feb 12;11(2):e0149350. doi: 10.1371/journal.pone.0149350 (PMC4752186; doi:10.1371/journal.pone.0149350)
Supplement: S3 Table — (PDF) [file pone.0149350.s009.pdf]

RV-Typer: a web server for serotyping of *Rhinoviruses* using alignment-free approach

Pandurang Kolekar[1], Vaishali Waman[1], Mohan Kale[2] and Urmila Kulkarni-Kale [1§].

[1]Bioinformatics Centre, Savitribai Phule Pune University (formerly University of Pune), Pune 411 007, India.
[2]Department of Statistics, Savitribai Phule Pune University (formerly University of Pune), Pune 411 007, India.
[§]Corresponding author

Table S3: The negative data set of 7101 protein sequences and their GenPept accession numbers used in this study.

Sr.No.	GenPept accession number (and optional sequence start-end positions)	Virus Species
1.	ACK37367.1	Human rhinovirus 1
2.	ABF51179.1	Human rhinovirus 3
3.	ABF51184.1	Human rhinovirus 4
4.	ACK37368.1	Human rhinovirus 5
5.	ABF51180.1	Human rhinovirus 6
6.	ACK37432.1	Human rhinovirus 7
7.	ACK37369.1	Human rhinovirus 8
8.	ACK37370.1	Human rhinovirus 9
9.	ACK37371.1	Human rhinovirus 9
10.	ACK37433.1	Human rhinovirus 9
11.	ACK37434.1	Human rhinovirus 10
12.	ABO69370.1	Human rhinovirus 11
13.	ABO69371.1	Human rhinovirus 12
14.	ACK37372.1	Human rhinovirus 13
15.	ACK37373.1	Human rhinovirus 13
16.	ABF51187.1	Human rhinovirus 15
17.	AAA69862.1	Human rhinovirus 16
18.	ABO69376.1	Human rhinovirus 17
19.	ACK37374.1	Human rhinovirus 18
20.	ACK37375.1	Human rhinovirus 19
21.	ACK37376.1	Human rhinovirus 20
22.	ACK37377.1	Human rhinovirus 21
23.	ACK37378.1	Human rhinovirus 22
24.	ABF51191.1	Human rhinovirus 23
25.	ACK37446.1	Human rhinovirus 24
26.	ACK37379.1	Human rhinovirus 25
27.	ACK37380.1	Human rhinovirus 26
28.	ACK37442.1	Human rhinovirus 27
29.	ACK37381.1	Human rhinovirus 29
30.	ACK37435.1	Human rhinovirus 30
31.	ACK37382.1	Human rhinovirus 31
32.	ACK37382.1	Human rhinovirus 31
33.	ACK37383.1	Human rhinovirus 32

34.	ACK37384.1	Human rhinovirus 33
35.	ACK37445.1	Human rhinovirus 34
36.	ACK37443.1	Human rhinovirus 35
37.	ABF51199.1	Human rhinovirus 36
38.	ABO69379.1	Human rhinovirus 37
39.	ACK37436.1	Human rhinovirus 38
40.	AAV27300.1	Human rhinovirus 39
41.	ACK37385.1	Human rhinovirus 40
42.	ABF51185.1	Human rhinovirus 41
43.	ACK37386.1	Human rhinovirus 42
44.	ACK37387.1	Human rhinovirus 43
45.	ABF51193.1	Human rhinovirus 44
46.	ACK37388.1	Human rhinovirus 45
47.	ABF51200.1	Human rhinovirus 46
48.	ACK37389.1	Human rhinovirus 47
49.	ACK37390.1	Human rhinovirus 49
50.	ACK37391.1	Human rhinovirus 50
51.	ACK37392.1	Human rhinovirus 51
52.	ACK37393.1	Human rhinovirus 52
53.	ACK37444.1	Human rhinovirus 52
54.	ACK37394.1	Human rhinovirus 54
55.	ACK37395.1	Human rhinovirus 54
56.	ABF51205.1	Human rhinovirus 55
57.	ACK37396.1	Human rhinovirus 56
58.	ACK37397.1	Human rhinovirus 57
59.	ACK37398.1	Human rhinovirus 58
60.	ABF51194.1	Human rhinovirus 59
61.	ACK37399.1	Human rhinovirus 60
62.	ACK37400.1	Human rhinovirus 61
63.	ACK37401.1	Human rhinovirus 62
64.	ACK37402.1	Human rhinovirus 63
65.	ACK37437.1	Human rhinovirus 64
66.	ACK37403.1	Human rhinovirus 65
67.	ACK37404.1	Human rhinovirus 66
68.	ACK37405.1	Human rhinovirus 67
69.	ACK37406.1	Human rhinovirus 68
70.	ACK37407.1	Human rhinovirus 69
71.	ABF51183.1	Human rhinovirus 70
72.	ACK37408.1	Human rhinovirus 71
73.	ACK37409.1	Human rhinovirus 72
74.	ABF51186.1	Human rhinovirus 73
75.	ABF51188.1	Human rhinovirus 74
76.	ABF51204.1	Human rhinovirus 75
77.	ACK37438.1	Human rhinovirus 76

78.	ACK37410.1	Human rhinovirus 77
79.	ACK37439.1	Human rhinovirus 78
80.	ACK37411.1	Human rhinovirus 79
81.	ACK37412.1	Human rhinovirus 80
82.	ACK37413.1	Human rhinovirus 81
83.	ACK37414.1	Human rhinovirus 81
84.	ACK37415.1	Human rhinovirus 81
85.	ACK37416.1	Human rhinovirus 82
86.	ACK37417.1	Human rhinovirus 83
87.	ACK37418.1	Human rhinovirus 84
88.	ACK37419.1	Human rhinovirus 85
89.	ACK37420.1	Human rhinovirus 86
90.	ACK37421.1	Human rhinovirus 89
91.	ACK37422.1	Human rhinovirus 89
92.	ACK37440.1	Human rhinovirus 89
93.	ACK37423.1	Human rhinovirus 90
94.	ACK37424.1	Human rhinovirus 91
95.	ACK37425.1	Human rhinovirus 92
96.	ABO69381.1	Human rhinovirus 93
97.	ACK37441.1	Human rhinovirus 94
98.	ACK37426.1	Human rhinovirus 95
99.	ACK37427.1	Human rhinovirus 96
100.	ACK37428.1	Human rhinovirus 97
101.	ACK37429.1	Human rhinovirus 98
102.	ACK37430.1	Human rhinovirus 99
103.	ACK37431.1	Human rhinovirus 100
104.	ABQ16587.1	Human rhinovirus NAT001 (TYPE 1)
105.	ABQ51392.1	Human rhinovirus NAT045 (type 2
106.	ABO76708.1	Human rhinovirus QPM (type 3)
107.	ABU62849.1	Human rhinovirus C (type 5)
108.	ABU62850.1	Human rhinovirus C (type 6)
109.	ABK29455.2	Human rhinovirus C (type 7)
110.	ACU00185.1	Human rhinovirus C (type 8
111.	ACU00186.1	Human rhinovirus C (type 9
112.	ACV51798.1	Human rhinovirus C strain QCE (type 10)
113.	ACR14890.2	Human rhinovirus C (type 11)
114.	ACK37367.1	Human rhinovirus 1
115.	ABF51179.1	Human rhinovirus 3
116.	ABF51184.1	Human rhinovirus 4
117.	ACK37368.1	Human rhinovirus 5
118.	ABF51180.1	Human rhinovirus 6
119.	ACK37432.1	Human rhinovirus 7
120.	ACK37369.1	Human rhinovirus 8
121.	ACK37370.1	Human rhinovirus 9

122.	ACK37371.1	Human rhinovirus 9
123.	ACK37433.1	Human rhinovirus 9
124.	ACK37434.1	Human rhinovirus 10
125.	ABO69371.1	Human rhinovirus 12
126.	ACK37372.1	Human rhinovirus 13
127.	ACK37373.1	Human rhinovirus 13
128.	ABF51187.1	Human rhinovirus 15
129.	AAA69862.1	Human rhinovirus 16
130.	AY436687.1	Human rhinovirus 17
131.	ACK37374.1	Human rhinovirus 18
132.	ACK37375.1	Human rhinovirus 19
133.	ACK37376.1	Human rhinovirus 20
134.	ACK37377.1	Human rhinovirus 21
135.	ACK37378.1	Human rhinovirus 22
136.	ABF51191.1	Human rhinovirus 23
137.	ACK37446.1	Human rhinovirus 24
138.	ACK37379.1	Human rhinovirus 25
139.	ACK37380.1	Human rhinovirus 26
140.	ACK37442.1	Human rhinovirus 27
141.	ACK37381.1	Human rhinovirus 29
142.	ACK37435.1	Human rhinovirus 30
143.	ACK37382.1	Human rhinovirus 31
144.	ACK37382.1	Human rhinovirus 31
145.	ACK37383.1	Human rhinovirus 32
146.	ACK37384.1	Human rhinovirus 33
147.	ACK37445.1	Human rhinovirus 34
148.	ACK37443.1	Human rhinovirus 35
149.	ABF51199.1	Human rhinovirus 36
150.	ABO69379.1	Human rhinovirus 37
151.	ACK37436.1	Human rhinovirus 38
152.	AAV27300.1	Human rhinovirus 39
153.	ACK37385.1	Human rhinovirus 40
154.	ABF51185.1	Human rhinovirus 41
155.	ACK37386.1	Human rhinovirus 42
156.	ACK37387.1	Human rhinovirus 43
157.	ABF51193.1	Human rhinovirus 44
158.	ACK37388.1	Human rhinovirus 45
159.	ABF51200.1	Human rhinovirus 46
160.	ACK37389.1	Human rhinovirus 47
161.	ACK37390.1	Human rhinovirus 49
162.	ACK37391.1	Human rhinovirus 50
163.	ACK37392.1	Human rhinovirus 51
164.	ACK37393.1	Human rhinovirus 52
165.	ACK37444.1	Human rhinovirus 52

166.	ACK37394.1	Human rhinovirus 54
167.	ACK37395.1	Human rhinovirus 54
168.	ABF51205.1	Human rhinovirus 55
169.	ACK37396.1	Human rhinovirus 56
170.	ACK37397.1	Human rhinovirus 57
171.	ACK37398.1	Human rhinovirus 58
172.	ABF51194.1	Human rhinovirus 59
173.	ACK37399.1	Human rhinovirus 60
174.	ACK37400.1	Human rhinovirus 61
175.	ACK37401.1	Human rhinovirus 62
176.	ACK37402.1	Human rhinovirus 63
177.	ACK37437.1	Human rhinovirus 64
178.	ACK37403.1	Human rhinovirus 65
179.	ACK37404.1	Human rhinovirus 66
180.	ACK37405.1	Human rhinovirus 67
181.	ACK37406.1	Human rhinovirus 68
182.	ACK37407.1	Human rhinovirus 69
183.	ABF51183.1	Human rhinovirus 70
184.	ACK37408.1	Human rhinovirus 71
185.	ACK37409.1	Human rhinovirus 72
186.	ABF51186.1	Human rhinovirus 73
187.	ABF51188.1	Human rhinovirus 74
188.	ABF51204.1	Human rhinovirus 75
189.	ACK37438.1	Human rhinovirus 76
190.	ACK37410.1	Human rhinovirus 77
191.	ACK37439.1	Human rhinovirus 78
192.	ACK37411.1	Human rhinovirus 79
193.	ACK37412.1	Human rhinovirus 80
194.	ACK37413.1	Human rhinovirus 81
195.	ACK37414.1	Human rhinovirus 81
196.	ACK37415.1	Human rhinovirus 81
197.	ACK37416.1	Human rhinovirus 82
198.	ACK37417.1	Human rhinovirus 83
199.	ACK37418.1	Human rhinovirus 84
200.	ACK37419.1	Human rhinovirus 85
201.	ACK37420.1	Human rhinovirus 86
202.	ACK37421.1	Human rhinovirus 89
203.	ACK37422.1	Human rhinovirus 89
204.	ACK37440.1	Human rhinovirus 89
205.	ACK37423.1	Human rhinovirus 90
206.	ACK37424.1	Human rhinovirus 91
207.	ACK37425.1	Human rhinovirus 92
208.	ABO69381.1	Human rhinovirus 93
209.	ACK37441.1	Human rhinovirus 94

210.	ACK37426.1	Human rhinovirus 95
211.	ACK37427.1	Human rhinovirus 96
212.	ACK37428.1	Human rhinovirus 97
213.	ACK37429.1	Human rhinovirus 98
214.	ACK37430.1	Human rhinovirus 99
215.	ACK37431.1	Human rhinovirus 100
216.	ABQ16587.1	Human rhinovirus NAT001 (type 1)
217.	ABQ51392.1	Human rhinovirus NAT045 (type 2)
218.	ABO76708.1	Human rhinovirus QPM (type 3)
219.	ABU62848.1	Human rhinovirus C (type 4)
220.	ABU62849.1	Human rhinovirus C (type 5)
221.	ABU62850.1	Human rhinovirus C (type 6)
222.	ABK29455.2	Human rhinovirus C (type 7)
223.	ACU00185.1	Human rhinovirus C (type 8)
224.	ACU00186.1	Human rhinovirus C (type 9)
225.	ACV51798.1	Human rhinovirus C strain QCE (type 10)
226.	ACR14890.2	Human rhinovirus C (type 11)
227.	ABO69381.1	Human rhinovirus 93
228.	ABO69379.1	Human rhinovirus 37
229.	ABO69376.1	Human rhinovirus 17
230.	ABO76708.1	Human rhinovirus QPM (type 1)
231.	ABU62848.1	Human rhinovirus C (type 4)
232.	ABU62849.1	Human rhinovirus C (type 5)
233.	ABU62850.1	Human rhinovirus C (type 6)
234.	ABK29455.2	Human rhinovirus C (type 7)
235.	ABO76708.1	Human rhinovirus QPM (type 3)
236.	ABU62848.1	Human rhinovirus C (type 4)
237.	ABU62849.1	Human rhinovirus C (type 5)
238.	ABU62850.1	Human rhinovirus C (type 6)
239.	ABK29455.2	Human rhinovirus C (type 7)
240.	AAF05693.1	Bovine enterovirus strain K2577
241.	AAF05693.1	Bovine enterovirus strain K2577
242.	AAF05693.1	Bovine enterovirus strain K2577
243.	AAF05693.1	Bovine enterovirus strain K2577
244.	AAU89432	Human poliovirus 1
245.	AAU89431	Human poliovirus 1
246.	AAU89430	Human poliovirus 1
247.	AAU89429	Human poliovirus 1
248.	AAU89428	Human poliovirus 1
249.	AAU89427	Human poliovirus 1
250.	AAU89426	Human poliovirus 1
251.	AAU89425	Human poliovirus 1
252.	AAU89424	Human poliovirus 1
253.	AAU89423	Human poliovirus 1

254.	AAU89422	Human poliovirus 1
255.	AAU89421	Human poliovirus 1
256.	AAU89420	Human poliovirus 1
257.	AAU89419	Human poliovirus 1
258.	AAU89418	Human poliovirus 1
259.	AAU89417	Human poliovirus 1
260.	AAU89416	Human poliovirus 1
261.	AAU89415	Human poliovirus 1
262.	AAU89414	Human poliovirus 1
263.	AAU89413	Human poliovirus 1
264.	AAU89412	Human poliovirus 1
265.	AAU89411	Human poliovirus 1
266.	AAU89410	Human poliovirus 1
267.	AAU89409	Human poliovirus 1
268.	AAU89408	Human poliovirus 1
269.	AAU89407	Human poliovirus 1
270.	AAU89406	Human poliovirus 1
271.	AAU89405	Human poliovirus 1
272.	AAU89404	Human poliovirus 1
273.	AAU89403	Human poliovirus 1
274.	AAU89402	Human poliovirus 1
275.	AAU89401	Human poliovirus 1
276.	AAU89400	Human poliovirus 1
277.	AAU89399	Human poliovirus 1
278.	AAU89398	Human poliovirus 1
279.	AAU89397	Human poliovirus 1
280.	AAU89396	Human poliovirus 1
281.	AAU89395	Human poliovirus 1
282.	AAU89394	Human poliovirus 1
283.	AAU89393	Human poliovirus 1
284.	AAF35025	Human poliovirus 1
285.	AAF35024	Human poliovirus 1
286.	AAF35023	Human poliovirus 1
287.	AAF35022	Human poliovirus 1
288.	AAF35021	Human poliovirus 1
289.	AAF35020	Human poliovirus 1
290.	AAF35019	Human poliovirus 1
291.	AAF35018	Human poliovirus 1
292.	AAF35017	Human poliovirus 1
293.	AAF35016	Human poliovirus 1
294.	AAF35015	Human poliovirus 1
295.	AAF35014	Human poliovirus 1
296.	AAF35013	Human poliovirus 1
297.	AAF35012	Human poliovirus 1

298.	AAF35011	Human poliovirus 1
299.	AAF35010	Human poliovirus 1
300.	AAF35009	Human poliovirus 1
301.	AAF35008	Human poliovirus 1
302.	AAF35007	Human poliovirus 1
303.	AAF35006	Human poliovirus 1
304.	AAF35005	Human poliovirus 1
305.	AAF35004	Human poliovirus 1
306.	AAF35003	Human poliovirus 1
307.	AAF35002	Human poliovirus 1
308.	CAB65072	Human poliovirus 1
309.	CAB65071	Human poliovirus 1
310.	CAB65070	Human poliovirus 1
311.	CAB65069	Human poliovirus 1
312.	CAB65068	Human poliovirus 1
313.	CAB65067	Human poliovirus 1
314.	CAB65066	Human poliovirus 1
315.	CAB65065	Human poliovirus 1
316.	CAB65064	Human poliovirus 1
317.	CAB65063	Human poliovirus 1
318.	CAB65062	Human poliovirus 1
319.	CAB65061	Human poliovirus 1
320.	CAB65060	Human poliovirus 1
321.	CAB65059	Human poliovirus 1
322.	CAB65058	Human poliovirus 1
323.	AAD17701	Human coxsackievirus A6
324.	BAL14611	Human enterovirus 71
325.	BAL14610	Human enterovirus 71
326.	BAL14609	Human enterovirus 71
327.	BAL14608	Human enterovirus 71
328.	BAL14607	Human enterovirus 71
329.	BAL14606	Human enterovirus 71
330.	BAL14605	Human enterovirus 71
331.	BAL14604	Human enterovirus 71
332.	BAL14603	Human enterovirus 71
333.	BAL14602	Human enterovirus 71
334.	BAL14601	Human enterovirus 71
335.	BAL14600	Human enterovirus 71
336.	BAL14599	Human enterovirus 71
337.	BAL14596	Human enterovirus 71
338.	BAL14595	Human enterovirus 71
339.	BAL14594	Human enterovirus 71
340.	BAL14592	Human enterovirus 71
341.	BAL14591	Human enterovirus 71

342.	BAL14590	Human enterovirus 71
343.	BAL14589	Human enterovirus 71
344.	BAL14586	Human enterovirus 71
345.	BAL14585	Human enterovirus 71
346.	BAL14598	Human enterovirus 71
347.	BAL14597	Human enterovirus 71
348.	BAL14593	Human enterovirus 71
349.	BAL14588	Human enterovirus 71
350.	BAL14587	Human enterovirus 71
351.	AAV74198	Human echovirus 13
352.	AAD17753	Human enterovirus 69
353.	AAD17752	Human enterovirus 68
354.	AAD17751	Echovirus 34
355.	AAD17750	Human echovirus 33
356.	AAD17749	Human echovirus 32
357.	AAD17748	Human echovirus 31
358.	AAD17747	Human echovirus 30
359.	AAD17746	Human echovirus 30
360.	AAD17745	Human echovirus 30
361.	AAD17744	Human echovirus 30
362.	AAD17743	Human echovirus 29
363.	AAD17742	Human echovirus 27
364.	AAD17741	Human echovirus 26
365.	AAD17740	Human echovirus 25
366.	AAD17739	Human echovirus 24
367.	AAD17738	Human echovirus 21
368.	AAD17737	Human echovirus 20
369.	AAD17736	Human echovirus 19
370.	AAD17735	Human echovirus 18
371.	AAD17734	Human echovirus 17
372.	AAD17733	Human echovirus 15
373.	AAD17732	Human echovirus 14
374.	AAD17731	Human echovirus 13
375.	AAD17730	Human echovirus 11
376.	AAD17729	Human echovirus 8
377.	AAD17728	Human echovirus 7
378.	AAD17727	Human echovirus 6
379.	AAD17726	Human echovirus 6
380.	AAD17725	Human echovirus 6
381.	AAD17724	Human echovirus 5
382.	AAD17723	Human echovirus 4
383.	AAD17722	Human echovirus 4
384.	AAD17721	Human echovirus 4
385.	AAD17720	Human echovirus 3

386.	AAD17719	Human echovirus 2
387.	AAD17718	Human echovirus 1
388.	AAD17717	Human coxsackievirus B6
389.	AAD17716	Human coxsackievirus B2
390.	AAD17715	Human coxsackievirus A24
391.	AAD17714	Human coxsackievirus A22
392.	AAD17713	Human coxsackievirus A20
393.	AAD17712	Human coxsackievirus A19
394.	AAD17711	Human coxsackievirus A18
395.	AAD17710	Human coxsackievirus A17
396.	AAD17709	Human coxsackievirus A15
397.	AAD17708	Human coxsackievirus A14
398.	AAD17707	Human coxsackievirus A13
399.	AAD17706	Human coxsackievirus A12
400.	AAD17705	Human coxsackievirus A11
401.	AAD17704	Human coxsackievirus A10
402.	AAD17703	Human coxsackievirus A8
403.	AAD17702	Human coxsackievirus A7
404.	AAD17700	Human coxsackievirus A5
405.	AAD17699	Human coxsackievirus A4
406.	AAD17698	Human coxsackievirus A3
407.	AAD17697	Human coxsackievirus A1
408.	BAK64189	Human enterovirus 71
409.	BAK64188	Human enterovirus 71
410.	BAK64187	Human enterovirus 71
411.	BAK64186	Human enterovirus 71
412.	BAK64185	Human enterovirus 71
413.	BAK64184	Human enterovirus 71
414.	BAK64183	Human enterovirus 71
415.	BAK64182	Human enterovirus 71
416.	BAK64181	Human enterovirus 71
417.	BAK64180	Human enterovirus 71
418.	BAK64179	Human enterovirus 71
419.	BAK64178	Human enterovirus 71
420.	BAK64177	Human enterovirus 71
421.	BAK64176	Human enterovirus 71
422.	BAK64175	Human enterovirus 71
423.	BAK64174	Human enterovirus 71
424.	BAK64173	Human enterovirus 71
425.	BAK64172	Human enterovirus 71
426.	BAK64171	Human enterovirus 71
427.	BAK64170	Human enterovirus 71
428.	BAK64169	Human enterovirus 71
429.	BAK64168	Human enterovirus 71

430.	BAK64167	Human enterovirus 71
431.	BAK64166	Human enterovirus 71
432.	BAK20495	Human echovirus 30
433.	BAK20494	Human echovirus 30
434.	BAK20493	Human echovirus 30
435.	BAK20492	Human echovirus 30
436.	BAK20491	Human echovirus 30
437.	ABK51760	Human enterovirus 71
438.	ABK51759	Human enterovirus 71
439.	AAV74199	Human echovirus 13
440.	AAU05387	Human echovirus 30
441.	AAU05386	Human echovirus 30
442.	AAU05385	Human echovirus 30
443.	AAU05384	Human echovirus 30
444.	BAJ07635	Human enterovirus 71
445.	BAJ07634	Human enterovirus 71
446.	BAJ07633	Human enterovirus 71
447.	BAJ07632	Human enterovirus 71
448.	BAJ07631	Human enterovirus 71
449.	BAJ07630	Human enterovirus 71
450.	AAF43593	Untyped enterovirus VA86-6765
451.	AAF18370	Human echovirus 30
452.	AAF18369	Human echovirus 30
453.	AAF18368	Human echovirus 30
454.	AAF18367	Human echovirus 30
455.	AAF18366	Human echovirus 30
456.	AAF18365	Human echovirus 30
457.	AAF18364	Human echovirus 30
458.	AAF18363	Human echovirus 30
459.	AAF18362	Human echovirus 30
460.	AAF18361	Human echovirus 30
461.	AAF18360	Human echovirus 30
462.	AAF18359	Human echovirus 30
463.	AAF18358	Human echovirus 30
464.	AAF18357	Human echovirus 30
465.	AAF18356	Human echovirus 30
466.	AAF18355	Human echovirus 30
467.	AAF18354	Human echovirus 30
468.	AAF18353	Human echovirus 30
469.	AAF18352	Human echovirus 30
470.	AAF18351	Human echovirus 30
471.	AAF18350	Human echovirus 30
472.	AAF18349	Human echovirus 30
473.	AAF18348	Human echovirus 30

474.	AAF18347	Human echovirus 30
475.	AAF18346	Human echovirus 30
476.	AAF18345	Human echovirus 30
477.	AAF13593	Human enterovirus 71
478.	AAF13592	Human enterovirus 71
479.	AAF13591	Human enterovirus 71
480.	AAF13590	Human enterovirus 71
481.	AAF13589	Human enterovirus 71
482.	AAF13588	Human enterovirus 71
483.	AAF13587	Human enterovirus 71
484.	AAF13586	Human enterovirus 71
485.	AAF13585	Human enterovirus 71
486.	AAF13584	Human enterovirus 71
487.	AAF13583	Human enterovirus 71
488.	AAF13582	Human enterovirus 71
489.	AAF13581	Human enterovirus 71
490.	AAF13580	Human enterovirus 71
491.	AAF13579	Human enterovirus 71
492.	AAF13578	Human enterovirus 71
493.	AAF13577	Human enterovirus 71
494.	AAF13576	Human enterovirus 71
495.	AAF13575	Human enterovirus 71
496.	AAF13574	Human enterovirus 71
497.	AAF13573	Human enterovirus 71
498.	AAF13572	Human enterovirus 71
499.	AAF13571	Human enterovirus 71
500.	AAF13570	Human enterovirus 71
501.	AAF13569	Human enterovirus 71
502.	AAF13568	Human enterovirus 71
503.	AAF13567	Human enterovirus 71
504.	AAF13566	Human enterovirus 71
505.	AAF13565	Human enterovirus 71
506.	AAF13564	Human enterovirus 71
507.	AAF13563	Human enterovirus 71
508.	AAF13562	Human enterovirus 71
509.	AAF13561	Human enterovirus 71
510.	AAF13560	Human enterovirus 71
511.	AAF13559	Human enterovirus 71
512.	AAF13558	Human enterovirus 71
513.	AAF13557	Human enterovirus 71
514.	AAF13556	Human enterovirus 71
515.	AAF13555	Human enterovirus 71
516.	AAF13554	Human enterovirus 71
517.	AAF13553	Human enterovirus 71

518.	AAF13552	Human enterovirus 71
519.	AAF13551	Human enterovirus 71
520.	AAF13550	Human enterovirus 71
521.	AAF13549	Human enterovirus 71
522.	AAF13548	Human enterovirus 71
523.	AAF13547	Human enterovirus 71
524.	AAF13546	Human enterovirus 71
525.	AAF13545	Human enterovirus 71
526.	AAF13544	Human enterovirus 71
527.	AAF13543	Human enterovirus 71
528.	AAF13542	Human enterovirus 71
529.	AAF13541	Human enterovirus 71
530.	AAF13540	Human enterovirus 71
531.	AAF13539	Human enterovirus 71
532.	AAF13538	Human enterovirus 71
533.	AAF13537	Human enterovirus 71
534.	AAF13536	Human enterovirus 71
535.	AAF13535	Human enterovirus 71
536.	AAF13534	Human enterovirus 71
537.	AAF13533	Human enterovirus 71
538.	AAF13532	Human enterovirus 71
539.	AAF13531	Human enterovirus 71
540.	AAF13530	Human enterovirus 71
541.	AAF13529	Human enterovirus 71
542.	AAF13528	Human enterovirus 71
543.	AAF13527	Human enterovirus 71
544.	AAF13526	Human enterovirus 71
545.	AAF13525	Human enterovirus 71
546.	AAF13524	Human enterovirus 71
547.	AAF13523	Human enterovirus 71
548.	AAF13522	Human enterovirus 71
549.	AAF13521	Human enterovirus 71
550.	AAF13520	Human enterovirus 71
551.	AAF13519	Human enterovirus 71
552.	AAF13518	Human enterovirus 71
553.	AAF13517	Human enterovirus 71
554.	AAF13516	Human enterovirus 71
555.	AAF13515	Human enterovirus 71
556.	AAF13514	Human enterovirus 71
557.	AAF13513	Human enterovirus 71
558.	AAF13512	Human enterovirus 71
559.	AAF13511	Human enterovirus 71
560.	AAF13510	Human enterovirus 71
561.	AAF13509	Human enterovirus 71

562.	AAF13508	Human enterovirus 71
563.	AAF13507	Human enterovirus 71
564.	AAF13506	Human enterovirus 71
565.	AAF13505	Human enterovirus 71
566.	AAF13504	Human enterovirus 71
567.	AAF13503	Human enterovirus 71
568.	AAF13502	Human enterovirus 71
569.	AAF13501	Human enterovirus 71
570.	AAF13500	Human enterovirus 71
571.	AAF13499	Human enterovirus 71
572.	AAF13498	Human enterovirus 71
573.	ACS88974	Human coxsackievirus A10
574.	ACS88973	Human coxsackievirus A10
575.	ACS88972	Human coxsackievirus A10
576.	ACS88971	Human coxsackievirus A10
577.	ACS88970	Human coxsackievirus A10
578.	ACS88969	Human coxsackievirus A10
579.	AAP74361	Human poliovirus 2
580.	AAP20426	Human enterovirus 71
581.	BAC77678	Human echovirus 13
582.	BAC77677	Human echovirus 13
583.	BAC77676	Human echovirus 13
584.	BAC77675	Human echovirus 13
585.	BAE79470	Human echovirus 30
586.	BAE79469	Human echovirus 30
587.	BAE79468	Human echovirus 30
588.	BAE79467	Human echovirus 30
589.	BAE79466	Human echovirus 30
590.	BAE79465	Human echovirus 30
591.	BAE79464	Human echovirus 30
592.	BAE79463	Human echovirus 30
593.	BAE79462	Human echovirus 30
594.	BAE79461	Human echovirus 30
595.	BAE79460	Human echovirus 30
596.	BAE79459	Human echovirus 30
597.	BAE79458	Human echovirus 30
598.	BAE79457	Human echovirus 30
599.	BAE79456	Human echovirus 30
600.	BAE79455	Human echovirus 30
601.	BAE79454	Human echovirus 30
602.	BAE79453	Human echovirus 30
603.	BAE79452	Human echovirus 30
604.	BAE79451	Human echovirus 30
605.	BAE79450	Human echovirus 30

606.	BAE79449	Human echovirus 30
607.	BAE79448	Human echovirus 30
608.	BAE79447	Human echovirus 30
609.	BAE79446	Human echovirus 30
610.	BAE79445	Human echovirus 30
611.	BAE79444	Human echovirus 30
612.	BAC76037	Human echovirus 13
613.	BAC76036	Human echovirus 13
614.	BAC76035	Human echovirus 13
615.	BAC76034	Human echovirus 13
616.	BAC76033	Human echovirus 13
617.	BAC76032	Human echovirus 13
618.	BAC76031	Human echovirus 13
619.	BAC76030	Human echovirus 13
620.	BAC76029	Human echovirus 13
621.	BAC76028	Human echovirus 13
622.	BAC76084	Human coxsackievirus A10
623.	BAF32974	Human echovirus 30
624.	BAF32973	Human echovirus 30
625.	BAF32972	Human echovirus 30
626.	BAF32971	Human echovirus 30
627.	BAF32970	Human echovirus 30
628.	BAF32969	Human echovirus 30
629.	BAF32968	Human echovirus 30
630.	BAD98605	Human enterovirus 71
631.	BAD98604	Human enterovirus 71
632.	BAD98603	Human enterovirus 71
633.	BAD98602	Human enterovirus 71
634.	BAD98601	Human enterovirus 71
635.	BAD98600	Human enterovirus 71
636.	BAD98599	Human enterovirus 71
637.	BAD98598	Human enterovirus 71
638.	BAD98597	Human enterovirus 71
639.	BAD98596	Human enterovirus 71
640.	BAD98595	Human enterovirus 71
641.	BAD98594	Human enterovirus 71
642.	BAD98593	Human enterovirus 71
643.	BAD98592	Human enterovirus 71
644.	BAD98591	Human enterovirus 71
645.	BAD98590	Human enterovirus 71
646.	BAD98589	Human enterovirus 71
647.	BAD98588	Human enterovirus 71
648.	BAD98587	Human enterovirus 71
649.	BAD98586	Human enterovirus 71

650.	BAD98585	Human enterovirus 71
651.	BAD98584	Human enterovirus 71
652.	BAD98583	Human enterovirus 71
653.	BAD98582	Human enterovirus 71
654.	BAD98581	Human enterovirus 71
655.	BAD98580	Human enterovirus 71
656.	BAD98579	Human enterovirus 71
657.	BAD98578	Human enterovirus 71
658.	BAD98577	Human enterovirus 71
659.	BAD98576	Human enterovirus 71
660.	BAD98575	Human enterovirus 71
661.	BAD98574	Human enterovirus 71
662.	BAD98573	Human enterovirus 71
663.	BAD98572	Human enterovirus 71
664.	BAD98571	Human enterovirus 71
665.	BAD98570	Human enterovirus 71
666.	BAD98569	Human enterovirus 71
667.	BAD16713.2	Human enterovirus 71
668.	BAD16711.2	Human enterovirus 71
669.	BAD16717	Human enterovirus 71
670.	BAD16716	Human enterovirus 71
671.	BAD16715	Human enterovirus 71
672.	BAD16714	Human enterovirus 71
673.	BAD16712	Human enterovirus 71
674.	BAD16710	Human enterovirus 71
675.	BAD12623.2	Human enterovirus 71
676.	BAD12622.2	Human enterovirus 71
677.	BAD12621.2	Human enterovirus 71
678.	BAD12620.2	Human enterovirus 71
679.	BAD12624	Human enterovirus 71
680.	BAD12619	Human echovirus 18
681.	BAD12618	Human echovirus 18
682.	BAD12617	Human echovirus 18
683.	BAD12616	Human echovirus 18
684.	BAD12615	Human echovirus 13
685.	BAD12614	Human echovirus 9
686.	BAD12613	Human echovirus 6
687.	BAD12612	Human echovirus 6
688.	BAD12611	Human echovirus 6
689.	BAD12610	Human coxsackievirus B5
690.	BAD12609	Human coxsackievirus B5
691.	BAD12608	Human coxsackievirus B1
692.	BAD12607	Human coxsackievirus A10
693.	BAD12606	Human coxsackievirus A10

694.	BAD12605	Human coxsackievirus A10
695.	BAD12604	Human coxsackievirus A10
696.	BAD12603	Human coxsackievirus A10
697.	BAD12602	Human coxsackievirus A10
698.	BAD12601	Human coxsackievirus A10
699.	BAD12600	Human coxsackievirus A9
700.	BAD12599	Human coxsackievirus A9
701.	BAD12598	Human coxsackievirus A9
702.	BAD12597	Human coxsackievirus A9
703.	BAD02309	Human coxsackievirus A10
704.	BAC92728	Human coxsackievirus A10
705.	BAC92727	Human coxsackievirus A10
706.	BAC92726	Human enterovirus 71
707.	BAC92725	Human enterovirus 71
708.	BAC92724	Human enterovirus 71
709.	BAC92723	Human enterovirus 71
710.	BAC85165	Human coxsackievirus A16
711.	BAC85164	Human coxsackievirus A16
712.	BAC85163	Human coxsackievirus A2
713.	BAC85162	Human coxsackievirus A2
714.	BAC85161	Human coxsackievirus A10
715.	BAC85160	Human coxsackievirus A10
716.	BAC85159	Human coxsackievirus A10
717.	BAC85158	Human coxsackievirus A10
718.	BAC21662	Human enterovirus 71
719.	BAC21661	Human enterovirus 71
720.	BAC21660	Human enterovirus 71
721.	BAC21659	Human enterovirus 71
722.	BAC21658	Human enterovirus 71
723.	BAC21657	Human enterovirus 71
724.	BAC21706	Human enterovirus 71
725.	BAC21705	Human enterovirus 71
726.	BAC21704	Human enterovirus 71
727.	BAC21703	Human enterovirus 71
728.	BAC21702	Human enterovirus 71
729.	BAC21701	Human enterovirus 71
730.	ACS12928	Human enterovirus 71
731.	ACS12927	Human enterovirus 71
732.	ACS12926	Human enterovirus 71
733.	ACS12925	Human enterovirus 71
734.	ACS12924	Human enterovirus 71
735.	AAB22445.2	Human coxsackievirus B4
736.	ADA68444	Human enterovirus 71
737.	ADA68443	Human enterovirus 71

738.	ACT98552	Human enterovirus 97
739.	ACT98551	Human enterovirus 75
740.	ACT98550	Human enterovirus 73
741.	ACT98549	Human echovirus 30
742.	ACT98548	Human echovirus 30
743.	ACT98547	Human echovirus 30
744.	ACT98546	Human echovirus 29
745.	ACT98545	Human echovirus 29
746.	ACT98544	Human echovirus 29
747.	ACT98543	Human echovirus 29
748.	ACT98542	Human echovirus 29
749.	ACT98541	Human echovirus 29
750.	ACT98540	Human echovirus 24
751.	ACT98539	Human echovirus 24
752.	ACT98538	Human echovirus 24
753.	ACT98537	Human echovirus 24
754.	ACT98536	Human echovirus 24
755.	ACT98535	Human echovirus 21
756.	ACT98534	Human echovirus 21
757.	ACT98533	Human echovirus 21
758.	ACT98532	Human echovirus 21
759.	ACT98531	Human echovirus 20
760.	ACT98530	Human echovirus 20
761.	ACT98529	Human echovirus 20
762.	ACT98528	Human echovirus 19
763.	ACT98527	Human echovirus 19
764.	ACT98526	Human echovirus 19
765.	ACT98525	Human echovirus 19
766.	ACT98524	Human echovirus 18
767.	ACT98523	Human echovirus 17
768.	ACT98522	Human echovirus 14
769.	ACT98521	Human echovirus 14
770.	ACT98520	Human echovirus 14
771.	ACT98519	Human echovirus 14
772.	ACT98518	Human echovirus 14
773.	ACT98517	Human echovirus 14
774.	ACT98516	Human echovirus 14
775.	ACT98515	Human echovirus 14
776.	ACT98514	Human echovirus 14
777.	ACT98513	Human echovirus 13
778.	ACT98512	Human echovirus 13
779.	ACT98511	Human echovirus 13
780.	ACT98510	Human echovirus 13
781.	ACT98509	Human echovirus 12

782.	ACT98508	Human echovirus 12
783.	ACT98507	Human echovirus 12
784.	ACT98506	Human echovirus 11
785.	ACT98505	Human echovirus 11
786.	ACT98504	Human echovirus 11
787.	ACT98503	Human echovirus 11
788.	ACT98502	Human echovirus 11
789.	ACT98501	Human echovirus 11
790.	ACT98500	Human echovirus 9
791.	ACT98499	Human echovirus 7
792.	ACT98498	Human echovirus 7
793.	ACT98497	Human echovirus 7
794.	ACT98496	Human echovirus 6
795.	ACT98495	Human echovirus 6
796.	ACT98494	Human echovirus 6
797.	ACT98493	Human echovirus 6
798.	ACT98492	Human echovirus 6
799.	ACT98491	Human echovirus 6
800.	ACT98490	Human echovirus 6
801.	ACT98489	Human echovirus 6
802.	ACT98488	Human echovirus 3
803.	ACT98487	Human echovirus 3
804.	ACT98486	Human echovirus 1
805.	ACT98485	Human echovirus 1
806.	ACT98484	Human echovirus 1
807.	ACT98483	Human echovirus 1
808.	ACT98482	Human coxsackievirus B5
809.	ACT98481	Human coxsackievirus B5
810.	ACT98480	Human coxsackievirus B4
811.	ACT98479	Human coxsackievirus B4
812.	ACT98478	Human coxsackievirus B3
813.	ACT98477	Human coxsackievirus B3
814.	ACT98476	Human coxsackievirus B3
815.	ACT98475	Human coxsackievirus B3
816.	ACT98474	Human coxsackievirus B3
817.	ACT98473	Human coxsackievirus B3
818.	ACT98472	Human coxsackievirus B3
819.	ACT98471	Human coxsackievirus B3
820.	ACT98470	Human coxsackievirus B3
821.	ACT98469	Human coxsackievirus B3
822.	ACT98468	Human coxsackievirus B3
823.	ACT98467	Human coxsackievirus B3
824.	ACT98466	Human coxsackievirus B3
825.	ACT98465	Human coxsackievirus B3

826.	ACT98464	Human coxsackievirus B3
827.	ACT98463	Human coxsackievirus B3
828.	ACT98462	Human coxsackievirus B3
829.	ACT98461	Human coxsackievirus B3
830.	ACT98460	Human coxsackievirus B3
831.	ACT98459	Human coxsackievirus B3
832.	ACT98458	Human coxsackievirus B3
833.	ACT98457	Human coxsackievirus B3
834.	ACT98456	Human coxsackievirus B3
835.	ACT98455	Human coxsackievirus B3
836.	ACT98454	Human coxsackievirus B2
837.	ACT98453	Human coxsackievirus B2
838.	ACT98452	Human coxsackievirus B2
839.	ACT98451	Human coxsackievirus B2
840.	ACT98450	Human coxsackievirus B2
841.	ACT98449	Human coxsackievirus B2
842.	ACT98448	Human coxsackievirus B1
843.	ACT98447	Human coxsackievirus B1
844.	ACT98446	Human coxsackievirus B1
845.	ACT98445	Human coxsackievirus B1
846.	ACT98444	Human coxsackievirus B1
847.	ACT98443	Human coxsackievirus B1
848.	ACT98442	Human coxsackievirus A9
849.	ACT98441	Human coxsackievirus A9
850.	ACT98440	Human coxsackievirus A9
851.	ACT98439	Human coxsackievirus A9
852.	ACT98438	Human coxsackievirus A9
853.	ACT98437	Human coxsackievirus A24
854.	ACT98436	Human coxsackievirus A24
855.	ACT98435	Human coxsackievirus A21
856.	ACT98434	Human coxsackievirus A20
857.	ABS82575	Human enterovirus 71
858.	AAD27821	Swine vesicular disease virus
859.	ABG78787	Human poliovirus 2
860.	ABG78786	Human poliovirus 2
861.	ABG78785	Human poliovirus 2
862.	ABG78784	Human poliovirus 2
863.	ABG78783	Human poliovirus 2
864.	ABG78782	Human poliovirus 2
865.	ABG78781	Human poliovirus 1
866.	ABG78780	Human poliovirus 2
867.	ABG78779	Human poliovirus 2
868.	ABG78778	Human poliovirus 2
869.	ABG78777	Human poliovirus 2

870.	ABG78776	Human poliovirus 2
871.	ABG78775	Human poliovirus 2
872.	ABG78774	Human poliovirus 1
873.	ABG78773	Human poliovirus 1
874.	ABG78772	Human poliovirus 3
875.	ABG78771	Human poliovirus 3
876.	AEO00845	Human enterovirus 71
877.	AEO00844	Human enterovirus 71
878.	AEO00843	Human enterovirus 71
879.	AEO00842	Human enterovirus 71
880.	AEO00841	Human enterovirus 71
881.	AEO00840	Human enterovirus 71
882.	AEO00839	Human enterovirus 71
883.	AEO00838	Human enterovirus 71
884.	AEO00837	Human enterovirus 71
885.	AEO00836	Human enterovirus 71
886.	AEO00835	Human enterovirus 71
887.	AEO00834	Human enterovirus 71
888.	AEO00833	Human enterovirus 71
889.	AEO00832	Human enterovirus 71
890.	AEO00831	Human enterovirus 71
891.	AEO00830	Human enterovirus 71
892.	AEO00829	Human enterovirus 71
893.	AEO00828	Human enterovirus 71
894.	AEO00827	Human enterovirus 71
895.	AEO00826	Human enterovirus 71
896.	AEO00825	Human enterovirus 71
897.	AEO00824	Human enterovirus 71
898.	AEO00823	Human enterovirus 71
899.	AEO00822	Human enterovirus 71
900.	ADQ64048	Human echovirus 11
901.	ADQ64047	Human echovirus 11
902.	ADQ64046	Human echovirus 11
903.	ADQ64045	Human echovirus 11
904.	ADQ64044	Human echovirus 11
905.	ADQ64043	Human echovirus 11
906.	ADQ64042	Human echovirus 11
907.	ADH01807	Human enterovirus 71
908.	ADH01806	Human enterovirus 71
909.	ADH01805	Human enterovirus 71
910.	ADH01804	Human enterovirus 71
911.	ADH01803	Human enterovirus 71
912.	ADH01802	Human enterovirus 71
913.	ADC53098	Human enterovirus 71

914.	ADC53097	Human enterovirus 71
915.	ADC53096	Human enterovirus 71
916.	ADC53095	Human enterovirus 71
917.	ADC53094	Human enterovirus 71
918.	ADC53093	Human enterovirus 71
919.	ADC53092	Human enterovirus 71
920.	ADC53091	Human enterovirus 71
921.	ADC53090	Human enterovirus 71
922.	ADC53089	Human enterovirus 71
923.	ADC53088	Human enterovirus 71
924.	ADC53087	Human enterovirus 71
925.	ADC53086	Human enterovirus 71
926.	ADC53085	Human enterovirus 71
927.	ADC53084	Human enterovirus 71
928.	ADC53083	Human enterovirus 71
929.	ADC53082	Human enterovirus 71
930.	ADC53081	Human enterovirus 71
931.	ADC53080	Human enterovirus 71
932.	ADC53079	Human enterovirus 71
933.	ADC53078	Human enterovirus 71
934.	ADC53077	Human enterovirus 71
935.	ADC53076	Human enterovirus 71
936.	ADC53075	Human enterovirus 71
937.	ADC53074	Human enterovirus 71
938.	ADF43062	Human coxsackievirus A10
939.	ADF43061	Human coxsackievirus A10
940.	ADF43060	Human coxsackievirus A10
941.	ADF43059	Human coxsackievirus A10
942.	ADF43058	Human coxsackievirus A10
943.	ADF43057	Human coxsackievirus A10
944.	ADF43056	Human coxsackievirus A10
945.	ADF43055	Human coxsackievirus A10
946.	ADF43054	Human coxsackievirus A10
947.	ADF43053	Human coxsackievirus A10
948.	ADF43052	Human coxsackievirus A10
949.	ADF43051	Human coxsackievirus A10
950.	ADF43050	Human coxsackievirus A10
951.	ADF43049	Human coxsackievirus A10
952.	ADF35570	Human coxsackievirus B1
953.	ADF35569	Human coxsackievirus B1
954.	ADF35568	Human coxsackievirus B1
955.	ADF35567	Human coxsackievirus B1
956.	ACS26243	Human echovirus 6
957.	ACS26242	Human echovirus 6

958.	ACS26241	Human echovirus 6
959.	ACS26240	Human echovirus 6
960.	ACS26239	Human echovirus 6
961.	ACS26238	Human echovirus 6
962.	ACS26237	Human echovirus 6
963.	ABP38353	Human poliovirus 2
964.	ABP38352	Human poliovirus 3
965.	ABP38351	Human poliovirus 3
966.	ABP38350	Human poliovirus 3
967.	ABP38349	Human poliovirus 2
968.	ABP38348	Human poliovirus 2
969.	ABP38347	Human poliovirus 2
970.	ABP38346	Human poliovirus 2
971.	ABP38345	Human poliovirus 2
972.	ABP38344	Human poliovirus 2
973.	ABP38343	Human poliovirus 2
974.	ABP38342	Human poliovirus 2
975.	ABP38341	Human poliovirus 1
976.	AAP33061	Human poliovirus 3
977.	ACJ64145	Human poliovirus 1
978.	ACJ64144	Human poliovirus 1
979.	ACJ64143	Human poliovirus 1
980.	ACJ64142	Human poliovirus 1
981.	ACJ64141	Human poliovirus 1
982.	ACJ64140	Human poliovirus 1
983.	ACJ64139	Human poliovirus 1
984.	ACJ64138	Human poliovirus 1
985.	ACJ64137	Human poliovirus 2
986.	ACJ64136	Human poliovirus 2
987.	ACJ64135	Human poliovirus 1
988.	ACJ64134	Human poliovirus 2
989.	AAM83184	Human echovirus 11
990.	AAM83183	Human echovirus 11
991.	AAM83182	Human echovirus 11
992.	AAM83181	Human echovirus 11
993.	AAM83180	Human echovirus 11
994.	AAM83179	Human echovirus 11
995.	AAM83178	Human echovirus 11
996.	AAM83177	Human echovirus 11
997.	AAM83176	Human echovirus 11
998.	AAM83175	Human echovirus 11
999.	AAM83174	Human echovirus 11
1000.	AAM83173	Human echovirus 11
1001.	AAM83172	Human echovirus 11

1002.	AAM83171	Human echovirus 11
1003.	AAM83170	Human echovirus 11
1004.	AAM83169	Human echovirus 11
1005.	AAM83168	Human echovirus 11
1006.	AAM83167	Human echovirus 11
1007.	AAM83166	Human echovirus 11
1008.	AAM83165	Human echovirus 11
1009.	AAM83164	Human echovirus 11
1010.	AAM83163	Human echovirus 11
1011.	AAM83162	Human echovirus 11
1012.	AAM83161	Human echovirus 11
1013.	AAM83160	Human echovirus 11
1014.	AAM83159	Human echovirus 11
1015.	AAM83158	Human echovirus 11
1016.	AAM83157	Human echovirus 11
1017.	AAM83156	Human echovirus 11
1018.	AAM83155	Human echovirus 11
1019.	AAM83154	Human echovirus 11
1020.	AAM83153	Human echovirus 11
1021.	AAM83152	Human echovirus 11
1022.	AAM83151	Human echovirus 11
1023.	AAM83150	Human echovirus 11
1024.	AAM83149	Human echovirus 11
1025.	AAM83148	Human echovirus 11
1026.	AAM83147	Human echovirus 11
1027.	AAM83146	Human echovirus 11
1028.	AAM83145	Human echovirus 11
1029.	AAM83144	Human echovirus 11
1030.	AAM83143	Human echovirus 11
1031.	AAM83142	Human echovirus 11
1032.	AAM83141	Human echovirus 11
1033.	AAM83140	Human echovirus 11
1034.	AAM83139	Human echovirus 11
1035.	AAM83138	Human echovirus 11
1036.	AAM83137	Human echovirus 11
1037.	AAM83136	Human echovirus 11
1038.	AAM83135	Human echovirus 11
1039.	AAM83134	Human echovirus 11
1040.	AAL56710	Human enterovirus 71
1041.	AAL56709	Human enterovirus 71
1042.	AAL56708	Human enterovirus 71
1043.	AAL56707	Human enterovirus 71
1044.	AAL56706	Human enterovirus 71
1045.	AAL56705	Human enterovirus 71

1046.	AAL56704	Human enterovirus 71
1047.	AAL56703	Human enterovirus 71
1048.	AAL56702	Human enterovirus 71
1049.	AAL56701	Human enterovirus 71
1050.	AAL56700	Human enterovirus 71
1051.	AAL56699	Human enterovirus 71
1052.	AAL56698	Human enterovirus 71
1053.	AAL56697	Human enterovirus 71
1054.	AAL56696	Human enterovirus 71
1055.	AAL56695	Human enterovirus 71
1056.	AAL56694	Human enterovirus 71
1057.	AAL56693	Human enterovirus 71
1058.	AAL56692	Human enterovirus 71
1059.	AAL56691	Human enterovirus 71
1060.	AAL56690	Human enterovirus 71
1061.	AAL56689	Human enterovirus 71
1062.	AAL56688	Human enterovirus 71
1063.	AAL56687	Human enterovirus 71
1064.	AAL56686	Human enterovirus 71
1065.	AAL56685	Human enterovirus 71
1066.	AAL56684	Human enterovirus 71
1067.	AAL56683	Human enterovirus 71
1068.	AAL56682	Human enterovirus 71
1069.	AAL56681	Human enterovirus 71
1070.	AAL56680	Human enterovirus 71
1071.	AAL56679	Human enterovirus 71
1072.	AAL56678	Human enterovirus 71
1073.	AAL56677	Human enterovirus 71
1074.	AAL56676	Human enterovirus 71
1075.	AAL56675	Human enterovirus 71
1076.	AAL56674	Human enterovirus 71
1077.	AAL56673	Human enterovirus 71
1078.	AAL56672	Human enterovirus 71
1079.	AAL56671	Human enterovirus 71
1080.	AAL56670	Human enterovirus 71
1081.	AAL56669	Human enterovirus 71
1082.	AAL56668	Human enterovirus 71
1083.	ACO38652	Human enterovirus 71
1084.	ACO38651	Human enterovirus 71
1085.	ACO38650	Human enterovirus 71
1086.	ABB43277	Human echovirus 11
1087.	ABB43276	Human echovirus 11
1088.	ABB43275	Human echovirus 11
1089.	ABB43274	Human echovirus 11

1090.	AAM97534	Human enterovirus 71
1091.	AAM97533	Human enterovirus 71
1092.	AAM97532	Human enterovirus 71
1093.	AAM97531	Human enterovirus 71
1094.	AAM97530	Human enterovirus 71
1095.	AAM97529	Human enterovirus 71
1096.	AAM97528	Human enterovirus 71
1097.	AAM97527	Human enterovirus 71
1098.	AAM97526	Human enterovirus 71
1099.	AAM97525	Human enterovirus 71
1100.	AAM97524	Human enterovirus 71
1101.	AAL30020	Human poliovirus 1
1102.	AAL30019	Human poliovirus 1
1103.	AAL30018	Human poliovirus 1
1104.	AAL30017	Human poliovirus 1
1105.	AAL30016	Human poliovirus 1
1106.	AAL30015	Human poliovirus 1
1107.	AAK62678	Human enterovirus 71
1108.	BAG24260	Human enterovirus 71
1109.	BAG24259	Human enterovirus 71
1110.	BAG24258	Human enterovirus 71
1111.	BAG24257	Human enterovirus 71
1112.	BAG24256	Human enterovirus 71
1113.	BAG24255	Human enterovirus 71
1114.	BAG24254	Human enterovirus 71
1115.	BAG24253	Human enterovirus 71
1116.	BAG24252	Human enterovirus 71
1117.	BAG24251	Human enterovirus 71
1118.	BAG24250	Human enterovirus 71
1119.	BAG24249	Human enterovirus 71
1120.	BAG24248	Human enterovirus 71
1121.	BAG24247	Human enterovirus 71
1122.	BAG24246	Human enterovirus 71
1123.	BAG24245	Human enterovirus 71
1124.	BAG24244	Human enterovirus 71
1125.	BAG24243	Human enterovirus 71
1126.	BAG24242	Human enterovirus 71
1127.	BAG24241	Human enterovirus 71
1128.	BAG24240	Human enterovirus 71
1129.	BAG24239	Human enterovirus 71
1130.	BAG24238	Human enterovirus 71
1131.	BAG24237	Human enterovirus 71
1132.	BAG24236	Human enterovirus 71
1133.	BAG24235	Human enterovirus 71

1134.	BAG24234	Human enterovirus 71
1135.	BAG24233	Human enterovirus 71
1136.	BAG24232	Human enterovirus 71
1137.	BAG24231	Human enterovirus 71
1138.	BAG24230	Human enterovirus 71
1139.	BAH59290	Human echovirus 24
1140.	ABF68757	Human poliovirus 1
1141.	BAB69814	Human enterovirus 71
1142.	BAB69813	Human enterovirus 71
1143.	BAB69812	Human enterovirus 71
1144.	BAB69811	Human enterovirus 71
1145.	BAB69810	Human enterovirus 71
1146.	BAB69809	Human enterovirus 71
1147.	BAB69808	Human enterovirus 71
1148.	ADT82699	Human enterovirus 71
1149.	ADT82698	Human enterovirus 71
1150.	ADT82697	Human enterovirus 71
1151.	ADT82696	Human enterovirus 71
1152.	ADT82695	Human enterovirus 71
1153.	AEQ61907	Human enterovirus 71
1154.	AAY33005	Human poliovirus 3
1155.	AAY33004	Human poliovirus 3
1156.	AAY33003	Human poliovirus 3
1157.	AAY33002	Human poliovirus 2
1158.	AAY33001	Human poliovirus 2
1159.	AAY33000	Human poliovirus 2
1160.	AAY32999	Human poliovirus 2
1161.	AAY32998	Human poliovirus 2
1162.	AAY32997	Human poliovirus 1
1163.	AAY32996	Human poliovirus 1
1164.	AAY32995	Human poliovirus 1
1165.	AAY32994	Human poliovirus 1
1166.	AAY32993	Human poliovirus 1
1167.	AAY32992	Human poliovirus 1
1168.	AAY32991	Human poliovirus 1
1169.	ABU88088	Human echovirus 30
1170.	ABU88087	Human echovirus 30
1171.	ABU88086	Human echovirus 30
1172.	ABU88085	Human echovirus 30
1173.	ABI24107	Human echovirus 30
1174.	ABI24106	Human echovirus 30
1175.	ABI24105	Human echovirus 30
1176.	ABI24104	Human echovirus 30
1177.	ABI24103	Human echovirus 30

1178.	ABI24102	Human echovirus 30
1179.	ABI24101	Human echovirus 30
1180.	ABI24100	Human echovirus 30
1181.	ABI24099	Human echovirus 30
1182.	ABI24098	Human echovirus 30
1183.	ABI24097	Human echovirus 30
1184.	ABI24095	Human echovirus 30
1185.	ABI24094	Human echovirus 30
1186.	ABI24093	Human echovirus 30
1187.	ABI24092	Human echovirus 30
1188.	ABI24091	Human echovirus 30
1189.	ABI24090	Human echovirus 30
1190.	ABI24089	Human echovirus 30
1191.	ABI24088	Human echovirus 30
1192.	ABI24087	Human echovirus 30
1193.	ABI24086	Human echovirus 30
1194.	ABI24085	Human echovirus 30
1195.	ABI24084	Human echovirus 30
1196.	ACU46762	Human poliovirus 3
1197.	ADZ57524	Human coxsackievirus A24
1198.	ADZ57523	Human coxsackievirus A24
1199.	ADZ57522	Human coxsackievirus A24
1200.	ADZ57521	Human coxsackievirus A24
1201.	ADZ57520	Human coxsackievirus A24
1202.	ADZ57519	Human coxsackievirus A24
1203.	ADZ57518	Human coxsackievirus A24
1204.	ADZ57517	Human coxsackievirus A24
1205.	ADZ57516	Human coxsackievirus A24
1206.	ADZ57515	Human coxsackievirus A24
1207.	ADZ57514	Human coxsackievirus A24
1208.	ADZ57513	Human coxsackievirus A24
1209.	ADZ57512	Human coxsackievirus A24
1210.	ADZ57511	Human coxsackievirus A24
1211.	ADZ57510	Human coxsackievirus A24
1212.	ADZ57509	Human coxsackievirus A24
1213.	ADZ57508	Human coxsackievirus A24
1214.	ADZ57507	Human coxsackievirus A24
1215.	ADZ57506	Human coxsackievirus A24
1216.	ADZ57505	Human coxsackievirus A24
1217.	ADZ57504	Human coxsackievirus A24
1218.	ADZ57503	Human coxsackievirus A24
1219.	ADZ57502	Human coxsackievirus A24
1220.	ADZ57501	Human coxsackievirus A24
1221.	ADZ57500	Human coxsackievirus A24

1222.	ADZ57499	Human coxsackievirus A24
1223.	ADZ57498	Human coxsackievirus A24
1224.	ADZ57497	Human coxsackievirus A24
1225.	ADZ57496	Human coxsackievirus A24
1226.	ADZ57495	Human coxsackievirus A24
1227.	ADZ57494	Human coxsackievirus A24
1228.	ADZ57493	Human coxsackievirus A24
1229.	ADZ57492	Human coxsackievirus A24
1230.	ADZ57491	Human coxsackievirus A24
1231.	ADZ57490	Human coxsackievirus A24
1232.	ADZ57489	Human coxsackievirus A24
1233.	ADZ57488	Human coxsackievirus A24
1234.	AEE81795	Human echovirus 6
1235.	AEE81794	Human echovirus 6
1236.	AEE81793	Human echovirus 6
1237.	AEE81792	Human echovirus 6
1238.	AEE81791	Human echovirus 6
1239.	AEE81790	Human echovirus 6
1240.	AEE81789	Human echovirus 6
1241.	AEE81788	Human echovirus 6
1242.	AEE81787	Human echovirus 6
1243.	AEE81786	Human echovirus 6
1244.	AEE81785	Human echovirus 6
1245.	AEE81784	Human echovirus 6
1246.	AEE81783	Human echovirus 6
1247.	AEE81782	Human echovirus 6
1248.	AEE81781	Human echovirus 6
1249.	AEE81780	Human echovirus 6
1250.	ACU46753	Human poliovirus 1
1251.	ADO39544	Human echovirus 11
1252.	ADO39543	Human echovirus 11
1253.	ADO39542	Human echovirus 11
1254.	ADO39541	Human echovirus 11
1255.	ADO39540	Human echovirus 11
1256.	ADO39539	Human echovirus 11
1257.	ADO39538	Human echovirus 11
1258.	ADO39537	Human echovirus 11
1259.	ADO39536	Human echovirus 11
1260.	ADO39535	Human echovirus 11
1261.	ACF04414	Human coxsackievirus A24
1262.	ACF04413	Human coxsackievirus A24
1263.	ACF04412	Human coxsackievirus A24
1264.	ACF04411	Human coxsackievirus A24
1265.	ACF04410	Human coxsackievirus A24

1266.	ACF04409	Human coxsackievirus A24
1267.	ACF04408	Human coxsackievirus A24
1268.	ACF04407	Human coxsackievirus A24
1269.	ACF04406	Human coxsackievirus A24
1270.	ACK37272	Human poliovirus 3
1271.	ACK37271	Human poliovirus 3
1272.	ACK37270	Human poliovirus 3
1273.	ACK37269	Human poliovirus 3
1274.	ACK37268	Human poliovirus 3
1275.	ACK37267	Human poliovirus 3
1276.	ACK37266	Human poliovirus 3
1277.	ACK37265	Human poliovirus 3
1278.	ACK37264	Human poliovirus 3
1279.	ACK37263	Human poliovirus 3
1280.	ACK37262	Human poliovirus 3
1281.	ACK37261	Human poliovirus 3
1282.	ACK37260	Human poliovirus 3
1283.	ACK37259	Human poliovirus 3
1284.	ACK37258	Human poliovirus 3
1285.	ACK37257	Human poliovirus 3
1286.	ACK37256	Human poliovirus 3
1287.	ACK37255	Human poliovirus 3
1288.	ABV26334	Human poliovirus 1
1289.	ABV26291	Human poliovirus 3
1290.	ABV26290	Human poliovirus 3
1291.	ABV26289	Human poliovirus 3
1292.	ABQ52389	Human enterovirus 96
1293.	ABQ52388	Human enterovirus 96
1294.	ABQ52387	Human enterovirus 96
1295.	ABQ52386	Human enterovirus 96
1296.	ABQ52385	Human enterovirus 96
1297.	ABQ52384	Human enterovirus 96
1298.	ABQ52383	Human enterovirus 96
1299.	ABQ52382	Human enterovirus 97
1300.	ABQ52381	Human enterovirus 76
1301.	ACU46751	Human poliovirus 1
1302.	ACU46747	Human poliovirus 3
1303.	ACU46746	Human poliovirus 3
1304.	ACU46745	Human poliovirus 3
1305.	ACU46761	Human poliovirus 3
1306.	ACU46760	Human poliovirus 2
1307.	ACU46759	Human poliovirus 3
1308.	ACU46758	Human poliovirus 2
1309.	ACU46757	Human poliovirus 2

1310.	ACU46756	Human poliovirus 2
1311.	ACU46755	Human poliovirus 2
1312.	ACU46754	Human poliovirus 1
1313.	ACU46752	Human poliovirus 1
1314.	ACU46750	Human poliovirus 2
1315.	ACU46749	Human poliovirus 2
1316.	ACU46748	Human poliovirus 2
1317.	ABS32203	Human poliovirus 2
1318.	AAB25284	coxsackievirus A2 CAV-2, Peptide, 297 aa
1319.	ACK36954	Human poliovirus 1
1320.	ACK36953	Human poliovirus 1
1321.	ACK36952	Human poliovirus 1
1322.	ACK36951	Human poliovirus 1
1323.	ABH10610	Human enterovirus 71
1324.	ABI24096	Human echovirus 30
1325.	ABN79676	Human enterovirus C
1326.	AAX19085	Human enterovirus 71
1327.	AAX19084	Human enterovirus 71
1328.	AAX19083	Human enterovirus 71
1329.	AAX19082	Human enterovirus 71
1330.	AAX19081	Human enterovirus 71
1331.	AAX19080	Human enterovirus 71
1332.	AEQ61280	Human enterovirus 71
1333.	AEQ61279	Human enterovirus 71
1334.	AEQ61278	Human enterovirus 71
1335.	AEQ61277	Human enterovirus 71
1336.	AET76436	Human enterovirus 71
1337.	AET76435	Human enterovirus 71
1338.	AET76434	Human enterovirus 71
1339.	AET76433	Human enterovirus 71
1340.	AET76432	Human enterovirus 71
1341.	AET76431	Human enterovirus 71
1342.	AET76430	Human enterovirus 71
1343.	AET76429	Human enterovirus 71
1344.	AET76428	Human enterovirus 71
1345.	AET76427	Human enterovirus 71
1346.	AET76426	Human enterovirus 71
1347.	AET76425	Human enterovirus 71
1348.	AET76424	Human enterovirus 71
1349.	AET76423	Human enterovirus 71
1350.	AET76422	Human enterovirus 71
1351.	AET76421	Human enterovirus 71
1352.	AET76420	Human enterovirus 71
1353.	AET76419	Human enterovirus 71

1354.	AET76418	Human enterovirus 71
1355.	AET76417	Human enterovirus 71
1356.	AET76416	Human enterovirus 71
1357.	AET76415	Human enterovirus 71
1358.	AET76414	Human enterovirus 71
1359.	AET76413	Human enterovirus 71
1360.	AET76412	Human enterovirus 71
1361.	AET76411	Human enterovirus 71
1362.	AET76410	Human enterovirus 71
1363.	AET76409	Human enterovirus 71
1364.	AET76408	Human enterovirus 71
1365.	AET76407	Human enterovirus 71
1366.	AET76406	Human enterovirus 71
1367.	AET76405	Human enterovirus 71
1368.	AET76404	Human enterovirus 71
1369.	AET76403	Human enterovirus 71
1370.	AET76402	Human enterovirus 71
1371.	AET76401	Human enterovirus 71
1372.	AET76400	Human enterovirus 71
1373.	AET76399	Human enterovirus 71
1374.	AET76398	Human enterovirus 71
1375.	AET76397	Human enterovirus 71
1376.	AET76396	Human enterovirus 71
1377.	AET76395	Human enterovirus 71
1378.	AET76394	Human enterovirus 71
1379.	AET76393	Human enterovirus 71
1380.	AET76392	Human enterovirus 71
1381.	AET76391	Human enterovirus 71
1382.	AET76390	Human enterovirus 71
1383.	AET76389	Human enterovirus 71
1384.	AET76388	Human enterovirus 71
1385.	ACK37361	Human enterovirus 71
1386.	ACK37360	Human enterovirus 71
1387.	ACK37359	Human enterovirus 71
1388.	ACK37358	Human enterovirus 71
1389.	ACK37357	Human enterovirus 71
1390.	ACK37356	Human enterovirus 71
1391.	ACK37355	Human enterovirus 71
1392.	ACK37354	Human enterovirus 71
1393.	AEM23783	Human coxsackievirus A16
1394.	AEM23782	Human coxsackievirus A16
1395.	AEM23781	Human coxsackievirus A16
1396.	AEM23780	Human enterovirus 71
1397.	AEM23779	Human enterovirus 71

1398.	AEM23778	Human enterovirus 71
1399.	AEM23777	Human enterovirus 71
1400.	AEM23776	Human enterovirus 71
1401.	AEM23775	Human enterovirus 71
1402.	AEM23774	Human enterovirus 71
1403.	AEM23773	Human enterovirus 71
1404.	AEK06431	Human coxsackievirus B5
1405.	AEK06430	Human coxsackievirus B5
1406.	AEK06429	Human coxsackievirus B5
1407.	AEK06428	Human coxsackievirus B5
1408.	AEK06427	Human coxsackievirus B5
1409.	AEK06426	Human coxsackievirus B5
1410.	AEK06425	Human coxsackievirus B5
1411.	AEK06424	Human coxsackievirus B5
1412.	AEK06423	Human coxsackievirus B5
1413.	AEK06422	Human coxsackievirus B5
1414.	AEK06421	Human coxsackievirus B5
1415.	AEK06420	Human coxsackievirus B5
1416.	AEK06419	Human coxsackievirus B5
1417.	AEK06418	Human coxsackievirus B5
1418.	AEK06417	Human coxsackievirus B5
1419.	AEK06416	Human coxsackievirus B5
1420.	AEK06415	Human coxsackievirus B5
1421.	AEI98587	Human coxsackievirus B5
1422.	AEI98586	Human coxsackievirus B5
1423.	AEI98582	Human coxsackievirus B5
1424.	AEI98581	Human coxsackievirus B5
1425.	AEI98580	Human coxsackievirus B5
1426.	AEI98562	Human coxsackievirus B1
1427.	AEI98561	Human coxsackievirus B1
1428.	AEI98560	Human coxsackievirus B1
1429.	AEI98559	Human coxsackievirus B1
1430.	AEI71368	Human enterovirus 71
1431.	AEI71367	Human enterovirus 71
1432.	AEI71366	Human enterovirus 71
1433.	AEI71365	Human enterovirus 71
1434.	AEI71364	Human enterovirus 71
1435.	AEI71363	Human enterovirus 71
1436.	AEI71362	Human enterovirus 71
1437.	AEI71361	Human enterovirus 71
1438.	AEI71360	Human enterovirus 71
1439.	AEI71359	Human enterovirus 71
1440.	AEI71358	Human enterovirus 71
1441.	AEI71357	Human enterovirus 71

1442.	AEI71356	Human enterovirus 71
1443.	AEI71355	Human enterovirus 71
1444.	AEI71354	Human enterovirus 71
1445.	AEI71353	Human enterovirus 71
1446.	AEI71352	Human enterovirus 71
1447.	AEI71351	Human enterovirus 71
1448.	AEI71350	Human enterovirus 71
1449.	AEI71349	Human enterovirus 71
1450.	AEI71348	Human enterovirus 71
1451.	AEI71347	Human enterovirus 71
1452.	AEI71346	Human enterovirus 71
1453.	AEI71345	Human enterovirus 71
1454.	AEI71344	Human enterovirus 71
1455.	AEI71343	Human enterovirus 71
1456.	AEI71342	Human enterovirus 71
1457.	AEI71341	Human enterovirus 71
1458.	AEI71340	Human enterovirus 71
1459.	AEI71339	Human enterovirus 71
1460.	AEI71338	Human enterovirus 71
1461.	AEI71337	Human enterovirus 71
1462.	AEI71336	Human enterovirus 71
1463.	AEI71335	Human enterovirus 71
1464.	AEI71334	Human enterovirus 71
1465.	AEI71333	Human enterovirus 71
1466.	AEI71332	Human enterovirus 71
1467.	AEI71331	Human enterovirus 71
1468.	AEI71330	Human enterovirus 71
1469.	AEI71329	Human enterovirus 71
1470.	AEI71328	Human enterovirus 71
1471.	AEI71327	Human enterovirus 71
1472.	AEI71326	Human enterovirus 71
1473.	AEI71325	Human enterovirus 71
1474.	AEI71324	Human enterovirus 71
1475.	AEI71323	Human enterovirus 71
1476.	AEI71322	Human enterovirus 71
1477.	AEI71321	Human enterovirus 71
1478.	AEI71320	Human enterovirus 71
1479.	AEI71319	Human enterovirus 71
1480.	AEI71318	Human enterovirus 71
1481.	AEI71317	Human enterovirus 71
1482.	AEI71316	Human enterovirus 71
1483.	AEI71315	Human enterovirus 71
1484.	AEI71314	Human enterovirus 71
1485.	AEI71313	Human enterovirus 71

1486.	AEI71312	Human enterovirus 71
1487.	AEI71311	Human enterovirus 71
1488.	AEI71310	Human enterovirus 71
1489.	AEI71309	Human enterovirus 71
1490.	AEI71308	Human enterovirus 71
1491.	AEI71307	Human enterovirus 71
1492.	AEI71306	Human enterovirus 71
1493.	AEI71305	Human enterovirus 71
1494.	AEI71304	Human enterovirus 71
1495.	AEI71303	Human enterovirus 71
1496.	AEI71302	Human enterovirus 71
1497.	AEI71301	Human enterovirus 71
1498.	AEI71300	Human enterovirus 71
1499.	AEI71299	Human enterovirus 71
1500.	AEI71298	Human enterovirus 71
1501.	AEI71297	Human enterovirus 71
1502.	AEI71296	Human enterovirus 71
1503.	AEI71295	Human enterovirus 71
1504.	AEI71294	Human enterovirus 71
1505.	AEI71293	Human enterovirus 71
1506.	AEI71292	Human enterovirus 71
1507.	AEI71291	Human enterovirus 71
1508.	AEI71290	Human enterovirus 71
1509.	AEI71289	Human enterovirus 71
1510.	AEI71288	Human enterovirus 71
1511.	AEI71287	Human enterovirus 71
1512.	AEI71286	Human enterovirus 71
1513.	AEI71285	Human enterovirus 71
1514.	AEI71284	Human enterovirus 71
1515.	AEI71283	Human enterovirus 71
1516.	AEI71282	Human enterovirus 71
1517.	AEI71281	Human enterovirus 71
1518.	AEI71280	Human enterovirus 71
1519.	AEI71279	Human enterovirus 71
1520.	AEI71278	Human enterovirus 71
1521.	AEI71277	Human enterovirus 71
1522.	AEI71276	Human enterovirus 71
1523.	AEI71275	Human enterovirus 71
1524.	AEI71274	Human enterovirus 71
1525.	AEI71273	Human enterovirus 71
1526.	AEI71272	Human enterovirus 71
1527.	AEI71271	Human enterovirus 71
1528.	AEI71270	Human enterovirus 71
1529.	AEI71269	Human enterovirus 71

1530.	AEI71268	Human enterovirus 71
1531.	AEI71267	Human enterovirus 71
1532.	AEI71266	Human enterovirus 71
1533.	AEI71265	Human enterovirus 71
1534.	AEI71264	Human enterovirus 71
1535.	AEI71263	Human enterovirus 71
1536.	AEI71262	Human enterovirus 71
1537.	AEI71261	Human enterovirus 71
1538.	AEI71260	Human enterovirus 71
1539.	AEI71259	Human enterovirus 71
1540.	AEI71258	Human enterovirus 71
1541.	AEI71257	Human enterovirus 71
1542.	AEI71256	Human enterovirus 71
1543.	AEI71255	Human enterovirus 71
1544.	AEI71254	Human enterovirus 71
1545.	AEI71253	Human enterovirus 71
1546.	AEI71252	Human enterovirus 71
1547.	AEI71251	Human enterovirus 71
1548.	AEI71250	Human enterovirus 71
1549.	AEI71249	Human enterovirus 71
1550.	AEI71248	Human enterovirus 71
1551.	AEI71247	Human enterovirus 71
1552.	AEI71246	Human enterovirus 71
1553.	AEI71245	Human enterovirus 71
1554.	AEI71244	Human enterovirus 71
1555.	AEI71243	Human enterovirus 71
1556.	AEI71242	Human enterovirus 71
1557.	AEI71241	Human enterovirus 71
1558.	AEI71240	Human enterovirus 71
1559.	ACK36950	Human poliovirus 1
1560.	ACK36949	Human poliovirus 1
1561.	ACK36948	Human poliovirus 1
1562.	AEO12126	Human coxsackievirus A16
1563.	AEO12125	Human coxsackievirus A16
1564.	AEO12124	Human coxsackievirus A16
1565.	AEO12123	Human coxsackievirus A16
1566.	AEO12122	Human coxsackievirus A16
1567.	AEO12121	Human coxsackievirus A16
1568.	AEO12120	Human coxsackievirus A16
1569.	AEO12119	Human coxsackievirus A16
1570.	AEO12118	Human coxsackievirus A16
1571.	AEO12117	Human coxsackievirus A16
1572.	AEM23817	Human enterovirus 71
1573.	AEM23816	Human enterovirus 71

1574.	AEM23815	Human enterovirus 71
1575.	AEM23814	Human enterovirus 71
1576.	AEM23813	Human enterovirus 71
1577.	AEM23812	Human enterovirus 71
1578.	AEM23811	Human enterovirus 71
1579.	AEM23810	Human enterovirus 71
1580.	AEM23809	Human enterovirus 71
1581.	AEM23808	Human enterovirus 71
1582.	AEM23807	Human enterovirus 71
1583.	AEM23806	Human enterovirus 71
1584.	AEM23805	Human enterovirus 71
1585.	AEM23804	Human enterovirus 71
1586.	AEM23803	Human enterovirus 71
1587.	AEM23802	Human enterovirus 71
1588.	AEM23801	Human enterovirus 71
1589.	AEM23800	Human enterovirus 71
1590.	AEM23799	Human enterovirus 71
1591.	AEM23798	Human enterovirus 71
1592.	AEE25796	Human echovirus 6
1593.	AEE25795	Human echovirus 6
1594.	AEE25794	Human echovirus 6
1595.	AEE25793	Human echovirus 6
1596.	AEE25792	Human echovirus 6
1597.	AEE25791	Human echovirus 6
1598.	AEE25790	Human echovirus 6
1599.	AEE25789	Human echovirus 6
1600.	AEE25788	Human echovirus 6
1601.	AEE25787	Human echovirus 6
1602.	AEE25786	Human echovirus 6
1603.	AEE25785	Human echovirus 6
1604.	AAK69378	Human enterovirus 71
1605.	AAK69377	Human enterovirus 71
1606.	AAK69376	Human enterovirus 71
1607.	AAK69375	Human enterovirus 71
1608.	AAK69374	Human enterovirus 71
1609.	AAK69373	Human enterovirus 71
1610.	AAK69372	Human enterovirus 71
1611.	AAK69371	Human enterovirus 71
1612.	AAK69370	Human enterovirus 71
1613.	AAK69369	Human enterovirus 71
1614.	AAK69368	Human enterovirus 71
1615.	AAK69367	Human enterovirus 71
1616.	AAK69366	Human enterovirus 71
1617.	AAK69365	Human enterovirus 71

1618.	AAK69364	Human enterovirus 71
1619.	AAK69363	Human enterovirus 71
1620.	AAK69362	Human enterovirus 71
1621.	AAK69361	Human enterovirus 71
1622.	AAK69360	Human enterovirus 71
1623.	ADN93443	Human coxsackievirus B5
1624.	ABS56999	Human enterovirus 71
1625.	AEI71239	Human enterovirus 71
1626.	AEI71238	Human enterovirus 71
1627.	AEI71237	Human enterovirus 71
1628.	AAL69629	Simian picornavirus 7
1629.	ACO82307	Human echovirus 30
1630.	ACO82306	Human echovirus 30
1631.	ACO82305	Human echovirus 30
1632.	ACO82304	Human echovirus 30
1633.	ACO82303	Human echovirus 30
1634.	ACO82302	Human echovirus 30
1635.	ACO82301	Human echovirus 30
1636.	ACO82300	Human echovirus 30
1637.	ACO82299	Human echovirus 30
1638.	ACO82298	Human echovirus 30
1639.	ACO82297	Human echovirus 30
1640.	ACO82296	Human echovirus 30
1641.	ACO82295	Human echovirus 30
1642.	ACO82294	Human echovirus 30
1643.	ACO82293	Human echovirus 30
1644.	ACO82292	Human echovirus 30
1645.	ACO82291	Human echovirus 30
1646.	ACO82290	Human echovirus 30
1647.	YP_003795178	Human enterovirus 109
1648.	ADG63654	Human echovirus 25
1649.	ADP89611	Human enterovirus 71
1650.	ADP89610	Human enterovirus 71
1651.	ADP89609	Human enterovirus 71
1652.	ADP89608	Human enterovirus 71
1653.	ADP89607	Human enterovirus 71
1654.	ADP89606	Human enterovirus 71
1655.	ADP89605	Human enterovirus 71
1656.	ADP89604	Human enterovirus 71
1657.	ADP89603	Human enterovirus 71
1658.	ADP89602	Human enterovirus 71
1659.	ADP89601	Human enterovirus 71
1660.	ADP89600	Human enterovirus 71
1661.	ADP89599	Human enterovirus 71

1662.	ADP89598	Human enterovirus 71
1663.	ADP89597	Human enterovirus 71
1664.	ADP89596	Human enterovirus 71
1665.	ADP89595	Human enterovirus 71
1666.	ADD52076	Human enterovirus 71
1667.	ADD52075	Human enterovirus 71
1668.	ADD52074	Human enterovirus 71
1669.	ADD52073	Human enterovirus 71
1670.	ADD52072	Human enterovirus 71
1671.	ADD52071	Human enterovirus 71
1672.	YP_003104788	Human enterovirus 107
1673.	ACZ65994	Human enterovirus 71
1674.	ACZ65993	Human enterovirus 71
1675.	ACZ65992	Human enterovirus 71
1676.	ACZ65991	Human enterovirus 71
1677.	ACZ65990	Human enterovirus 71
1678.	ACZ65989	Human enterovirus 71
1679.	ACZ65988	Human enterovirus 71
1680.	ACZ65987	Human enterovirus 71
1681.	ACZ65986	Human enterovirus 71
1682.	ACZ65985	Human enterovirus 71
1683.	ACZ65984	Human enterovirus 71
1684.	ACZ65983	Human enterovirus 71
1685.	ACZ65982	Human enterovirus 71
1686.	ACZ65981	Human enterovirus 71
1687.	ACZ65980	Human enterovirus 71
1688.	ACZ65979	Human enterovirus 71
1689.	ACZ65978	Human enterovirus 71
1690.	ACZ65977	Human enterovirus 71
1691.	ACZ65976	Human enterovirus 71
1692.	YP_003104778	Human enterovirus 98
1693.	ABQ96314	Human enterovirus B
1694.	ABQ96313	Human enterovirus B
1695.	ABQ96312	Human enterovirus B
1696.	ABQ96311	Human enterovirus B
1697.	ABQ96310	Human enterovirus B
1698.	ABQ96309	Human enterovirus B
1699.	ABQ96308	Human enterovirus B
1700.	ABQ96307	Human enterovirus B
1701.	ABQ96306	Human enterovirus B
1702.	ABQ96305	Human enterovirus B
1703.	ABQ96304	Human enterovirus B
1704.	ABQ96303	Human enterovirus B
1705.	ABQ96302	Human enterovirus B

1706.	ABQ96301	Human enterovirus B
1707.	ABQ96300	Human enterovirus B
1708.	ABQ96299	Human enterovirus B
1709.	ABQ96298	Human enterovirus B
1710.	ABQ96297	Human enterovirus B
1711.	ABQ96296	Human enterovirus B
1712.	AAZ80287	Human enterovirus 76
1713.	AAZ80284	Human echovirus 30
1714.	AAZ80278	Human coxsackievirus A11
1715.	AAZ80277	Human echovirus 3
1716.	AAZ80270	Human coxsackievirus A24
1717.	AAZ80265	Human coxsackievirus A17
1718.	ADD52076	Human enterovirus 71
1719.	ADD52075	Human enterovirus 71
1720.	ADD52074	Human enterovirus 71
1721.	ADD52073	Human enterovirus 71
1722.	ADD52072	Human enterovirus 71
1723.	ADD52071	Human enterovirus 71
1724.	YP_003104788	Human enterovirus 107
1725.	ACZ65994	Human enterovirus 71
1726.	ACZ65993	Human enterovirus 71
1727.	ACZ65992	Human enterovirus 71
1728.	ACZ65991	Human enterovirus 71
1729.	ACZ65990	Human enterovirus 71
1730.	ACZ65989	Human enterovirus 71
1731.	ACZ65988	Human enterovirus 71
1732.	ACZ65987	Human enterovirus 71
1733.	ACZ65986	Human enterovirus 71
1734.	ACZ65985	Human enterovirus 71
1735.	ACZ65984	Human enterovirus 71
1736.	ACZ65983	Human enterovirus 71
1737.	ACZ65982	Human enterovirus 71
1738.	ACZ65981	Human enterovirus 71
1739.	ACZ65980	Human enterovirus 71
1740.	ACZ65979	Human enterovirus 71
1741.	ACZ65978	Human enterovirus 71
1742.	ACZ65977	Human enterovirus 71
1743.	ACZ65976	Human enterovirus 71
1744.	YP_003104778	Human enterovirus 98
1745.	ABQ96314	Human enterovirus B
1746.	ABQ96313	Human enterovirus B
1747.	ABQ96312	Human enterovirus B
1748.	ABQ96311	Human enterovirus B
1749.	ABQ96310	Human enterovirus B

1750.	ABQ96309	Human enterovirus B
1751.	ABQ96308	Human enterovirus B
1752.	ABQ96307	Human enterovirus B
1753.	ABQ96306	Human enterovirus B
1754.	ABQ96305	Human enterovirus B
1755.	ABQ96304	Human enterovirus B
1756.	ABQ96303	Human enterovirus B
1757.	ABQ96302	Human enterovirus B
1758.	ABQ96301	Human enterovirus B
1759.	ABQ96300	Human enterovirus B
1760.	ABQ96299	Human enterovirus B
1761.	ABQ96298	Human enterovirus B
1762.	ABQ96297	Human enterovirus B
1763.	ABQ96296	Human enterovirus B
1764.	AAZ80287	Human enterovirus 76
1765.	AAZ80284	Human echovirus 30
1766.	AAZ80278	Human coxsackievirus A11
1767.	AAZ80277	Human echovirus 3
1768.	AAZ80270	Human coxsackievirus A24
1769.	AAZ80265	Human coxsackievirus A17
1770.	AAZ80258	Human enterovirus 97
1771.	AAZ80256	Enterovirus sp.
1772.	AAZ80255	Human echovirus 9
1773.	AAZ80252	Human coxsackievirus A21
1774.	AAZ80250	Human coxsackievirus A13
1775.	AAZ80249	Human coxsackievirus A13
1776.	AAZ80239	Human coxsackievirus A21
1777.	AAZ80234	Human echovirus 25
1778.	AAZ80233	Human echovirus 29
1779.	AAZ80228	Human coxsackievirus A21
1780.	AAZ80223	Human coxsackievirus A20
1781.	AAZ80222	Human coxsackievirus B3
1782.	AAZ80213	Human coxsackievirus A20
1783.	AAZ80202	Human echovirus 26
1784.	AAZ80196	Human coxsackievirus A7
1785.	AAZ80190	Human coxsackievirus A13
1786.	AAZ80181	Human enterovirus 100
1787.	AAZ80175	Human coxsackievirus A13
1788.	AAZ80169	Human enterovirus 96
1789.	AAZ80166	Human enterovirus 90
1790.	AAZ80162	Human coxsackievirus B4
1791.	AAZ80159	Human echovirus 9
1792.	AAZ80155	Human echovirus 9
1793.	AAZ80150	Human coxsackievirus A21

1794.	AAZ80145	Human coxsackievirus A20
1795.	AAZ80144	Human coxsackievirus A24
1796.	AAZ80140	Human coxsackievirus A14
1797.	AAZ80131	Human enterovirus 90
1798.	AAZ80130	Human coxsackievirus A20
1799.	AAZ80127	Human echovirus 17
1800.	AAZ80126	Human coxsackievirus A11
1801.	AAZ80121	Human echovirus 14
1802.	AAZ80120	Human coxsackievirus A4
1803.	AAZ80112	Human coxsackievirus A13
1804.	AAZ80109	Human coxsackievirus A22
1805.	AAZ80106	Human coxsackievirus A20
1806.	AAZ80100	Human coxsackievirus A13
1807.	ACZ28924	Human poliovirus 3
1808.	ACZ28923	Human poliovirus 3
1809.	ACZ28922	Human poliovirus 3
1810.	ACZ28921	Human poliovirus 3
1811.	ACZ28920	Human poliovirus 3
1812.	ACZ28919	Human poliovirus 3
1813.	ACZ28918	Human poliovirus 3
1814.	ACZ28917	Human poliovirus 3
1815.	ACZ28916	Human poliovirus 3
1816.	ACZ28915	Human poliovirus 3
1817.	ACZ28914	Human poliovirus 3
1818.	ACZ28913	Human poliovirus 3
1819.	ACZ28912	Human poliovirus 3
1820.	ACZ28911	Human poliovirus 3
1821.	ACZ28910	Human poliovirus 3
1822.	ACZ28909	Human poliovirus 3
1823.	ACZ28908	Human poliovirus 3
1824.	ACZ28907	Human poliovirus 3
1825.	ACZ28906	Human poliovirus 3
1826.	ACZ28905	Human poliovirus 3
1827.	ACZ28904	Human poliovirus 3
1828.	ACZ28903	Human poliovirus 3
1829.	ACW84382	Human enterovirus 71
1830.	ACW84381	Human enterovirus 71
1831.	ACW84380	Human enterovirus 71
1832.	ACW84379	Human enterovirus 71
1833.	ACW84378	Human enterovirus 71
1834.	ACW84377	Human enterovirus 71
1835.	ACW84376	Human enterovirus 71
1836.	ACW84375	Human enterovirus 71
1837.	ACW84374	Human enterovirus 71

1838.	ACW84373	Human enterovirus 71
1839.	ACV84420	Human enterovirus 71
1840.	ACV84419	Human enterovirus 71
1841.	ACV84418	Human enterovirus 71
1842.	ACV84417	Human enterovirus 71
1843.	ACV84416	Human enterovirus 71
1844.	ACV84415	Human enterovirus 71
1845.	ACV84414	Human enterovirus 71
1846.	ACV84413	Human enterovirus 71
1847.	ACV84412	Human enterovirus 71
1848.	ACV84411	Human enterovirus 71
1849.	ACV84410	Human enterovirus 71
1850.	ACV84409	Human enterovirus 71
1851.	ACV84408	Human enterovirus 71
1852.	ACV84407	Human enterovirus 71
1853.	ACV84406	Human enterovirus 71
1854.	ACV84405	Human enterovirus 71
1855.	ACV84404	Human enterovirus 71
1856.	ACV84403	Human enterovirus 71
1857.	ACV84402	Human enterovirus 71
1858.	ACV84401	Human enterovirus 71
1859.	ACV84400	Human enterovirus 71
1860.	ACV84399	Human enterovirus 71
1861.	ACV84398	Human enterovirus 71
1862.	ACV84397	Human enterovirus 71
1863.	ACT67681	Human echovirus 24
1864.	ACT67680	Human echovirus 24
1865.	ACT67679	Human coxsackievirus B3
1866.	ACT67678	Human coxsackievirus A9
1867.	ACT67677	Human coxsackievirus B5
1868.	ACT67676	Human coxsackievirus B5
1869.	ACT67675	Human coxsackievirus B5
1870.	ACT67674	Human coxsackievirus B5
1871.	ACT67673	Human coxsackievirus B5
1872.	ACT67672	Human coxsackievirus B5
1873.	ACT67671	Human coxsackievirus B5
1874.	ACT67670	Human coxsackievirus B5
1875.	ACT67669	Human coxsackievirus B5
1876.	ACT67668	Human coxsackievirus B5
1877.	ACT67667	Human coxsackievirus B5
1878.	ACT67666	Human echovirus 25
1879.	ACT67665	Human echovirus 25
1880.	ACT67664	Human echovirus 25
1881.	ACT67663	Human echovirus 25

1882.	ACT67662	Human echovirus 25
1883.	ACT67661	Human echovirus 25
1884.	ACT67660	Human echovirus 25
1885.	ACT52665	Human enterovirus 90
1886.	ACT52664	Human enterovirus 90
1887.	ACT52663	Human enterovirus 90
1888.	ACT52662	Human enterovirus 76
1889.	ACT52661	Human enterovirus 76
1890.	ACT52660	Human enterovirus 71
1891.	ACT52659	Human enterovirus 71
1892.	ACT52658	Human enterovirus 71
1893.	ACT52657	Human enterovirus 71
1894.	ACT52656	Human enterovirus 71
1895.	ACT52655	Human enterovirus 71
1896.	ACT52654	Human enterovirus 71
1897.	ACT52653	Human enterovirus 71
1898.	ACT52652	Human enterovirus 71
1899.	ACT52651	Human enterovirus 71
1900.	ACT52650	Human enterovirus 71
1901.	ACT52649	Human enterovirus 71
1902.	ACT52648	Human enterovirus 71
1903.	ACT52647	Human enterovirus 71
1904.	ACT52646	Human enterovirus 71
1905.	ACT52645	Human enterovirus 71
1906.	ACT52644	Human enterovirus 71
1907.	ACT52643	Human enterovirus 71
1908.	ACT52642	Human enterovirus 71
1909.	ACT52641	Human enterovirus 71
1910.	ACT52640	Human enterovirus 71
1911.	ACT52639	Human enterovirus 71
1912.	ACT52638	Human enterovirus 71
1913.	ACT52637	Human enterovirus 71
1914.	ACT52636	Human enterovirus 71
1915.	ACT52635	Human enterovirus 71
1916.	ACT52634	Human enterovirus 71
1917.	ACT52633	Human enterovirus 71
1918.	ACT52632	Human enterovirus 71
1919.	ACT52631	Human enterovirus 71
1920.	ACT52630	Human enterovirus 71
1921.	ACT52629	Human enterovirus 71
1922.	ACT52628	Human enterovirus 71
1923.	ACT52627	Human coxsackievirus A16
1924.	ACT52626	Human coxsackievirus A16
1925.	ACT52625	Human coxsackievirus A16

1926.	ACT52624	Human coxsackievirus A16
1927.	ACT52623	Human coxsackievirus A16
1928.	ACT52622	Human coxsackievirus A16
1929.	ACT52621	Human coxsackievirus A16
1930.	ACT52620	Human coxsackievirus A16
1931.	ACT52619	Human coxsackievirus A16
1932.	ACT52618	Human coxsackievirus A16
1933.	ACT52617	Human coxsackievirus A16
1934.	ACT52616	Human coxsackievirus A14
1935.	ACT52615	Human coxsackievirus A5
1936.	ACT52614	Human coxsackievirus A4
1937.	ACT52613	Human coxsackievirus A4
1938.	ACT52612	Human coxsackievirus A4
1939.	ACT52611	Human coxsackievirus A4
1940.	ACT52610	Human coxsackievirus A4
1941.	ACT52609	Human coxsackievirus A4
1942.	ACO89613	Human enterovirus 71
1943.	ACO89612	Human enterovirus 71
1944.	ACO89611	Human enterovirus 71
1945.	ACO89610	Human enterovirus 71
1946.	ACO89609	Human enterovirus 71
1947.	ACO89608	Human enterovirus 71
1948.	ACO89607	Human enterovirus 71
1949.	ACO89606	Human enterovirus 71
1950.	ACO89605	Human enterovirus 71
1951.	ACO89604	Human enterovirus 71
1952.	ACO89603	Human enterovirus 71
1953.	ACO89602	Human enterovirus 71
1954.	ACO89601	Human enterovirus 71
1955.	ACO89600	Human enterovirus 71
1956.	ACO89599	Human enterovirus 71
1957.	ACO89598	Human enterovirus 71
1958.	ACO89597	Human enterovirus 71
1959.	ACO89596	Human enterovirus 71
1960.	ACO89595	Human enterovirus 71
1961.	ACO89594	Human enterovirus 71
1962.	ACO89593	Human enterovirus 71
1963.	ACO89592	Human enterovirus 71
1964.	ACO89591	Human enterovirus 71
1965.	ACO89590	Human enterovirus 71
1966.	ACO89589	Human enterovirus 71
1967.	ACO89588	Human enterovirus 71
1968.	ACO89587	Human enterovirus 71
1969.	ACO89586	Human enterovirus 71

1970.	ACO89585	Human enterovirus 71
1971.	ACO89584	Human enterovirus 71
1972.	ACC86051	Human echovirus 9
1973.	ACC86050	Human echovirus 18
1974.	ACC86049	Human coxsackievirus B5
1975.	ACC86048	Human coxsackievirus B5
1976.	ACC86047	Human coxsackievirus B5
1977.	ACC86046	Human coxsackievirus B5
1978.	ACC86045	Human coxsackievirus B5
1979.	ACC86043	Human coxsackievirus B5
1980.	ACC86042	Human coxsackievirus B5
1981.	ACC86041	Human coxsackievirus B5
1982.	ACC86040	Human coxsackievirus B5
1983.	ACC86039	Human coxsackievirus B5
1984.	ACC86038	Human coxsackievirus B5
1985.	ACC86037	Human coxsackievirus B5
1986.	ACA24477	Human enterovirus B
1987.	ACA24476	Human enterovirus B
1988.	ACA24475	Human enterovirus B
1989.	ACA24474	Human enterovirus B
1990.	ACA24473	Human enterovirus B
1991.	ACA24472	Human enterovirus B
1992.	ACA24471	Human enterovirus B
1993.	ACA24470	Human enterovirus B
1994.	ACA24469	Human enterovirus B
1995.	ACA24468	Human enterovirus B
1996.	ACA24467	Human enterovirus B
1997.	ACA24466	Human enterovirus B
1998.	ACA24465	Human enterovirus B
1999.	ACA24464	Human enterovirus B
2000.	ACA24463	Human enterovirus B
2001.	ACA24462	Human enterovirus B
2002.	ACA24461	Human enterovirus B
2003.	ACA24460	Human enterovirus B
2004.	ACA24459	Human enterovirus B
2005.	ACA24458	Human enterovirus B
2006.	ACA24457	Human enterovirus B
2007.	ACA24456	Human enterovirus B
2008.	AAP49483	Human echovirus 13
2009.	AAP49482	Human echovirus 13
2010.	AAP49481	Human echovirus 13
2011.	AAP49480	Human echovirus 13
2012.	AAP49479	Human echovirus 13
2013.	AAP49478	Human echovirus 13

2014.	AAP49477	Human echovirus 13
2015.	AAP49476	Human echovirus 13
2016.	AAP49475	Human echovirus 13
2017.	AAP49474	Human echovirus 13
2018.	AAP49473	Human echovirus 13
2019.	AAP49472	Human echovirus 13
2020.	AAP49471	Human echovirus 13
2021.	AAP49470	Human echovirus 13
2022.	AAP49469	Human echovirus 13
2023.	AAP49468	Human echovirus 13
2024.	AAP49467	Human echovirus 13
2025.	AAP49466	Human echovirus 13
2026.	AAP49464	Human echovirus 13
2027.	AAN64264	Human echovirus 30
2028.	AAN64263	Human echovirus 30
2029.	AAN64262	Human echovirus 30
2030.	AAN64261	Human echovirus 30
2031.	AAN64260	Human echovirus 30
2032.	AAN64259	Human echovirus 30
2033.	AAN64258	Human echovirus 30
2034.	AAN64257	Human echovirus 30
2035.	AAN64256	Human echovirus 30
2036.	AAN64255	Human echovirus 30
2037.	AAN64254	Human echovirus 30
2038.	AAN64253	Human echovirus 30
2039.	AAN64252	Human echovirus 30
2040.	AAN64251	Human echovirus 30
2041.	AAN64250	Human echovirus 30
2042.	AAN64249	Human echovirus 30
2043.	AAL17904	Human enterovirus 71
2044.	AAL17903	Human enterovirus 71
2045.	AAL17902	Human enterovirus 71
2046.	AAL17901	Human enterovirus 71
2047.	AAL17900	Human enterovirus 71
2048.	AAL17899	Human enterovirus 71
2049.	AAL17898	Human enterovirus 71
2050.	AAL17897	Human enterovirus 71
2051.	AAL17896	Human enterovirus 71
2052.	AAL17895	Human enterovirus 71
2053.	AAL17894	Human enterovirus 71
2054.	AAL17893	Human enterovirus 71
2055.	AAL17892	Human enterovirus 71
2056.	AAL17891	Human enterovirus 71
2057.	AAL17890	Human enterovirus 71

2058.	AAL17889	Human enterovirus 71
2059.	AAL17888	Human enterovirus 71
2060.	AAL17887	Human enterovirus 71
2061.	AAL17886	Human enterovirus 71
2062.	AAL17885	Human enterovirus 71
2063.	AAL17884	Human enterovirus 71
2064.	AAL17883	Human enterovirus 71
2065.	AAL17882	Human enterovirus 71
2066.	AAL17881	Human enterovirus 71
2067.	AAL17880	Human enterovirus 71
2068.	AAL17879	Human enterovirus 71
2069.	AAL17878	Human enterovirus 71
2070.	AAL17877	Human enterovirus 71
2071.	AAK37902	Human echovirus 30
2072.	AAK37901	Human echovirus 30
2073.	AAK37900	Human echovirus 30
2074.	AAK37899	Human echovirus 30
2075.	AAK37898	Human echovirus 30
2076.	AAK37897	Human echovirus 30
2077.	AAK37896	Human echovirus 30
2078.	AAK37895	Human echovirus 30
2079.	AAK37894	Human echovirus 30
2080.	AAK37893	Human echovirus 30
2081.	AAK37892	Human echovirus 30
2082.	AAK37891	Human echovirus 30
2083.	AAK37890	Human echovirus 30
2084.	AAK37889	Human echovirus 30
2085.	AAK37888	Human echovirus 30
2086.	AAK37887	Human echovirus 30
2087.	AAK37886	Human echovirus 30
2088.	AAK37885	Human echovirus 30
2089.	AAK37884	Human echovirus 30
2090.	AAK37883	Human echovirus 30
2091.	AAK37882	Human echovirus 30
2092.	AAK37881	Human echovirus 30
2093.	AAK37880	Human echovirus 30
2094.	AAK37879	Human echovirus 30
2095.	AAK37878	Human echovirus 30
2096.	AAK37877	Human echovirus 30
2097.	AAK37876	Human echovirus 30
2098.	AAK37875	Human echovirus 30
2099.	AAK37874	Human echovirus 30
2100.	AAK37873	Human echovirus 30
2101.	AAK37872	Human echovirus 30

2102.	AAK37871	Human echovirus 30
2103.	AAK37870	Human echovirus 30
2104.	AAK37869	Human echovirus 30
2105.	AAK37868	Human echovirus 30
2106.	AAK37867	Human echovirus 30
2107.	AAK37866	Human echovirus 30
2108.	AAK37865	Human echovirus 30
2109.	AAK37864	Human echovirus 30
2110.	AAK37863	Human echovirus 30
2111.	AAK37862	Human echovirus 30
2112.	AAK37861	Human echovirus 30
2113.	AAK37860	Human echovirus 30
2114.	AAK37859	Human echovirus 30
2115.	AAK37858	Human echovirus 30
2116.	AAK37857	Human echovirus 30
2117.	AAK37856	Human echovirus 30
2118.	AAK37855	Human echovirus 30
2119.	AAK37854	Human echovirus 30
2120.	AAK37853	Human echovirus 30
2121.	AAK37852	Human echovirus 30
2122.	AAK37851	Human echovirus 30
2123.	AAK37850	Human echovirus 30
2124.	AAK37849	Human echovirus 30
2125.	AAK37848	Human echovirus 30
2126.	AAK37847	Human echovirus 30
2127.	AAK37846	Human echovirus 30
2128.	AAK37845	Human echovirus 30
2129.	AAK37844	Human echovirus 30
2130.	AAK37843	Human echovirus 30
2131.	AAK37842	Human echovirus 30
2132.	AAK37841	Human echovirus 30
2133.	AAK37840	Human echovirus 30
2134.	AAF17848	Human echovirus 30
2135.	AAF17847	Human echovirus 30
2136.	AAF17846	Human echovirus 30
2137.	AAF17845	Human echovirus 30
2138.	AAF17844	Human echovirus 30
2139.	AAF17843	Human echovirus 30
2140.	AAF17842	Human echovirus 30
2141.	AAF17841	Human echovirus 30
2142.	AAF17840	Human echovirus 30
2143.	AAF17839	Human echovirus 30
2144.	AAF17838	Human echovirus 30
2145.	AAF17837	Human echovirus 30

2146.	AAF17836	Human echovirus 30
2147.	AAF17835	Human echovirus 30
2148.	AAF17834	Human echovirus 30
2149.	AAF17833	Human echovirus 30
2150.	AAF17832	Human echovirus 30
2151.	AAF17831	Human echovirus 30
2152.	AAF17830	Human echovirus 30
2153.	AAF17829	Human echovirus 30
2154.	AAF17828	Human echovirus 30
2155.	AAF17827	Human echovirus 30
2156.	AAF17826	Human echovirus 30
2157.	AAF17825	Human echovirus 30
2158.	AAF17824	Human echovirus 30
2159.	AAF17823	Human echovirus 30
2160.	AAF17822	Human echovirus 30
2161.	AAF17821	Human echovirus 30
2162.	AAF17820	Human echovirus 30
2163.	AAF17819	Human echovirus 30
2164.	AAF17818	Human echovirus 30
2165.	AAF17817	Human echovirus 30
2166.	AAF17816	Human echovirus 30
2167.	AAF17815	Human echovirus 30
2168.	AAF17814	Human echovirus 30
2169.	AAF17813	Human echovirus 30
2170.	AAF17812	Human echovirus 30
2171.	AAF17811	Human echovirus 30
2172.	AAF17810	Human echovirus 30
2173.	AAF17809	Human echovirus 30
2174.	AAF17808	Human echovirus 30
2175.	AAF17807	Human echovirus 30
2176.	AAF17806	Human echovirus 30
2177.	AAF17805	Human echovirus 30
2178.	AAF17804	Human echovirus 30
2179.	AAF17803	Human echovirus 30
2180.	AAF17802	Human echovirus 30
2181.	AAF17801	Human echovirus 30
2182.	AAF17800	Human echovirus 30
2183.	AAF17799	Human echovirus 30
2184.	AAF17798	Human echovirus 30
2185.	AAF17797	Human echovirus 30
2186.	AAF17796	Human echovirus 30
2187.	AAF17795	Human echovirus 30
2188.	AAF17794	Human echovirus 30
2189.	AAF17793	Human echovirus 30

2190.	AAF17792	Human echovirus 30
2191.	AAF17791	Human echovirus 30
2192.	AAF17790	Human echovirus 30
2193.	AAF17789	Human echovirus 30
2194.	AAF17788	Human echovirus 30
2195.	AAF17787	Human echovirus 30
2196.	AAF17786	Human echovirus 30
2197.	AAF17785	Human echovirus 30
2198.	AAF17784	Human echovirus 30
2199.	AAF17783	Human echovirus 30
2200.	AAF17782	Human echovirus 30
2201.	AAF17781	Human echovirus 30
2202.	AAF17780	Human echovirus 30
2203.	AAF17779	Human echovirus 30
2204.	AAF17778	Human echovirus 30
2205.	AAF17777	Human echovirus 30
2206.	AAF17776	Human echovirus 30
2207.	AAF17775	Human echovirus 30
2208.	AAF17774	Human echovirus 30
2209.	AAF17773	Human echovirus 30
2210.	AAF17772	Human echovirus 30
2211.	AAF17771	Human echovirus 30
2212.	AAF17770	Human echovirus 30
2213.	AAF17769	Human echovirus 30
2214.	AAF17768	Human echovirus 30
2215.	AAF17767	Human echovirus 30
2216.	AAF17766	Human echovirus 30
2217.	AAF17765	Human echovirus 30
2218.	AAF17764	Human echovirus 30
2219.	AAF17763	Human echovirus 30
2220.	AAF17762	Human echovirus 30
2221.	AAF17761	Human echovirus 30
2222.	AAF17760	Human echovirus 30
2223.	AAF17759	Human echovirus 30
2224.	AAF17758	Human echovirus 30
2225.	AAF17757	Human echovirus 30
2226.	AAF17756	Human echovirus 30
2227.	AAF17755	Human echovirus 30
2228.	AAF17754	Human echovirus 30
2229.	AAF17753	Human echovirus 30
2230.	AAF17752	Human echovirus 30
2231.	AAF17751	Human echovirus 30
2232.	AAF17750	Human echovirus 30
2233.	AAF17749	Human echovirus 30

2234.	AAF17748	Human echovirus 30
2235.	AAF17747	Human echovirus 30
2236.	AAF17746	Human echovirus 30
2237.	AAF17745	Human echovirus 30
2238.	AAF17744	Human echovirus 30
2239.	AAF17743	Human echovirus 30
2240.	AAF17742	Human echovirus 30
2241.	AAF17741	Human echovirus 30
2242.	ADA84748	Human coxsackievirus A4
2243.	AAM77873	Human enterovirus 73
2244.	NP_740448	Bovine enterovirus
2245.	ACH90151	Human enterovirus 71
2246.	ACH90150	Human enterovirus 71
2247.	ACH90149	Human enterovirus 71
2248.	ACH90148	Human enterovirus 71
2249.	ACH90147	Human enterovirus 71
2250.	ACH90146	Human enterovirus 71
2251.	ACH90145	Human enterovirus 71
2252.	ACH90144	Human enterovirus 71
2253.	ACH90143	Human enterovirus 71
2254.	ACH90142	Human enterovirus 71
2255.	ABR08709	Human enterovirus 74
2256.	CAA74812.2	Swine vesicular disease virus
2257.	CAA74856	Swine vesicular disease virus
2258.	CAA74829	Swine vesicular disease virus
2259.	CAA74824	Swine vesicular disease virus
2260.	CAA74823	Swine vesicular disease virus
2261.	CAA74822	Swine vesicular disease virus
2262.	CAA74821	Swine vesicular disease virus
2263.	CAA74820	Swine vesicular disease virus
2264.	CAA74816	Swine vesicular disease virus
2265.	CAA74814	Swine vesicular disease virus
2266.	CAA74813	Swine vesicular disease virus
2267.	ADP00594	Human enterovirus 71
2268.	ADP00593	Human enterovirus 71
2269.	ADP00592	Human enterovirus 71
2270.	ADP00591	Human enterovirus 71
2271.	ADP00590	Human enterovirus 71
2272.	ADP00589	Human enterovirus 71
2273.	ADP00588	Human enterovirus 71
2274.	ADP00587	Human enterovirus 71
2275.	ADP00586	Human enterovirus 71
2276.	ADP00585	Human enterovirus 71
2277.	ADP00583	Human enterovirus 71

2278.	BAE44373	Human coxsackievirus A6
2279.	BAE44372	Human coxsackievirus A6
2280.	BAE44371	Human coxsackievirus A6
2281.	BAE44383	Human echovirus 18
2282.	BAE44382	Human echovirus 16
2283.	BAE44381	Human echovirus 6
2284.	BAE44380	Human echovirus 6
2285.	BAE44379	Human echovirus 6
2286.	BAE44378	Human echovirus 6
2287.	BAE44375	Human coxsackievirus B3
2288.	BAE44374	Human coxsackievirus B2
2289.	BAE44370	Human coxsackievirus A4
2290.	BAE44369	Human coxsackievirus A4
2291.	BAE44368	Human coxsackievirus A4
2292.	BAE44367	Human coxsackievirus A4
2293.	BAE44366	Human coxsackievirus A4
2294.	BAE44365	Human coxsackievirus A4
2295.	BAD12585	Human echovirus 11
2296.	BAD12584	Human coxsackievirus A12
2297.	BAD12583	Human coxsackievirus A8
2298.	BAD12582	Human coxsackievirus A8
2299.	BAD12577	Human coxsackievirus A4
2300.	BAD12576	Human coxsackievirus A4
2301.	BAD12575	Human coxsackievirus A4
2302.	BAD12574	Human coxsackievirus A4
2303.	BAD12573	Human coxsackievirus A4
2304.	BAF02514	Human echovirus 11
2305.	BAF02513	Human echovirus 11
2306.	BAF02512	Human echovirus 11
2307.	BAF02511	Human echovirus 11
2308.	BAF02510	Human echovirus 11
2309.	BAF02509	Human echovirus 11
2310.	BAF02508	Human echovirus 11
2311.	BAF02507	Human echovirus 11
2312.	BAF02487	Human echovirus 13
2313.	BAF02486	Human echovirus 13
2314.	BAF02485	Human echovirus 13
2315.	BAF02484	Human echovirus 13
2316.	BAF02483	Human echovirus 13
2317.	BAF02482	Human echovirus 13
2318.	BAF02481	Human echovirus 13
2319.	BAF02480	Human echovirus 13
2320.	BAF02479	Human echovirus 13
2321.	BAF02478	Human echovirus 13

2322.	BAF02477	Human echovirus 13
2323.	BAF02476	Human echovirus 13
2324.	BAF02475	Human echovirus 13
2325.	BAF02474	Human echovirus 13
2326.	BAF02473	Human echovirus 13
2327.	BAF02471	Human echovirus 13
2328.	CAB53952	Human echovirus 30
2329.	CAB53951	Human echovirus 30
2330.	CAB53950	Human echovirus 30
2331.	CAB53949	Human echovirus 30
2332.	CAB53948	Human echovirus 30
2333.	BAL14863	Human echovirus 30
2334.	BAL14862	Human echovirus 30
2335.	BAL14857	Human echovirus 14
2336.	BAL14849	Human echovirus 11
2337.	BAL04484	Human enterovirus 71
2338.	BAL04483	Human enterovirus 71
2339.	BAL04481	Human enterovirus 71
2340.	BAL04479	Human enterovirus 71
2341.	BAL04476	Human enterovirus 71
2342.	BAL04475	Human enterovirus 71
2343.	BAL04485	Human enterovirus 71
2344.	BAL04482	Human enterovirus 71
2345.	BAL04480	Human enterovirus 71
2346.	BAL04478	Human enterovirus 71
2347.	BAL04477	Human enterovirus 71
2348.	BAL04474	Human enterovirus 71
2349.	BAL04473	Human enterovirus 71
2350.	BAL04472	Human enterovirus 71
2351.	BAH24182	Human coxsackievirus A4
2352.	BAJ09242	Human echovirus 30
2353.	ADE44302	Human coxsackievirus A24
2354.	ADE44301	Human coxsackievirus A24
2355.	ADO24472	Human echovirus 6
2356.	ADV91577	Human enterovirus 71
2357.	ADV91576	Human enterovirus 71
2358.	ADV91575	Human enterovirus 71
2359.	ADN68569	Human enterovirus 71
2360.	ADN68568	Human enterovirus 71
2361.	ADN68567	Human enterovirus 71
2362.	ADN68566	Human enterovirus 71
2363.	ADN68565	Human enterovirus 71
2364.	ADN68564	Human enterovirus 71
2365.	ADN68563	Human enterovirus 71

2366.	ADN68562	Human enterovirus 71
2367.	ADN68561	Human enterovirus 71
2368.	ADN68560	Human enterovirus 71
2369.	ADN68559	Human enterovirus 71
2370.	ADN68558	Human enterovirus 71
2371.	ADN68557	Human enterovirus 71
2372.	ADN68556	Human enterovirus 71
2373.	ADN68555	Human enterovirus 71
2374.	ADN68554	Human enterovirus 71
2375.	ADN68553	Human enterovirus 71
2376.	ADN68552	Human enterovirus 71
2377.	ADN68551	Human enterovirus 71
2378.	ADQ38962	Human coxsackievirus B1
2379.	ADO85710	Human poliovirus 1
2380.	ACV73757	Human poliovirus 3
2381.	ACV73756	Human coxsackievirus B5
2382.	ACV73755	Human coxsackievirus B5
2383.	ACV73754	Human coxsackievirus B5
2384.	ACV73753	Human coxsackievirus B4
2385.	ACV73752	Human coxsackievirus B4
2386.	ACV73751	Human coxsackievirus B4
2387.	ACV73750	Human coxsackievirus B3
2388.	ACV73749	Human coxsackievirus B3
2389.	ACV73748	Human coxsackievirus B3
2390.	ACV73747	Human coxsackievirus B1
2391.	ACV73746	Human coxsackievirus B1
2392.	ACV73745	Human coxsackievirus B1
2393.	ADU04526	Human echovirus 11
2394.	ADU04525	Human echovirus 11
2395.	ADU04524	Human echovirus 11
2396.	ADU04523	Human echovirus 11
2397.	ADU04522	Human echovirus 11
2398.	ADU04521	Human echovirus 11
2399.	ADU04520	Human echovirus 11
2400.	ADU04519	Human echovirus 11
2401.	ADW54322	Human poliovirus 1
2402.	ADW54321	Human poliovirus 1
2403.	ADW54320	Human poliovirus 1
2404.	ADW54319	Human poliovirus 1
2405.	ADW54318	Human poliovirus 1
2406.	ADW54317	Human poliovirus 1
2407.	ADW54316	Human poliovirus 1
2408.	ADW54315	Human poliovirus 1
2409.	ADW54314	Human poliovirus 1

2410.	ADW54313	Human poliovirus 1
2411.	ADW54312	Human poliovirus 1
2412.	ADW54311	Human poliovirus 1
2413.	ADW54310	Human poliovirus 1
2414.	ADW54309	Human poliovirus 1
2415.	ADW54308	Human poliovirus 1
2416.	ADW54307	Human poliovirus 1
2417.	ADW54306	Human poliovirus 1
2418.	ADW54305	Human poliovirus 1
2419.	ADW54304	Human poliovirus 1
2420.	ADW54303	Human poliovirus 1
2421.	ADW54302	Human poliovirus 1
2422.	ADW54301	Human poliovirus 1
2423.	ADW54300	Human poliovirus 1
2424.	ADW54299	Human poliovirus 1
2425.	ADW54298	Human poliovirus 1
2426.	ADW54297	Human poliovirus 1
2427.	ADE62186	Human echovirus 30
2428.	ADE62185	Human echovirus 30
2429.	ADE62184	Human echovirus 25
2430.	ADE62183	Human echovirus 18
2431.	ADE62182	Human echovirus 18
2432.	ADE62181	Human echovirus 18
2433.	ADE62180	Human echovirus 18
2434.	ADE62179	Human echovirus 18
2435.	ADE62178	Human echovirus 18
2436.	ADE62177	Human echovirus 13
2437.	ADE62176	Human echovirus 11
2438.	ADE62175	Human echovirus 9
2439.	ADE62174	Human echovirus 9
2440.	ADE62173	Human echovirus 9
2441.	ADE62172	Human echovirus 9
2442.	ADE62171	Human echovirus 9
2443.	ADE62170	Human echovirus 9
2444.	ADE62169	Human echovirus 7
2445.	ADE62168	Human echovirus 6
2446.	ADE62167	Human echovirus 6
2447.	ADE62166	Human echovirus 6
2448.	ADE62165	Human echovirus 6
2449.	ADE62164	Human echovirus 6
2450.	ADE62163	Human echovirus 6
2451.	ADE62162	Human echovirus 6
2452.	ADE62161	Human echovirus 6
2453.	ADE62160	Human echovirus 6

2454.	ADE62159	Human echovirus 5
2455.	ADE62158	Human coxsackievirus B4
2456.	ADE62157	Human coxsackievirus B4
2457.	ADE62156	Human coxsackievirus B4
2458.	ADE62155	Human coxsackievirus A9
2459.	ADE62154	Human enterovirus 71
2460.	ADE62153	Human enterovirus 71
2461.	ADE62152	Human enterovirus 71
2462.	ADE62151	Human enterovirus 71
2463.	ADE62150	Human coxsackievirus A16
2464.	ADE62149	Human coxsackievirus A16
2465.	ADE62148	Human coxsackievirus A16
2466.	ABH04341	Human poliovirus 2
2467.	ABH04340	Human poliovirus 2
2468.	ABH04339	Human poliovirus 2
2469.	ABH04338	Human poliovirus 2
2470.	ABH04337	Human poliovirus 2
2471.	ABH04336	Human poliovirus 2
2472.	ABH04335	Human poliovirus 2
2473.	ABH04334	Human poliovirus 2
2474.	ABH04333	Human poliovirus 2
2475.	ABH04332	Human poliovirus 2
2476.	ABH04331	Human poliovirus 2
2477.	ABH04330	Human poliovirus 1
2478.	ABH04329	Human poliovirus 1
2479.	ABH04328	Human poliovirus 1
2480.	ADU04526	Human echovirus 11
2481.	ADU04525	Human echovirus 11
2482.	ADU04524	Human echovirus 11
2483.	ADU04523	Human echovirus 11
2484.	ADU04522	Human echovirus 11
2485.	ADU04521	Human echovirus 11
2486.	ADU04520	Human echovirus 11
2487.	ADU04519	Human echovirus 11
2488.	ADW54322	Human poliovirus 1
2489.	ADW54321	Human poliovirus 1
2490.	ADW54320	Human poliovirus 1
2491.	ADW54319	Human poliovirus 1
2492.	ADW54318	Human poliovirus 1
2493.	ADW54317	Human poliovirus 1
2494.	ADW54316	Human poliovirus 1
2495.	ADW54315	Human poliovirus 1
2496.	ADW54314	Human poliovirus 1
2497.	ADW54313	Human poliovirus 1

2498.	ADW54312	Human poliovirus 1
2499.	ADW54311	Human poliovirus 1
2500.	ADW54310	Human poliovirus 1
2501.	ADW54309	Human poliovirus 1
2502.	ADW54308	Human poliovirus 1
2503.	ADW54307	Human poliovirus 1
2504.	ADW54306	Human poliovirus 1
2505.	ADW54305	Human poliovirus 1
2506.	ADW54304	Human poliovirus 1
2507.	ADW54303	Human poliovirus 1
2508.	ADW54302	Human poliovirus 1
2509.	ADW54301	Human poliovirus 1
2510.	ADW54300	Human poliovirus 1
2511.	ADW54299	Human poliovirus 1
2512.	ADW54298	Human poliovirus 1
2513.	ADW54297	Human poliovirus 1
2514.	ADE62186	Human echovirus 30
2515.	ADE62185	Human echovirus 30
2516.	ADE62184	Human echovirus 25
2517.	ADE62183	Human echovirus 18
2518.	ADE62182	Human echovirus 18
2519.	ADE62181	Human echovirus 18
2520.	ADE62180	Human echovirus 18
2521.	ADE62179	Human echovirus 18
2522.	ADE62178	Human echovirus 18
2523.	ADE62177	Human echovirus 13
2524.	ADE62176	Human echovirus 11
2525.	ADE62175	Human echovirus 9
2526.	ADE62174	Human echovirus 9
2527.	ADE62173	Human echovirus 9
2528.	ADE62172	Human echovirus 9
2529.	ADE62171	Human echovirus 9
2530.	ADE62170	Human echovirus 9
2531.	ADE62169	Human echovirus 7
2532.	ADE62168	Human echovirus 6
2533.	ADE62167	Human echovirus 6
2534.	ADE62166	Human echovirus 6
2535.	ADE62165	Human echovirus 6
2536.	ADE62164	Human echovirus 6
2537.	ADE62163	Human echovirus 6
2538.	ADE62162	Human echovirus 6
2539.	ADE62161	Human echovirus 6
2540.	ADE62160	Human echovirus 6
2541.	ADE62159	Human echovirus 5

2542.	ADE62158	Human coxsackievirus B4
2543.	ADE62157	Human coxsackievirus B4
2544.	ADE62156	Human coxsackievirus B4
2545.	ADE62155	Human coxsackievirus A9
2546.	ADE62154	Human enterovirus 71
2547.	ADE62153	Human enterovirus 71
2548.	ADE62152	Human enterovirus 71
2549.	ADE62151	Human enterovirus 71
2550.	ADE62150	Human coxsackievirus A16
2551.	ADE62149	Human coxsackievirus A16
2552.	ADE62148	Human coxsackievirus A16
2553.	ABH04341	Human poliovirus 2
2554.	ABH04340	Human poliovirus 2
2555.	ABH04339	Human poliovirus 2
2556.	ABH04338	Human poliovirus 2
2557.	ABH04337	Human poliovirus 2
2558.	ABH04336	Human poliovirus 2
2559.	ABH04335	Human poliovirus 2
2560.	ABH04334	Human poliovirus 2
2561.	ABH04333	Human poliovirus 2
2562.	ABH04332	Human poliovirus 2
2563.	ABH04331	Human poliovirus 2
2564.	ABH04330	Human poliovirus 1
2565.	ABH04329	Human poliovirus 1
2566.	ABH04328	Human poliovirus 1
2567.	BAC79093	Human coxsackievirus A6
2568.	BAC79092	Human coxsackievirus A6
2569.	BAC79091	Human coxsackievirus A6
2570.	BAC79090	Human coxsackievirus A6
2571.	BAC79089	Human coxsackievirus A6
2572.	BAC79088	Human coxsackievirus A6
2573.	BAC79087	Human coxsackievirus A6
2574.	BAC79086	Human coxsackievirus A6
2575.	BAC79085	Human coxsackievirus A6
2576.	BAC79078	Human coxsackievirus A4
2577.	AAS88607	Human echovirus 6
2578.	BAD11165	Human coxsackievirus A6
2579.	BAC79084	Human coxsackievirus A5
2580.	BAC79083	Human coxsackievirus A5
2581.	BAC79082	Human coxsackievirus A5
2582.	BAC79081	Human coxsackievirus A5
2583.	BAC79080	Human coxsackievirus A5
2584.	BAC79079	Human coxsackievirus A5
2585.	CBK52637	Human echovirus 6

2586.	CBK52636	Human echovirus 6
2587.	CBK52635	Human echovirus 6
2588.	CBK52634	Human echovirus 6
2589.	CBK52633	Human echovirus 6
2590.	CBK52632	Human echovirus 6
2591.	CBK52631	Human echovirus 6
2592.	CBK52630	Human echovirus 6
2593.	CBK52629	Human echovirus 6
2594.	CBK52628	Human echovirus 6
2595.	CBK52627	Human echovirus 6
2596.	CBK52626	Human echovirus 6
2597.	CBK52625	Human echovirus 6
2598.	CBK52624	Human echovirus 6
2599.	CBK52623	Human echovirus 6
2600.	CBK52622	Human echovirus 6
2601.	CBK52621	Human echovirus 6
2602.	CBK52620	Human echovirus 6
2603.	CBK52619	Human echovirus 6
2604.	CBK52618	Human echovirus 6
2605.	CBK52617	Human echovirus 6
2606.	CBK52616	Human echovirus 6
2607.	CBK52615	Human echovirus 6
2608.	CBK52614	Human echovirus 6
2609.	CBK52613	Human echovirus 6
2610.	CBK52612	Human echovirus 6
2611.	CBK52611	Human echovirus 6
2612.	CBK52610	Human echovirus 6
2613.	CBK52609	Human echovirus 6
2614.	CBK52608	Human echovirus 6
2615.	CBK52607	Human echovirus 6
2616.	CBK52606	Human echovirus 6
2617.	CBK52605	Human echovirus 6
2618.	CBK52604	Human echovirus 6
2619.	CBK52603	Human echovirus 6
2620.	CBK52602	Human echovirus 6
2621.	CBK52601	Human echovirus 6
2622.	CBK52600	Human echovirus 6
2623.	CBK52599	Human echovirus 6
2624.	ADV17629	Human poliovirus 2
2625.	ADV17628	Human poliovirus 2
2626.	ADV17627	Human poliovirus 1
2627.	ADV17626	Human poliovirus 1
2628.	ADV17625	Human poliovirus 1
2629.	ADV17624	Human poliovirus 1

2630.	ADV17623	Human poliovirus 1
2631.	ADV17622	Human poliovirus 1
2632.	ADV17621	Human poliovirus 1
2633.	ADV17620	Human poliovirus 1
2634.	ADV17619	Human poliovirus 1
2635.	ADV17618	Human poliovirus 1
2636.	ADV17617	Human poliovirus 1
2637.	ADV17616	Human poliovirus 1
2638.	ADV17615	Human poliovirus 3
2639.	ADV17614	Human poliovirus 3
2640.	ADV17613	Human poliovirus 3
2641.	ADV17612	Human poliovirus 3
2642.	ADV17611	Human poliovirus 3
2643.	ADV17610	Human poliovirus 3
2644.	ADV17609	Human poliovirus 3
2645.	ADV17608	Human poliovirus 3
2646.	ADV17607	Human poliovirus 3
2647.	ADV17606	Human poliovirus 3
2648.	ADV17605	Human poliovirus 3
2649.	ADV17604	Human poliovirus 3
2650.	ADV17603	Human poliovirus 3
2651.	ADV17602	Human poliovirus 3
2652.	ADV17601	Human poliovirus 3
2653.	ADD81985	Human poliovirus 1
2654.	ADD81984	Human poliovirus 1
2655.	ADD81983	Human poliovirus 1
2656.	ADD81982	Human poliovirus 1
2657.	ADD81981	Human poliovirus 3
2658.	ADD81980	Human poliovirus 3
2659.	ADD81979	Human poliovirus 3
2660.	ADD81978	Human poliovirus 3
2661.	ADD81977	Human poliovirus 3
2662.	ADD81976	Human poliovirus 3
2663.	ADD81975	Human poliovirus 3
2664.	ADD81974	Human poliovirus 1
2665.	ABS29715	Human echovirus 30
2666.	ABS29714	Human echovirus 30
2667.	ABS29713	Human echovirus 30
2668.	ABS29712	Human echovirus 30
2669.	ABS29711	Human echovirus 30
2670.	ABS29710	Human echovirus 30
2671.	ABS29709	Human echovirus 30
2672.	ABS29708	Human echovirus 30
2673.	ABS29707	Human echovirus 30

2674.	ABS29706	Human echovirus 30
2675.	ABS29705	Human echovirus 30
2676.	ABS29704	Human echovirus 30
2677.	ABS29703	Human echovirus 30
2678.	ABS29702	Human echovirus 30
2679.	AAS94267	Human poliovirus 1
2680.	AAS94266	Human poliovirus 1
2681.	AAS94265	Human poliovirus 1
2682.	AAS94264	Human poliovirus 1
2683.	AAS94263	Human poliovirus 1
2684.	AAS94262	Human poliovirus 1
2685.	AAS94261	Human poliovirus 1
2686.	AAS94260	Human poliovirus 1
2687.	AAS94259	Human poliovirus 1
2688.	AAS94258	Human poliovirus 1
2689.	AAS94257	Human poliovirus 1
2690.	ACZ13358	Human poliovirus 3
2691.	ACZ13356	Human poliovirus 3
2692.	ACZ13355	Human poliovirus 3
2693.	AAL89594.2	Human poliovirus 3
2694.	BAF95280	Human echovirus 12
2695.	BAF95279	Human echovirus 12
2696.	BAF95278	Human echovirus 11
2697.	BAF95274	Human echovirus 11
2698.	BAF95273	Human echovirus 11
2699.	BAF95272	Human echovirus 9
2700.	BAF95271	Human echovirus 9
2701.	BAF95270	Human echovirus 7
2702.	BAF95269	Human echovirus 7
2703.	BAF95268	Human echovirus 7
2704.	BAF95266	Human echovirus 7
2705.	BAF95265	Human echovirus 7
2706.	BAF95263	Human echovirus 6
2707.	BAF95262	Human echovirus 6
2708.	BAF95261	Human echovirus 6
2709.	BAF95258	Human echovirus 6
2710.	BAF95257	Human echovirus 6
2711.	BAF95256	Human echovirus 6
2712.	BAF95255	Human echovirus 6
2713.	BAF95253	Human echovirus 3
2714.	BAF95249	Human echovirus 3
2715.	BAF95248	Human echovirus 2
2716.	BAF95246	Human echovirus 2
2717.	BAF95245	Human echovirus 2

2718.	BAF95244	Human echovirus 2
2719.	BAF95243	Human echovirus 2
2720.	BAF95242	Human echovirus 2
2721.	BAF95241	Human echovirus 2
2722.	BAF95240	Human echovirus 2
2723.	BAF95239	Human echovirus 2
2724.	BAF95238	Human echovirus 1
2725.	BAF95235	Human echovirus 1
2726.	BAF95234	Human echovirus 1
2727.	BAF95232	Human echovirus 1
2728.	BAF95224	Human coxsackievirus B3
2729.	BAF95221	Human coxsackievirus B3
2730.	BAF95220	Human coxsackievirus B3
2731.	BAF95219	Human coxsackievirus B3
2732.	BAF95218	Human coxsackievirus B3
2733.	BAF95217	Human coxsackievirus B3
2734.	BAF95212	Human coxsackievirus B1
2735.	BAF95211	Human coxsackievirus A9
2736.	BAF95210	Human coxsackievirus A9
2737.	BAF95208	Human coxsackievirus A9
2738.	BAE47905	Human echovirus 33
2739.	BAE47904	Human echovirus 33
2740.	BAE47902	Human echovirus 25
2741.	BAE47901	Human echovirus 12
2742.	CAQ16959	Human echovirus 30
2743.	CAQ16958	Human echovirus 30
2744.	CAQ16957	Human echovirus 30
2745.	CAQ16956	Human echovirus 30
2746.	CAQ16955	Human echovirus 30
2747.	CAQ16954	Human echovirus 30
2748.	CAQ16953	Human echovirus 30
2749.	CAQ16952	Human echovirus 30
2750.	CAQ16951	Human echovirus 30
2751.	CAQ16950	Human echovirus 30
2752.	CAQ16949	Human echovirus 30
2753.	CAQ16948	Human echovirus 30
2754.	CAQ16947	Human echovirus 30
2755.	CAQ16946	Human echovirus 30
2756.	CAQ16945	Human echovirus 30
2757.	CAQ16944	Human echovirus 30
2758.	CAQ16943	Human echovirus 30
2759.	CAQ16942	Human echovirus 30
2760.	CAQ16941	Human echovirus 30
2761.	CAQ16940	Human echovirus 30

2762.	CAQ16939	Human echovirus 30
2763.	CAQ16938	Human echovirus 30
2764.	CAQ16937	Human echovirus 30
2765.	BAC79114	Human coxsackievirus A10
2766.	BAC79113	Human coxsackievirus A10
2767.	BAC79112	Human coxsackievirus A10
2768.	BAC79111	Human coxsackievirus A10
2769.	BAC79110	Human coxsackievirus A10
2770.	BAC79109	Human coxsackievirus A10
2771.	BAC79108	Human coxsackievirus A10
2772.	BAC79107	Human coxsackievirus A6
2773.	BAC79106	Human coxsackievirus A6
2774.	BAC79105	Human coxsackievirus A6
2775.	BAC79104	Human coxsackievirus A6
2776.	BAC79103	Human coxsackievirus A6
2777.	BAC79102	Human coxsackievirus A6
2778.	BAC79101	Human coxsackievirus A6
2779.	BAC79100	Human coxsackievirus A6
2780.	BAC79099	Human coxsackievirus A6
2781.	BAC79098	Human coxsackievirus A6
2782.	BAC79097	Human coxsackievirus A6
2783.	BAC79096	Human coxsackievirus A6
2784.	BAC79095	Human coxsackievirus A6
2785.	BAC79094	Human coxsackievirus A6
2786.	AAO27429	Human poliovirus 3
2787.	AAO27428	Human poliovirus 3
2788.	AAO27427	Human poliovirus 3
2789.	AAO27426	Human poliovirus 3
2790.	AAO27425	Human poliovirus 3
2791.	AAO27424	Human poliovirus 3
2792.	AAO27423	Human poliovirus 3
2793.	AAO27422	Human poliovirus 3
2794.	AAO27421	Human poliovirus 3
2795.	AAO27420	Human poliovirus 3
2796.	AAO27419	Human poliovirus 3
2797.	AAO27418	Human poliovirus 3
2798.	AAO27417	Human poliovirus 3
2799.	AAO27416	Human poliovirus 3
2800.	AAO27415	Human poliovirus 3
2801.	AAO27414	Human poliovirus 3
2802.	AAO27413	Human poliovirus 3
2803.	AAO27412	Human poliovirus 3
2804.	AAO27411	Human poliovirus 3
2805.	AAO27410	Human poliovirus 3

2806.	AAO27409	Human poliovirus 3
2807.	AAO27408	Human poliovirus 1
2808.	AAO27407	Human poliovirus 1
2809.	AAO27406	Human poliovirus 1
2810.	AAO27405	Human poliovirus 1
2811.	AAO27404	Human poliovirus 1
2812.	AAO27403	Human poliovirus 1
2813.	AAO27402	Human poliovirus 1
2814.	AAO27401	Human poliovirus 1
2815.	AAO27400	Human poliovirus 1
2816.	AAO27399	Human poliovirus 1
2817.	AAO27398	Human poliovirus 1
2818.	AAO27397	Human poliovirus 1
2819.	AAO27396	Human poliovirus 1
2820.	AAO27395	Human poliovirus 1
2821.	AAO27394	Human poliovirus 1
2822.	AAO27393	Human poliovirus 1
2823.	AAO27392	Human poliovirus 1
2824.	AAO27391	Human poliovirus 1
2825.	AAO27390	Human poliovirus 1
2826.	AAO27389	Human poliovirus 1
2827.	AAO27388	Human poliovirus 1
2828.	AAO27387	Human poliovirus 1
2829.	AAO27386	Human poliovirus 1
2830.	AAO27385	Human poliovirus 1
2831.	AAO27384	Human poliovirus 1
2832.	AAO27383	Human poliovirus 1
2833.	AAO27382	Human poliovirus 1
2834.	AAO27381	Human poliovirus 1
2835.	AAO27380	Human poliovirus 1
2836.	AAO27379	Human poliovirus 1
2837.	AAO27378	Human poliovirus 1
2838.	AAO27377	Human poliovirus 1
2839.	AAO27376	Human poliovirus 1
2840.	AAO27375	Human poliovirus 1
2841.	AAO27374	Human poliovirus 1
2842.	AAO27373	Human poliovirus 1
2843.	AAO27372	Human poliovirus 1
2844.	AAO27371	Human poliovirus 1
2845.	AAO27370	Human poliovirus 1
2846.	AAO27369	Human poliovirus 1
2847.	AAO27368	Human poliovirus 1
2848.	AAL92484.2	Human poliovirus 2
2849.	AAL89595.2	Human poliovirus 1

2850.	AAL92487	Human poliovirus 2
2851.	AAL92486	Human poliovirus 2
2852.	AAL92485	Human poliovirus 2
2853.	AAL92483	Human poliovirus 2
2854.	AAL89599	Human poliovirus 1
2855.	AAL89598	Human poliovirus 1
2856.	AAL89597	Human poliovirus 1
2857.	AAL89596	Human poliovirus 1
2858.	AAL89593	Human poliovirus 3
2859.	AAL89592	Human poliovirus 3
2860.	AAL89591	Human poliovirus 3
2861.	AAL89590	Human poliovirus 3
2862.	CAM97633.3	Human echovirus 13
2863.	CAM97644.3	Human echovirus 18
2864.	CAM97624.3	Human echovirus 13
2865.	CAM97651.3	Human echovirus 18
2866.	CAM97730	Human coxsackievirus B4
2867.	CAM97729	Human echovirus 13
2868.	CAM97728	Human echovirus 25
2869.	CAM97727	Human echovirus 18
2870.	CAM97726	Human echovirus 9
2871.	CAM97725	Human echovirus 18
2872.	CAM97724	Human echovirus 13
2873.	CAM97723	Human echovirus 18
2874.	CAM97722	Human echovirus 6
2875.	CAM97721	Human echovirus 13
2876.	CAM97720	Human echovirus 13
2877.	CAM97719	Human echovirus 13
2878.	CAM97718	Human echovirus 6
2879.	CAM97717	Human echovirus 6
2880.	CAM97716	Human coxsackievirus B5
2881.	CAM97715	Human coxsackievirus B5
2882.	CAM97714	Human echovirus 13
2883.	CAM97713	Human echovirus 6
2884.	CAM97712	Human coxsackievirus B3
2885.	CAM97711	Human echovirus 13
2886.	CAM97710	Human echovirus 13
2887.	CAM97709	Human echovirus 6
2888.	CAM97708	Human echovirus 25
2889.	CAM97707	Human coxsackievirus B5
2890.	CAM97706	Human echovirus 30
2891.	CAM97705	Human echovirus 6
2892.	CAM97704	Human echovirus 6
2893.	CAM97703	Human coxsackievirus B1

2894.	CAM97702	Human coxsackievirus B5
2895.	CAM97701	Human echovirus 25
2896.	CAM97700	Human coxsackievirus B4
2897.	CAM97699	Human echovirus 9
2898.	CAM97698	Human coxsackievirus B2
2899.	CAM97697	Human echovirus 13
2900.	CAM97696	Human echovirus 30
2901.	CAM97695	Human echovirus 18
2902.	CAM97694	Human echovirus 9
2903.	CAM97693	Human echovirus 13
2904.	CAM97692	Human echovirus 18
2905.	CAM97691	Human echovirus 6
2906.	CAM97690	Human echovirus 18
2907.	CAM97689	Human echovirus 13
2908.	CAM97688	Human echovirus 6
2909.	CAM97687	Human echovirus 13
2910.	CAM97686	Human coxsackievirus B4
2911.	CAM97685	Human coxsackievirus B5
2912.	CAM97684	Human echovirus 13
2913.	CAM97683	Human coxsackievirus B3
2914.	CAM97682	Human echovirus 13
2915.	CAM97681	Human echovirus 13
2916.	CAM97680	Human echovirus 13
2917.	CAM97679	Human coxsackievirus B5
2918.	CAM97678	Human coxsackievirus B2
2919.	CAM97677	Human echovirus 6
2920.	CAM97676	Human echovirus 30
2921.	CAM97675	Human coxsackievirus B5
2922.	CAM97674	Human coxsackievirus B5
2923.	CAM97673	Human echovirus 13
2924.	CAM97672	Human coxsackievirus B5
2925.	CAM97671	Human echovirus 13
2926.	CAM97670	Human echovirus 6
2927.	CAM97669	Human echovirus 6
2928.	CAM97668	Human echovirus 6
2929.	CAM97667	Human echovirus 6
2930.	CAM97666	Human echovirus 13
2931.	CAM97665	Human coxsackievirus B1
2932.	CAM97664	Human echovirus 13
2933.	CAM97663	Human echovirus 21
2934.	CAM97662	Human echovirus 13
2935.	CAM97661	Human echovirus 27
2936.	CAM97659	Human echovirus 30
2937.	CAM97658	Human echovirus 30

2938.	CAM97657	Human echovirus 30
2939.	CAM97656	Human echovirus 30
2940.	CAM97655	Human echovirus 30
2941.	CAM97654	Human echovirus 30
2942.	CAM97653	Human echovirus 30
2943.	CAM97652	Human echovirus 30
2944.	CAM97650	Human echovirus 30
2945.	CAM97649	Human coxsackievirus B3
2946.	CAM97648	Human echovirus 30
2947.	CAM97647	Human echovirus 18
2948.	CAM97646	Human echovirus 30
2949.	CAM97645	Human echovirus 30
2950.	CAM97642	Human echovirus 30
2951.	CAM97641	Human echovirus 30
2952.	CAM97640	Human coxsackievirus B5
2953.	CAM97639	Human echovirus 30
2954.	CAM97638	Human echovirus 33
2955.	CAM97637	Human echovirus 30
2956.	CAM97636	Human echovirus 30
2957.	CAM97635	Human echovirus 30
2958.	CAM97634	Human coxsackievirus B5
2959.	CAM97632	Human echovirus 30
2960.	CAM97631	Human echovirus 30
2961.	CAM97630	Human echovirus 30
2962.	CAM97629	Human echovirus 30
2963.	CAM97628	Human coxsackievirus B3
2964.	CAM97627	Human echovirus 30
2965.	CAM97626	Human echovirus 11
2966.	CAM97625	Human echovirus 30
2967.	CAM97623	Human echovirus 30
2968.	CAM97622	Human coxsackievirus B3
2969.	CAM97621	Human echovirus 30
2970.	CAM97620	Human coxsackievirus B5
2971.	CAM97619	Human echovirus 30
2972.	CAQ15744	Human coxsackievirus B4
2973.	CAQ15743	Human coxsackievirus B4
2974.	CAQ15742	Human coxsackievirus B4
2975.	CAQ15741	Human coxsackievirus B4
2976.	CAQ15740	Human coxsackievirus B4
2977.	BAC10667	Human coxsackievirus A6
2978.	BAC10664	Human coxsackievirus A6
2979.	BAD11171	Human coxsackievirus A12
2980.	BAD11170	Human coxsackievirus A12
2981.	BAD11169	Human coxsackievirus A10

2982.	BAD11168	Human coxsackievirus A10
2983.	BAD11167	Human coxsackievirus A10
2984.	BAD11166	Human coxsackievirus A10
2985.	BAD11164	Human coxsackievirus A5
2986.	BAD11163	Human coxsackievirus A5
2987.	BAD11162	Human coxsackievirus A4
2988.	BAD11161	Human coxsackievirus A2
2989.	BAD11160	Human coxsackievirus A2
2990.	BAD11159	Human coxsackievirus A2
2991.	CAJ21288	Human poliovirus 1
2992.	CAJ21287	Human poliovirus 1
2993.	CAO81991	Human poliovirus 2
2994.	CAO81990	Human poliovirus 2
2995.	CAM92321	Human poliovirus 3
2996.	CAM92320	Human poliovirus 3
2997.	CAM92319	Human poliovirus 3
2998.	CAM92318	Human poliovirus 3
2999.	CAM92317	Human poliovirus 3
3000.	CAM92316	Human poliovirus 3
3001.	CAM92315	Human poliovirus 3
3002.	CAJ85816	Human echovirus 30
3003.	CAJ85815	Human echovirus 30
3004.	CAJ85814	Human echovirus 30
3005.	CAJ85813	Human echovirus 30
3006.	CAJ85812	Human echovirus 30
3007.	CAJ85811	Human echovirus 30
3008.	CAJ85810	Human echovirus 30
3009.	CAJ85809	Human echovirus 30
3010.	CAJ85808	Human echovirus 30
3011.	CAJ85807	Human echovirus 30
3012.	CAJ85806	Human echovirus 30
3013.	CAJ85805	Human echovirus 30
3014.	CAJ85804	Human echovirus 30
3015.	CAJ85803	Human echovirus 30
3016.	CAJ85802	Human echovirus 30
3017.	CAJ85801	Human echovirus 30
3018.	CAJ85800	Human echovirus 30
3019.	CAJ85799	Human echovirus 30
3020.	CAJ85798	Human echovirus 30
3021.	CAJ85797	Human echovirus 30
3022.	CAJ85796	Human echovirus 30
3023.	CAJ85795	Human echovirus 30
3024.	CAJ85794	Human echovirus 30
3025.	CAJ85793	Human echovirus 30

3026.	CAD61284	Human echovirus 13
3027.	CAD61283	Human echovirus 13
3028.	CAD61282	Human echovirus 13
3029.	CAD61281	Human echovirus 13
3030.	CAD61280	Human echovirus 13
3031.	CAD61279	Human echovirus 13
3032.	CAO91527.2	Human enterovirus C
3033.	CAO91522.2	Human enterovirus C
3034.	CAO91529.2	Human enterovirus C
3035.	CAO91528.2	Human enterovirus C
3036.	CAO91526.2	Human enterovirus C
3037.	CAO91524.2	Human enterovirus C
3038.	CAO91523.2	Human enterovirus C
3039.	CAO91521.2	Human enterovirus C
3040.	CAO91519.2	Human enterovirus C
3041.	CAO91518.2	Human enterovirus C
3042.	CAO91517.2	Human enterovirus C
3043.	CAO91515.2	Human enterovirus C
3044.	CAO91513.2	Human enterovirus C
3045.	CAO91512.2	Human enterovirus C
3046.	CAO91511.2	Human enterovirus C
3047.	CAO91508.2	Human enterovirus C
3048.	CAO91507.2	Human enterovirus C
3049.	CAO91505.2	Human enterovirus C
3050.	CAO91503.2	Human enterovirus C
3051.	CAO91500.2	Human enterovirus C
3052.	CAO91499.2	Human enterovirus C
3053.	CAO91498.2	Human enterovirus C
3054.	CAO91494.2	Human enterovirus C
3055.	CAO91493.2	Human enterovirus C
3056.	CAO91492.2	Human enterovirus C
3057.	CAO91491.2	Human enterovirus C
3058.	CAO91489.2	Human enterovirus C
3059.	CAO91488.2	Human enterovirus C
3060.	CAO91487.2	Human enterovirus C
3061.	CAO91485.2	Human enterovirus C
3062.	CAO91484.2	Human enterovirus C
3063.	AAD50446	Human coxsackievirus B3
3064.	ADM13768	Human coxsackievirus A24
3065.	ADM13767	Human coxsackievirus A24
3066.	ADM13766	Human coxsackievirus A24
3067.	ADM13765	Human coxsackievirus A21
3068.	ADM13764	Human coxsackievirus A21
3069.	ADM13763	Human coxsackievirus A21

3070.	ADM13762	Human coxsackievirus A20
3071.	ADM13761	Human enterovirus 96
3072.	ACK38201	Human enterovirus 71
3073.	ACK38200	Human enterovirus 71
3074.	ACK38199	Human enterovirus 71
3075.	ACK38198	Human enterovirus 71
3076.	ACK38197	Human enterovirus 71
3077.	ACK38196	Human enterovirus 71
3078.	ACK38195	Human enterovirus 71
3079.	ACK38194	Human enterovirus 71
3080.	ACK38193	Human enterovirus 71
3081.	AAC36125	Human poliovirus 1
3082.	AAC36124	Human poliovirus 1
3083.	AAC36123	Human poliovirus 1
3084.	AAC36122	Human poliovirus 1
3085.	AAC36121	Human poliovirus 1
3086.	AAC36120	Human poliovirus 1
3087.	AAC36119	Human poliovirus 1
3088.	AAG24528	Human poliovirus 2
3089.	AAG24527	Human poliovirus 2
3090.	AAG24526	Human poliovirus 2
3091.	AAG24525	Human poliovirus 2
3092.	AAG24524	Human poliovirus 2
3093.	AAG24523	Human poliovirus 2
3094.	ACH95444	Human enterovirus 71
3095.	ACH95443	Human enterovirus 71
3096.	ACH95442	Human enterovirus 71
3097.	ACH95441	Human enterovirus 71
3098.	ACH95440	Human enterovirus 71
3099.	ACH95439	Human enterovirus 71
3100.	ACH95438	Human enterovirus 71
3101.	ACH95437	Human enterovirus 71
3102.	ACH95436	Human enterovirus 71
3103.	ACH95435	Human enterovirus 71
3104.	ACH95434	Human enterovirus 71
3105.	ACH95433	Human enterovirus 71
3106.	ADI95489.1	Human poliovirus 3
3107.	ADI95488.1	Human poliovirus 3
3108.	ADI95487.1	Human poliovirus 3
3109.	ADI95486.1	Human poliovirus 3
3110.	ADI95485.1	Human poliovirus 3
3111.	ADD85106.1	Human echovirus 3
3112.	AAZ76205.1	Human coxsackievirus B3
3113.	AAZ76204.1	Human coxsackievirus B3

3114.	AAZ76203.1	Human coxsackievirus B3
3115.	AAZ76202.1	Human coxsackievirus B3
3116.	AAZ76201.1	Human coxsackievirus B3
3117.	AAZ76200.1	Human coxsackievirus B3
3118.	AAZ76199.1	Human coxsackievirus B3
3119.	AAZ76198.1	Human coxsackievirus B3
3120.	AAZ76197.1	Human coxsackievirus B3
3121.	AAZ76196.1	Human coxsackievirus B3
3122.	AAZ76195.1	Human coxsackievirus B3
3123.	AAZ76194.1	Human coxsackievirus B3
3124.	AAZ76193.1	Human coxsackievirus B3
3125.	AAZ76192.1	Human coxsackievirus B3
3126.	AAZ76191.1	Human coxsackievirus B3
3127.	AAZ76190.1	Human coxsackievirus B3
3128.	AAZ76189.1	Human coxsackievirus B3
3129.	AAZ76188.1	Human coxsackievirus B3
3130.	AAZ76187.1	Human coxsackievirus B3
3131.	AAZ76186.1	Human coxsackievirus B3
3132.	AAZ76185.1	Human coxsackievirus B3
3133.	AAZ76184.1	Human coxsackievirus B3
3134.	AAO59354.1	Human echovirus 18
3135.	CAB77530.1	Human poliovirus 2
3136.	CAB81766.1	Human echovirus 30
3137.	CAB81765.1	Human echovirus 30
3138.	CAB81764.1	Human echovirus 30
3139.	CAB81763.1	Human echovirus 30
3140.	BAK26733.1	Human coxsackievirus A16
3141.	BAK26732.1	Human coxsackievirus A16
3142.	BAK26731.1	Human coxsackievirus A16
3143.	BAK26730.1	Human coxsackievirus A16
3144.	BAK26729.1	Human coxsackievirus A16
3145.	BAK26728.1	Human coxsackievirus A16
3146.	BAK26727.1	Human coxsackievirus A16
3147.	BAK26726.1	Human coxsackievirus A16
3148.	BAK26725.1	Human coxsackievirus A16
3149.	BAK26724.1	Human coxsackievirus A16
3150.	BAK26723.1	Human coxsackievirus A16
3151.	BAK26722.1	Human coxsackievirus A16
3152.	BAK26721.1	Human coxsackievirus A16
3153.	BAK26720.1	Human coxsackievirus A16
3154.	BAK26719.1	Human coxsackievirus A16
3155.	BAK26718.1	Human coxsackievirus A16
3156.	BAK26717.1	Human coxsackievirus A16
3157.	BAK26716.1	Human coxsackievirus A16

3158.	BAK26715.1	Human coxsackievirus A16
3159.	BAK26714.1	Human coxsackievirus A16
3160.	BAK26713.1	Human coxsackievirus A16
3161.	BAK26712.1	Human coxsackievirus A16
3162.	BAK26711.1	Human coxsackievirus A16
3163.	BAK26710.1	Human coxsackievirus A16
3164.	BAK26709.1	Human coxsackievirus A16
3165.	BAK26708.1	Human coxsackievirus A16
3166.	BAK26707.1	Human coxsackievirus A16
3167.	BAK26706.1	Human coxsackievirus A16
3168.	BAK26705.1	Human coxsackievirus A16
3169.	BAK26704.1	Human coxsackievirus A16
3170.	BAK26703.1	Human coxsackievirus A16
3171.	BAK26702.1	Human coxsackievirus A16
3172.	BAK26701.1	Human coxsackievirus A16
3173.	BAK26700.1	Human coxsackievirus A16
3174.	BAK26699.1	Human coxsackievirus A16
3175.	BAK26698.1	Human coxsackievirus A16
3176.	BAK26697.1	Human coxsackievirus A16
3177.	BAK26696.1	Human coxsackievirus A16
3178.	BAK26695.1	Human coxsackievirus A16
3179.	BAK26694.1	Human coxsackievirus A16
3180.	BAK26693.1	Human coxsackievirus A16
3181.	BAK26692.1	Human coxsackievirus A16
3182.	BAK26691.1	Human coxsackievirus A16
3183.	BAK26690.1	Human coxsackievirus A16
3184.	BAK26689.1	Human coxsackievirus A16
3185.	BAK26688.1	Human coxsackievirus A16
3186.	BAK26687.1	Human coxsackievirus A16
3187.	BAK26686.1	Human coxsackievirus A16
3188.	BAK26685.1	Human coxsackievirus A16
3189.	BAK26684.1	Human coxsackievirus A16
3190.	BAK26683.1	Human coxsackievirus A16
3191.	BAK26682.1	Human coxsackievirus A16
3192.	BAK26681.1	Human coxsackievirus A16
3193.	BAK26680.1	Human coxsackievirus A16
3194.	BAK26679.1	Human coxsackievirus A16
3195.	BAK26678.1	Human coxsackievirus A16
3196.	BAK26677.1	Human coxsackievirus A16
3197.	BAK26676.1	Human coxsackievirus A16
3198.	BAK26675.1	Human coxsackievirus A16
3199.	BAK26674.1	Human coxsackievirus A16
3200.	BAK26673.1	Human coxsackievirus A16
3201.	BAK26672.1	Human coxsackievirus A16

3202.	BAK26671.1	Human coxsackievirus A16
3203.	BAK26670.1	Human coxsackievirus A16
3204.	BAK26669.1	Human coxsackievirus A16
3205.	BAK26668.1	Human coxsackievirus A16
3206.	BAK26667.1	Human coxsackievirus A16
3207.	BAK26666.1	Human coxsackievirus A16
3208.	BAK26665.1	Human coxsackievirus A16
3209.	BAK26664.1	Human coxsackievirus A16
3210.	BAK26663.1	Human coxsackievirus A16
3211.	BAK26662.1	Human coxsackievirus A16
3212.	BAK26661.1	Human coxsackievirus A16
3213.	BAK26660.1	Human coxsackievirus A16
3214.	BAK26659.1	Human coxsackievirus A16
3215.	BAK26658.1	Human coxsackievirus A16
3216.	BAK26657.1	Human coxsackievirus A16
3217.	BAK26656.1	Human coxsackievirus A16
3218.	BAK26655.1	Human coxsackievirus A16
3219.	BAK26654.1	Human coxsackievirus A16
3220.	BAK26653.1	Human coxsackievirus A16
3221.	BAK26652.1	Human coxsackievirus A16
3222.	BAK26651.1	Human coxsackievirus A16
3223.	BAK26650.1	Human coxsackievirus A16
3224.	BAK26649.1	Human coxsackievirus A16
3225.	BAK26648.1	Human coxsackievirus A16
3226.	BAK26647.1	Human coxsackievirus A16
3227.	BAK26646.1	Human coxsackievirus A16
3228.	BAK26645.1	Human coxsackievirus A16
3229.	BAK26644.1	Human coxsackievirus A16
3230.	BAK26643.1	Human coxsackievirus A16
3231.	BAK26642.1	Human coxsackievirus A16
3232.	BAK26641.1	Human coxsackievirus A16
3233.	BAK26640.1	Human coxsackievirus A16
3234.	BAK26639.1	Human coxsackievirus A16
3235.	BAK26638.1	Human coxsackievirus A16
3236.	BAK26637.1	Human coxsackievirus A16
3237.	BAK26636.1	Human coxsackievirus A16
3238.	BAK26635.1	Human coxsackievirus A16
3239.	BAK26634.1	Human coxsackievirus A16
3240.	BAK26633.1	Human coxsackievirus A16
3241.	BAK26632.1	Human coxsackievirus A16
3242.	BAK26631.1	Human coxsackievirus A16
3243.	BAK26630.1	Human coxsackievirus A16
3244.	BAK26629.1	Human coxsackievirus A16
3245.	BAK26628.1	Human coxsackievirus A16

3246.	BAK26627.1	Human coxsackievirus A16
3247.	BAK26626.1	Human coxsackievirus A16
3248.	BAK26625.1	Human coxsackievirus A16
3249.	BAK26624.1	Human coxsackievirus A16
3250.	BAK26623.1	Human coxsackievirus A16
3251.	BAK26622.1	Human coxsackievirus A16
3252.	BAK26621.1	Human coxsackievirus A16
3253.	BAK26620.1	Human coxsackievirus A16
3254.	BAK26619.1	Human coxsackievirus A16
3255.	BAK26618.1	Human coxsackievirus A16
3256.	BAK26617.1	Human coxsackievirus A16
3257.	BAK26616.1	Human coxsackievirus A16
3258.	BAK26615.1	Human coxsackievirus A16
3259.	BAK26614.1	Human coxsackievirus A16
3260.	BAK26613.1	Human coxsackievirus A16
3261.	BAK26612.1	Human coxsackievirus A16
3262.	BAK26611.1	Human coxsackievirus A16
3263.	BAK26610.1	Human coxsackievirus A16
3264.	BAK26609.1	Human coxsackievirus A16
3265.	BAK26608.1	Human coxsackievirus A16
3266.	BAK26607.1	Human coxsackievirus A16
3267.	BAK26606.1	Human coxsackievirus A16
3268.	BAK26605.1	Human coxsackievirus A16
3269.	BAK26604.1	Human coxsackievirus A16
3270.	BAK26603.1	Human coxsackievirus A16
3271.	BAK26602.1	Human coxsackievirus A16
3272.	BAK26601.1	Human coxsackievirus A16
3273.	BAK26600.1	Human coxsackievirus A16
3274.	BAK26599.1	Human coxsackievirus A16
3275.	BAK26598.1	Human coxsackievirus A16
3276.	BAK26597.1	Human coxsackievirus A16
3277.	BAK26596.1	Human coxsackievirus A16
3278.	BAK26595.1	Human coxsackievirus A16
3279.	BAK26594.1	Human coxsackievirus A16
3280.	BAK26593.1	Human coxsackievirus A16
3281.	BAK26592.1	Human coxsackievirus A16
3282.	BAK26591.1	Human coxsackievirus A16
3283.	BAK26590.1	Human coxsackievirus A16
3284.	BAK26589.1	Human coxsackievirus A16
3285.	BAK26588.1	Human coxsackievirus A16
3286.	BAK26587.1	Human coxsackievirus A16
3287.	BAK26586.1	Human coxsackievirus A16
3288.	BAK26585.1	Human coxsackievirus A16
3289.	BAK26584.1	Human coxsackievirus A16

3290.	BAK26583.1	Human coxsackievirus A16
3291.	BAK26582.1	Human coxsackievirus A16
3292.	BAK26581.1	Human coxsackievirus A16
3293.	BAK26580.1	Human coxsackievirus A16
3294.	BAK26579.1	Human coxsackievirus A16
3295.	BAK26578.1	Human coxsackievirus A16
3296.	BAK26577.1	Human coxsackievirus A16
3297.	BAK26576.1	Human coxsackievirus A16
3298.	BAK26575.1	Human coxsackievirus A16
3299.	BAK26574.1	Human coxsackievirus A16
3300.	BAK26573.1	Human coxsackievirus A16
3301.	BAK26572.1	Human coxsackievirus A16
3302.	BAK26571.1	Human coxsackievirus A16
3303.	BAK26570.1	Human coxsackievirus A16
3304.	BAK26569.1	Human coxsackievirus A16
3305.	BAK26568.1	Human coxsackievirus A16
3306.	BAK26567.1	Human coxsackievirus A16
3307.	ADG36488.1	Human echovirus 19
3308.	ACR07935.1	Human coxsackievirus B3
3309.	ACR07934.1	Human coxsackievirus B3
3310.	ACR07933.1	Human echovirus 30
3311.	ACR07932.1	Human coxsackievirus B3
3312.	ACC55255.1	Human echovirus 9
3313.	AAO59375.1	Human echovirus 13
3314.	AAO59364.1	Human coxsackievirus A24
3315.	AAO59363.1	Human coxsackievirus A24
3316.	AAO59357.1	Human enterovirus 74
3317.	AAO59353.1	Human echovirus 18
3318.	CAB77542.1	Human poliovirus 3
3319.	CAB77540.1	Human poliovirus 2
3320.	CAB77539.1	Human poliovirus 2
3321.	CAB77538.1	Human poliovirus 2
3322.	CAB77537.1	Human poliovirus 2
3323.	CAB77536.1	Human poliovirus 2
3324.	CAB77535.1	Human poliovirus 2
3325.	CAB77534.1	Human poliovirus 2
3326.	CAB77533.1	Human poliovirus 2
3327.	CAB77532.1	Human poliovirus 2
3328.	CAB77531.1	Human poliovirus 2
3329.	BAK54010.1	Human coxsackievirus A6
3330.	BAK54008.1	Human coxsackievirus A6
3331.	BAK54007.1	Human coxsackievirus A6
3332.	BAK54006.1	Human coxsackievirus A6
3333.	BAK54005.1	Human coxsackievirus A6

3334.	ADU04568.1	Human echovirus 6
3335.	ADU04567.1	Human echovirus 6
3336.	ADU04566.1	Human echovirus 6
3337.	ADU04565.1	Human echovirus 6
3338.	ADU04564.1	Human echovirus 6
3339.	ADU04563.1	Human echovirus 6
3340.	ADU04562.1	Human echovirus 6
3341.	ADU04561.1	Human echovirus 6
3342.	ADU04560.1	Human echovirus 6
3343.	ADU04559.1	Human echovirus 6
3344.	ADU04558.1	Human echovirus 6
3345.	ADU04557.1	Human echovirus 6
3346.	ADU04556.1	Human echovirus 6
3347.	ADU04555.1	Human echovirus 6
3348.	ADU04554.1	Human echovirus 6
3349.	ADU04553.1	Human echovirus 6
3350.	ADU04552.1	Human echovirus 6
3351.	ADU04551.1	Human echovirus 6
3352.	ADU04550.1	Human echovirus 6
3353.	ADU04549.1	Human echovirus 6
3354.	ADU04548.1	Human echovirus 6
3355.	ADU04547.1	Human echovirus 6
3356.	ADU04546.1	Human echovirus 6
3357.	ADU04545.1	Human echovirus 6
3358.	ADU04544.1	Human echovirus 6
3359.	ADU04543.1	Human echovirus 11
3360.	ADU04542.1	Human echovirus 11
3361.	ADU04541.1	Human echovirus 11
3362.	ADU04540.1	Human echovirus 11
3363.	ADU04539.1	Human echovirus 11
3364.	ADU04538.1	Human echovirus 11
3365.	ADU04537.1	Human echovirus 11
3366.	ADU04536.1	Human echovirus 11
3367.	ADU04535.1	Human echovirus 11
3368.	ADU04534.1	Human echovirus 11
3369.	ADU04533.1	Human echovirus 11
3370.	ADU04532.1	Human echovirus 11
3371.	ADU04531.1	Human echovirus 11
3372.	ADU04530.1	Human echovirus 11
3373.	ADU04529.1	Human echovirus 11
3374.	ADU04528.1	Human echovirus 11
3375.	ADU04527.1	Human echovirus 11
3376.	CAB53952.1	Human echovirus 30
3377.	CAB53951.1	Human echovirus 30

3378.	CAB53950.1	Human echovirus 30
3379.	CAB53949.1	Human echovirus 30
3380.	CAB53948.1	Human echovirus 30
3381.	BAL14863.1	Human echovirus 30
3382.	BAL14862.1	Human echovirus 30
3383.	BAL14857.1	Human echovirus 14
3384.	BAL14849.1	Human echovirus 11
3385.	BAL04484.1	Human enterovirus 71
3386.	BAL04483.1	Human enterovirus 71
3387.	BAL04481.1	Human enterovirus 71
3388.	BAL04479.1	Human enterovirus 71
3389.	BAL04476.1	Human enterovirus 71
3390.	BAL04475.1	Human enterovirus 71
3391.	BAL04485.1	Human enterovirus 71
3392.	BAL04482.1	Human enterovirus 71
3393.	BAL04480.1	Human enterovirus 71
3394.	BAL04478.1	Human enterovirus 71
3395.	BAL04477.1	Human enterovirus 71
3396.	BAL04474.1	Human enterovirus 71
3397.	BAL04473.1	Human enterovirus 71
3398.	BAL04472.1	Human enterovirus 71
3399.	BAH24182.1	Human coxsackievirus A4
3400.	BAJ09242.1	Human echovirus 30
3401.	ADE44302.1	Human coxsackievirus A24
3402.	ADE44301.1	Human coxsackievirus A24
3403.	ADO24477.1	Human echovirus 9
3404.	ADO24472.1	Human echovirus 6
3405.	ADV91577.1	Human enterovirus 71
3406.	ADV91576.1	Human enterovirus 71
3407.	ADV91575.1	Human enterovirus 71
3408.	ADN68569.1	Human enterovirus 71
3409.	ADN68568.1	Human enterovirus 71
3410.	ADN68567.1	Human enterovirus 71
3411.	ADN68566.1	Human enterovirus 71
3412.	ADN68565.1	Human enterovirus 71
3413.	ADN68564.1	Human enterovirus 71
3414.	ADN68563.1	Human enterovirus 71
3415.	ADN68562.1	Human enterovirus 71
3416.	ADN68561.1	Human enterovirus 71
3417.	ADN68560.1	Human enterovirus 71
3418.	ADN68559.1	Human enterovirus 71
3419.	ADN68558.1	Human enterovirus 71
3420.	ADN68557.1	Human enterovirus 71
3421.	ADN68556.1	Human enterovirus 71

3422.	ADN68555.1	Human enterovirus 71
3423.	ADN68554.1	Human enterovirus 71
3424.	ADN68553.1	Human enterovirus 71
3425.	ADN68552.1	Human enterovirus 71
3426.	ADN68551.1	Human enterovirus 71
3427.	ADQ38962.1	Human coxsackievirus B1
3428.	ADO85710.1	Human poliovirus 1
3429.	ACV73757.1	Human poliovirus 3
3430.	ACV73756.1	Human coxsackievirus B5
3431.	ACV73755.1	Human coxsackievirus B5
3432.	ACV73754.1	Human coxsackievirus B5
3433.	ACV73753.1	Human coxsackievirus B4
3434.	ACV73752.1	Human coxsackievirus B4
3435.	ACV73751.1	Human coxsackievirus B4
3436.	ACV73750.1	Human coxsackievirus B3
3437.	ACV73749.1	Human coxsackievirus B3
3438.	ACV73748.1	Human coxsackievirus B3
3439.	ACV73747.1	Human coxsackievirus B1
3440.	ACV73746.1	Human coxsackievirus B1
3441.	ACV73745.1	Human coxsackievirus B1
3442.	ACV73744.1	Human echovirus 30
3443.	ACV73743.1	Human echovirus 17
3444.	ACV73741.1	Human echovirus 17
3445.	ACV73740.1	Human echovirus 14
3446.	ACV73739.1	Human echovirus 6
3447.	ACV73738.1	Human echovirus 6
3448.	ACV73737.1	Human echovirus 3
3449.	ACV73736.1	Human echovirus 3
3450.	ADD84742.1	Human coxsackievirus A16
3451.	ADD84741.1	Human coxsackievirus A16
3452.	ADD84740.1	Human coxsackievirus A16
3453.	ADD84739.1	Human coxsackievirus A16
3454.	ADD84738.1	Human coxsackievirus A16
3455.	ADD84737.1	Human coxsackievirus A16
3456.	ADD84736.1	Human coxsackievirus A16
3457.	ADD84735.1	Human coxsackievirus A16
3458.	ADD84734.1	Human coxsackievirus A16
3459.	ADD84733.1	Human coxsackievirus A16
3460.	ADD84732.1	Human coxsackievirus A16
3461.	ADD84731.1	Human coxsackievirus A16
3462.	ACV73792.1	Human coxsackievirus A24
3463.	ACV73791.1	Human echovirus 30
3464.	ACV73790.1	Human echovirus 30
3465.	ACV73789.1	Human echovirus 30

3466.	ACV73788.1	Human echovirus 30
3467.	ACV73787.1	Human echovirus 30
3468.	ACV73786.1	Human echovirus 30
3469.	ACV73785.1	Human echovirus 30
3470.	ACV73784.1	Human echovirus 30
3471.	ACV73783.1	Human echovirus 30
3472.	ACV73782.1	Human echovirus 30
3473.	ACV73781.1	Human echovirus 30
3474.	ACV73780.1	Human echovirus 25
3475.	ACV73779.1	Human echovirus 25
3476.	ACV73778.1	Human echovirus 25
3477.	ACV73777.1	Human echovirus 25
3478.	ACV73776.1	Human echovirus 25
3479.	ACV73775.1	Human echovirus 18
3480.	ACV73774.1	Human echovirus 18
3481.	ACV73773.1	Human echovirus 18
3482.	ACV73772.1	Human echovirus 18
3483.	ACV73771.1	Human echovirus 18
3484.	ACV73770.1	Human echovirus 18
3485.	ACV73769.1	Human echovirus 18
3486.	ACV73768.1	Human echovirus 18
3487.	ACV73767.1	Human echovirus 16
3488.	ACV73766.1	Human echovirus 14
3489.	ACV73765.1	Human echovirus 14
3490.	ACV73764.1	Human echovirus 14
3491.	ACV73763.1	Human echovirus 9
3492.	ACV73762.1	Human echovirus 9
3493.	ACV73761.1	Human echovirus 9
3494.	ACV73760.1	Human echovirus 5
3495.	ACV73759.1	Human echovirus 5
3496.	ACV73758.1	Human echovirus 1
3497.	ABW98564.1	Human enterovirus 71
3498.	ABW98563.1	Human enterovirus 71
3499.	ABW98562.1	Human enterovirus 71
3500.	ABW98561.1	Human enterovirus 71
3501.	ABW98560.1	Human enterovirus 71
3502.	ABW98559.1	Human enterovirus 71
3503.	ABW98558.1	Human enterovirus 71
3504.	ABW98557.1	Human enterovirus 71
3505.	ABW98556.1	Human enterovirus 71
3506.	ABW98555.1	Human enterovirus 71
3507.	ABW98554.1	Human enterovirus 71
3508.	ABW98553.1	Human enterovirus 71
3509.	ABW98552.1	Human enterovirus 71

3510.	ABW98551.1	Human enterovirus 71
3511.	ABW98550.1	Human enterovirus 71
3512.	ABW98549.1	Human enterovirus 71
3513.	ABW98548.1	Human enterovirus 71
3514.	ABW98547.1	Human enterovirus 71
3515.	ABW98546.1	Human enterovirus 71
3516.	ABW98545.1	Human enterovirus 71
3517.	ABW98544.1	Human enterovirus 71
3518.	ABW98543.1	Human enterovirus 71
3519.	ABW98542.1	Human enterovirus 71
3520.	ABW98541.1	Human enterovirus 71
3521.	ABW98540.1	Human enterovirus 71
3522.	ABW98539.1	Human enterovirus 71
3523.	ABW98538.1	Human enterovirus 71
3524.	ABW24066.1	Human poliovirus 1
3525.	ABW24065.1	Human poliovirus 1
3526.	ABW24064.1	Human poliovirus 1
3527.	ABW24063.1	Human poliovirus 1
3528.	ABW24062.1	Human poliovirus 1
3529.	ABW24061.1	Human poliovirus 1
3530.	ABW24060.1	Human poliovirus 1
3531.	ABW24059.1	Human poliovirus 1
3532.	ABW24058.1	Human poliovirus 1
3533.	ABW24057.1	Human poliovirus 1
3534.	ABW24056.1	Human poliovirus 1
3535.	ABW24055.1	Human poliovirus 1
3536.	ABW24054.1	Human poliovirus 1
3537.	ABW24053.1	Human poliovirus 1
3538.	ABW24052.1	Human poliovirus 1
3539.	ABW24051.1	Human poliovirus 1
3540.	ABW24050.1	Human poliovirus 1
3541.	ABW24049.1	Human poliovirus 1
3542.	ABW24048.1	Human poliovirus 1
3543.	ABW24047.1	Human poliovirus 1
3544.	ABW24046.1	Human poliovirus 1
3545.	ABW24045.1	Human poliovirus 1
3546.	ABW24044.1	Human poliovirus 1
3547.	ABW24043.1	Human poliovirus 1
3548.	ABW24042.1	Human poliovirus 1
3549.	ABW24041.1	Human poliovirus 1
3550.	ABW24040.1	Human poliovirus 1
3551.	ABW24039.1	Human poliovirus 1
3552.	ABW24038.1	Human poliovirus 1
3553.	ABV91317.1	Human coxsackievirus A24

3554.	ABV91316.1	Human coxsackievirus A24
3555.	ABV91315.1	Human coxsackievirus A24
3556.	ABV91314.1	Human coxsackievirus A24
3557.	ABV91313.1	Human coxsackievirus A24
3558.	ABV91312.1	Human coxsackievirus A24
3559.	ABV91311.1	Human coxsackievirus A24
3560.	ABV91310.1	Human coxsackievirus A24
3561.	ABV91309.1	Human coxsackievirus A24
3562.	ABV91308.1	Human coxsackievirus A24
3563.	ABV91307.1	Human coxsackievirus A24
3564.	ABR00995.1	Human poliovirus 2
3565.	ABR00994.1	Human poliovirus 2
3566.	ABR00993.1	Human poliovirus 2
3567.	ABR00992.1	Human poliovirus 2
3568.	ABR00991.1	Human poliovirus 2
3569.	ABR00990.1	Human poliovirus 2
3570.	ABR00989.1	Human poliovirus 2
3571.	ABR00988.1	Human poliovirus 2
3572.	ABR00987.1	Human poliovirus 3
3573.	ABR00986.1	Human poliovirus 3
3574.	ABR00985.1	Human poliovirus 3
3575.	ABR00984.1	Human poliovirus 3
3576.	ABR00983.1	Human poliovirus 3
3577.	ABR00982.1	Human poliovirus 3
3578.	ABR00981.1	Human poliovirus 3
3579.	ABR00980.1	Human poliovirus 3
3580.	ABF82335.1	Human coxsackievirus B1
3581.	ABE99819.1	Human enterovirus 75
3582.	ABE99818.1	Human enterovirus 75
3583.	ABE99817.1	Human enterovirus 75
3584.	ABE99816.1	Human enterovirus 75
3585.	ABE99815.1	Human enterovirus 75
3586.	ABE99814.1	Human enterovirus 75
3587.	ABA55824.1	Human echovirus 30
3588.	AAZ29481.1	Human echovirus 30
3589.	AAZ29480.1	Human echovirus 30
3590.	AAZ29479.1	Human echovirus 30
3591.	AAZ29478.1	Human echovirus 30
3592.	AAZ29477.1	Human echovirus 30
3593.	AAZ29476.1	Human echovirus 30
3594.	AAZ29475.1	Human echovirus 30
3595.	AAZ29474.1	Human echovirus 30
3596.	AAZ29473.1	Human echovirus 30
3597.	AAZ29472.1	Human echovirus 30

3598.	AAZ29471.1	Human echovirus 30
3599.	AAZ29470.1	Human echovirus 30
3600.	AAZ29469.1	Human echovirus 30
3601.	AAZ29468.1	Human echovirus 30
3602.	AAZ29467.1	Human echovirus 30
3603.	AAZ29466.1	Human echovirus 30
3604.	AAZ29465.1	Human echovirus 30
3605.	AAZ29464.1	Human echovirus 30
3606.	AAZ29463.1	Human echovirus 30
3607.	AAU07824.1	Human coxsackievirus B5
3608.	AAU07823.1	Human coxsackievirus B5
3609.	AAU07822.1	Human echovirus 30
3610.	AAU07821.1	Human echovirus 30
3611.	AAU07820.1	Human echovirus 30
3612.	AAU07819.1	Human echovirus 30
3613.	AAU07818.1	Human echovirus 30
3614.	AAU07817.1	Human echovirus 30
3615.	AAU07816.1	Human echovirus 30
3616.	AAU07815.1	Human echovirus 30
3617.	AAU07814.1	Human echovirus 30
3618.	AAU07813.1	Human echovirus 30
3619.	CCC55366.1	Human coxsackievirus A10
3620.	CCC55365.1	Human coxsackievirus A10
3621.	CCC55364.1	Human coxsackievirus A10
3622.	CCC55363.1	Human coxsackievirus A10
3623.	CCC55362.1	Human coxsackievirus A10
3624.	CCC55361.1	Human coxsackievirus A10
3625.	CCC55360.1	Human coxsackievirus A10
3626.	CCC55359.1	Human coxsackievirus A10
3627.	CCC55358.1	Human coxsackievirus A10
3628.	CCC55357.1	Human coxsackievirus A10
3629.	CCC55356.1	Human coxsackievirus A10
3630.	CCC55355.1	Human coxsackievirus A10
3631.	CCC55354.1	Human coxsackievirus A10
3632.	CCC55353.1	Human coxsackievirus A10
3633.	CCC55352.1	Human coxsackievirus A10
3634.	CCC55351.1	Human coxsackievirus A10
3635.	CCC55350.1	Human coxsackievirus A10
3636.	CCC55349.1	Human coxsackievirus A10
3637.	CCC55348.1	Human coxsackievirus A10
3638.	CCC55347.1	Human coxsackievirus A10
3639.	CCC55346.1	Human coxsackievirus A10
3640.	CCC55345.1	Human coxsackievirus A10
3641.	CCC55344.1	Human coxsackievirus A10

3642.	CCC55343.1	Human coxsackievirus A10
3643.	CCC55342.1	Human coxsackievirus A10
3644.	CCC55341.1	Human coxsackievirus A10
3645.	AER00261.1	Human echovirus 30
3646.	AER00260.1	Human echovirus 30
3647.	AAL15448.1	Human enterovirus 71
3648.	AEM60329.1	Human echovirus 30
3649.	AEM60328.1	Human echovirus 30
3650.	AEM60327.1	Human echovirus 30
3651.	AEM60326.1	Human echovirus 30
3652.	AEM60325.1	Human echovirus 30
3653.	AEM60324.1	Human echovirus 30
3654.	AEM60323.1	Human echovirus 30
3655.	AEM60322.1	Human echovirus 30
3656.	AEM60321.1	Human echovirus 30
3657.	AEM60320.1	Human echovirus 30
3658.	AEM60319.1	Human echovirus 30
3659.	AEM60318.1	Human echovirus 30
3660.	AEM60317.1	Human echovirus 30
3661.	AEM60316.1	Human echovirus 30
3662.	AEM60315.1	Human echovirus 30
3663.	AEM60314.1	Human echovirus 30
3664.	AEM60313.1	Human echovirus 30
3665.	AEM60312.1	Human echovirus 30
3666.	AEM60311.1	Human echovirus 30
3667.	AEM60310.1	Human echovirus 30
3668.	AEM60309.1	Human echovirus 30
3669.	AEM60308.1	Human echovirus 30
3670.	AEM60307.1	Human echovirus 30
3671.	AEM60306.1	Human echovirus 30
3672.	AEM60305.1	Human echovirus 30
3673.	AEM60304.1	Human echovirus 30
3674.	AEM60303.1	Human echovirus 30
3675.	AEM60302.1	Human echovirus 30
3676.	AEM60301.1	Human echovirus 30
3677.	AEM60300.1	Human echovirus 30
3678.	AEM60299.1	Human echovirus 30
3679.	AEM60298.1	Human echovirus 30
3680.	AEM60297.1	Human echovirus 30
3681.	AEM60296.1	Human echovirus 30
3682.	AEM60295.1	Human echovirus 30
3683.	AEM60294.1	Human echovirus 30
3684.	AEM60293.1	Human echovirus 30
3685.	AEM60292.1	Human echovirus 30

3686.	AEM60291.1	Human echovirus 30
3687.	AEM60290.1	Human echovirus 30
3688.	AEM60289.1	Human echovirus 30
3689.	AEM60288.1	Human echovirus 30
3690.	AEM60287.1	Human echovirus 30
3691.	AEM60286.1	Human echovirus 30
3692.	AEM60285.1	Human echovirus 30
3693.	AEM60284.1	Human echovirus 30
3694.	AEM60283.1	Human echovirus 30
3695.	AEM60282.1	Human echovirus 30
3696.	AEE39302.1	Human enterovirus 71
3697.	AEE39301.1	Human enterovirus 71
3698.	AEE39300.1	Human enterovirus 71
3699.	AEE39299.1	Human enterovirus 71
3700.	AEE39298.1	Human enterovirus 71
3701.	AEP67962.1	Human enterovirus 73
3702.	AEP67961.1	Human coxsackievirus B3
3703.	AEP67960.1	Human coxsackievirus A21
3704.	AEP67959.1	Human coxsackievirus A21
3705.	AEP67958.1	Human coxsackievirus A21
3706.	AEP67957.1	Human echovirus 33
3707.	AEP67956.1	Human echovirus 25
3708.	AEP67955.1	Human echovirus 25
3709.	AEP67954.1	Human echovirus 24
3710.	AEP67953.1	Human echovirus 24
3711.	AEP67952.1	Human echovirus 21
3712.	AEP67951.1	Human echovirus 21
3713.	AEP67950.1	Human echovirus 13
3714.	AEP67949.1	Human echovirus 13
3715.	AEP67948.1	Human echovirus 13
3716.	AEP67947.1	Human echovirus 12
3717.	AEP67946.1	Human echovirus 12
3718.	AEP67945.1	Human echovirus 11
3719.	AEP67944.1	Human echovirus 4
3720.	AEP67943.1	Human echovirus 4
3721.	AEP67942.1	Human echovirus 1
3722.	AEM06541.1	Human poliovirus 1
3723.	AEM06540.1	Human poliovirus 1
3724.	AEM06539.1	Human poliovirus 1
3725.	AEM06538.1	Human poliovirus 1
3726.	AEM06537.1	Human poliovirus 2
3727.	AEM06536.1	Human poliovirus 2
3728.	AEM06535.1	Human poliovirus 3
3729.	AEM06534.1	Human poliovirus 3

3730.	ADH29926.1	Human echovirus 6
3731.	ADH29925.1	Human echovirus 6
3732.	ADH29924.1	Human echovirus 6
3733.	ADH29923.1	Human echovirus 6
3734.	ADH29922.1	Human echovirus 6
3735.	ADH29921.1	Human echovirus 6
3736.	ADH29915.1	Human echovirus 6
3737.	ADH29907.1	Human echovirus 6
3738.	ADH29906.1	Human echovirus 6
3739.	ADH29905.1	Human echovirus 6
3740.	ADV92305.1	Human coxsackievirus A24
3741.	ADV92304.1	Human coxsackievirus A24
3742.	ADV92303.1	Human coxsackievirus A24
3743.	ADV92302.1	Human coxsackievirus A24
3744.	ADV92301.1	Human coxsackievirus A24
3745.	ADV92300.1	Human coxsackievirus A24
3746.	ADV92299.1	Human coxsackievirus A24
3747.	ADV92298.1	Human coxsackievirus A24
3748.	ADV92297.1	Human coxsackievirus A24
3749.	ADV92296.1	Human coxsackievirus A24
3750.	ADV92295.1	Human coxsackievirus A24
3751.	ADV92294.1	Human coxsackievirus A24
3752.	ADY80289.1	Human poliovirus 3 strain Sabin
3753.	ADY80288.1	Human poliovirus 3 strain Sabin
3754.	ADY80287.1	Human poliovirus 3 strain Sabin
3755.	ADY80286.1	Human poliovirus 2 strain Sabin
3756.	ADY80285.1	Human poliovirus 1 strain Sabin
3757.	ADY80284.1	Human poliovirus 1 strain Sabin
3758.	ADY80283.1	Human poliovirus 1 strain Sabin
3759.	ADY80282.1	Human echovirus 13
3760.	ADY80281.1	Human echovirus 13
3761.	ADY80280.1	Human echovirus 13
3762.	ADY80279.1	Human echovirus 13
3763.	ADY80278.1	Human echovirus 13
3764.	ADY80277.1	Human echovirus 12
3765.	ADY80276.1	Human echovirus 12
3766.	ADY80275.1	Human echovirus 11
3767.	ADY80274.1	Human echovirus 7
3768.	ADY80273.1	Human echovirus 7
3769.	ADY80272.1	Human echovirus 7
3770.	ADY80271.1	Human echovirus 7
3771.	ADY80270.1	Human echovirus 7
3772.	ADY80269.1	Human echovirus 7
3773.	ADY80268.1	Human echovirus 7

3774.	ADY80267.1	Human echovirus 7
3775.	ADY80266.1	Human echovirus 7
3776.	ADY80265.1	Human echovirus 7
3777.	ADY80264.1	Human echovirus 7
3778.	ADY80263.1	Human echovirus 7
3779.	ADY80262.1	Human echovirus 7
3780.	ADY80261.1	Human coxsackievirus B3
3781.	BAJ76897.1	Human enterovirus 68
3782.	BAJ76896.1	Human enterovirus 68
3783.	BAJ76895.1	Human enterovirus 68
3784.	BAJ76894.1	Human enterovirus 68
3785.	BAJ76893.1	Human enterovirus 68
3786.	BAJ76892.1	Human enterovirus 68
3787.	BAJ76891.1	Human enterovirus 68
3788.	BAJ76890.1	Human enterovirus 68
3789.	BAJ76889.1	Human enterovirus 68
3790.	BAJ76888.1	Human enterovirus 68
3791.	BAJ76887.1	Human enterovirus 68
3792.	BAJ76886.1	Human enterovirus 68
3793.	BAJ76885.1	Human enterovirus 68
3794.	BAJ76884.1	Human enterovirus 68
3795.	BAJ76883.1	Human enterovirus 68
3796.	BAJ76882.1	Human enterovirus 68
3797.	BAJ76881.1	Human enterovirus 68
3798.	BAJ76880.1	Human enterovirus 68
3799.	BAJ76879.1	Human enterovirus 68
3800.	BAJ76878.1	Human enterovirus 68
3801.	BAJ76877.1	Human enterovirus 68
3802.	BAJ76876.1	Human enterovirus 68
3803.	BAJ76875.1	Human enterovirus 68
3804.	BAJ76874.1	Human enterovirus 68
3805.	BAJ76873.1	Human enterovirus 68
3806.	BAJ76872.1	Human enterovirus 68
3807.	BAJ76871.1	Human enterovirus 68
3808.	BAJ76870.1	Human enterovirus 68
3809.	BAJ76869.1	Human enterovirus 68
3810.	BAJ76868.1	Human enterovirus 68
3811.	BAJ76867.1	Human enterovirus 68
3812.	BAJ76866.1	Human enterovirus 68
3813.	BAJ76865.1	Human enterovirus 68
3814.	BAJ76864.1	Human enterovirus 68
3815.	BAJ76863.1	Human enterovirus 68
3816.	BAJ76862.1	Human enterovirus 68
3817.	BAJ76861.1	Human enterovirus 68

3818.	BAJ76860.1	Human enterovirus 68
3819.	BAJ76859.1	Human enterovirus 68
3820.	ABB82181.1	Human poliovirus 3
3821.	ABB82180.1	Human poliovirus 3
3822.	ABB82179.1	Human poliovirus 3
3823.	ABB82178.1	Human poliovirus 3
3824.	ABB82177.1	Human poliovirus 3
3825.	ABB82176.1	Human poliovirus 3
3826.	ABB82175.1	Human poliovirus 3
3827.	ABB82174.1	Human poliovirus 3
3828.	ABB82173.1	Human poliovirus 3
3829.	ABB82172.1	Human poliovirus 3
3830.	ABB82171.1	Human poliovirus 3
3831.	ABB82170.1	Human poliovirus 3
3832.	ABB82169.1	Human poliovirus 2
3833.	ABB82168.1	Human poliovirus 2
3834.	ABB82167.1	Human poliovirus 2
3835.	ABB82166.1	Human poliovirus 2
3836.	ABB82165.1	Human poliovirus 2
3837.	ABB82164.1	Human poliovirus 2
3838.	ABB82163.1	Human poliovirus 2
3839.	ABB82162.1	Human poliovirus 2
3840.	ABB82161.1	Human poliovirus 2
3841.	ABB82160.1	Human poliovirus 2
3842.	ABB82159.1	Human poliovirus 2
3843.	ABB82158.1	Human poliovirus 2
3844.	ABB82157.1	Human poliovirus 2
3845.	ABB82156.1	Human poliovirus 2
3846.	ABB82155.1	Human poliovirus 2
3847.	ABB82154.1	Human poliovirus 2
3848.	ABB82153.1	Human poliovirus 2
3849.	ABB82152.1	Human poliovirus 2
3850.	ABB82151.1	Human poliovirus 1
3851.	ABB82150.1	Human poliovirus 1
3852.	ABB82149.1	Human poliovirus 1
3853.	ABB82148.1	Human poliovirus 1
3854.	ABB82147.1	Human poliovirus 1
3855.	ABB82146.1	Human poliovirus 1
3856.	ABB82145.1	Human poliovirus 1
3857.	ABB82144.1	Human poliovirus 1
3858.	ABB82143.1	Human poliovirus 1
3859.	ABB82142.1	Human poliovirus 1
3860.	ABB82141.1	Human poliovirus 1
3861.	ABB82140.1	Human poliovirus 1

3862.	ABB82139.1	Human poliovirus 1
3863.	ABB82138.1	Human poliovirus 1
3864.	ABB82137.1	Human poliovirus 1
3865.	ABB82136.1	Human poliovirus 1
3866.	ABB82135.1	Human poliovirus 1
3867.	ABB82134.1	Human poliovirus 1
3868.	ABB82133.1	Human poliovirus 1
3869.	ABB82132.1	Human poliovirus 1
3870.	ABB82131.1	Human poliovirus 1
3871.	ABB82130.1	Human poliovirus 1
3872.	ABB82129.1	Human poliovirus 1
3873.	ABB82128.1	Human poliovirus 1
3874.	ABB82127.1	Human poliovirus 1
3875.	ABB82126.1	Human poliovirus 1
3876.	ABB82125.1	Human poliovirus 1
3877.	ABB82124.1	Human poliovirus 1
3878.	ABB82123.1	Human poliovirus 1
3879.	ABB82122.1	Human poliovirus 1
3880.	ABB82121.1	Human poliovirus 1
3881.	ABB82120.1	Human poliovirus 1
3882.	ABB82119.1	Human poliovirus 1
3883.	ABB82118.1	Human poliovirus 1
3884.	ABB82117.1	Human poliovirus 1
3885.	ABB82116.1	Human poliovirus 1
3886.	ABB82115.1	Human poliovirus 1
3887.	ABB82114.1	Human poliovirus 1
3888.	ABB82113.1	Human poliovirus 1
3889.	ABB82112.1	Human poliovirus 1
3890.	ABB82111.1	Human poliovirus 1
3891.	ACU29636.1	Human coxsackievirus B5
3892.	ACU29635.1	Human coxsackievirus B5
3893.	ACU29634.1	Human coxsackievirus B5
3894.	ACU29633.1	Human coxsackievirus B5
3895.	ACU29632.1	Human coxsackievirus B5
3896.	ACU29631.1	Human coxsackievirus B5
3897.	ACU29630.1	Human coxsackievirus B5
3898.	ACU29629.1	Human coxsackievirus B5
3899.	ACU29628.1	Human coxsackievirus B5
3900.	ACU29627.1	Human coxsackievirus B5
3901.	ACU29626.1	Human coxsackievirus B4
3902.	ACU29625.1	Human coxsackievirus B4
3903.	ACU29624.1	Human coxsackievirus B4
3904.	ACU29623.1	Human coxsackievirus B4
3905.	ACU29622.1	Human coxsackievirus B4

3906.	ACU29620.1	Human echovirus 6
3907.	ACU29619.1	Human echovirus 6
3908.	ACU29618.1	Human echovirus 6
3909.	ACU29617.1	Human echovirus 6
3910.	ACU29616.1	Human echovirus 6
3911.	ACU29615.1	Human echovirus 6
3912.	ACU29614.1	Human echovirus 30
3913.	ACU29613.1	Human echovirus 30
3914.	ACU29612.1	Human echovirus 30
3915.	ACU29611.1	Human echovirus 30
3916.	ACU29610.1	Human echovirus 30
3917.	ACU29609.1	Human echovirus 30
3918.	ACU29608.1	Human echovirus 30
3919.	ACK58699.1	Human enterovirus 71
3920.	ACK58698.1	Human enterovirus 71
3921.	ACK58697.1	Human enterovirus 71
3922.	ACK58696.1	Human enterovirus 71
3923.	ACK58695.1	Human enterovirus 71
3924.	ACK58694.1	Human enterovirus 71
3925.	ACK58693.1	Human enterovirus 71
3926.	ACK58692.1	Human enterovirus 71
3927.	ACK58691.1	Human enterovirus 71
3928.	ACK58690.1	Human enterovirus 71
3929.	AAK91993.1	Human enterovirus 71
3930.	AAK91992.1	Human enterovirus 71
3931.	AAK91991.1	Human enterovirus 71
3932.	AAK91990.1	Human enterovirus 71
3933.	AAK91989.1	Human enterovirus 71
3934.	AAK91988.1	Human enterovirus 71
3935.	AAK91987.1	Human enterovirus 71
3936.	AAK91986.1	Human enterovirus 71
3937.	AAK91985.1	Human enterovirus 71
3938.	AAK91984.1	Human enterovirus 71
3939.	AAK91983.1	Human enterovirus 71
3940.	AAK91982.1	Human enterovirus 71
3941.	AAK91981.1	Human enterovirus 71
3942.	AAK91980.1	Human enterovirus 71
3943.	AAK91979.1	Human enterovirus 71
3944.	AAK91978.1	Human enterovirus 71
3945.	AAK91977.1	Human enterovirus 71
3946.	AAK91976.1	Human enterovirus 71
3947.	AAK91975.1	Human enterovirus 71
3948.	AAK91974.1	Human enterovirus 71
3949.	AAK91973.1	Human enterovirus 71

3950.	AAK91972.1	Human enterovirus 71
3951.	AAK91971.1	Human enterovirus 71
3952.	AAK91970.1	Human enterovirus 71
3953.	AAK91969.1	Human enterovirus 71
3954.	AAK91968.1	Human enterovirus 71
3955.	AAK91967.1	Human enterovirus 71
3956.	AAK91966.1	Human enterovirus 71
3957.	AAK91965.1	Human enterovirus 71
3958.	AAK91964.1	Human enterovirus 71
3959.	AAK91963.1	Human enterovirus 71
3960.	AAK91962.1	Human enterovirus 71
3961.	AAK91961.1	Human enterovirus 71
3962.	AAK91960.1	Human enterovirus 71
3963.	AAK91959.1	Human enterovirus 71
3964.	AAK91958.1	Human enterovirus 71
3965.	AAK91957.1	Human enterovirus 71
3966.	AAK91956.1	Human enterovirus 71
3967.	AAK91955.1	Human enterovirus 71
3968.	AAK91954.1	Human enterovirus 71
3969.	AAK91953.1	Human enterovirus 71
3970.	AAK91952.1	Human enterovirus 71
3971.	AAK91951.1	Human enterovirus 71
3972.	AAK91950.1	Human enterovirus 71
3973.	AAK91949.1	Human enterovirus 71
3974.	AAK91948.1	Human enterovirus 71
3975.	AAK91947.1	Human enterovirus 71
3976.	AAK91946.1	Human enterovirus 71
3977.	AAK91945.1	Human enterovirus 71
3978.	AAK91944.1	Human enterovirus 71
3979.	AAK91943.1	Human enterovirus 71
3980.	AAK91942.1	Human enterovirus 71
3981.	AAK91941.1	Human enterovirus 71
3982.	AAK91940.1	Human enterovirus 71
3983.	AAK91939.1	Human enterovirus 71
3984.	AAK91938.1	Human enterovirus 71
3985.	AAK91937.1	Human enterovirus 71
3986.	AAK91936.1	Human enterovirus 71
3987.	AAK91935.1	Human enterovirus 71
3988.	AAK91934.1	Human enterovirus 71
3989.	AAK91933.1	Human enterovirus 71
3990.	AAK91932.1	Human enterovirus 71
3991.	AAK91931.1	Human enterovirus 71
3992.	AAK91930.1	Human enterovirus 71
3993.	AAK91929.1	Human enterovirus 71

3994.	AAK91928.1	Human enterovirus 71
3995.	ACU29621.1	Human echovirus 6
3996.	CAJ88924.1	Human echovirus 30
3997.	CAJ88921.1	Human echovirus 30
3998.	CAJ88920.1	Human echovirus 30
3999.	CAJ88919.1	Human echovirus 30
4000.	CAJ88918.1	Human echovirus 30
4001.	CAJ88987.1	Human echovirus 30
4002.	CAJ88986.1	Human echovirus 30
4003.	CAJ88985.1	Human echovirus 13
4004.	CAJ88984.1	Human echovirus 30
4005.	CAJ88983.1	Human echovirus 30
4006.	CAJ88982.1	Human echovirus 30
4007.	CAJ88981.1	Human echovirus 18
4008.	CAJ88980.1	Human echovirus 30
4009.	CAJ88979.1	Human echovirus 13
4010.	CAJ88978.1	Human echovirus 30
4011.	CAJ88977.1	Human echovirus 30
4012.	CAJ88976.1	Human echovirus 30
4013.	CAJ88975.1	Human echovirus 18
4014.	CAJ88974.1	Human echovirus 13
4015.	CAJ88973.1	Human echovirus 30
4016.	CAJ88972.1	Human echovirus 13
4017.	CAJ88971.1	Human echovirus 30
4018.	CAJ88970.1	Human echovirus 6
4019.	CAJ88969.1	Human echovirus 18
4020.	CAJ88968.1	Human echovirus 30
4021.	CAJ88967.1	Human echovirus 18
4022.	CAJ88966.1	Human echovirus 6
4023.	CAJ88965.1	Human echovirus 30
4024.	CAJ88964.1	Human coxsackievirus A9
4025.	CAJ88963.1	Human echovirus 30
4026.	CAJ88962.1	Human echovirus 6
4027.	CAJ88961.1	Human echovirus 30
4028.	CAJ88960.1	Human echovirus 30
4029.	CAJ88959.1	Human echovirus 30
4030.	CAJ88958.1	Human echovirus 2
4031.	CAJ88957.1	Human echovirus 30
4032.	CAJ88956.1	Human echovirus 30
4033.	CAJ88955.1	Human echovirus 30
4034.	CAJ88954.1	Human echovirus 18
4035.	CAJ88953.1	Human echovirus 18
4036.	CAJ88952.1	Human echovirus 30
4037.	CAJ88951.1	Human echovirus 30

4038.	CAJ88950.1	Human echovirus 18
4039.	CAJ88949.1	Human echovirus 30
4040.	CAJ88948.1	Human coxsackievirus B5
4041.	CAJ88947.1	Human echovirus 30
4042.	CAJ88946.1	Human echovirus 33
4043.	CAJ88945.1	Human echovirus 30
4044.	CAJ88944.1	Human echovirus 30
4045.	CAJ88943.1	Human echovirus 30
4046.	CAJ88942.1	Human echovirus 13
4047.	CAJ88941.1	Human coxsackievirus B3
4048.	CAJ88940.1	Human coxsackievirus B3
4049.	CAJ88939.1	Human echovirus 30
4050.	CAJ88938.1	Human echovirus 30
4051.	CAJ88937.1	Human echovirus 11
4052.	CAJ88936.1	Human echovirus 30
4053.	CAJ88935.1	Human coxsackievirus B3
4054.	CAJ88934.1	Human echovirus 13
4055.	CAJ88933.1	Human coxsackievirus B3
4056.	CAJ88932.1	Human echovirus 13
4057.	CAJ88931.1	Human coxsackievirus B5
4058.	CAJ88930.1	Human coxsackievirus B3
4059.	CAJ88929.1	Human echovirus 7
4060.	CAJ88928.1	Human echovirus 3
4061.	CAJ88927.1	Human echovirus 3
4062.	CAJ88926.1	Human echovirus 30
4063.	CAJ88925.1	Human coxsackievirus B5
4064.	CAJ88923.1	Human coxsackievirus B5
4065.	CAJ88922.1	Human coxsackievirus B5
4066.	CAJ88917.1	Human coxsackievirus B5
4067.	CAJ88916.1	Human echovirus 4
4068.	CAJ88915.1	Human echovirus 18
4069.	CBX36841.1	Human poliovirus 1
4070.	CBX36838.1	Human poliovirus 84/ROU/2008
4071.	CBX36837.1	Human poliovirus 79/ROU/2008
4072.	CBX36836.1	Human poliovirus 78/ROU/2008
4073.	CBX36835.1	Human poliovirus 3
4074.	CBX36833.1	Human poliovirus 1
4075.	CBX36832.1	Human poliovirus 60/ROU/2008
4076.	CBX36831.1	Human poliovirus 57/ROU/2008
4077.	CBX36829.1	Human poliovirus 30/ROU/2008
4078.	CBX36840.1	Human poliovirus 2
4079.	CBX36839.1	Human poliovirus 1
4080.	CBX36834.1	Human poliovirus 2
4081.	CBX36830.1	Human poliovirus 40/ROU/2008

4082.	CBX36828.1	Human poliovirus 1
4083.	BAJ05485.1	Human enterovirus 71
4084.	BAJ05480.1	Human enterovirus 71
4085.	BAJ05457.1	Human enterovirus 71
4086.	BAJ05453.1	Human enterovirus 71
4087.	BAJ05451.1	Human enterovirus 71
4088.	BAJ05450.1	Human enterovirus 71
4089.	BAJ05446.1	Human enterovirus 71
4090.	BAJ05530.1	Human enterovirus 71
4091.	BAJ05519.1	Human enterovirus 71
4092.	BAJ05418.1	Human enterovirus 71
4093.	BAJ05412.1	Human enterovirus 71
4094.	BAJ05405.1	Human enterovirus 71
4095.	BAJ05488.1	Human enterovirus 71
4096.	BAJ05476.1	Human enterovirus 71
4097.	BAJ05467.1	Human enterovirus 71
4098.	BAJ05463.1	Human enterovirus 71
4099.	BAJ05448.1	Human enterovirus 71
4100.	BAJ05447.1	Human enterovirus 71
4101.	BAJ05443.1	Human enterovirus 71
4102.	BAJ05442.1	Human enterovirus 71
4103.	BAJ05441.1	Human enterovirus 71
4104.	BAJ05440.1	Human enterovirus 71
4105.	BAJ05433.1	Human enterovirus 71
4106.	BAJ05432.1	Human enterovirus 71
4107.	BAJ05422.1	Human enterovirus 71
4108.	BAJ05421.1	Human enterovirus 71
4109.	BAJ05416.1	Human enterovirus 71
4110.	BAK08576.1	Human enterovirus 68
4111.	BAK08575.1	Human enterovirus 68
4112.	BAK08574.1	Human enterovirus 68
4113.	BAK08573.1	Human enterovirus 68
4114.	BAK08572.1	Human enterovirus 68
4115.	BAK08571.1	Human enterovirus 68
4116.	BAK08570.1	Human enterovirus 68
4117.	BAK08569.1	Human enterovirus 68
4118.	BAK08568.1	Human enterovirus 68
4119.	BAK08567.1	Human enterovirus 68
4120.	BAJ05601.1	Human enterovirus 71
4121.	BAJ05588.1	Human enterovirus 71
4122.	BAJ05509.1	Human enterovirus 71
4123.	BAJ05506.1	Human enterovirus 71
4124.	BAJ05493.1	Human enterovirus 71
4125.	BAJ05602.1	Human enterovirus 71

4126.	BAJ05598.1	Human enterovirus 71
4127.	BAJ05597.1	Human enterovirus 71
4128.	BAJ05595.1	Human enterovirus 71
4129.	BAJ05592.1	Human enterovirus 71
4130.	BAJ05591.1	Human enterovirus 71
4131.	BAJ05590.1	Human enterovirus 71
4132.	BAJ05589.1	Human enterovirus 71
4133.	BAJ05587.1	Human enterovirus 71
4134.	BAJ05585.1	Human enterovirus 71
4135.	BAJ05584.1	Human enterovirus 71
4136.	BAJ05582.1	Human enterovirus 71
4137.	BAJ05579.1	Human enterovirus 71
4138.	BAJ05578.1	Human enterovirus 71
4139.	BAJ05577.1	Human enterovirus 71
4140.	BAJ05576.1	Human enterovirus 71
4141.	BAJ05575.1	Human enterovirus 71
4142.	BAJ05572.1	Human enterovirus 71
4143.	BAJ05571.1	Human enterovirus 71
4144.	BAJ05570.1	Human enterovirus 71
4145.	BAJ05569.1	Human enterovirus 71
4146.	BAJ05568.1	Human enterovirus 71
4147.	BAJ05567.1	Human enterovirus 71
4148.	BAJ05566.1	Human enterovirus 71
4149.	BAJ05565.1	Human enterovirus 71
4150.	BAJ05563.1	Human enterovirus 71
4151.	BAJ05562.1	Human enterovirus 71
4152.	BAJ05561.1	Human enterovirus 71
4153.	BAJ05560.1	Human enterovirus 71
4154.	BAJ05558.1	Human enterovirus 71
4155.	BAJ05557.1	Human enterovirus 71
4156.	BAJ05556.1	Human enterovirus 71
4157.	BAJ05555.1	Human enterovirus 71
4158.	BAJ05554.1	Human enterovirus 71
4159.	BAJ05553.1	Human enterovirus 71
4160.	BAJ05552.1	Human enterovirus 71
4161.	BAJ05549.1	Human enterovirus 71
4162.	BAJ05547.1	Human enterovirus 71
4163.	BAJ05544.1	Human enterovirus 71
4164.	BAJ05543.1	Human enterovirus 71
4165.	BAJ05542.1	Human enterovirus 71
4166.	BAJ05522.1	Human enterovirus 71
4167.	BAJ05498.1	Human enterovirus 71
4168.	BAJ05474.1	Human enterovirus 71
4169.	BAJ05516.1	Human enterovirus 71

4170.	BAJ05550.1	Human enterovirus 71
4171.	BAJ05489.1	Human enterovirus 71
4172.	BAJ05487.1	Human enterovirus 71
4173.	BAJ05486.1	Human enterovirus 71
4174.	BAJ05475.1	Human enterovirus 71
4175.	BAJ05473.1	Human enterovirus 71
4176.	BAJ05470.1	Human enterovirus 71
4177.	BAJ05469.1	Human enterovirus 71
4178.	BAJ05466.1	Human enterovirus 71
4179.	BAJ05464.1	Human enterovirus 71
4180.	BAJ05462.1	Human enterovirus 71
4181.	BAJ05461.1	Human enterovirus 71
4182.	BAJ05460.1	Human enterovirus 71
4183.	BAJ05459.1	Human enterovirus 71
4184.	BAJ05455.1	Human enterovirus 71
4185.	BAJ05454.1	Human enterovirus 71
4186.	BAJ05449.1	Human enterovirus 71
4187.	BAJ05429.1	Human enterovirus 71
4188.	CBI83607.1	Human enterovirus 71
4189.	CBI83606.1	Human enterovirus 71
4190.	CBI83605.1	Human enterovirus 71
4191.	CBI83604.1	Human enterovirus 71
4192.	CBI83603.1	Human enterovirus 71
4193.	CBI83602.1	Human enterovirus 71
4194.	CBI83601.1	Human enterovirus 71
4195.	CBI83600.1	Human enterovirus 71
4196.	CBI83599.1	Human enterovirus 71
4197.	CBI83598.1	Human enterovirus 71
4198.	CBI83597.1	Human enterovirus 71
4199.	CBI83596.1	Human enterovirus 71
4200.	CBI83595.1	Human enterovirus 71
4201.	CBI83594.1	Human enterovirus 71
4202.	CBI83593.1	Human enterovirus 71
4203.	CBI83592.1	Human enterovirus 71
4204.	CBI83591.1	Human enterovirus 71
4205.	CBI83590.1	Human enterovirus 71
4206.	CBI83589.1	Human enterovirus 71
4207.	CBI83588.1	Human enterovirus 71
4208.	CBI83587.1	Human enterovirus 71
4209.	CBI83586.1	Human enterovirus 71
4210.	CBI83585.1	Human enterovirus 71
4211.	CBI83584.1	Human enterovirus 71
4212.	CBI83583.1	Human enterovirus 71
4213.	CBI83582.1	Human enterovirus 71

4214.	CBI83581.1	Human enterovirus 71
4215.	CBI63374.1	Human enterovirus 71
4216.	CBI63373.1	Human enterovirus 71
4217.	CBI63372.1	Human enterovirus 71
4218.	CBI63371.1	Human enterovirus 71
4219.	CBI63370.1	Human enterovirus 71
4220.	CBI63369.1	Human enterovirus 71
4221.	CBI63368.1	Human enterovirus 71
4222.	CBI63367.1	Human enterovirus 71
4223.	CBI63366.1	Human enterovirus 71
4224.	CBI63365.1	Human enterovirus 71
4225.	CBI63364.1	Human enterovirus 71
4226.	CBI63363.1	Human enterovirus 71
4227.	CBI63362.1	Human enterovirus 71
4228.	CBI63361.1	Human enterovirus 71
4229.	CBI63360.1	Human enterovirus 71
4230.	CBI63359.1	Human enterovirus 71
4231.	CBI63358.1	Human enterovirus 71
4232.	CBI63357.1	Human enterovirus 71
4233.	CBI63356.1	Human enterovirus 71
4234.	CBI63355.1	Human enterovirus 71
4235.	CBI63354.1	Human enterovirus 71
4236.	CBI63353.1	Human enterovirus 71
4237.	CBI63352.1	Human enterovirus 71
4238.	CBI63351.1	Human enterovirus 71
4239.	CBI63350.1	Human enterovirus 71
4240.	CBI63349.1	Human enterovirus 71
4241.	CBI63348.1	Human enterovirus 71
4242.	CBI63347.1	Human enterovirus 71
4243.	CBI63346.1	Human enterovirus 71
4244.	CBI63345.1	Human enterovirus 71
4245.	CBI63344.1	Human enterovirus 71
4246.	CBI63343.1	Human enterovirus 71
4247.	CBI63342.1	Human enterovirus 71
4248.	CBI63341.1	Human enterovirus 71
4249.	CBI63340.1	Human enterovirus 71
4250.	CBI63339.1	Human enterovirus 71
4251.	CBI63338.1	Human enterovirus 71
4252.	CBI63337.1	Human enterovirus 71
4253.	CBI63336.1	Human enterovirus 71
4254.	CBI63335.1	Human enterovirus 71
4255.	CBI63334.1	Human enterovirus 71
4256.	CBI63333.1	Human enterovirus 71
4257.	CBI63332.1	Human enterovirus 71

4258.	CBI63331.1	Human enterovirus 71
4259.	CBI63330.1	Human enterovirus 71
4260.	CBI63329.1	Human enterovirus 71
4261.	CBI63328.1	Human enterovirus 71
4262.	CBI63327.1	Human enterovirus 71
4263.	CBI63326.1	Human enterovirus 71
4264.	CBI63325.1	Human enterovirus 71
4265.	CBI63324.1	Human enterovirus 71
4266.	CBI63323.1	Human enterovirus 71
4267.	CBI63322.1	Human enterovirus 71
4268.	CBI63321.1	Human enterovirus 71
4269.	CBI63320.1	Human enterovirus 71
4270.	CBI63319.1	Human enterovirus 71
4271.	CBI63318.1	Human enterovirus 71
4272.	CBI63317.1	Human enterovirus 71
4273.	ACR07834.1	Human enterovirus 71
4274.	ACR07833.1	Human enterovirus 71
4275.	ADP00584.1	Human enterovirus 71
4276.	BAJ05539.1	Human enterovirus 71
4277.	BAJ05538.1	Human enterovirus 71
4278.	BAJ05537.1	Human enterovirus 71
4279.	BAJ05536.1	Human enterovirus 71
4280.	BAJ05535.1	Human enterovirus 71
4281.	BAJ05533.1	Human enterovirus 71
4282.	BAJ05531.1	Human enterovirus 71
4283.	BAJ05528.1	Human enterovirus 71
4284.	BAJ05527.1	Human enterovirus 71
4285.	BAJ05526.1	Human enterovirus 71
4286.	BAJ05525.1	Human enterovirus 71
4287.	BAJ05524.1	Human enterovirus 71
4288.	BAJ05521.1	Human enterovirus 71
4289.	BAJ05517.1	Human enterovirus 71
4290.	BAJ05514.1	Human enterovirus 71
4291.	BAJ05513.1	Human enterovirus 71
4292.	BAJ05502.1	Human enterovirus 71
4293.	BAJ05499.1	Human enterovirus 71
4294.	BAJ05495.1	Human enterovirus 71
4295.	BAJ05491.1	Human enterovirus 71
4296.	BAJ05490.1	Human enterovirus 71
4297.	BAJ05603.1	Human enterovirus 71
4298.	BAJ05600.1	Human enterovirus 71
4299.	BAJ05599.1	Human enterovirus 71
4300.	BAJ05596.1	Human enterovirus 71
4301.	BAJ05594.1	Human enterovirus 71

4302.	BAJ05593.1	Human enterovirus 71
4303.	BAJ05586.1	Human enterovirus 71
4304.	BAJ05583.1	Human enterovirus 71
4305.	BAJ05581.1	Human enterovirus 71
4306.	BAJ05580.1	Human enterovirus 71
4307.	BAJ05574.1	Human enterovirus 71
4308.	BAJ05573.1	Human enterovirus 71
4309.	BAJ05564.1	Human enterovirus 71
4310.	BAJ05559.1	Human enterovirus 71
4311.	BAJ05551.1	Human enterovirus 71
4312.	BAJ05548.1	Human enterovirus 71
4313.	BAJ05546.1	Human enterovirus 71
4314.	BAJ05545.1	Human enterovirus 71
4315.	BAJ05541.1	Human enterovirus 71
4316.	BAJ05540.1	Human enterovirus 71
4317.	BAJ05534.1	Human enterovirus 71
4318.	BAJ05532.1	Human enterovirus 71
4319.	BAJ05529.1	Human enterovirus 71
4320.	BAJ05523.1	Human enterovirus 71
4321.	BAJ05520.1	Human enterovirus 71
4322.	BAJ05518.1	Human enterovirus 71
4323.	BAJ05515.1	Human enterovirus 71
4324.	BAJ05512.1	Human enterovirus 71
4325.	BAJ05511.1	Human enterovirus 71
4326.	BAJ05510.1	Human enterovirus 71
4327.	BAJ05508.1	Human enterovirus 71
4328.	BAJ05507.1	Human enterovirus 71
4329.	BAJ05505.1	Human enterovirus 71
4330.	BAJ05504.1	Human enterovirus 71
4331.	BAJ05503.1	Human enterovirus 71
4332.	BAJ05501.1	Human enterovirus 71
4333.	BAJ05500.1	Human enterovirus 71
4334.	BAJ05497.1	Human enterovirus 71
4335.	BAJ05496.1	Human enterovirus 71
4336.	BAJ05494.1	Human enterovirus 71
4337.	BAJ05492.1	Human enterovirus 71
4338.	BAJ05484.1	Human enterovirus 71
4339.	BAJ05483.1	Human enterovirus 71
4340.	BAJ05482.1	Human enterovirus 71
4341.	BAJ05481.1	Human enterovirus 71
4342.	BAJ05479.1	Human enterovirus 71
4343.	BAJ05478.1	Human enterovirus 71
4344.	BAJ05477.1	Human enterovirus 71
4345.	BAJ05472.1	Human enterovirus 71

4346.	BAJ05471.1	Human enterovirus 71
4347.	BAJ05468.1	Human enterovirus 71
4348.	BAJ05465.1	Human enterovirus 71
4349.	BAJ05458.1	Human enterovirus 71
4350.	BAJ05456.1	Human enterovirus 71
4351.	BAJ05452.1	Human enterovirus 71
4352.	BAJ05445.1	Human enterovirus 71
4353.	BAJ05444.1	Human enterovirus 71
4354.	BAJ05439.1	Human enterovirus 71
4355.	BAJ05438.1	Human enterovirus 71
4356.	BAJ05437.1	Human enterovirus 71
4357.	BAJ05436.1	Human enterovirus 71
4358.	BAJ05435.1	Human enterovirus 71
4359.	BAJ05434.1	Human enterovirus 71
4360.	BAJ05431.1	Human enterovirus 71
4361.	BAJ05430.1	Human enterovirus 71
4362.	BAJ05428.1	Human enterovirus 71
4363.	BAJ05427.1	Human enterovirus 71
4364.	BAJ05426.1	Human enterovirus 71
4365.	BAJ05425.1	Human enterovirus 71
4366.	BAJ05424.1	Human enterovirus 71
4367.	BAJ05423.1	Human enterovirus 71
4368.	BAJ05420.1	Human enterovirus 71
4369.	BAJ05419.1	Human enterovirus 71
4370.	BAJ05417.1	Human enterovirus 71
4371.	BAJ05415.1	Human enterovirus 71
4372.	BAJ05414.1	Human enterovirus 71
4373.	BAJ05413.1	Human enterovirus 71
4374.	BAJ05411.1	Human enterovirus 71
4375.	BAJ05410.1	Human enterovirus 71
4376.	BAJ05409.1	Human enterovirus 71
4377.	BAJ05408.1	Human enterovirus 71
4378.	BAJ05407.1	Human enterovirus 71
4379.	BAJ05406.1	Human enterovirus 71
4380.	ABC71496.1	Human coxsackievirus A4
4381.	ABC71495.1	Human coxsackievirus A4
4382.	CAD23448.1	Human echovirus 30
4383.	CAD23447.1	Human echovirus 30
4384.	CAD23446.1	Human echovirus 30
4385.	CAD23445.1	Human echovirus 30
4386.	CAD23444.1	Human echovirus 30
4387.	CAD23443.1	Human echovirus 30
4388.	ADQ54222.1	Human enterovirus 71
4389.	ADQ54221.1	Human enterovirus 71

4390.	ADQ54220.1	Human enterovirus 71
4391.	ADQ54219.1	Human enterovirus 71
4392.	ADQ54218.1	Human enterovirus 71
4393.	YP_001497166.1	Human enterovirus 100
4394.	AEG64735.1	Human echovirus 30
4395.	AEG64734.1	Human echovirus 30
4396.	AEG64733.1	Human echovirus 30
4397.	AEG64732.1	Human echovirus 30
4398.	AEG64731.1	Human echovirus 30
4399.	AEG64730.1	Human echovirus 30
4400.	AEG64729.1	Human echovirus 30
4401.	AEG64728.1	Human echovirus 30
4402.	AEG42526.1	Human enterovirus 80
4403.	AEG42525.1	Human enterovirus 73
4404.	AEG42524.1	Human echovirus 20
4405.	AEG42523.1	Human echovirus 20
4406.	AEG42522.1	Human echovirus 14
4407.	AEG42521.1	Human echovirus 14
4408.	AEG42520.1	Human echovirus 14
4409.	AEG42519.1	Human echovirus 12
4410.	AEG42518.1	Human echovirus 7
4411.	AEG42517.1	Human echovirus 7
4412.	AEG42516.1	Human echovirus 7
4413.	AEG42515.1	Human echovirus 3
4414.	AEB97941.1	Human enterovirus 71
4415.	ADY76973.1	Chimpanzee enterovirus CPS-2011
4416.	ADY76972.1	Chimpanzee enterovirus CPS-2011
4417.	ADY76971.1	Chimpanzee enterovirus CPS-2011
4418.	AEA48880.1	Human coxsackievirus A16
4419.	AEA48879.1	Human coxsackievirus A16
4420.	AEA48878.1	Human coxsackievirus A16
4421.	AEA48877.1	Human coxsackievirus A16
4422.	AEA48876.1	Human coxsackievirus A16
4423.	AEA48875.1	Human coxsackievirus A16
4424.	AEA48874.1	Human coxsackievirus A16
4425.	AEA48873.1	Human coxsackievirus A16
4426.	AEA48872.1	Human coxsackievirus A16
4427.	AEA48871.1	Human coxsackievirus A16
4428.	AEA48870.1	Human coxsackievirus A16
4429.	AEA48869.1	Human coxsackievirus A16
4430.	AEA48868.1	Human coxsackievirus A16
4431.	AEA48867.1	Human coxsackievirus A16
4432.	AEA48866.1	Human coxsackievirus A16
4433.	AEA48865.1	Human coxsackievirus A16

4434.	AEA48864.1	Human coxsackievirus A16
4435.	AEA48863.1	Human coxsackievirus A16
4436.	AEA48862.1	Human coxsackievirus A16
4437.	AEA48861.1	Human coxsackievirus A16
4438.	AEA48860.1	Human coxsackievirus A16
4439.	AEA48859.1	Human coxsackievirus A16
4440.	AEA48858.1	Human coxsackievirus A16
4441.	AEA48857.1	Human coxsackievirus A16
4442.	AEA48856.1	Human coxsackievirus A16
4443.	AEA48855.1	Human coxsackievirus A16
4444.	AEA48854.1	Human coxsackievirus A16
4445.	AEA48853.1	Human coxsackievirus A16
4446.	AEA48852.1	Human coxsackievirus A16
4447.	AEA48851.1	Human coxsackievirus A16
4448.	AEA48850.1	Human coxsackievirus A16
4449.	AEA48849.1	Human coxsackievirus A16
4450.	AEA48848.1	Human coxsackievirus A16
4451.	AEA48847.1	Human coxsackievirus A16
4452.	AEA48846.1	Human coxsackievirus A16
4453.	AEA48845.1	Human coxsackievirus A16
4454.	AEA48844.1	Human coxsackievirus A16
4455.	AEA48843.1	Human coxsackievirus A16
4456.	AEA48842.1	Human coxsackievirus A16
4457.	AEA48841.1	Human coxsackievirus A16
4458.	AEA48840.1	Human coxsackievirus A16
4459.	AEA48839.1	Human coxsackievirus A16
4460.	AEA48838.1	Human coxsackievirus A16
4461.	AEA48837.1	Human coxsackievirus A16
4462.	AEA48836.1	Human coxsackievirus A16
4463.	AEA48835.1	Human coxsackievirus A16
4464.	AEA48834.1	Human coxsackievirus A16
4465.	AEA48833.1	Human coxsackievirus A16
4466.	AEA48832.1	Human coxsackievirus A16
4467.	AEA48831.1	Human coxsackievirus A16
4468.	AEA48830.1	Human coxsackievirus A16
4469.	AEA48829.1	Human coxsackievirus A16
4470.	AEA48828.1	Human coxsackievirus A16
4471.	AEA48827.1	Human coxsackievirus A16
4472.	AEA48826.1	Human coxsackievirus A16
4473.	AEA48825.1	Human coxsackievirus A16
4474.	AEA48824.1	Human coxsackievirus A16
4475.	AEA48823.1	Human coxsackievirus A16
4476.	AEA48822.1	Human coxsackievirus A16
4477.	AEA48821.1	Human coxsackievirus A16

4478.	AEA48820.1	Human coxsackievirus A16
4479.	AEA48819.1	Human coxsackievirus A16
4480.	AEA48818.1	Human coxsackievirus A16
4481.	AEA48817.1	Human coxsackievirus A16
4482.	AEA48816.1	Human coxsackievirus A16
4483.	AEA48815.1	Human coxsackievirus A16
4484.	AEA48814.1	Human coxsackievirus A16
4485.	AEA48813.1	Human coxsackievirus A16
4486.	AEA48812.1	Human coxsackievirus A16
4487.	ADY80315.1	Human enterovirus 83
4488.	ADY80314.1	Human echovirus 24
4489.	ADY80313.1	Human echovirus 24
4490.	ADY80312.1	Human echovirus 24
4491.	ADY80311.1	Human echovirus 24
4492.	ADY80309.1	Human echovirus 14
4493.	ADY80308.1	Human echovirus 14
4494.	ADY80307.1	Human echovirus 13
4495.	ADY80305.1	Human echovirus 13
4496.	ADY80304.1	Human echovirus 13
4497.	ADY80303.1	Human echovirus 12
4498.	ADY80302.1	Human echovirus 12
4499.	ADY80301.1	Human echovirus 11
4500.	ADY80300.1	Human echovirus 11
4501.	ADY80299.1	Human echovirus 11
4502.	ADY80298.1	Human echovirus 11
4503.	ADY80297.1	Human echovirus 6
4504.	ADY80296.1	Human coxsackievirus B3
4505.	ADY80295.1	Human coxsackievirus B1
4506.	ADY80294.1	Human coxsackievirus B1
4507.	ADY80293.1	Human coxsackievirus A20
4508.	ADY80292.1	Human coxsackievirus A10
4509.	ADY80291.1	Human coxsackievirus A8
4510.	ADY80290.1	Human coxsackievirus A8
4511.	ACD76110.1	Human enterovirus 71
4512.	ACD76109.1	Human enterovirus 71
4513.	ACD76108.1	Human enterovirus 71
4514.	ACH47021.1	Human enterovirus 71
4515.	ACE79397.1	Human enterovirus 71
4516.	ACE79396.1	Human enterovirus 71
4517.	ACE79395.1	Human enterovirus 71
4518.	ACE79394.1	Human enterovirus 71
4519.	ACE79393.1	Human enterovirus 71
4520.	ACE79392.1	Human enterovirus 71
4521.	ACE79391.1	Human enterovirus 71

4522.	ACE79390.1	Human enterovirus 71
4523.	ACE79389.1	Human enterovirus 71
4524.	AAY21642.1	Human enterovirus 71
4525.	AAY21641.1	Human enterovirus 71
4526.	AAY21640.1	Human enterovirus 71
4527.	AAY21639.1	Human enterovirus 71
4528.	AAY21638.1	Human enterovirus 71
4529.	AAY21637.1	Human enterovirus 71
4530.	AAY21636.1	Human enterovirus 71
4531.	AAY21635.1	Human enterovirus 71
4532.	AAY21634.1	Human enterovirus 71
4533.	AAY21633.1	Human enterovirus 71
4534.	AAY21632.1	Human enterovirus 71
4535.	AAY21631.1	Human enterovirus 71
4536.	AAY21630.1	Human enterovirus 71
4537.	AAY21629.1	Human enterovirus 71
4538.	AAF44103.1	Human coxsackievirus B6
4539.	AAF44102.1	Human coxsackievirus B6
4540.	AAF44101.1	Human coxsackievirus B2
4541.	AAP55268.1	Human poliovirus 1
4542.	AAP55267.1	Human poliovirus 1
4543.	AAP55266.1	Human poliovirus 1
4544.	AAP55265.1	Human poliovirus 1
4545.	AAP55264.1	Human poliovirus 1
4546.	AAP55263.1	Human poliovirus 1
4547.	AAP55262.1	Human poliovirus 1
4548.	AAP55261.1	Human poliovirus 1
4549.	AAP55260.1	Human poliovirus 1
4550.	AAP55259.1	Human poliovirus 1
4551.	AAP55258.1	Human poliovirus 1
4552.	AAQ09089.1	Human poliovirus 1
4553.	AAQ09088.1	Human poliovirus 1
4554.	AAQ09087.1	Human poliovirus 1
4555.	AAQ09086.1	Human poliovirus 1
4556.	AAQ09085.1	Human poliovirus 1
4557.	AAQ09084.1	Human poliovirus 1
4558.	AAQ09083.1	Human poliovirus 1
4559.	AAQ09082.1	Human poliovirus 1
4560.	AAQ09081.1	Human poliovirus 1
4561.	AAQ09080.1	Human poliovirus 1
4562.	AAQ09079.1	Human poliovirus 1
4563.	AAQ09078.1	Human poliovirus 1
4564.	AAQ09077.1	Human poliovirus 1
4565.	AAQ09076.1	Human poliovirus 1

4566.	AAQ09075.1	Human poliovirus 1
4567.	AAQ09074.1	Human poliovirus 1
4568.	AAQ09073.1	Human poliovirus 1
4569.	AAQ09072.1	Human poliovirus 1
4570.	AAQ09071.1	Human poliovirus 1
4571.	AAQ09070.1	Human poliovirus 1
4572.	AAQ09069.1	Human poliovirus 1
4573.	AAQ09068.1	Human poliovirus 1
4574.	AAQ09067.1	Human poliovirus 1
4575.	AAQ09066.1	Human poliovirus 1
4576.	AAQ09065.1	Human poliovirus 1
4577.	AAQ09064.1	Human poliovirus 1
4578.	AAQ09063.1	Human poliovirus 1
4579.	AAQ09062.1	Human poliovirus 1
4580.	AAQ09061.1	Human poliovirus 1
4581.	AAQ09060.1	Human poliovirus 1
4582.	AAQ09059.1	Human poliovirus 1
4583.	AAQ09058.1	Human poliovirus 1
4584.	AAQ09057.1	Human poliovirus 1
4585.	AAQ09056.1	Human poliovirus 1
4586.	AAQ09055.1	Human poliovirus 1
4587.	AAQ09054.1	Human poliovirus 1
4588.	AAQ09053.1	Human poliovirus 1
4589.	AAQ09052.1	Human poliovirus 1
4590.	AAQ09051.1	Human poliovirus 1
4591.	AAQ09050.1	Human poliovirus 1
4592.	AAQ09049.1	Human poliovirus 1
4593.	AAQ09048.1	Human poliovirus 1
4594.	AAQ09047.1	Human poliovirus 1
4595.	AAQ09046.1	Human poliovirus 1
4596.	AAQ09045.1	Human poliovirus 1
4597.	AAQ09044.1	Human poliovirus 1
4598.	AAQ09043.1	Human poliovirus 1
4599.	AAQ09042.1	Human poliovirus 1
4600.	AAQ09041.1	Human poliovirus 1
4601.	AAQ09040.1	Human poliovirus 1
4602.	AAQ09039.1	Human poliovirus 1
4603.	AAQ09038.1	Human poliovirus 1
4604.	AAQ09037.1	Human poliovirus 1
4605.	AAQ09036.1	Human poliovirus 1
4606.	ACV74268.1	Human coxsackievirus A24
4607.	ACV74267.1	Human coxsackievirus A24
4608.	ACV74266.1	Human coxsackievirus A24
4609.	ACV74260.1	Human coxsackievirus A24

4610.	ACV74259.1	Human coxsackievirus A24
4611.	AAL17766.1	Human poliovirus 1
4612.	BAI39635.1	Human enterovirus 71
4613.	BAI39634.1	Human enterovirus 71
4614.	BAI39633.1	Human enterovirus 71
4615.	BAI39632.1	Human enterovirus 71
4616.	BAI39631.1	Human enterovirus 71
4617.	BAI39630.1	Human enterovirus 71
4618.	BAI39629.1	Human enterovirus 71
4619.	BAI39628.1	Human enterovirus 71
4620.	BAI39627.1	Human enterovirus 71
4621.	BAH78577.1	Human poliovirus 2
4622.	BAH78576.1	Human poliovirus 2
4623.	BAH78575.1	Human poliovirus 2
4624.	BAH78574.1	Human poliovirus 2
4625.	BAH78573.1	Human poliovirus 2
4626.	BAH78572.1	Human poliovirus 2
4627.	BAH78571.1	Human poliovirus 2
4628.	BAH78570.1	Human poliovirus 2
4629.	BAH78569.1	Human poliovirus 2
4630.	BAH78568.1	Human poliovirus 2
4631.	BAH78567.1	Human poliovirus 2
4632.	BAH78566.1	Human poliovirus 2
4633.	BAH78565.1	Human poliovirus 2
4634.	BAH78564.1	Human poliovirus 2
4635.	BAH78563.1	Human poliovirus 2
4636.	BAH78562.1	Human poliovirus 2
4637.	BAH78561.1	Human poliovirus 2
4638.	BAH78560.1	Human poliovirus 2
4639.	BAH78559.1	Human poliovirus 2
4640.	BAH78558.1	Human poliovirus 2
4641.	BAH78557.1	Human poliovirus 2
4642.	BAH78556.1	Human poliovirus 2
4643.	BAH78555.1	Human poliovirus 2
4644.	BAH78554.1	Human poliovirus 2
4645.	BAH78553.1	Human poliovirus 2
4646.	BAH78552.1	Human poliovirus 2
4647.	BAH78551.1	Human poliovirus 2
4648.	BAH78550.1	Human poliovirus 2
4649.	BAH78549.1	Human poliovirus 2
4650.	BAH78548.1	Human poliovirus 2
4651.	BAH78547.1	Human poliovirus 2
4652.	BAH78546.1	Human poliovirus 2
4653.	BAH78545.1	Human poliovirus 2

4654.	BAH78544.1	Human poliovirus 2
4655.	BAH78543.1	Human poliovirus 2
4656.	BAH78542.1	Human poliovirus 2
4657.	BAH78541.1	Human poliovirus 2
4658.	BAH78540.1	Human poliovirus 2
4659.	BAH78539.1	Human poliovirus 2
4660.	BAH78538.1	Human poliovirus 2
4661.	BAH78537.1	Human poliovirus 2
4662.	BAH78536.1	Human poliovirus 2
4663.	BAH78535.1	Human poliovirus 2
4664.	BAH78534.1	Human poliovirus 2
4665.	BAH78533.1	Human poliovirus 2
4666.	BAH78532.1	Human poliovirus 2
4667.	BAH78531.1	Human poliovirus 2
4668.	BAH78525.1	Human poliovirus 1
4669.	BAH78524.1	Human poliovirus 1
4670.	BAH78523.1	Human poliovirus 1
4671.	BAH78522.1	Human poliovirus 1
4672.	BAH78520.1	Human poliovirus 1
4673.	BAH78518.1	Human poliovirus 1
4674.	BAH78517.1	Human poliovirus 1
4675.	BAH78516.1	Human poliovirus 1
4676.	BAH78515.1	Human poliovirus 1
4677.	BAH78514.1	Human poliovirus 1
4678.	BAH78513.1	Human poliovirus 1
4679.	BAH78511.1	Human poliovirus 1
4680.	BAH78509.1	Human poliovirus 1
4681.	BAH78508.1	Human poliovirus 1
4682.	BAH78507.1	Human poliovirus 1
4683.	BAH78506.1	Human poliovirus 1
4684.	BAH78505.1	Human poliovirus 1
4685.	BAH78504.1	Human poliovirus 1
4686.	BAH78503.1	Human poliovirus 1
4687.	BAH78502.1	Human poliovirus 1
4688.	BAH78501.1	Human poliovirus 1
4689.	BAH78500.1	Human poliovirus 1
4690.	BAH78499.1	Human poliovirus 1
4691.	BAH78498.1	Human poliovirus 1
4692.	BAH78497.1	Human poliovirus 1
4693.	BAH78496.1	Human poliovirus 1
4694.	BAH78495.1	Human poliovirus 1
4695.	BAH78494.1	Human poliovirus 1
4696.	BAH78493.1	Human poliovirus 1
4697.	BAH78492.1	Human poliovirus 1

4698.	BAH78491.1	Human poliovirus 1
4699.	BAH78490.1	Human poliovirus 1
4700.	BAH78489.1	Human poliovirus 1
4701.	BAH78488.1	Human poliovirus 1
4702.	BAH78487.1	Human poliovirus 1
4703.	BAH78486.1	Human poliovirus 1
4704.	BAH78485.1	Human poliovirus 1
4705.	BAH78484.1	Human poliovirus 1
4706.	BAH78483.1	Human poliovirus 1
4707.	BAH78482.1	Human poliovirus 1
4708.	BAH78481.1	Human poliovirus 1
4709.	BAH78480.1	Human poliovirus 1
4710.	BAH78479.1	Human poliovirus 1
4711.	BAH78478.1	Human poliovirus 1
4712.	BAH78477.1	Human poliovirus 1
4713.	BAH78476.1	Human poliovirus 1
4714.	BAH78475.1	Human poliovirus 1
4715.	BAH78474.1	Human poliovirus 1
4716.	BAH78472.1	Human poliovirus 1
4717.	BAH78469.1	Human poliovirus 1
4718.	BAH78468.1	Human poliovirus 1
4719.	BAH78467.1	Human poliovirus 1
4720.	BAH78465.1	Human poliovirus 1
4721.	BAH78463.1	Human poliovirus 1
4722.	BAH78462.1	Human poliovirus 1
4723.	BAH78461.1	Human poliovirus 1
4724.	BAH78460.1	Human poliovirus 1
4725.	BAH78459.1	Human poliovirus 1
4726.	BAH78458.1	Human poliovirus 1
4727.	BAH78457.1	Human poliovirus 1
4728.	BAH78456.1	Human poliovirus 1
4729.	BAH78455.1	Human poliovirus 1
4730.	BAH78453.1	Human poliovirus 1
4731.	BAH78451.1	Human poliovirus 1
4732.	BAH78449.1	Human poliovirus 1
4733.	BAH78448.1	Human poliovirus 1
4734.	BAH78447.1	Human poliovirus 1
4735.	BAH78446.1	Human poliovirus 1
4736.	BAH78443.1	Human poliovirus 1
4737.	BAH78442.1	Human poliovirus 1
4738.	BAH78440.1	Human poliovirus 1
4739.	BAH78438.1	Human poliovirus 1
4740.	BAH78437.1	Human poliovirus 1
4741.	BAH78432.1	Human poliovirus 1

4742.	BAH78431.1	Human poliovirus 1
4743.	BAH78430.1	Human poliovirus 1
4744.	BAH78428.1	Human poliovirus 1
4745.	BAH78427.1	Human poliovirus 1
4746.	BAH78426.1	Human poliovirus 1
4747.	BAH78425.1	Human poliovirus 1
4748.	BAH78423.1	Human poliovirus 1
4749.	BAH78422.1	Human poliovirus 1
4750.	BAH78421.1	Human poliovirus 1
4751.	BAH78420.1	Human poliovirus 1
4752.	BAH78416.1	Human poliovirus 1
4753.	BAH78415.1	Human poliovirus 1
4754.	BAH78414.1	Human poliovirus 1
4755.	BAH78411.1	Human poliovirus 1
4756.	BAH78410.1	Human poliovirus 1
4757.	BAH78409.1	Human poliovirus 1
4758.	BAH78407.1	Human poliovirus 1
4759.	BAH78405.1	Human poliovirus 1
4760.	BAH78402.1	Human poliovirus 1
4761.	BAH78401.1	Human poliovirus 1
4762.	BAH78400.1	Human poliovirus 1
4763.	BAH78395.1	Human poliovirus 1
4764.	BAH78394.1	Human poliovirus 1
4765.	BAH78392.1	Human poliovirus 1
4766.	BAH78391.1	Human poliovirus 1
4767.	BAH78390.1	Human poliovirus 1
4768.	BAH78389.1	Human poliovirus 1
4769.	BAH78387.1	Human poliovirus 1
4770.	BAH78384.1	Human poliovirus 1
4771.	BAH78383.1	Human poliovirus 1
4772.	BAH78382.1	Human poliovirus 1
4773.	BAH78380.1	Human poliovirus 1
4774.	BAH78379.1	Human poliovirus 1
4775.	BAH78378.1	Human poliovirus 1
4776.	BAH78377.1	Human poliovirus 1
4777.	BAH78374.1	Human poliovirus 1
4778.	BAH78373.1	Human poliovirus 1
4779.	BAH78372.1	Human poliovirus 1
4780.	BAH78371.1	Human poliovirus 1
4781.	BAH78365.1	Human poliovirus 1
4782.	BAH78359.1	Human poliovirus 1
4783.	BAH78358.1	Human poliovirus 1
4784.	BAH78354.1	Human poliovirus 1
4785.	BAH78351.1	Human poliovirus 1

4786.	BAH78348.1	Human poliovirus 1
4787.	BAH78347.1	Human poliovirus 1
4788.	BAH78346.1	Human poliovirus 1
4789.	BAH78345.1	Human poliovirus 1
4790.	BAH78344.1	Human poliovirus 1
4791.	BAH78343.1	Human poliovirus 1
4792.	BAH78342.1	Human poliovirus 1
4793.	BAH78341.1	Human poliovirus 1
4794.	BAH78340.1	Human poliovirus 1
4795.	BAH78339.1	Human poliovirus 1
4796.	BAH78337.1	Human poliovirus 1
4797.	BAH78336.1	Human poliovirus 1
4798.	BAH78335.1	Human poliovirus 1
4799.	BAH78334.1	Human poliovirus 1
4800.	BAH78333.1	Human poliovirus 1
4801.	BAH78332.1	Human poliovirus 1
4802.	BAH78331.1	Human poliovirus 1
4803.	BAH78330.1	Human poliovirus 1
4804.	BAH78329.1	Human poliovirus 1
4805.	BAH78328.1	Human poliovirus 1
4806.	BAH78327.1	Human poliovirus 1
4807.	BAH78326.1	Human poliovirus 1
4808.	BAH78324.1	Human poliovirus 1
4809.	BAH78323.1	Human poliovirus 1
4810.	BAH78322.1	Human poliovirus 1
4811.	BAH78321.1	Human poliovirus 1
4812.	BAH78317.1	Human poliovirus 1
4813.	BAH78316.1	Human poliovirus 1
4814.	BAH78314.1	Human poliovirus 1
4815.	BAH78313.1	Human poliovirus 1
4816.	BAH78312.1	Human poliovirus 1
4817.	BAH78310.1	Human poliovirus 1
4818.	BAH78306.1	Human poliovirus 1
4819.	BAH78305.1	Human poliovirus 1
4820.	BAH78304.1	Human poliovirus 1
4821.	BAH78297.1	Human poliovirus 1
4822.	BAH78296.1	Human poliovirus 1
4823.	BAH78292.1	Human poliovirus 1
4824.	BAH78291.1	Human poliovirus 1
4825.	BAH78290.1	Human poliovirus 1
4826.	BAH78289.1	Human poliovirus 1
4827.	BAH78285.1	Human poliovirus 1
4828.	BAH78282.1	Human poliovirus 1
4829.	BAH78281.1	Human poliovirus 1

4830.	BAH78277.1	Human poliovirus 1
4831.	BAH78276.1	Human poliovirus 1
4832.	BAH78274.1	Human poliovirus 1
4833.	BAH78273.1	Human poliovirus 1
4834.	BAH78272.1	Human poliovirus 1
4835.	BAH78268.1	Human poliovirus 1
4836.	BAH78267.1	Human poliovirus 1
4837.	BAH78266.1	Human poliovirus 1
4838.	BAH78264.1	Human poliovirus 1
4839.	BAH78263.1	Human poliovirus 1
4840.	BAH78260.1	Human poliovirus 1
4841.	BAH78255.1	Human poliovirus 1
4842.	BAH78253.1	Human poliovirus 1
4843.	BAH78252.1	Human poliovirus 1
4844.	BAH78251.1	Human poliovirus 1
4845.	BAH78249.1	Human poliovirus 1
4846.	BAH78248.1	Human poliovirus 1
4847.	BAH78247.1	Human poliovirus 1
4848.	BAH78244.1	Human poliovirus 1
4849.	BAH78242.1	Human poliovirus 1
4850.	BAH78241.1	Human poliovirus 1
4851.	BAH78239.1	Human poliovirus 1
4852.	BAH78238.1	Human poliovirus 1
4853.	BAH78236.1	Human poliovirus 1
4854.	BAH78234.1	Human poliovirus 1
4855.	BAH78233.1	Human poliovirus 1
4856.	BAH78232.1	Human poliovirus 1
4857.	BAH78231.1	Human poliovirus 1
4858.	BAH78230.1	Human poliovirus 1
4859.	BAH78228.1	Human poliovirus 1
4860.	BAH78227.1	Human poliovirus 1
4861.	BAH78226.1	Human poliovirus 1
4862.	BAH78225.1	Human poliovirus 1
4863.	BAH78224.1	Human poliovirus 1
4864.	BAH78223.1	Human poliovirus 1
4865.	BAH78222.1	Human poliovirus 1
4866.	BAH78221.1	Human poliovirus 1
4867.	BAH78220.1	Human poliovirus 1
4868.	BAH78216.1	Human poliovirus 1
4869.	BAH78215.1	Human poliovirus 1
4870.	BAH78210.1	Human poliovirus 1
4871.	BAH78208.1	Human poliovirus 1
4872.	BAH78207.1	Human poliovirus 1
4873.	BAH78206.1	Human poliovirus 1

4874.	BAH78202.1	Human poliovirus 1
4875.	BAH78201.1	Human poliovirus 1
4876.	BAH78200.1	Human poliovirus 1
4877.	BAH78199.1	Human poliovirus 1
4878.	BAH78194.1	Human poliovirus 1
4879.	BAH78193.1	Human poliovirus 1
4880.	BAH78192.1	Human poliovirus 1
4881.	BAH78191.1	Human poliovirus 1
4882.	BAH78190.1	Human poliovirus 1
4883.	BAH78188.1	Human poliovirus 1
4884.	BAH78187.1	Human poliovirus 1
4885.	BAH78186.1	Human poliovirus 1
4886.	BAH78183.1	Human poliovirus 1
4887.	BAH78182.1	Human poliovirus 1
4888.	BAH78181.1	Human poliovirus 1
4889.	BAH78673.1	Human poliovirus 3
4890.	BAH78672.1	Human poliovirus 3
4891.	BAH78671.1	Human poliovirus 3
4892.	BAH78670.1	Human poliovirus 3
4893.	BAH78669.1	Human poliovirus 3
4894.	BAH78668.1	Human poliovirus 3
4895.	BAH78667.1	Human poliovirus 3
4896.	BAH78666.1	Human poliovirus 3
4897.	BAH78665.1	Human poliovirus 3
4898.	BAH78664.1	Human poliovirus 3
4899.	BAH78663.1	Human poliovirus 3
4900.	BAH78662.1	Human poliovirus 3
4901.	BAH78661.1	Human poliovirus 3
4902.	BAH78660.1	Human poliovirus 3
4903.	BAH78659.1	Human poliovirus 3
4904.	BAH78658.1	Human poliovirus 3
4905.	BAH78657.1	Human poliovirus 3
4906.	BAH78656.1	Human poliovirus 3
4907.	BAH78655.1	Human poliovirus 3
4908.	BAH78654.1	Human poliovirus 3
4909.	BAH78653.1	Human poliovirus 3
4910.	BAH78652.1	Human poliovirus 3
4911.	BAH78651.1	Human poliovirus 3
4912.	BAH78650.1	Human poliovirus 3
4913.	BAH78649.1	Human poliovirus 3
4914.	BAH78648.1	Human poliovirus 3
4915.	BAH78647.1	Human poliovirus 3
4916.	BAH78646.1	Human poliovirus 3
4917.	BAH78645.1	Human poliovirus 3

4918.	BAH78644.1	Human poliovirus 3
4919.	BAH78643.1	Human poliovirus 3
4920.	BAH78642.1	Human poliovirus 3
4921.	BAH78641.1	Human poliovirus 3
4922.	BAH78640.1	Human poliovirus 3
4923.	BAH78639.1	Human poliovirus 3
4924.	BAH78638.1	Human poliovirus 3
4925.	BAH78637.1	Human poliovirus 3
4926.	BAH78636.1	Human poliovirus 3
4927.	BAH78635.1	Human poliovirus 3
4928.	BAH78634.1	Human poliovirus 3
4929.	BAH78633.1	Human poliovirus 3
4930.	BAH78632.1	Human poliovirus 3
4931.	BAH78631.1	Human poliovirus 3
4932.	BAH78630.1	Human poliovirus 3
4933.	BAH78629.1	Human poliovirus 3
4934.	BAH78628.1	Human poliovirus 3
4935.	BAH78627.1	Human poliovirus 3
4936.	BAH78626.1	Human poliovirus 3
4937.	BAH78625.1	Human poliovirus 3
4938.	BAH78624.1	Human poliovirus 3
4939.	BAH78623.1	Human poliovirus 3
4940.	BAH78622.1	Human poliovirus 3
4941.	BAH78621.1	Human poliovirus 3
4942.	BAH78620.1	Human poliovirus 2
4943.	BAH78619.1	Human poliovirus 2
4944.	BAH78618.1	Human poliovirus 2
4945.	BAH78617.1	Human poliovirus 2
4946.	BAH78616.1	Human poliovirus 2
4947.	BAH78615.1	Human poliovirus 2
4948.	BAH78614.1	Human poliovirus 2
4949.	BAH78613.1	Human poliovirus 2
4950.	BAH78612.1	Human poliovirus 2
4951.	BAH78611.1	Human poliovirus 2
4952.	BAH78610.1	Human poliovirus 2
4953.	BAH78609.1	Human poliovirus 2
4954.	BAH78608.1	Human poliovirus 2
4955.	BAH78607.1	Human poliovirus 2
4956.	BAH78606.1	Human poliovirus 2
4957.	BAH78605.1	Human poliovirus 2
4958.	BAH78604.1	Human poliovirus 2
4959.	BAH78603.1	Human poliovirus 2
4960.	BAH78602.1	Human poliovirus 2
4961.	BAH78601.1	Human poliovirus 2

4962.	BAH78600.1	Human poliovirus 2
4963.	BAH78599.1	Human poliovirus 2
4964.	BAH78598.1	Human poliovirus 2
4965.	BAH78597.1	Human poliovirus 2
4966.	BAH78596.1	Human poliovirus 2
4967.	BAH78595.1	Human poliovirus 2
4968.	BAH78594.1	Human poliovirus 2
4969.	BAH78593.1	Human poliovirus 2
4970.	BAH78592.1	Human poliovirus 2
4971.	BAH78591.1	Human poliovirus 2
4972.	BAH78590.1	Human poliovirus 2
4973.	BAH78589.1	Human poliovirus 2
4974.	BAH78588.1	Human poliovirus 2
4975.	BAH78587.1	Human poliovirus 2
4976.	BAH78586.1	Human poliovirus 2
4977.	BAH78585.1	Human poliovirus 2
4978.	BAH78584.1	Human poliovirus 2
4979.	BAH78583.1	Human poliovirus 2
4980.	BAH78582.1	Human poliovirus 2
4981.	BAH78581.1	Human poliovirus 2
4982.	BAH78580.1	Human poliovirus 2
4983.	BAH78579.1	Human poliovirus 2
4984.	BAH78578.1	Human poliovirus 2
4985.	ACM47740.1	Human enterovirus 71
4986.	ACM47739.1	Human enterovirus 71
4987.	ACM47738.1	Human enterovirus 71
4988.	NP_740540.1	Human enterovirus B
4989.	ACH47026.1	Human enterovirus 71
4990.	ACH47025.1	Human enterovirus 71
4991.	ACH47024.1	Human enterovirus 71
4992.	ACH47023.1	Human enterovirus 71
4993.	ACH47022.1	Human enterovirus 71
4994.	AAU07812.1	Human echovirus 30
4995.	AAU07811.1	Human echovirus 30
4996.	AAU07810.1	Human echovirus 30
4997.	AAU07809.1	Human echovirus 30
4998.	AAU07808.1	Human echovirus 30
4999.	AAU07807.1	Human echovirus 30
5000.	AAU07806.1	Human echovirus 30
5001.	ACV73734.1	Human echovirus 25
5002.	ACV73733.1	Human poliovirus 3
5003.	ACV73732.1	Human poliovirus 3
5004.	ACV73731.1	Human poliovirus 2
5005.	ACV73730.1	Human echovirus 30

5006.	ACV73729.1	Human echovirus 30
5007.	ACV73728.1	Human echovirus 18
5008.	ACV73727.1	Human echovirus 18
5009.	ACV73726.1	Human echovirus 17
5010.	ACV73725.1	Human echovirus 17
5011.	ACV73724.1	Human echovirus 14
5012.	ACV73723.1	Human echovirus 14
5013.	ACV73722.1	Human echovirus 11
5014.	ACV73721.1	Human echovirus 11
5015.	ACV73720.1	Human echovirus 9
5016.	ACV73719.1	Human echovirus 9
5017.	ACV73718.1	Human echovirus 7
5018.	ACV73717.1	Human echovirus 7
5019.	ACV73716.1	Human echovirus 6
5020.	ACV73715.1	Human echovirus 6
5021.	ACV73714.1	Human echovirus 3
5022.	ACV73713.1	Human echovirus 3
5023.	ACV73712.1	Human coxsackievirus B5
5024.	ACV73711.1	Human coxsackievirus B5
5025.	ACV73710.1	Human coxsackievirus B4
5026.	ACV73709.1	Human coxsackievirus B4
5027.	ACV73708.1	Human coxsackievirus B3
5028.	ACV73707.1	Human coxsackievirus B3
5029.	ACV73706.1	Human coxsackievirus B2
5030.	ACV73705.1	Human coxsackievirus B1
5031.	ACV73704.1	Human coxsackievirus B1
5032.	ACV73703.1	Human coxsackievirus A9
5033.	ACV73702.1	Human enterovirus 71
5034.	ACV73701.1	Human coxsackievirus A16
5035.	ACC55254.1	Human echovirus 9
5036.	ACC55253.1	Human echovirus 9
5037.	ACC55252.1	Human echovirus 9
5038.	ACC55251.1	Human echovirus 9
5039.	ACC55250.1	Human echovirus 9
5040.	ACC55239.1	Human coxsackievirus B5
5041.	ACA35099.1	Human echovirus 30
5042.	ACA35098.1	Human echovirus 30
5043.	ACA35097.1	Human echovirus 30
5044.	ACA35096.1	Human echovirus 30
5045.	ACA35095.1	Human echovirus 30
5046.	ACA35094.1	Human echovirus 30
5047.	ACA35093.1	Human echovirus 30
5048.	ACA35092.1	Human echovirus 30
5049.	ACA35091.1	Human echovirus 30

5050.	ACA35090.1	Human echovirus 30
5051.	ACA35089.1	Human echovirus 30
5052.	ACA35088.1	Human echovirus 30
5053.	ACA35087.1	Human echovirus 30
5054.	ACA35086.1	Human echovirus 30
5055.	ACA35085.1	Human echovirus 30
5056.	ACA35084.1	Human echovirus 30
5057.	ACA35083.1	Human echovirus 30
5058.	ACA35082.1	Human echovirus 30
5059.	ACA35081.1	Human echovirus 30
5060.	ACA35080.1	Human echovirus 30
5061.	ACA35079.1	Human echovirus 30
5062.	ACA35078.1	Human echovirus 30
5063.	ACA35077.1	Human echovirus 30
5064.	AAR20995.1	Human echovirus 30
5065.	AAR20994.1	Human echovirus 30
5066.	AAR20993.1	Human echovirus 30
5067.	AAR20992.1	Human echovirus 30
5068.	AAR20991.1	Human echovirus 30
5069.	AAR20990.1	Human echovirus 30
5070.	AAR20989.1	Human echovirus 30
5071.	AAR20988.1	Human echovirus 30
5072.	AAR20987.1	Human echovirus 30
5073.	AAR20986.1	Human echovirus 30
5074.	AAR20985.1	Human echovirus 30
5075.	AAR20984.1	Human echovirus 30
5076.	AAR20983.1	Human echovirus 30
5077.	AAR20982.1	Human echovirus 30
5078.	AAR20981.1	Human echovirus 30
5079.	AAR20980.1	Human echovirus 30
5080.	AAR20979.1	Human echovirus 30
5081.	AAR20978.1	Human echovirus 30
5082.	AAR20977.1	Human echovirus 30
5083.	AAR20976.1	Human echovirus 30
5084.	AAR20975.1	Human echovirus 30
5085.	AAR20974.1	Human echovirus 30
5086.	AAR20973.1	Human echovirus 30
5087.	AAR20972.1	Human echovirus 30
5088.	AAR20971.1	Human echovirus 30
5089.	AAR20970.1	Human echovirus 30
5090.	AAR20969.1	Human echovirus 30
5091.	AAR20968.1	Human echovirus 30
5092.	AAP56314.1	Human poliovirus 1
5093.	AAP56313.1	Human poliovirus 1

5094.	AAP56312.1	Human poliovirus 1
5095.	AAP56311.1	Human poliovirus 1
5096.	AAP51722.1	Human echovirus 13
5097.	AAP51721.1	Human echovirus 13
5098.	AAP51720.1	Human echovirus 13
5099.	AAP51719.1	Human echovirus 13
5100.	AAP51718.1	Human echovirus 13
5101.	AAP51717.1	Human echovirus 13
5102.	AAP51716.1	Human echovirus 13
5103.	AAP51715.1	Human echovirus 13
5104.	AAP51714.1	Human echovirus 13
5105.	AAP51713.1	Human echovirus 13
5106.	AAP51712.1	Human echovirus 13
5107.	AAP51711.1	Human echovirus 13
5108.	AAP51710.1	Human echovirus 13
5109.	AAP51709.1	Human echovirus 13
5110.	AEQ34961.1	Human enterovirus 71
5111.	AEQ34960.1	Human enterovirus 71
5112.	AEQ34959.1	Human enterovirus 71
5113.	AEQ34958.1	Human enterovirus 71
5114.	AEQ34957.1	Human enterovirus 71
5115.	AEQ34956.1	Human enterovirus 71
5116.	AEQ34955.1	Human enterovirus 71
5117.	AEQ34954.1	Human enterovirus 71
5118.	AEQ34953.1	Human enterovirus 71
5119.	AEQ34952.1	Human enterovirus 71
5120.	AEQ34951.1	Human enterovirus 71
5121.	AEQ34950.1	Human enterovirus 71
5122.	AEQ34949.1	Human enterovirus 71
5123.	AEQ34948.1	Human enterovirus 71
5124.	AEQ34947.1	Human enterovirus 71
5125.	AEQ34946.1	Human enterovirus 71
5126.	AEQ34945.1	Human enterovirus 71
5127.	AEQ34944.1	Human enterovirus 71
5128.	AEQ34943.1	Human enterovirus 71
5129.	AEQ34942.1	Human enterovirus 71
5130.	AEQ34941.1	Human enterovirus 71
5131.	AEQ34940.1	Human enterovirus 71
5132.	AEQ34939.1	Human enterovirus 71
5133.	AEQ34938.1	Human enterovirus 71
5134.	AEQ34937.1	Human enterovirus 71
5135.	ADT71655.1	Human enterovirus 71
5136.	ADT71654.1	Human enterovirus 71
5137.	ADT71653.1	Human enterovirus 71

5138.	ADT71652.1	Human enterovirus 71
5139.	ADT71651.1	Human enterovirus 71
5140.	ADT71650.1	Human enterovirus 71
5141.	ABK88244.1	Human enterovirus 94
5142.	ABK88243.1	Human enterovirus 94
5143.	ABK88242.1	Human enterovirus 94
5144.	AEX07904.1	Human coxsackievirus A16
5145.	AET86868.1	Human enterovirus 71
5146.	AET86867.1	Human enterovirus 71
5147.	AET86866.1	Human enterovirus 71
5148.	AET86865.1	Human enterovirus 71
5149.	AET86864.1	Human enterovirus 71
5150.	AET86863.1	Human enterovirus 71
5151.	AET86862.1	Human enterovirus 71
5152.	AET86861.1	Human enterovirus 71
5153.	ACB73010.1	Human poliovirus 3
5154.	ACB73009.1	Human poliovirus 3
5155.	AEP67986.1	Human enterovirus 71
5156.	AEP67985.1	Human enterovirus 71
5157.	AEP67984.1	Human enterovirus 71
5158.	AEP67983.1	Human enterovirus 71
5159.	AEP67982.1	Human enterovirus 71
5160.	AEP67981.1	Human enterovirus 71
5161.	AEP67980.1	Human enterovirus 71
5162.	AEP67979.1	Human enterovirus 71
5163.	AEP67978.1	Human enterovirus 71
5164.	AEP67977.1	Human enterovirus 71
5165.	AEP67976.1	Human enterovirus 71
5166.	AEP67975.1	Human enterovirus 71
5167.	AEP67974.1	Human enterovirus 71
5168.	AEP67973.1	Human enterovirus 71
5169.	AEP67972.1	Human enterovirus 71
5170.	AEP67971.1	Human enterovirus 71
5171.	AEP67970.1	Human enterovirus 71
5172.	AEP67969.1	Human enterovirus 71
5173.	AEP67968.1	Human enterovirus 71
5174.	AEP67967.1	Human enterovirus 71
5175.	AEP67966.1	Human enterovirus 71
5176.	AEP67965.1	Human enterovirus 71
5177.	AEP67964.1	Human enterovirus 71
5178.	AEP67963.1	Human enterovirus 71
5179.	AEN79447.1	Human enterovirus 71
5180.	AEN79446.1	Human enterovirus 71
5181.	AEN79445.1	Human enterovirus 71

5182.	AEN79444.1	Human enterovirus 71
5183.	AEM76450.1	Human enterovirus 71
5184.	AEM76449.1	Human enterovirus 71
5185.	AEM76448.1	Human enterovirus 71
5186.	AEM76447.1	Human enterovirus 71
5187.	AEM76446.1	Human enterovirus 71
5188.	AEM76445.1	Human enterovirus 71
5189.	AEM76444.1	Human enterovirus 71
5190.	AEM76443.1	Human enterovirus 71
5191.	AEE26190.1	Human echovirus 13
5192.	AEE26189.1	Human coxsackievirus B2
5193.	AEE26188.1	Human coxsackievirus B2
5194.	AEC13060.1	Human coxsackievirus B5
5195.	AEC13059.1	Human coxsackievirus B3
5196.	ACB73007.1	Human poliovirus 2
5197.	ADN06422.1	Human coxsackievirus B5
5198.	ADN06421.1	Human coxsackievirus B5
5199.	ADN06420.1	Human coxsackievirus B5
5200.	ADN06419.1	Human coxsackievirus B5
5201.	ADN06418.1	Human coxsackievirus B5
5202.	ADN06417.1	Human coxsackievirus B5
5203.	ADN06416.1	Human coxsackievirus B5
5204.	ADN06415.1	Human coxsackievirus B5
5205.	ADN06414.1	Human coxsackievirus B5
5206.	ADN06413.1	Human coxsackievirus B5
5207.	ADN06412.1	Human coxsackievirus B5
5208.	ADN06411.1	Human coxsackievirus B5
5209.	ADN06410.1	Human coxsackievirus B5
5210.	ADN06409.1	Human coxsackievirus B5
5211.	ADN06408.1	Human coxsackievirus B5
5212.	ADN06407.1	Human coxsackievirus B5
5213.	ADN06406.1	Human coxsackievirus B5
5214.	ADN06405.1	Human coxsackievirus B5
5215.	ADN06404.1	Human coxsackievirus B5
5216.	ADN06403.1	Human coxsackievirus B5
5217.	ADN06402.1	Human coxsackievirus B5
5218.	ADN06401.1	Human coxsackievirus B5
5219.	ADN06400.1	Human coxsackievirus B5
5220.	ADN06399.1	Human coxsackievirus B5
5221.	ADN06398.1	Human coxsackievirus B5
5222.	ADN06397.1	Human coxsackievirus B5
5223.	ADN06396.1	Human coxsackievirus B5
5224.	ADN06395.1	Human coxsackievirus B5
5225.	ADN06394.1	Human coxsackievirus B5

5226.	ADN06393.1	Human coxsackievirus B5
5227.	ADN06392.1	Human coxsackievirus B5
5228.	ADN06391.1	Human coxsackievirus B5
5229.	ADN06390.1	Human coxsackievirus B5
5230.	AAT81422.1	Human coxsackievirus A9
5231.	AAT81421.1	Human coxsackievirus A9
5232.	ACB73014.1	Human poliovirus 3
5233.	ACB73013.1	Human poliovirus 3
5234.	ACB73012.1	Human poliovirus 3
5235.	AAP82209.1	Human enterovirus 71
5236.	AAP82208.1	Human enterovirus 71
5237.	AAP82207.1	Human enterovirus 71
5238.	AAP82206.1	Human enterovirus 71
5239.	AAP82205.1	Human enterovirus 71
5240.	AAP82204.1	Human enterovirus 71
5241.	AAP82203.1	Human enterovirus 71
5242.	AAP82202.1	Human enterovirus 71
5243.	AAP82201.1	Human enterovirus 71
5244.	AAP82200.1	Human enterovirus 71
5245.	AAP82199.1	Human enterovirus 71
5246.	AAP82198.1	Human enterovirus 71
5247.	AAP82197.1	Human enterovirus 71
5248.	AAP82196.1	Human enterovirus 71
5249.	AAP82195.1	Human enterovirus 71
5250.	AAP82194.1	Human enterovirus 71
5251.	AAP82193.1	Human enterovirus 71
5252.	AAP82192.1	Human enterovirus 71
5253.	AAP82191.1	Human enterovirus 71
5254.	AAP82190.1	Human enterovirus 71
5255.	AAP82189.1	Human enterovirus 71
5256.	AAP82188.1	Human enterovirus 71
5257.	AAP82187.1	Human enterovirus 71
5258.	AAP82186.1	Human enterovirus 71
5259.	AAP82185.1	Human enterovirus 71
5260.	AAP82184.1	Human enterovirus 71
5261.	AAP82183.1	Human enterovirus 71
5262.	AAP82182.1	Human enterovirus 71
5263.	AAO48570.1	Human enterovirus 71
5264.	AAO48569.1	Human enterovirus 71
5265.	AAO48568.1	Human enterovirus 71
5266.	AAO48567.1	Human enterovirus 71
5267.	AAO48566.1	Human enterovirus 71
5268.	AAO48565.1	Human enterovirus 71
5269.	AAO48564.1	Human enterovirus 71

5270.	AAO48563.1	Human enterovirus 71
5271.	AAO48562.1	Human enterovirus 71
5272.	AAO48561.1	Human enterovirus 71
5273.	AAO48560.1	Human enterovirus 71
5274.	AAO48559.1	Human enterovirus 71
5275.	AAO48558.1	Human enterovirus 71
5276.	AAO48557.1	Human enterovirus 71
5277.	AAO48556.1	Human enterovirus 71
5278.	AAO48555.1	Human enterovirus 71
5279.	AAO48554.1	Human enterovirus 71
5280.	AAO48553.1	Human enterovirus 71
5281.	AAO48552.1	Human enterovirus 71
5282.	AAO48551.1	Human enterovirus 71
5283.	AAO48550.1	Human enterovirus 71
5284.	AAO48549.1	Human enterovirus 71
5285.	AAO48548.1	Human enterovirus 71
5286.	AAO48547.1	Human enterovirus 71
5287.	AAO48546.1	Human enterovirus 71
5288.	AAO48545.1	Human enterovirus 71
5289.	AAO48544.1	Human enterovirus 71
5290.	AAO48543.1	Human enterovirus 71
5291.	AAO48542.1	Human enterovirus 71
5292.	AAO48541.1	Human enterovirus 71
5293.	AAO48540.1	Human enterovirus 71
5294.	AAO48539.1	Human enterovirus 71
5295.	AAO48538.1	Human enterovirus 71
5296.	AAO48537.1	Human enterovirus 71
5297.	AAO48536.1	Human enterovirus 71
5298.	AAO48535.1	Human enterovirus 71
5299.	AAO48534.1	Human enterovirus 71
5300.	AAO48533.1	Human enterovirus 71
5301.	AAO48532.1	Human enterovirus 71
5302.	AAO48531.1	Human enterovirus 71
5303.	AAO48530.1	Human enterovirus 71
5304.	AAO48529.1	Human enterovirus 71
5305.	AAO48528.1	Human enterovirus 71
5306.	ADC43796.1	Human echovirus 19
5307.	ADC43795.1	Human echovirus 14
5308.	ADC43794.1	Human echovirus 7
5309.	ADC43793.1	Human echovirus 6
5310.	ADC43792.1	Human echovirus 3
5311.	ADC43791.1	Human coxsackievirus B5
5312.	ADC43790.1	Human coxsackievirus B3
5313.	ADC43789.1	Human coxsackievirus B3

5314.	ADC43788.1	Human coxsackievirus B3
5315.	CAJ98666.1	Porcine enterovirus B
5316.	AAT06852.1	Human poliovirus 1
5317.	AAT06851.1	Human poliovirus 1
5318.	AAT06850.1	Human poliovirus 1
5319.	AAQ11823.1	Human poliovirus 1
5320.	AAQ11822.1	Human poliovirus 1
5321.	AAQ11821.1	Human poliovirus 1
5322.	AAQ11820.1	Human poliovirus 1
5323.	AAQ11819.1	Human poliovirus 1
5324.	AAQ11818.1	Human poliovirus 1
5325.	AAQ11817.1	Human poliovirus 1
5326.	AAQ11816.1	Human poliovirus 1
5327.	AAQ11815.1	Human poliovirus 1
5328.	AAQ11814.1	Human poliovirus 1
5329.	AAQ11813.1	Human poliovirus 1
5330.	AAQ11812.1	Human poliovirus 1
5331.	AAQ11811.1	Human poliovirus 1
5332.	AAQ11810.1	Human poliovirus 1
5333.	AAQ11809.1	Human poliovirus 1
5334.	AAQ11808.1	Human poliovirus 1
5335.	AAQ11807.1	Human poliovirus 1
5336.	AAQ11806.1	Human poliovirus 1
5337.	AAQ11805.1	Human poliovirus 1
5338.	AAQ11804.1	Human poliovirus 1
5339.	AAQ11803.1	Human poliovirus 1
5340.	AAQ11802.1	Human poliovirus 1
5341.	AAQ11801.1	Human poliovirus 1
5342.	AAQ11800.1	Human poliovirus 1
5343.	AAQ11799.1	Human poliovirus 1
5344.	AAQ11798.1	Human poliovirus 1
5345.	AAQ11797.1	Human poliovirus 1
5346.	AAQ11796.1	Human poliovirus 1
5347.	AAQ11795.1	Human poliovirus 1
5348.	AAQ11794.1	Human poliovirus 1
5349.	AAQ11793.1	Human poliovirus 1
5350.	AAQ11792.1	Human poliovirus 1
5351.	AAQ11791.1	Human poliovirus 1
5352.	AAQ11790.1	Human poliovirus 1
5353.	AAQ11789.1	Human poliovirus 1
5354.	AAQ11788.1	Human poliovirus 1
5355.	AAQ11787.1	Human poliovirus 1
5356.	AAQ11786.1	Human poliovirus 1
5357.	AAQ11785.1	Human poliovirus 1

5358.	AAQ11784.1	Human poliovirus 1
5359.	AAQ11783.1	Human poliovirus 1
5360.	AAQ11782.1	Human poliovirus 1
5361.	AAQ11781.1	Human poliovirus 1
5362.	AAQ11780.1	Human poliovirus 1
5363.	AAQ11779.1	Human poliovirus 1
5364.	AAQ11778.1	Human poliovirus 1
5365.	AAQ11777.1	Human poliovirus 1
5366.	AAQ11776.1	Human poliovirus 1
5367.	AAQ11775.1	Human poliovirus 1
5368.	AAQ11774.1	Human poliovirus 1
5369.	AAQ11773.1	Human poliovirus 1
5370.	AAQ11772.1	Human poliovirus 1
5371.	AAQ11771.1	Human poliovirus 1
5372.	AAQ11770.1	Human poliovirus 1
5373.	AAQ11769.1	Human poliovirus 1
5374.	AAQ11768.1	Human poliovirus 1
5375.	AAQ11767.1	Human poliovirus 1
5376.	AAQ11766.1	Human poliovirus 1
5377.	AAQ11765.1	Human poliovirus 1
5378.	AAQ11764.1	Human poliovirus 1
5379.	AAQ11763.1	Human poliovirus 1
5380.	AAQ11762.1	Human poliovirus 1
5381.	AAQ11761.1	Human poliovirus 1
5382.	AAQ11760.1	Human poliovirus 1
5383.	AAQ11759.1	Human poliovirus 1
5384.	AAQ11758.1	Human poliovirus 1
5385.	AAQ11757.1	Human poliovirus 1
5386.	AAQ11756.1	Human poliovirus 1
5387.	AAQ11755.1	Human poliovirus 1
5388.	AAQ11754.1	Human poliovirus 1
5389.	AAQ11753.1	Human poliovirus 1
5390.	AAQ11752.1	Human poliovirus 1
5391.	AAQ11751.1	Human poliovirus 1
5392.	AAQ11750.1	Human poliovirus 1
5393.	AAQ11749.1	Human poliovirus 1
5394.	AAP83958.1	Human poliovirus 1
5395.	AAP83957.1	Human poliovirus 1
5396.	AAP83956.1	Human poliovirus 1
5397.	AAP83955.1	Human poliovirus 1
5398.	AAP83954.1	Human poliovirus 1
5399.	AAU29364.1	Human poliovirus 3
5400.	AAU29363.1	Human poliovirus 1
5401.	AAT06910.1	Human poliovirus 1

5402.	AAT06909.1	Human poliovirus 1
5403.	AAT06908.1	Human poliovirus 1
5404.	AAT06907.1	Human poliovirus 1
5405.	AAT06906.1	Human poliovirus 1
5406.	AAT06905.1	Human poliovirus 1
5407.	AAT06904.1	Human poliovirus 1
5408.	AAT06903.1	Human poliovirus 1
5409.	AAT06902.1	Human poliovirus 1
5410.	AAT06901.1	Human poliovirus 1
5411.	AAT06900.1	Human poliovirus 1
5412.	AAT06899.1	Human poliovirus 1
5413.	AAT06898.1	Human poliovirus 1
5414.	AAT06897.1	Human poliovirus 1
5415.	AAT06896.1	Human poliovirus 1
5416.	AAT06895.1	Human poliovirus 1
5417.	AAT06894.1	Human poliovirus 1
5418.	AAT06893.1	Human poliovirus 1
5419.	AAT06892.1	Human poliovirus 1
5420.	AAT06891.1	Human poliovirus 1
5421.	AAT06890.1	Human poliovirus 1
5422.	AAT06889.1	Human poliovirus 1
5423.	AAT06888.1	Human poliovirus 1
5424.	AAT06887.1	Human poliovirus 1
5425.	AAT06886.1	Human poliovirus 1
5426.	AAT06885.1	Human poliovirus 1
5427.	AAT06884.1	Human poliovirus 1
5428.	AAT06883.1	Human poliovirus 1
5429.	AAT06882.1	Human poliovirus 1
5430.	AAT06881.1	Human poliovirus 1
5431.	AAT06880.1	Human poliovirus 1
5432.	AAT06879.1	Human poliovirus 1
5433.	AAT06878.1	Human poliovirus 1
5434.	AAT06877.1	Human poliovirus 1
5435.	AAT06876.1	Human poliovirus 1
5436.	AAT06875.1	Human poliovirus 1
5437.	AAT06874.1	Human poliovirus 1
5438.	AAT06873.1	Human poliovirus 1
5439.	AAT06872.1	Human poliovirus 1
5440.	AAT06871.1	Human poliovirus 1
5441.	AAT06870.1	Human poliovirus 1
5442.	AAT06869.1	Human poliovirus 1
5443.	AAT06868.1	Human poliovirus 1
5444.	AAT06867.1	Human poliovirus 1
5445.	AAT06866.1	Human poliovirus 1

5446.	AAT06865.1	Human poliovirus 1
5447.	AAT06864.1	Human poliovirus 1
5448.	AAT06863.1	Human poliovirus 1
5449.	AAT06862.1	Human poliovirus 1
5450.	AAT06861.1	Human poliovirus 1
5451.	AAT06860.1	Human poliovirus 1
5452.	AAT06859.1	Human poliovirus 1
5453.	AAT06858.1	Human poliovirus 1
5454.	AAT06857.1	Human poliovirus 1
5455.	AAT06856.1	Human poliovirus 1
5456.	AAT06855.1	Human poliovirus 1
5457.	AAT06854.1	Human poliovirus 1
5458.	AAT06853.1	Human poliovirus 1
5459.	AAT06849.1	Human poliovirus 1
5460.	AAT06848.1	Human poliovirus 1
5461.	AAT06847.1	Human poliovirus 1
5462.	AAT06846.1	Human poliovirus 1
5463.	AAT06845.1	Human poliovirus 1
5464.	AAT06844.1	Human poliovirus 1
5465.	AAB63214.1	Human enterovirus 71
5466.	AAB63213.1	Human enterovirus 71
5467.	AAB63212.1	Human enterovirus 71
5468.	AAB63211.1	Human enterovirus 71
5469.	AAB63210.1	Human enterovirus 71
5470.	BAK20218.1	Human echovirus 30
5471.	BAK20217.1	Human echovirus 30
5472.	BAK20216.1	Human echovirus 30
5473.	BAK20215.1	Human echovirus 30
5474.	BAK20214.1	Human echovirus 30
5475.	BAK20213.1	Human echovirus 30
5476.	BAK20212.1	Human echovirus 30
5477.	BAK20211.1	Human echovirus 30
5478.	BAK20210.1	Human echovirus 30
5479.	BAK20209.1	Human echovirus 30
5480.	BAK20208.1	Human echovirus 30
5481.	BAK20207.1	Human echovirus 30
5482.	BAK20206.1	Human echovirus 30
5483.	BAK20205.1	Human echovirus 30
5484.	BAK20204.1	Human echovirus 30
5485.	BAK20203.1	Human echovirus 30
5486.	BAK20202.1	Human echovirus 30
5487.	BAK20201.1	Human echovirus 30
5488.	BAK20200.1	Human echovirus 30
5489.	BAK20199.1	Human echovirus 30

5490.	BAK20198.1	Human echovirus 30
5491.	BAK20197.1	Human echovirus 30
5492.	BAK20196.1	Human echovirus 30
5493.	BAH80535.1	Human enterovirus 71 355/Toyama/2006
5494.	BAH80534.1	Human enterovirus 71 354/Toyama/2006
5495.	BAH80533.1	Human enterovirus 71 353/Toyama/2006
5496.	BAH80532.1	Human enterovirus 71 351/Toyama/2006
5497.	BAH80531.1	Human enterovirus 71 350/Toyama/2006
5498.	BAH80530.1	Human enterovirus 71 349/Toyama/2006
5499.	BAH80529.1	Human enterovirus 71 348/Toyama/2006
5500.	BAH80528.1	Human enterovirus 71 347/Toyama/2006
5501.	BAH80527.1	Human enterovirus 71 346/Toyama/2006
5502.	BAH80526.1	Human enterovirus 71 343/Toyama/2006
5503.	BAH80487.1	Human coxsackievirus A16 252/Toyama/2002
5504.	BAH80486.1	Human coxsackievirus A16 251/Toyama/2002
5505.	BAH80485.1	Human coxsackievirus A16 250/Toyama/2002
5506.	BAH80484.1	Human coxsackievirus A16 249/Toyama/2002
5507.	BAH80483.1	Human coxsackievirus A16 248/Toyama/2002
5508.	BAH80482.1	Human coxsackievirus A16 247/Toyama/2002
5509.	BAH80481.1	Human coxsackievirus A16 246/Toyama/2002
5510.	BAH80480.1	Human coxsackievirus A16 228/Toyama/2002
5511.	BAH80479.1	Human coxsackievirus A16 227/Toyama/2002
5512.	BAH80478.1	Human coxsackievirus A16 124/Toyama/2002
5513.	BAH80477.1	Human coxsackievirus A16 223/Toyama/2000
5514.	BAH80476.1	Human coxsackievirus A16 188/Toyama/2000
5515.	BAH80493.1	Human coxsackievirus A16 260/Toyama/2002
5516.	BAH80492.1	Human coxsackievirus A16 259/Toyama/2002
5517.	BAH80491.1	Human coxsackievirus A16 258/Toyama/2002
5518.	BAH80490.1	Human coxsackievirus A16 256/Toyama/2002
5519.	BAH80475.1	Human coxsackievirus A16 927/Toyama/1998
5520.	BAH80474.1	Human coxsackievirus A16 392/Toyama/1995
5521.	BAH80473.1	Human coxsackievirus A16 107/Toyama/1990
5522.	BAH80489.1	Human coxsackievirus A16 255/Toyama/2002
5523.	BAH80488.1	Human coxsackievirus A16 253/Toyama/2002
5524.	BAH80472.1	Human coxsackievirus A16 576/Toyama/1988
5525.	BAH80471.1	Human coxsackievirus A16 379/Toyama/1984
5526.	BAH80470.1	Human coxsackievirus A16 24/Toyama/1981
5527.	BAH80525.1	Human enterovirus 71 342/Toyama/2006
5528.	BAH80524.1	Human enterovirus 71 341/Toyama/2006
5529.	BAH80523.1	Human enterovirus 71 340/Toyama/2006
5530.	BAH80522.1	Human enterovirus 71 339/Toyama/2006
5531.	BAH80521.1	Human enterovirus 71 338/Toyama/2006
5532.	BAH80520.1	Human enterovirus 71 287/Toyama/2003
5533.	BAH80519.1	Human enterovirus 71 271/Toyama/2003

5534.	BAH80518.1	Human enterovirus 71 270/Toyama/2003
5535.	BAH80517.1	Human enterovirus 71 185/Toyama/2000
5536.	BAH80516.1	Human enterovirus 71 818/Toyama/1997
5537.	BAH80515.1	Human enterovirus 71 814/Toyama/1997
5538.	BAH80514.1	Human enterovirus 71 800/Toyama/1997
5539.	BAH80513.1	Human enterovirus 71 763/Toyama/1997
5540.	BAH80512.1	Human enterovirus 71 278/Toyama/1994
5541.	BAH80511.1	Human enterovirus 71 937/Toyama/1989
5542.	BAH80510.1	Human enterovirus 71 204/Toyama/1983
5543.	BAH80509.1	Human coxsackievirus A16 460/Toyama/2007
5544.	BAH80508.1	Human coxsackievirus A16 459/Toyama/2007
5545.	BAH80507.1	Human coxsackievirus A16 419/Toyama/2006
5546.	BAH80506.1	Human coxsackievirus A16 418/Toyama/2006
5547.	BAH80505.1	Human coxsackievirus A16 355/Toyama/2005
5548.	BAH80504.1	Human coxsackievirus A16 290/Toyama/2003
5549.	BAH80503.1	Human coxsackievirus A16 120/Toyama/2003
5550.	BAH80502.1	Human coxsackievirus A16 298/Toyama/2002
5551.	BAH80501.1	Human coxsackievirus A16 295/Toyama/2002
5552.	BAH80500.1	Human coxsackievirus A16 283/Toyama/2002
5553.	BAH80499.1	Human coxsackievirus A16 266/Toyama/2002
5554.	BAH80498.1	Human coxsackievirus A16 265/Toyama/2002
5555.	BAH80497.1	Human coxsackievirus A16 264/Toyama/2002
5556.	BAH80496.1	Human coxsackievirus A16 263/Toyama/2002
5557.	BAH80495.1	Human coxsackievirus A16 262/Toyama/2002
5558.	BAH80494.1	Human coxsackievirus A16 261/Toyama/2002
5559.	BAK64424.1	Human enterovirus 68
5560.	BAK64425.1	Human enterovirus 68
5561.	BAK64426.1	Human enterovirus 68
5562.	BAK64427.1	Human enterovirus 68
5563.	BAK64428.1	Human enterovirus 68
5564.	BAK64429.1	Human enterovirus 68
5565.	BAK64430.1	Human enterovirus 68
5566.	BAK64416.1	Human enterovirus 68
5567.	BAK64417.1	Human enterovirus 68
5568.	BAK64418.1	Human enterovirus 68
5569.	BAK64419.1	Human enterovirus 68
5570.	BAK64420.1	Human enterovirus 68
5571.	BAK64421.1	Human enterovirus 68
5572.	BAK64422.1	Human enterovirus 68
5573.	BAK64423.1	Human enterovirus 68
5574.	AAM12176.2	Human poliovirus 1
5575.	AAM12180.1	Human poliovirus 1
5576.	AAM12179.1	Human poliovirus 1
5577.	AAM12178.1	Human poliovirus 1

5578.	AAM12177.1	Human poliovirus 1
5579.	AAM12175.1	Human poliovirus 1
5580.	AAM12174.1	Human poliovirus 1
5581.	AAM12173.1	Human poliovirus 1
5582.	AAM12172.1	Human poliovirus 1
5583.	AAM12171.1	Human poliovirus 1
5584.	AAM12169.1	Human poliovirus 1
5585.	AAM12168.1	Human poliovirus 1
5586.	AAM12167.1	Human poliovirus 1
5587.	AAM12166.1	Human poliovirus 1
5588.	AAM12165.1	Human poliovirus 1
5589.	AAM12164.1	Human poliovirus 1
5590.	AAM12163.1	Human poliovirus 1
5591.	AAM12162.1	Human poliovirus 1
5592.	AAM12161.1	Human poliovirus 1
5593.	AAM12160.1	Human poliovirus 1
5594.	AAM12159.1	Human poliovirus 1
5595.	AAM12158.1	Human poliovirus 1
5596.	AAM12157.1	Human poliovirus 1
5597.	AAM12156.1	Human poliovirus 1
5598.	AAM12155.1	Human poliovirus 1
5599.	AAL96407.1	Human poliovirus 1
5600.	AAL96406.1	Human poliovirus 1
5601.	AAL96405.1	Human poliovirus 1
5602.	AAL96404.1	Human poliovirus 1
5603.	AAL96403.1	Human poliovirus 1
5604.	AAL96402.1	Human poliovirus 1
5605.	AAL96401.1	Human poliovirus 1
5606.	AAL96400.1	Human poliovirus 1
5607.	AAL96399.1	Human poliovirus 1
5608.	AAL96398.1	Human poliovirus 1
5609.	AAL96397.1	Human poliovirus 1
5610.	AAL96396.1	Human poliovirus 1
5611.	AAL96395.1	Human poliovirus 1
5612.	AAL96394.1	Human poliovirus 1
5613.	AAL96393.1	Human poliovirus 1
5614.	AAL96392.1	Human poliovirus 1
5615.	AAL96391.1	Human poliovirus 1
5616.	AAL96390.1	Human poliovirus 1
5617.	AAL96389.1	Human poliovirus 1
5618.	AAL96388.1	Human poliovirus 1
5619.	AAL96387.1	Human poliovirus 1
5620.	AAL96386.1	Human poliovirus 1
5621.	AAL96385.1	Human poliovirus 1

5622.	AAL96384.1	Human poliovirus 1
5623.	AAL96383.1	Human poliovirus 1
5624.	AAL96382.1	Human poliovirus 1
5625.	AAL96381.1	Human poliovirus 1
5626.	AAL96380.1	Human poliovirus 1
5627.	AAL96379.1	Human poliovirus 1
5628.	AAL96408.1	Human poliovirus 1
5629.	BAD72852.1	Human poliovirus 1
5630.	BAD72851.1	Human poliovirus 1
5631.	BAD72850.1	Human poliovirus 1
5632.	BAD72849.1	Human poliovirus 1
5633.	BAD72848.1	Human poliovirus 1
5634.	BAD72847.1	Human poliovirus 1
5635.	BAD72846.1	Human poliovirus 1
5636.	BAD72845.1	Human poliovirus 1
5637.	BAD72844.1	Human poliovirus 1
5638.	BAD72843.1	Human poliovirus 1
5639.	BAD72842.1	Human poliovirus 1
5640.	BAD72841.1	Human poliovirus 1
5641.	BAD08236.1	Human enterovirus 71
5642.	BAD08241.1	Human enterovirus 71
5643.	BAD08240.1	Human enterovirus 71
5644.	BAD08239.1	Human enterovirus 71
5645.	BAD08238.1	Human enterovirus 71
5646.	BAD08237.1	Human enterovirus 71
5647.	AAO48547.1	Human enterovirus 71
5648.	AAO48546.1	Human enterovirus 71
5649.	AAO48545.1	Human enterovirus 71
5650.	AAO48544.1	Human enterovirus 71
5651.	AAO48543.1	Human enterovirus 71
5652.	AAO48542.1	Human enterovirus 71
5653.	AAO48541.1	Human enterovirus 71
5654.	AAO48540.1	Human enterovirus 71
5655.	AAO48539.1	Human enterovirus 71
5656.	AAO48538.1	Human enterovirus 71
5657.	AAO48537.1	Human enterovirus 71
5658.	AAO48536.1	Human enterovirus 71
5659.	AAO48535.1	Human enterovirus 71
5660.	AAO48534.1	Human enterovirus 71
5661.	AAO48533.1	Human enterovirus 71
5662.	AAO48532.1	Human enterovirus 71
5663.	AAO48531.1	Human enterovirus 71
5664.	AAO48530.1	Human enterovirus 71
5665.	AAO48529.1	Human enterovirus 71

5666.	AAO48528.1	Human enterovirus 71
5667.	ADC43796.1	Human echovirus 19
5668.	ADC43795.1	Human echovirus 14
5669.	ADC43794.1	Human echovirus 7
5670.	ADC43793.1	Human echovirus 6
5671.	ADC43792.1	Human echovirus 3
5672.	ADC43791.1	Human coxsackievirus B5
5673.	ADC43790.1	Human coxsackievirus B3
5674.	ADC43789.1	Human coxsackievirus B3
5675.	ADC43788.1	Human coxsackievirus B3
5676.	AAT06852.1	Human poliovirus 1
5677.	AAT06851.1	Human poliovirus 1
5678.	AAT06850.1	Human poliovirus 1
5679.	AAQ11823.1	Human poliovirus 1
5680.	AAQ11822.1	Human poliovirus 1
5681.	AAQ11821.1	Human poliovirus 1
5682.	AAQ11820.1	Human poliovirus 1
5683.	AAQ11819.1	Human poliovirus 1
5684.	AAQ11818.1	Human poliovirus 1
5685.	AAQ11817.1	Human poliovirus 1
5686.	AAQ11816.1	Human poliovirus 1
5687.	AAQ11815.1	Human poliovirus 1
5688.	AAQ11814.1	Human poliovirus 1
5689.	AAQ11813.1	Human poliovirus 1
5690.	AAQ11812.1	Human poliovirus 1
5691.	AAQ11811.1	Human poliovirus 1
5692.	AAQ11810.1	Human poliovirus 1
5693.	AAQ11809.1	Human poliovirus 1
5694.	AAQ11808.1	Human poliovirus 1
5695.	AAQ11807.1	Human poliovirus 1
5696.	AAQ11806.1	Human poliovirus 1
5697.	AAQ11805.1	Human poliovirus 1
5698.	AAQ11804.1	Human poliovirus 1
5699.	AAQ11803.1	Human poliovirus 1
5700.	AAQ11802.1	Human poliovirus 1
5701.	AAQ11801.1	Human poliovirus 1
5702.	AAQ11800.1	Human poliovirus 1
5703.	AAQ11799.1	Human poliovirus 1
5704.	AAQ11798.1	Human poliovirus 1
5705.	AAQ11797.1	Human poliovirus 1
5706.	AAQ11796.1	Human poliovirus 1
5707.	AAQ11795.1	Human poliovirus 1
5708.	AAQ11794.1	Human poliovirus 1
5709.	AAQ11793.1	Human poliovirus 1

5710.	AAQ11792.1	Human poliovirus 1
5711.	AAQ11791.1	Human poliovirus 1
5712.	AAQ11790.1	Human poliovirus 1
5713.	AAQ11789.1	Human poliovirus 1
5714.	AAQ11788.1	Human poliovirus 1
5715.	AAQ11787.1	Human poliovirus 1
5716.	AAQ11786.1	Human poliovirus 1
5717.	AAQ11785.1	Human poliovirus 1
5718.	AAQ11784.1	Human poliovirus 1
5719.	AAQ11783.1	Human poliovirus 1
5720.	AAQ11782.1	Human poliovirus 1
5721.	AAQ11781.1	Human poliovirus 1
5722.	AAQ11780.1	Human poliovirus 1
5723.	AAQ11779.1	Human poliovirus 1
5724.	AAQ11778.1	Human poliovirus 1
5725.	AAQ11777.1	Human poliovirus 1
5726.	AAQ11776.1	Human poliovirus 1
5727.	AAQ11775.1	Human poliovirus 1
5728.	AAQ11774.1	Human poliovirus 1
5729.	AAQ11773.1	Human poliovirus 1
5730.	AAQ11772.1	Human poliovirus 1
5731.	AAQ11771.1	Human poliovirus 1
5732.	AAQ11770.1	Human poliovirus 1
5733.	AAQ11769.1	Human poliovirus 1
5734.	AAQ11768.1	Human poliovirus 1
5735.	AAQ11767.1	Human poliovirus 1
5736.	AAQ11766.1	Human poliovirus 1
5737.	AAQ11765.1	Human poliovirus 1
5738.	AAQ11764.1	Human poliovirus 1
5739.	AAQ11763.1	Human poliovirus 1
5740.	AAQ11762.1	Human poliovirus 1
5741.	AAQ11761.1	Human poliovirus 1
5742.	AAQ11760.1	Human poliovirus 1
5743.	AAQ11759.1	Human poliovirus 1
5744.	AAQ11758.1	Human poliovirus 1
5745.	AAQ11757.1	Human poliovirus 1
5746.	AAQ11756.1	Human poliovirus 1
5747.	AAQ11755.1	Human poliovirus 1
5748.	AAQ11754.1	Human poliovirus 1
5749.	AAQ11753.1	Human poliovirus 1
5750.	AAQ11752.1	Human poliovirus 1
5751.	AAQ11751.1	Human poliovirus 1
5752.	AAQ11750.1	Human poliovirus 1
5753.	AAQ11749.1	Human poliovirus 1

5754.	AAP83958.1	Human poliovirus 1
5755.	AAP83957.1	Human poliovirus 1
5756.	AAP83956.1	Human poliovirus 1
5757.	AAP83955.1	Human poliovirus 1
5758.	AAP83954.1	Human poliovirus 1
5759.	AAU29364.1	Human poliovirus 3
5760.	AAU29363.1	Human poliovirus 1
5761.	AAT06910.1	Human poliovirus 1
5762.	AAT06909.1	Human poliovirus 1
5763.	AAT06908.1	Human poliovirus 1
5764.	AAT06907.1	Human poliovirus 1
5765.	AAT06906.1	Human poliovirus 1
5766.	AAT06905.1	Human poliovirus 1
5767.	AAT06904.1	Human poliovirus 1
5768.	AAT06903.1	Human poliovirus 1
5769.	AAT06902.1	Human poliovirus 1
5770.	AAT06901.1	Human poliovirus 1
5771.	AAT06900.1	Human poliovirus 1
5772.	AAT06899.1	Human poliovirus 1
5773.	AAT06898.1	Human poliovirus 1
5774.	AAT06897.1	Human poliovirus 1
5775.	AAT06896.1	Human poliovirus 1
5776.	AAT06895.1	Human poliovirus 1
5777.	AAT06894.1	Human poliovirus 1
5778.	AAT06893.1	Human poliovirus 1
5779.	AAT06892.1	Human poliovirus 1
5780.	AAT06891.1	Human poliovirus 1
5781.	AAT06890.1	Human poliovirus 1
5782.	AAT06889.1	Human poliovirus 1
5783.	AAT06888.1	Human poliovirus 1
5784.	AAT06887.1	Human poliovirus 1
5785.	AAT06886.1	Human poliovirus 1
5786.	AAT06885.1	Human poliovirus 1
5787.	AAT06884.1	Human poliovirus 1
5788.	AAT06883.1	Human poliovirus 1
5789.	AAT06882.1	Human poliovirus 1
5790.	AAT06881.1	Human poliovirus 1
5791.	AAT06880.1	Human poliovirus 1
5792.	AAT06879.1	Human poliovirus 1
5793.	AAT06878.1	Human poliovirus 1
5794.	AAT06877.1	Human poliovirus 1
5795.	AAT06876.1	Human poliovirus 1
5796.	AAT06875.1	Human poliovirus 1
5797.	AAT06874.1	Human poliovirus 1

5798.	AAT06873.1	Human poliovirus 1
5799.	AAT06872.1	Human poliovirus 1
5800.	AAT06871.1	Human poliovirus 1
5801.	AAT06870.1	Human poliovirus 1
5802.	AAT06869.1	Human poliovirus 1
5803.	AAT06868.1	Human poliovirus 1
5804.	AAT06867.1	Human poliovirus 1
5805.	AAT06866.1	Human poliovirus 1
5806.	AAT06865.1	Human poliovirus 1
5807.	AAT06864.1	Human poliovirus 1
5808.	AAT06863.1	Human poliovirus 1
5809.	AAT06862.1	Human poliovirus 1
5810.	AAT06861.1	Human poliovirus 1
5811.	AAT06860.1	Human poliovirus 1
5812.	AAT06859.1	Human poliovirus 1
5813.	AAT06858.1	Human poliovirus 1
5814.	AAT06857.1	Human poliovirus 1
5815.	AAT06856.1	Human poliovirus 1
5816.	AAT06855.1	Human poliovirus 1
5817.	AAT06854.1	Human poliovirus 1
5818.	AAT06853.1	Human poliovirus 1
5819.	AAT06849.1	Human poliovirus 1
5820.	AAT06848.1	Human poliovirus 1
5821.	AAT06847.1	Human poliovirus 1
5822.	AAT06846.1	Human poliovirus 1
5823.	AAT06845.1	Human poliovirus 1
5824.	AAT06844.1	Human poliovirus 1
5825.	BAA04522.1	Human enterovirus 70
5826.	BAA04521.1	Human enterovirus 70
5827.	BAA04520.1	Human enterovirus 70
5828.	BAA04519.1	Human enterovirus 70
5829.	BAA04518.1	Human enterovirus 70
5830.	BAA04517.1	Human enterovirus 70
5831.	BAA04516.1	Human enterovirus 70
5832.	BAA04515.1	Human enterovirus 70
5833.	AEQ33602.1	Human coxsackievirus B1
5834.	AEF57533.1	Human echovirus 3
5835.	AEF57531.1	Human coxsackievirus A24
5836.	ADJ10908.1	Human poliovirus 2
5837.	AAL80008.1	Human enterovirus 71
5838.	AAB63247.1	Human enterovirus 71
5839.	AAB63246.1	Human enterovirus 71
5840.	AAB63245.1	Human enterovirus 71
5841.	AAB63244.1	Human enterovirus 71

5842.	AAB63243.1	Human enterovirus 71
5843.	AAB63242.1	Human enterovirus 71
5844.	AAB63241.1	Human enterovirus 71
5845.	AAB63240.1	Human enterovirus 71
5846.	AAB63239.1	Human enterovirus 71
5847.	AAB63238.1	Human enterovirus 71
5848.	AAB63237.1	Human enterovirus 71
5849.	AAB63236.1	Human enterovirus 71
5850.	AAB63235.1	Human enterovirus 71
5851.	AAB63234.1	Human enterovirus 71
5852.	AAB63233.1	Human enterovirus 71
5853.	AAB63232.1	Human enterovirus 71
5854.	AAB63231.1	Human enterovirus 71
5855.	AAB63230.1	Human enterovirus 71
5856.	AAB63229.1	Human enterovirus 71
5857.	AAB63228.1	Human enterovirus 71
5858.	AAB63227.1	Human enterovirus 71
5859.	AAB63226.1	Human enterovirus 71
5860.	AAB63225.1	Human enterovirus 71
5861.	AAB63224.1	Human enterovirus 71
5862.	AAB63223.1	Human enterovirus 71
5863.	AAB63222.1	Human enterovirus 71
5864.	AAB63221.1	Human enterovirus 71
5865.	AAB63220.1	Human enterovirus 71
5866.	AAB63219.1	Human enterovirus 71
5867.	AAB63218.1	Human enterovirus 71
5868.	AAB63217.1	Human enterovirus 71
5869.	AAB63216.1	Human enterovirus 71
5870.	AAB63215.1	Human enterovirus 71
5871.	AAT06843.1	Human poliovirus 1
5872.	AAT06842.1	Human poliovirus 1
5873.	AAT06841.1	Human poliovirus 1
5874.	AAT06840.1	Human poliovirus 1
5875.	AAT06839.1	Human poliovirus 1
5876.	AAP56310.1	Human poliovirus 1
5877.	ABP87976.1	Human enterovirus 90
5878.	CAL23420.1	Human coxsackievirus A16
5879.	CAL23419.1	Human coxsackievirus A16
5880.	CAL23418.1	Human coxsackievirus A16
5881.	CAL23417.1	Human coxsackievirus A16
5882.	CAL23416.1	Human coxsackievirus A16
5883.	CAL23415.1	Human coxsackievirus A16
5884.	CAL23414.1	Human coxsackievirus A16
5885.	CAL23413.1	Human coxsackievirus A16

5886.	CAL23412.1	Human coxsackievirus A16
5887.	CAL23411.1	Human coxsackievirus A16
5888.	CAL23410.1	Human coxsackievirus A16
5889.	CAL23409.1	Human coxsackievirus A16
5890.	CAL23408.1	Human coxsackievirus A16
5891.	CAL23407.1	Human coxsackievirus A16
5892.	CAL23406.1	Human coxsackievirus A16
5893.	CAL23405.1	Human coxsackievirus A16
5894.	CAL23404.1	Human coxsackievirus A16
5895.	CAL23403.1	Human coxsackievirus A16
5896.	CAL23402.1	Human coxsackievirus A16
5897.	CAL23401.1	Human coxsackievirus A16
5898.	CAL23400.1	Human coxsackievirus A16
5899.	CAL23399.1	Human coxsackievirus A16
5900.	CAL23398.1	Human coxsackievirus A16
5901.	CAL23397.1	Human coxsackievirus A16
5902.	CAL23396.1	Human coxsackievirus A16
5903.	CAL23395.1	Human coxsackievirus A16
5904.	CAL23394.1	Human coxsackievirus A16
5905.	CAL23393.1	Human coxsackievirus A16
5906.	CAL23392.1	Human coxsackievirus A16
5907.	CAL23391.1	Human coxsackievirus A16
5908.	CAL23390.1	Human coxsackievirus A16
5909.	CAL23389.1	Human coxsackievirus A16
5910.	CAL23388.1	Human coxsackievirus A16
5911.	CAL23387.1	Human coxsackievirus A16
5912.	CAL23386.1	Human coxsackievirus A16
5913.	CAL23385.1	Human coxsackievirus A16
5914.	CAL23384.1	Human coxsackievirus A16
5915.	CAL23383.1	Human coxsackievirus A16
5916.	CAL23382.1	Human coxsackievirus A16
5917.	CAL23381.1	Human coxsackievirus A16
5918.	CAL23380.1	Human coxsackievirus A16
5919.	CAL23379.1	Human coxsackievirus A16
5920.	CAL23378.1	Human coxsackievirus A16
5921.	CAL23377.1	Human coxsackievirus A16
5922.	CAL23376.1	Human coxsackievirus A16
5923.	CAL23375.1	Human coxsackievirus A16
5924.	CAL23374.1	Human coxsackievirus A16
5925.	CAL23373.1	Human coxsackievirus A16
5926.	CAL23372.1	Human coxsackievirus A16
5927.	CAL23371.1	Human coxsackievirus A16
5928.	CAL23370.1	Human coxsackievirus A16
5929.	CAL23369.1	Human coxsackievirus A16

5930.	CAB81943.1	Human echovirus 30
5931.	CAB81942.1	Human echovirus 30
5932.	ADC32811.1	Human echovirus 24
5933.	CAP74038.1	Human enterovirus 71 H0/6364/255/2006
5934.	CAP74037.1	Human enterovirus 71 H0/6344/348/2006
5935.	CAP74036.1	Human enterovirus 71 H0/6290/135/2006
5936.	CAP74035.1	Human enterovirus 71 H0/6332/439/2006
5937.	CAP74034.1	Human enterovirus 71 H0/5454/265/2005
5938.	CAP74033.1	Human enterovirus 71 H0/6344/349/2006
5939.	CAP74032.1	Human enterovirus 71 H0/6290/136/2006
5940.	CAP74031.1	Human enterovirus 71 H0/6334/528/2006
5941.	CAP74030.1	Human enterovirus 71 H0/6486/445/2006
5942.	CAP74029.1	Human enterovirus 71 H0/5440/288/2005
5943.	CAP74028.1	Human enterovirus 71 STH/MCN/2006
5944.	CAP74027.1	Human enterovirus 71 EP/4709/2001
5945.	CAP74026.1	Human enterovirus 71 EP/12105/2001
5946.	CAP74025.1	Human enterovirus 71 EP/449/2000
5947.	CAP74024.1	Human enterovirus 71 EP/17728/1998
5948.	CAP74023.1	Human enterovirus 71 EP/13372/1998
5949.	CAP74022.1	Human enterovirus 71 EP/12081/2001
5950.	CAP74021.1	Human enterovirus 71 EP/11168/2000
5951.	CAP74020.1	Human enterovirus 71 EP/11052/2001
5952.	CAP74019.1	Human enterovirus 71 EP/9027/1999
5953.	CAP74018.1	Human enterovirus 71 EP/8300/2004
5954.	CAP74017.1	Human enterovirus 71 EP/8277/2001
5955.	CAP74016.1	Human enterovirus 71 EP/7414/1999
5956.	CAP74015.1	Human enterovirus 71 EP/5746/2001
5957.	CAP74014.1	Human enterovirus 71 EP/5622/1999
5958.	CAP74013.1	Human enterovirus 71 EP/3267/2002
5959.	CAP74012.1	Human enterovirus 71 EP/2826/2002
5960.	CAP74011.1	Human enterovirus 71 EP/2405/2001
5961.	CAP74010.1	Human enterovirus 71 EP/2353/2002
5962.	CAP74009.1	Human enterovirus 71 H0/6314/248/2006
5963.	CAP74008.1	Human enterovirus 71 EP/2063/2001
5964.	CAP74007.1	Human enterovirus 71 H0/6438/418/2006
5965.	AAO34580.1	Human echovirus 33
5966.	AAO34579.1	Human echovirus 33
5967.	AAO34578.1	Human echovirus 33
5968.	AAO34577.1	Human echovirus 33
5969.	AAO34576.1	Human echovirus 33
5970.	AAO34575.1	Human echovirus 33
5971.	AAO34574.1	Human echovirus 33
5972.	AAO34573.1	Human echovirus 33
5973.	AAO34572.1	Human echovirus 33

5974.	AAO34571.1	Human echovirus 33
5975.	AAO34570.1	Human echovirus 33
5976.	AAO34569.1	Human echovirus 33
5977.	AAO34568.1	Human echovirus 33
5978.	AAO34567.1	Human echovirus 33
5979.	AAO34566.1	Human echovirus 33
5980.	AAO34565.1	Human echovirus 33
5981.	AAO34564.1	Human echovirus 33
5982.	AAO34563.1	Human echovirus 33
5983.	AAO34562.1	Human echovirus 33
5984.	AAO34561.1	Human echovirus 33
5985.	AAO34560.1	Human echovirus 33
5986.	AAO34559.1	Human echovirus 33
5987.	AAO34558.1	Human echovirus 33
5988.	AAO34557.1	Human echovirus 33
5989.	AAO34556.1	Human echovirus 33
5990.	AAO34555.1	Human echovirus 33
5991.	AAO34554.1	Human echovirus 33
5992.	AAO34553.1	Human echovirus 33
5993.	AAO34552.1	Human echovirus 33
5994.	AAO34551.1	Human echovirus 33
5995.	AAO34550.1	Human echovirus 33
5996.	AAO34549.1	Human echovirus 33
5997.	AAO34548.1	Human echovirus 33
5998.	AAO34547.1	Human echovirus 33
5999.	AAO34546.1	Human echovirus 33
6000.	AAO34545.1	Human echovirus 33
6001.	AAO34544.1	Human echovirus 33
6002.	AAO34543.1	Human echovirus 33
6003.	AAO34542.1	Human echovirus 33
6004.	AAO34541.1	Human echovirus 33
6005.	AAO34540.1	Human echovirus 33
6006.	AAO34539.1	Human echovirus 33
6007.	ABI20563.1	Human enterovirus 71
6008.	ABI20562.1	Human enterovirus 71
6009.	ABI20561.1	Human enterovirus 71
6010.	ABI20560.1	Human enterovirus 71
6011.	ABI20559.1	Human enterovirus 71
6012.	ABI20558.1	Human enterovirus 71
6013.	ABI20557.1	Human enterovirus 71
6014.	ABI20556.1	Human enterovirus 71
6015.	ABI20555.1	Human enterovirus 71
6016.	ABI20554.1	Human enterovirus 71
6017.	ABI20553.1	Human enterovirus 71

6018.	ABI20552.1	Human enterovirus 71
6019.	ABI20551.1	Human enterovirus 71
6020.	ABI20550.1	Human enterovirus 71
6021.	ABI20549.1	Human enterovirus 71
6022.	ABI20548.1	Human enterovirus 71
6023.	ABI20547.1	Human enterovirus 71
6024.	ABI20546.1	Human enterovirus 71
6025.	ABI20545.1	Human enterovirus 71
6026.	ABI20544.1	Human enterovirus 71
6027.	ABI20543.1	Human enterovirus 71
6028.	ABI20542.1	Human enterovirus 71
6029.	ABI20541.1	Human enterovirus 71
6030.	ABI20540.1	Human enterovirus 71
6031.	ABI20539.1	Human enterovirus 71
6032.	ABI20538.1	Human enterovirus 71
6033.	ABI20537.1	Human enterovirus 71
6034.	ABI20536.1	Human enterovirus 71
6035.	ABI20535.1	Human enterovirus 71
6036.	ABI20534.1	Human enterovirus 71
6037.	ABI20533.1	Human enterovirus 71
6038.	ABI20532.1	Human enterovirus 71
6039.	ABI20531.1	Human enterovirus 71
6040.	ABI20530.1	Human enterovirus 71
6041.	ABI20529.1	Human enterovirus 71
6042.	ABI20528.1	Human enterovirus 71
6043.	ABI20527.1	Human enterovirus 71
6044.	ABI20526.1	Human enterovirus 71
6045.	ABI20525.1	Human enterovirus 71
6046.	ABI20524.1	Human enterovirus 71
6047.	ABI20523.1	Human enterovirus 71
6048.	BAA04532.1	Human enterovirus 70
6049.	BAA04531.1	Human enterovirus 70
6050.	BAA04530.1	Human enterovirus 70
6051.	BAA04529.1	Human enterovirus 70
6052.	BAA04528.1	Human enterovirus 70
6053.	BAA04527.1	Human enterovirus 70
6054.	BAA04526.1	Human enterovirus 70
6055.	BAA04525.1	Human enterovirus 70
6056.	BAA04524.1	Human enterovirus 70
6057.	BAA04523.1	Human enterovirus 70
6058.	BAF46237.1	Human coxsackievirus A16
6059.	ABA55826.1	Human echovirus 30
6060.	ABA55825.1	Human echovirus 30
6061.	CAA07796.2	Human poliovirus 1

6062.	CAA07795.2	Human poliovirus 1
6063.	CAA07794.2	Human poliovirus 1
6064.	CAA07793.2	Human poliovirus 1
6065.	CAC17721.1	Human poliovirus 3
6066.	CAB77529.1	Human poliovirus 2
6067.	CAB77528.1	Human poliovirus 2
6068.	CAB77527.1	Human poliovirus 2
6069.	CAB77526.1	Human poliovirus 2
6070.	CAC17720.1	Human poliovirus 2
6071.	CAB53944.1	Human echovirus 20
6072.	CAB53943.1	Human echovirus 20
6073.	CAB53963.1	Human echovirus 33
6074.	CAB53960.1	Human echovirus 31
6075.	CAB53947.1	Human echovirus 29
6076.	CAB53946.1	Human echovirus 26
6077.	CAB53945.1	Human echovirus 24
6078.	CAB53969.1	Human echovirus 7
6079.	CAB53962.1	Human echovirus 3
6080.	CAB53965.1	Human echovirus 4
6081.	CAB53967.1	Human echovirus 6
6082.	CAB53968.1	Human echovirus 7
6083.	CAB53966.1	Human echovirus 5
6084.	CAB53964.1	Human echovirus 4
6085.	CAB53961.1	Human echovirus 3
6086.	CAB53935.1	Human echovirus 12
6087.	CAB53941.1	Human echovirus 19
6088.	CAB53942.1	Human echovirus 20
6089.	CAB53940.1	Human echovirus 19
6090.	CAB53939.1	Human echovirus 15
6091.	CAB53938.1	Human echovirus 14
6092.	CAB53937.1	Human echovirus 13
6093.	CAB53936.1	Human echovirus 1
6094.	CAB40215.1	Human echovirus 30
6095.	CAB40214.1	Human echovirus 30
6096.	CAB40213.1	Human echovirus 30
6097.	CAB40212.1	Human echovirus 30
6098.	CAB40211.1	Human echovirus 30
6099.	BAD88547.1	Human echovirus 30
6100.	BAD88546.1	Human echovirus 30
6101.	BAD86825.1	Human echovirus 18
6102.	BAD86824.1	Human echovirus 18
6103.	BAD86823.1	Human echovirus 18
6104.	BAD86822.1	Human echovirus 18
6105.	BAD86821.1	Human echovirus 18

6106.	BAD86820.1	Human echovirus 18
6107.	BAD86817.1	Human echovirus 6
6108.	BAD86816.1	Human echovirus 6
6109.	BAD86815.1	Human echovirus 6
6110.	BAD86814.1	Human echovirus 6
6111.	BAD86813.1	Human echovirus 6
6112.	BAD86812.1	Human echovirus 6
6113.	BAD86811.1	Human echovirus 6
6114.	BAD36914.1	Human echovirus 6
6115.	BAD36913.1	Human echovirus 25
6116.	BAD36912.1	Human echovirus 18
6117.	BAD36911.1	Human coxsackievirus A4
6118.	BAD36910.1	Human coxsackievirus A2
6119.	BAD36909.1	Human coxsackievirus A2
6120.	CAB81944.1	Human echovirus 30
6121.	ADH29909.1	Human echovirus 6
6122.	ADH29908.1	Human echovirus 6
6123.	ADZ93798.1	Human enterovirus 97
6124.	ADZ93787.1	Human echovirus 11
6125.	ADZ93786.1	Human echovirus 11
6126.	ADZ93784.1	Human enterovirus 81
6127.	ADZ93783.1	Human echovirus 32
6128.	ABV00420.1	Human enterovirus 71
6129.	BAE45140.1	Human enterovirus C
6130.	BAE45139.1	Human coxsackievirus A15
6131.	BAE45138.1	Human coxsackievirus A24
6132.	BAE45137.1	Human coxsackievirus A17
6133.	CCC55417.1	Human coxsackievirus A10
6134.	CCC55416.1	Human enterovirus 71
6135.	CCC55415.1	Human enterovirus 71
6136.	CCC55414.1	Human enterovirus 71
6137.	CCC55413.1	Human enterovirus 71
6138.	CCC55412.1	Human enterovirus 71
6139.	CCC55411.1	Human enterovirus 71
6140.	CCC55410.1	Human enterovirus 71
6141.	CCC55409.1	Human enterovirus 71
6142.	CCC55408.1	Human coxsackievirus A16
6143.	CCC55407.1	Human coxsackievirus A16
6144.	CCC55406.1	Human coxsackievirus A16
6145.	CCC55405.1	Human coxsackievirus A16
6146.	CCC55404.1	Human coxsackievirus A16
6147.	CCC55403.1	Human coxsackievirus A16
6148.	CCC55402.1	Human enterovirus 71
6149.	CCC55401.1	Human coxsackievirus A16

6150.	CCC55400.1	Human coxsackievirus A16
6151.	CCC55399.1	Human coxsackievirus A16
6152.	CCC55398.1	Human coxsackievirus A16
6153.	CCC55397.1	Human coxsackievirus A16
6154.	CCC55396.1	Human coxsackievirus A16
6155.	CCC55395.1	Human coxsackievirus A16
6156.	CCC55394.1	Human coxsackievirus A16
6157.	CCC55393.1	Human coxsackievirus A10
6158.	CCC55392.1	Human coxsackievirus A10
6159.	CCC55391.1	Human coxsackievirus A10
6160.	CCC55390.1	Human coxsackievirus A10
6161.	CCC55389.1	Human coxsackievirus A10
6162.	CCC55388.1	Human coxsackievirus A10
6163.	CCC55387.1	Human coxsackievirus A10
6164.	CCC55386.1	Human coxsackievirus A10
6165.	CCC55385.1	Human coxsackievirus A10
6166.	CCC55384.1	Human coxsackievirus A10
6167.	CCC55383.1	Human coxsackievirus A10
6168.	CCC55382.1	Human coxsackievirus A10
6169.	CCC55381.1	Human coxsackievirus A10
6170.	CCC55380.1	Human coxsackievirus A10
6171.	CCC55379.1	Human coxsackievirus A10
6172.	CCC55378.1	Human coxsackievirus A10
6173.	CCC55377.1	Human coxsackievirus A10
6174.	CCC55376.1	Human coxsackievirus A10
6175.	CCC55375.1	Human coxsackievirus A10
6176.	CCC55374.1	Human coxsackievirus A10
6177.	CCC55373.1	Human coxsackievirus A10
6178.	CCC55372.1	Human coxsackievirus A10
6179.	CCC55371.1	Human coxsackievirus A10
6180.	CCC55370.1	Human coxsackievirus A10
6181.	CCC55369.1	Human coxsackievirus A10
6182.	CCC55368.1	Human coxsackievirus A10
6183.	CCC55367.1	Human coxsackievirus A10
6184.	ACB73011.1	Human poliovirus 3
6185.	ACB73008.1	Human poliovirus 2
6186.	AAW78848.1	Human echovirus 30
6187.	AAW78847.1	Human echovirus 30
6188.	AAW78846.1	Human echovirus 30
6189.	AAW78845.1	Human echovirus 30
6190.	AAW78844.1	Human echovirus 30
6191.	AAW78843.1	Human echovirus 30
6192.	AAW78842.1	Human echovirus 30
6193.	AAW78841.1	Human echovirus 30

6194.	AAW78840.1	Human echovirus 30
6195.	AAW78839.1	Human echovirus 30
6196.	AAW78838.1	Human echovirus 30
6197.	AAW78837.1	Human echovirus 30
6198.	ACY74748.1	Human enterovirus 71
6199.	ACY74747.1	Human enterovirus 71
6200.	ACY74746.1	Human enterovirus 71
6201.	ACY74745.1	Human enterovirus 71
6202.	AAO75100.1	Human poliovirus 3
6203.	AAO75099.1	Human poliovirus 3
6204.	AAO75098.1	Human poliovirus 3
6205.	AAO75097.1	Human poliovirus 3
6206.	AAO75096.1	Human poliovirus 3
6207.	AAO75095.1	Human poliovirus 3
6208.	AAO75094.1	Human poliovirus 3
6209.	AAO75093.1	Human poliovirus 3
6210.	AAO75092.1	Human poliovirus 3
6211.	AAO75091.1	Human poliovirus 3
6212.	AAO75090.1	Human poliovirus 3
6213.	AAO75089.1	Human poliovirus 3
6214.	AAO75088.1	Human poliovirus 3
6215.	AAO75087.1	Human poliovirus 3
6216.	AAO75086.1	Human poliovirus 3
6217.	AAO75085.1	Human poliovirus 1
6218.	AAO75084.1	Human poliovirus 1
6219.	AAO75083.1	Human poliovirus 1
6220.	AAO75082.1	Human poliovirus 1
6221.	AAO75081.1	Human poliovirus 1
6222.	AAO75080.1	Human poliovirus 1
6223.	AAO75079.1	Human poliovirus 1
6224.	AAO75078.1	Human poliovirus 1
6225.	AAO75077.1	Human poliovirus 1
6226.	AAO75076.1	Human poliovirus 1
6227.	AAO75075.1	Human poliovirus 1
6228.	AAO75074.1	Human poliovirus 1
6229.	AAO75073.1	Human poliovirus 1
6230.	AAO75072.1	Human poliovirus 1
6231.	AAO75071.1	Human poliovirus 1
6232.	AAO75070.1	Human poliovirus 1
6233.	AAO75069.1	Human poliovirus 1
6234.	AAO75068.1	Human poliovirus 1
6235.	AAO75067.1	Human poliovirus 1
6236.	AAO75066.1	Human poliovirus 1
6237.	AAO75065.1	Human poliovirus 1

6238.	AAO75064.1	Human poliovirus 1
6239.	AAO75063.1	Human poliovirus 1
6240.	AAO75062.1	Human poliovirus 1
6241.	AAO75061.1	Human poliovirus 1
6242.	AAO75060.1	Human poliovirus 1
6243.	AAO75059.1	Human poliovirus 1
6244.	AAO75058.1	Human poliovirus 1
6245.	AAO75057.1	Human poliovirus 1
6246.	AAO75056.1	Human poliovirus 1
6247.	AAO75055.1	Human poliovirus 1
6248.	AAO75054.1	Human poliovirus 1
6249.	AAO75053.1	Human poliovirus 1
6250.	AAO75052.1	Human poliovirus 1
6251.	AAO75051.1	Human poliovirus 1
6252.	AAO75050.1	Human poliovirus 1
6253.	AAO75049.1	Human poliovirus 1
6254.	AAO75048.1	Human poliovirus 1
6255.	AAO75047.1	Human poliovirus 1
6256.	AAO75046.1	Human poliovirus 1
6257.	AAO75045.1	Human poliovirus 1
6258.	AAO75044.1	Human poliovirus 1
6259.	AAO75043.1	Human poliovirus 1
6260.	AAO75042.1	Human poliovirus 1
6261.	AAO75041.1	Human poliovirus 1
6262.	AAO75040.1	Human poliovirus 1
6263.	AAO91792.1	Human enterovirus 71
6264.	NP_740586.1	Human enterovirus C
6265.	AEV23217.1	Human enterovirus 71
6266.	AEV23216.1	Human enterovirus 71
6267.	AEV23215.1	Human enterovirus 71
6268.	AEV23214.1	Human enterovirus 71
6269.	AEV23213.1	Human enterovirus 71
6270.	AEV23212.1	Human enterovirus 71
6271.	AEV23211.1	Human enterovirus 71
6272.	AEV23210.1	Human enterovirus 71
6273.	AEV23209.1	Human enterovirus 71
6274.	AEV23208.1	Human enterovirus 71
6275.	AEV23207.1	Human enterovirus 71
6276.	AEV23206.1	Human enterovirus 71
6277.	AEV23205.1	Human enterovirus 71
6278.	AEV23204.1	Human enterovirus 71
6279.	CCC55340.1	Human coxsackievirus A6
6280.	CCC55339.1	Human coxsackievirus A6
6281.	CCC55338.1	Human coxsackievirus A6

6282.	CCC55337.1	Human coxsackievirus A6
6283.	CCC55336.1	Human coxsackievirus A6
6284.	CCC55335.1	Human coxsackievirus A6
6285.	CCC55334.1	Human coxsackievirus A6
6286.	CCC55333.1	Human coxsackievirus A6
6287.	CCC55332.1	Human coxsackievirus A6
6288.	CCC55331.1	Human coxsackievirus A6
6289.	CCC55330.1	Human coxsackievirus A6
6290.	CCC55329.1	Human coxsackievirus A6
6291.	CCC55328.1	Human coxsackievirus A6
6292.	CCC55327.1	Human coxsackievirus A6
6293.	CCC55326.1	Human coxsackievirus A6
6294.	CCC55325.1	Human coxsackievirus A6
6295.	CCC55324.1	Human coxsackievirus A6
6296.	CCC55323.1	Human coxsackievirus A6
6297.	CCC55322.1	Human coxsackievirus A6
6298.	CCC55321.1	Human coxsackievirus A6
6299.	CCC55320.1	Human coxsackievirus A6
6300.	CCC55319.1	Human coxsackievirus A6
6301.	CCC55318.1	Human coxsackievirus A6
6302.	CCC55317.1	Human coxsackievirus A6
6303.	CCC55316.1	Human coxsackievirus A6
6304.	CCC55315.1	Human coxsackievirus A6
6305.	CCC55314.1	Human coxsackievirus A6
6306.	CCC55313.1	Human coxsackievirus A6
6307.	CCC55312.1	Human coxsackievirus A6
6308.	CCC55311.1	Human coxsackievirus A6
6309.	CCC55310.1	Human coxsackievirus A6
6310.	CCC55309.1	Human coxsackievirus A6
6311.	CCC55308.1	Human coxsackievirus A6
6312.	CCC55307.1	Human coxsackievirus A6
6313.	CCC55306.1	Human coxsackievirus A6
6314.	CCC55305.1	Human coxsackievirus A6
6315.	CCC55304.1	Human coxsackievirus A6
6316.	CCC55303.1	Human coxsackievirus A6
6317.	CCC55302.1	Human coxsackievirus A6
6318.	ADH29914.1	Human echovirus 6
6319.	ADH29913.1	Human echovirus 6
6320.	ADH29912.1	Human echovirus 6
6321.	ADH29911.1	Human echovirus 6
6322.	ADH29910.1	Human echovirus 6
6323.	AEM00606.1	Human enterovirus 68
6324.	AEM00605.1	Human enterovirus 68
6325.	AEM00604.1	Human enterovirus 68

6326.	AEM00603.1	Human enterovirus 68
6327.	AEM00602.1	Human enterovirus 68
6328.	AEM00601.1	Human enterovirus 68
6329.	AEM00600.1	Human enterovirus 68
6330.	AEM00599.1	Human enterovirus 68
6331.	AEM00598.1	Human enterovirus 68
6332.	AEM00597.1	Human enterovirus 68
6333.	AEM00596.1	Human enterovirus 68
6334.	AEM00595.1	Human enterovirus 68
6335.	AEM00594.1	Human enterovirus 68
6336.	AEM00593.1	Human enterovirus 68
6337.	AEM00592.1	Human enterovirus 68
6338.	AEM00591.1	Human enterovirus 68
6339.	AEM00590.1	Human enterovirus 68
6340.	AEM00589.1	Human enterovirus 68
6341.	AEM00588.1	Human enterovirus 68
6342.	AEM00587.1	Human enterovirus 68
6343.	AEM00586.1	Human enterovirus 68
6344.	AEM00585.1	Human enterovirus 68
6345.	AEM00584.1	Human enterovirus 68
6346.	AEM00583.1	Human enterovirus 68
6347.	AEM00582.1	Human enterovirus 68
6348.	AEM00581.1	Human enterovirus 68
6349.	ACH70129.1	Human enterovirus 71
6350.	ACH70128.1	Human enterovirus 71
6351.	ACH70127.1	Human enterovirus 71
6352.	ACH70126.1	Human enterovirus 71
6353.	ACH70125.1	Human enterovirus 71
6354.	ACH70124.1	Human enterovirus 71
6355.	ACH70123.1	Human enterovirus 71
6356.	ACH70122.1	Human enterovirus 71
6357.	ACH70121.1	Human enterovirus 71
6358.	ACH70120.1	Human enterovirus 71
6359.	ACH70119.1	Human enterovirus 71
6360.	ACH70117.1	Human enterovirus 71
6361.	ACH70116.1	Human enterovirus 71
6362.	ACH70115.1	Human enterovirus 71
6363.	ACH70114.1	Human enterovirus 71
6364.	ACH70113.1	Human enterovirus 71
6365.	ACH70112.1	Human enterovirus 71
6366.	ACH70111.1	Human enterovirus 71
6367.	ACH70110.1	Human enterovirus 71
6368.	ACH70107.1	Human enterovirus 71
6369.	ACH70106.1	Human enterovirus 71

6370.	ACH70105.1	Human enterovirus 71
6371.	ACH70104.1	Human enterovirus 71
6372.	ACH70103.1	Human enterovirus 71
6373.	ACH70102.1	Human enterovirus 71
6374.	ACH70101.1	Human enterovirus 71
6375.	ACH70100.1	Human enterovirus 71
6376.	ACH70099.1	Human enterovirus 71
6377.	ACH70098.1	Human enterovirus 71
6378.	ACH70097.1	Human enterovirus 71
6379.	ACH70096.1	Human enterovirus 71
6380.	ACH70094.1	Human enterovirus 71
6381.	ACH70093.1	Human enterovirus 71
6382.	ACH70092.1	Human enterovirus 71
6383.	ACH70091.1	Human enterovirus 71
6384.	ACH70090.1	Human enterovirus 71
6385.	ACH70089.1	Human enterovirus 71
6386.	ACH70088.1	Human enterovirus 71
6387.	ACH70087.1	Human enterovirus 71
6388.	ACH70085.1	Human enterovirus 71
6389.	ACH70084.1	Human enterovirus 71
6390.	ACH70083.1	Human enterovirus 71
6391.	ACH70082.1	Human enterovirus 71
6392.	ACH70081.1	Human enterovirus 71
6393.	ACH70080.1	Human enterovirus 71
6394.	ACH70079.1	Human enterovirus 71
6395.	ACH70078.1	Human enterovirus 71
6396.	ACH70077.1	Human enterovirus 71
6397.	ACH70075.1	Human enterovirus 71
6398.	ACH70074.1	Human enterovirus 71
6399.	AET09717.1	Human enterovirus 71
6400.	AEE65452.1	Human enterovirus 71
6401.	AEE65451.1	Human enterovirus 71
6402.	AEE65450.1	Human enterovirus 71
6403.	AEE65449.1	Human enterovirus 71
6404.	AEE65448.1	Human enterovirus 71
6405.	AEE65447.1	Human enterovirus 71
6406.	AEE65446.1	Human enterovirus 71
6407.	AEE65445.1	Human enterovirus 71
6408.	AEE65444.1	Human enterovirus 71
6409.	AEE65443.1	Human enterovirus 71
6410.	AEE65442.1	Human enterovirus 71
6411.	AEE65441.1	Human enterovirus 71
6412.	AEE65440.1	Human enterovirus 71
6413.	AEE65439.1	Human enterovirus 71

6414.	AEE65438.1	Human enterovirus 71
6415.	AEE65437.1	Human enterovirus 71
6416.	AEE65436.1	Human enterovirus 71
6417.	AEE65435.1	Human enterovirus 71
6418.	AEE65434.1	Human enterovirus 71
6419.	AEE65433.1	Human enterovirus 71
6420.	AEE65432.1	Human enterovirus 71
6421.	AEE65431.1	Human enterovirus 71
6422.	AEE65430.1	Human enterovirus 71
6423.	AEE65429.1	Human enterovirus 71
6424.	AEE65428.1	Human enterovirus 71
6425.	AEE65427.1	Human enterovirus 71
6426.	AEE65426.1	Human enterovirus 71
6427.	AEE65425.1	Human enterovirus 71
6428.	AEE65424.1	Human enterovirus 71
6429.	AEE65423.1	Human enterovirus 71
6430.	AEE65422.1	Human enterovirus 71
6431.	AEE01038.1	Human enterovirus 71
6432.	AEE01037.1	Human enterovirus 71
6433.	AEE01036.1	Human enterovirus 71
6434.	AEE01035.1	Human enterovirus 71
6435.	AEE01034.1	Human enterovirus 71
6436.	AEE01033.1	Human enterovirus 71
6437.	AEE01032.1	Human enterovirus 71
6438.	AEE01031.1	Human enterovirus 71
6439.	AEE01030.1	Human enterovirus 71
6440.	AEE01029.1	Human enterovirus 71
6441.	AEE01028.1	Human enterovirus 71
6442.	AEE01027.1	Human enterovirus 71
6443.	AEE01026.1	Human enterovirus 71
6444.	AEE01025.1	Human enterovirus 71
6445.	AEE01024.1	Human enterovirus 71
6446.	AEE01023.1	Human enterovirus 71
6447.	AEE01022.1	Human enterovirus 71
6448.	AEE01021.1	Human enterovirus 71
6449.	AEE01020.1	Human enterovirus 71
6450.	AEE01019.1	Human enterovirus 71
6451.	AEE01018.1	Human enterovirus 71
6452.	AEE01017.1	Human enterovirus 71
6453.	AEE01016.1	Human enterovirus 71
6454.	AEE01015.1	Human enterovirus 71
6455.	AEE01014.1	Human enterovirus 71
6456.	AEE01013.1	Human enterovirus 71
6457.	AEE01012.1	Human enterovirus 71

6458.	AEE01011.1	Human enterovirus 71
6459.	AEE01010.1	Human enterovirus 71
6460.	AEE01009.1	Human enterovirus 71
6461.	AEE01008.1	Human enterovirus 71
6462.	AEE01007.1	Human enterovirus 71
6463.	AEE01006.1	Human enterovirus 71
6464.	AEE01005.1	Human enterovirus 71
6465.	AEE01004.1	Human enterovirus 71
6466.	AEE01003.1	Human enterovirus 71
6467.	AEE01002.1	Human enterovirus 71
6468.	AEE01001.1	Human enterovirus 71
6469.	AEE01000.1	Human enterovirus 71
6470.	AEE00999.1	Human enterovirus 71
6471.	AEE00998.1	Human enterovirus 71
6472.	AEE00997.1	Human enterovirus 71
6473.	AEE00996.1	Human enterovirus 71
6474.	AEE00995.1	Human enterovirus 71
6475.	AEE00994.1	Human enterovirus 71
6476.	AEE00993.1	Human enterovirus 71
6477.	AEE00992.1	Human enterovirus 71
6478.	AEE00991.1	Human enterovirus 71
6479.	AEE00990.1	Human enterovirus 71
6480.	AEE00989.1	Human enterovirus 71
6481.	AEE00988.1	Human enterovirus 71
6482.	AEE00987.1	Human enterovirus 71
6483.	AEE00986.1	Human enterovirus 71
6484.	AEE00985.1	Human enterovirus 71
6485.	AEE00984.1	Human enterovirus 71
6486.	AEE00983.1	Human enterovirus 71
6487.	AEE00982.1	Human enterovirus 71
6488.	AEE00981.1	Human enterovirus 71
6489.	AEE00980.1	Human enterovirus 71
6490.	AEE00979.1	Human enterovirus 71
6491.	AEE00978.1	Human enterovirus 71
6492.	AEE00977.1	Human enterovirus 71
6493.	AEE00976.1	Human enterovirus 71
6494.	AEE00975.1	Human enterovirus 71
6495.	AEE00974.1	Human enterovirus 71
6496.	AEE00973.1	Human enterovirus 71
6497.	AEE00972.1	Human enterovirus 71
6498.	AEE00971.1	Human enterovirus 71
6499.	AEE00970.1	Human enterovirus 71
6500.	AEE00969.1	Human enterovirus 71
6501.	AEE00968.1	Human enterovirus 71

6502.	AEE00967.1	Human enterovirus 71
6503.	AEE00966.1	Human enterovirus 71
6504.	AEE00965.1	Human enterovirus 71
6505.	AEE00964.1	Human enterovirus 71
6506.	AEE00963.1	Human enterovirus 71
6507.	AEE00962.1	Human enterovirus 71
6508.	AEE00961.1	Human enterovirus 71
6509.	AEE00960.1	Human enterovirus 71
6510.	AEE00959.1	Human enterovirus 71
6511.	AEE00958.1	Human enterovirus 71
6512.	AEE00957.1	Human enterovirus 71
6513.	AEE00956.1	Human enterovirus 71
6514.	AEE00955.1	Human enterovirus 71
6515.	AEE00954.1	Human enterovirus 71
6516.	AEE00953.1	Human enterovirus 71
6517.	AEE00952.1	Human enterovirus 71
6518.	AEE00951.1	Human enterovirus 71
6519.	AEE00950.1	Human enterovirus 71
6520.	AEE00949.1	Human enterovirus 71
6521.	AEE00948.1	Human enterovirus 71
6522.	AEE00947.1	Human enterovirus 71
6523.	AEE00946.1	Human enterovirus 71
6524.	AEE00945.1	Human enterovirus 71
6525.	AEE00944.1	Human enterovirus 71
6526.	AEE00943.1	Human enterovirus 71
6527.	AEE00942.1	Human enterovirus 71
6528.	AEE00941.1	Human enterovirus 71
6529.	AEE00940.1	Human enterovirus 71
6530.	AEE00939.1	Human enterovirus 71
6531.	AEE00938.1	Human enterovirus 71
6532.	AEE00937.1	Human enterovirus 71
6533.	AEE00936.1	Human enterovirus 71
6534.	AEE00935.1	Human enterovirus 71
6535.	AEE00934.1	Human enterovirus 71
6536.	AEE00933.1	Human enterovirus 71
6537.	AEE00932.1	Human enterovirus 71
6538.	AEE00931.1	Human enterovirus 71
6539.	AEE00930.1	Human enterovirus 71
6540.	AEE00929.1	Human enterovirus 71
6541.	AEE00928.1	Human enterovirus 71
6542.	AEE00927.1	Human enterovirus 71
6543.	AEE00926.1	Human enterovirus 71
6544.	AEE00925.1	Human enterovirus 71
6545.	AEE00924.1	Human enterovirus 71

6546.	AEE00923.1	Human enterovirus 71
6547.	AEE00922.1	Human enterovirus 71
6548.	AEE00921.1	Human enterovirus 71
6549.	AEE00920.1	Human enterovirus 71
6550.	AEE00919.1	Human enterovirus 71
6551.	AEE00918.1	Human enterovirus 71
6552.	AEE00917.1	Human enterovirus 71
6553.	AEE00916.1	Human enterovirus 71
6554.	AEE00915.1	Human enterovirus 71
6555.	AEE00914.1	Human enterovirus 71
6556.	AEE00913.1	Human enterovirus 71
6557.	AEE00912.1	Human enterovirus 71
6558.	AEE00911.1	Human enterovirus 71
6559.	AEE00910.1	Human enterovirus 71
6560.	AEE00909.1	Human enterovirus 71
6561.	AEE00908.1	Human enterovirus 71
6562.	AEE00907.1	Human enterovirus 71
6563.	AEE00906.1	Human enterovirus 71
6564.	AEE00905.1	Human enterovirus 71
6565.	AEE00904.1	Human enterovirus 71
6566.	AEE00903.1	Human enterovirus 71
6567.	AEE00902.1	Human enterovirus 71
6568.	AEE00901.1	Human enterovirus 71
6569.	AEE00900.1	Human enterovirus 71
6570.	AEE00899.1	Human enterovirus 71
6571.	AEE00898.1	Human enterovirus 71
6572.	AEE00897.1	Human enterovirus 71
6573.	AEE00896.1	Human enterovirus 71
6574.	AEE00895.1	Human enterovirus 71
6575.	AEE00894.1	Human enterovirus 71
6576.	AEE00893.1	Human enterovirus 71
6577.	AEE00892.1	Human enterovirus 71
6578.	AEE00891.1	Human enterovirus 71
6579.	AEE00890.1	Human enterovirus 71
6580.	AEE00889.1	Human enterovirus 71
6581.	AEE00888.1	Human enterovirus 71
6582.	AEE00887.1	Human enterovirus 71
6583.	AEE00886.1	Human enterovirus 71
6584.	AEE00885.1	Human enterovirus 71
6585.	AEE00884.1	Human enterovirus 71
6586.	AEE00883.1	Human enterovirus 71
6587.	AEE00882.1	Human enterovirus 71
6588.	AEE00881.1	Human enterovirus 71
6589.	AEE00880.1	Human enterovirus 71

6590.	AEE00879.1	Human enterovirus 71
6591.	AEE00878.1	Human enterovirus 71
6592.	AEE00877.1	Human enterovirus 71
6593.	AEE00876.1	Human enterovirus 71
6594.	AEE00875.1	Human enterovirus 71
6595.	AEE00874.1	Human enterovirus 71
6596.	AEE00873.1	Human enterovirus 71
6597.	AEE00872.1	Human enterovirus 71
6598.	AEE00871.1	Human enterovirus 71
6599.	AEE00870.1	Human enterovirus 71
6600.	AEE00869.1	Human enterovirus 71
6601.	AEE00868.1	Human enterovirus 71
6602.	AEE00867.1	Human enterovirus 71
6603.	AEE00866.1	Human enterovirus 71
6604.	AEE00865.1	Human enterovirus 71
6605.	AEE00864.1	Human enterovirus 71
6606.	AEE00863.1	Human enterovirus 71
6607.	AEE00862.1	Human enterovirus 71
6608.	AEE00861.1	Human enterovirus 71
6609.	AEE00860.1	Human enterovirus 71
6610.	AEE00859.1	Human enterovirus 71
6611.	AEE00858.1	Human enterovirus 71
6612.	AEE00857.1	Human enterovirus 71
6613.	AEE00856.1	Human enterovirus 71
6614.	AEE00855.1	Human enterovirus 71
6615.	AEE00854.1	Human enterovirus 71
6616.	AEE00853.1	Human enterovirus 71
6617.	AEE00852.1	Human enterovirus 71
6618.	AEE00851.1	Human enterovirus 71
6619.	AEE00850.1	Human enterovirus 71
6620.	AEE00849.1	Human enterovirus 71
6621.	AEE00848.1	Human enterovirus 71
6622.	AEE00847.1	Human enterovirus 71
6623.	AEE00846.1	Human enterovirus 71
6624.	AEE00845.1	Human enterovirus 71
6625.	AEE00844.1	Human enterovirus 71
6626.	AEE00843.1	Human enterovirus 71
6627.	AEE00842.1	Human enterovirus 71
6628.	AEE00841.1	Human enterovirus 71
6629.	AEE00840.1	Human enterovirus 71
6630.	AEE00839.1	Human enterovirus 71
6631.	AEE00838.1	Human enterovirus 71
6632.	AEE00837.1	Human enterovirus 71
6633.	AEE00836.1	Human enterovirus 71

6634.	NP_740471.1	Poliovirus
6635.	ABI51283.1	Human enterovirus 71
6636.	ABI30925.1	Human enterovirus 71
6637.	ABI30924.1	Human enterovirus 71
6638.	AAY15841.1	Human poliovirus 1
6639.	AAY17258.1	Human coxsackievirus B3
6640.	NP_714932.1	Simian enterovirus A
6641.	CAA24780.1	Human poliovirus 3
6642.	AEI98724.1	Human coxsackievirus A24
6643.	AEI98723.1	Human coxsackievirus A24
6644.	AEI98722.1	Human coxsackievirus A24
6645.	AEI98721.1	Human coxsackievirus A24
6646.	ADK22861.1	Human enterovirus 109
6647.	ADK22860.1	Human enterovirus 109
6648.	ADK22859.1	Human enterovirus 109
6649.	ADK22858.1	Human enterovirus 109
6650.	CAA24452.1	Human poliovirus 1
6651.	ADG63655.1	Human echovirus 25
6652.	ADG63653.1	Human echovirus 25
6653.	ADN86406.1	Human enterovirus 71
6654.	ADN86405.1	Human enterovirus 71
6655.	ADN86404.1	Human enterovirus 71
6656.	ADN86403.1	Human enterovirus 71
6657.	ADN86402.1	Human enterovirus 71
6658.	ADN86401.1	Human enterovirus 71
6659.	ADN86400.1	Human enterovirus 71
6660.	ADN86399.1	Human enterovirus 71
6661.	ADN86398.1	Human enterovirus 71
6662.	ADN86397.1	Human enterovirus 71
6663.	ADN86396.1	Human enterovirus 71
6664.	ADN86395.1	Human enterovirus 71
6665.	ADN86394.1	Human enterovirus 71
6666.	ADN86393.1	Human enterovirus 71
6667.	ADN86392.1	Human enterovirus 71
6668.	ADN86391.1	Human enterovirus 71
6669.	ADN86390.1	Human enterovirus 71
6670.	ADN86389.1	Human enterovirus 71
6671.	ADN86388.1	Human enterovirus 71
6672.	ADN86387.1	Human enterovirus 71
6673.	ADN86386.1	Human enterovirus 71
6674.	ADN86385.1	Human enterovirus 71
6675.	ADN86384.1	Human enterovirus 71
6676.	ADN86383.1	Human enterovirus 71
6677.	ADN86382.1	Human enterovirus 71

6678.	ADN86381.1	Human enterovirus 71
6679.	ADN86380.1	Human enterovirus 71
6680.	YP_003359169.1	Simian picornavirus strain N203
6681.	YP_001718535.1	Simian picornavirus strain N125
6682.	YP_001718546.1	Simian picornavirus 17
6683.	YP_001718579.1	Simian enterovirus SV6
6684.	YP_001718557.1	Simian enterovirus SV43
6685.	YP_001718514.1	Simian enterovirus SV19
6686.	NP_758533.1	Porcine enterovirus B
6687.	NP_740529.1	Human enterovirus A
6688.	ACZ16002.1	Human enterovirus 71
6689.	AAO52680.1	Human enterovirus 71
6690.	ADC55343.1	Human coxsackievirus A16
6691.	ADC55342.1	Human coxsackievirus A16
6692.	ADC55341.1	Human coxsackievirus A16
6693.	ADC55340.1	Human coxsackievirus A16
6694.	ADC55339.1	Human coxsackievirus A16
6695.	ADC55338.1	Human coxsackievirus A16
6696.	ADC55337.1	Human coxsackievirus A16
6697.	ADC55336.1	Human coxsackievirus A16
6698.	ADC55335.1	Human coxsackievirus A16
6699.	ADC55334.1	Human coxsackievirus A16
6700.	ADC55333.1	Human coxsackievirus A16
6701.	ADC55332.1	Human coxsackievirus A16
6702.	ADC55331.1	Human coxsackievirus A16
6703.	ADC55330.1	Human coxsackievirus A16
6704.	ADC55329.1	Human coxsackievirus A16
6705.	ADC55328.1	Human coxsackievirus A16
6706.	ADC55327.1	Human coxsackievirus A16
6707.	ADC55326.1	Human coxsackievirus A16
6708.	ADC55325.1	Human coxsackievirus A16
6709.	ADC55324.1	Human coxsackievirus A16
6710.	ADC55323.1	Human coxsackievirus A16
6711.	ADC55322.1	Human coxsackievirus A16
6712.	ADC55321.1	Human coxsackievirus A16
6713.	ADC55320.1	Human coxsackievirus A16
6714.	ADC55319.1	Human coxsackievirus A16
6715.	ADC55318.1	Human coxsackievirus A16
6716.	ADC55317.1	Human coxsackievirus A16
6717.	ADC55316.1	Human coxsackievirus A16
6718.	ADC55315.1	Human coxsackievirus A16
6719.	ADC55314.1	Human coxsackievirus A16
6720.	ADC55313.1	Human coxsackievirus A16
6721.	ADC55312.1	Human coxsackievirus A16

6722.	ADC55311.1	Human coxsackievirus A16
6723.	ADC55310.1	Human coxsackievirus A16
6724.	ADC55309.1	Human coxsackievirus A16
6725.	ADC55308.1	Human coxsackievirus A16
6726.	ADC55307.1	Human coxsackievirus A16
6727.	ADC55306.1	Human coxsackievirus A16
6728.	ADC55305.1	Human coxsackievirus A16
6729.	ADC55304.1	Human coxsackievirus A16
6730.	ADC55303.1	Human coxsackievirus A16
6731.	ADC55302.1	Human coxsackievirus A16
6732.	ADC55301.1	Human coxsackievirus A16
6733.	ADC55300.1	Human coxsackievirus A16
6734.	ADC55299.1	Human coxsackievirus A16
6735.	ADC55298.1	Human coxsackievirus A16
6736.	ADC55297.1	Human coxsackievirus A16
6737.	ADC55296.1	Human coxsackievirus A16
6738.	ADC55295.1	Human coxsackievirus A16
6739.	ADC55294.1	Human coxsackievirus A16
6740.	ADC55293.1	Human coxsackievirus A16
6741.	ADC55292.1	Human coxsackievirus A16
6742.	ADC55291.1	Human coxsackievirus A16
6743.	ADC55290.1	Human coxsackievirus A16
6744.	ADC55289.1	Human coxsackievirus A16
6745.	ADC55288.1	Human coxsackievirus A16
6746.	ADC55287.1	Human coxsackievirus A16
6747.	ADC55286.1	Human coxsackievirus A16
6748.	ACO51353.1	Human enterovirus 71
6749.	ACO51352.1	Human enterovirus 71
6750.	ACO51351.1	Human enterovirus 71
6751.	ACO51350.1	Human enterovirus 71
6752.	ACO51349.1	Human enterovirus 71
6753.	ACO51348.1	Human enterovirus 71
6754.	ACO51347.1	Human enterovirus 71
6755.	ACO51346.1	Human enterovirus 71
6756.	ACO51345.1	Human enterovirus 71
6757.	ACO51344.1	Human enterovirus 71
6758.	ACO51343.1	Human enterovirus 71
6759.	ACO51342.1	Human enterovirus 71
6760.	ACO51341.1	Human enterovirus 71
6761.	ACO51340.1	Human enterovirus 71
6762.	ACO51339.1	Human enterovirus 71
6763.	ACO51338.1	Human enterovirus 71
6764.	ACO51337.1	Human enterovirus 71
6765.	ACO51336.1	Human enterovirus 71

6766.	ACO51335.1	Human enterovirus 71
6767.	AEE00835.1	Human enterovirus 71
6768.	AEE00834.1	Human enterovirus 71
6769.	AEE00833.1	Human enterovirus 71
6770.	AEE00832.1	Human enterovirus 71
6771.	AEE00831.1	Human enterovirus 71
6772.	AEE00830.1	Human enterovirus 71
6773.	AEE00829.1	Human enterovirus 71
6774.	AEE00828.1	Human enterovirus 71
6775.	AEE00827.1	Human enterovirus 71
6776.	AEE00826.1	Human enterovirus 71
6777.	AEE00825.1	Human enterovirus 71
6778.	AEE00824.1	Human enterovirus 71
6779.	AEE00823.1	Human enterovirus 71
6780.	AEE00822.1	Human enterovirus 71
6781.	AEE00821.1	Human enterovirus 71
6782.	AEE00820.1	Human enterovirus 71
6783.	AEE00819.1	Human enterovirus 71
6784.	AEE00818.1	Human enterovirus 71
6785.	AEE00817.1	Human enterovirus 71
6786.	AEE00816.1	Human enterovirus 71
6787.	AEE00815.1	Human enterovirus 71
6788.	AEE00814.1	Human enterovirus 71
6789.	AEE00813.1	Human enterovirus 71
6790.	AEE00812.1	Human enterovirus 71
6791.	AEE00811.1	Human enterovirus 71
6792.	AEE00810.1	Human enterovirus 71
6793.	AEE00809.1	Human enterovirus 71
6794.	AEE00808.1	Human enterovirus 71
6795.	AEE00807.1	Human enterovirus 71
6796.	AEE00806.1	Human enterovirus 71
6797.	AEE00805.1	Human enterovirus 71
6798.	AEE00804.1	Human enterovirus 71
6799.	AEE00803.1	Human enterovirus 71
6800.	AEE00802.1	Human enterovirus 71
6801.	AEE00801.1	Human enterovirus 71
6802.	AEE00800.1	Human enterovirus 71
6803.	AEE00799.1	Human enterovirus 71
6804.	AEE00798.1	Human enterovirus 71
6805.	AEE00797.1	Human enterovirus 71
6806.	AEE00796.1	Human enterovirus 71
6807.	AEE00795.1	Human enterovirus 71
6808.	AEE00794.1	Human enterovirus 71
6809.	AEE00793.1	Human enterovirus 71

6810.	AEE00792.1	Human enterovirus 71
6811.	AEE00791.1	Human enterovirus 71
6812.	AEE00790.1	Human enterovirus 71
6813.	AEE00789.1	Human enterovirus 71
6814.	AEE00788.1	Human enterovirus 71
6815.	AEE00787.1	Human enterovirus 71
6816.	AEE00786.1	Human enterovirus 71
6817.	AEE00785.1	Human enterovirus 71
6818.	AEE00784.1	Human enterovirus 71
6819.	AEE00783.1	Human enterovirus 71
6820.	AEE00782.1	Human enterovirus 71
6821.	AEE00781.1	Human enterovirus 71
6822.	AEE00780.1	Human enterovirus 71
6823.	AEE00779.1	Human enterovirus 71
6824.	AEE00778.1	Human enterovirus 71
6825.	AEE00777.1	Human enterovirus 71
6826.	AEE00776.1	Human enterovirus 71
6827.	AEE00775.1	Human enterovirus 71
6828.	AEE00774.1	Human enterovirus 71
6829.	AEE00773.1	Human enterovirus 71
6830.	AEE00772.1	Human enterovirus 71
6831.	AEE00771.1	Human enterovirus 71
6832.	AEE00770.1	Human enterovirus 71
6833.	AEE00769.1	Human enterovirus 71
6834.	AEE00768.1	Human enterovirus 71
6835.	AEE00767.1	Human enterovirus 71
6836.	AEE00766.1	Human enterovirus 71
6837.	ADZ73523.1	Human echovirus 30
6838.	ADZ73522.1	Human echovirus 30
6839.	ADZ73521.1	Human echovirus 30
6840.	ACM47982.1	Human enterovirus 71
6841.	ABQ02695.1	Human enterovirus 71
6842.	ADP21253.1	Human enterovirus 71
6843.	ADP21252.1	Human enterovirus 71
6844.	ADP21251.1	Human enterovirus 71
6845.	ADP21250.1	Human enterovirus 71
6846.	ADP21249.1	Human enterovirus 71
6847.	ADP21248.1	Human enterovirus 71
6848.	ADP21247.1	Human enterovirus 71
6849.	ADP21246.1	Human enterovirus 71
6850.	ADB77854.1	Human enterovirus 71
6851.	ADB77853.1	Human enterovirus 71
6852.	ADB77852.1	Human enterovirus 71
6853.	ADB77851.1	Human enterovirus 71

6854.	ADB77850.1	Human enterovirus 71
6855.	ADB77849.1	Human enterovirus 71
6856.	ADB77848.1	Human enterovirus 71
6857.	ADB77847.1	Human enterovirus 71
6858.	ADB77846.1	Human enterovirus 71
6859.	ADB77845.1	Human enterovirus 71
6860.	ADB77844.1	Human enterovirus 71
6861.	ADB77843.1	Human enterovirus 71
6862.	ADB77842.1	Human enterovirus 71
6863.	ADB77841.1	Human enterovirus 71
6864.	ADB77840.1	Human enterovirus 71
6865.	ADB77839.1	Human enterovirus 71
6866.	ADB77838.1	Human enterovirus 71
6867.	ADB77837.1	Human enterovirus 71
6868.	ADB77836.1	Human enterovirus 71
6869.	ADB77835.1	Human enterovirus 71
6870.	ADB77834.1	Human enterovirus 71
6871.	ADB77833.1	Human enterovirus 71
6872.	ADB77832.1	Human enterovirus 71
6873.	ADB77831.1	Human enterovirus 71
6874.	ADB77830.1	Human enterovirus 71
6875.	ADB77829.1	Human enterovirus 71
6876.	ADB77828.1	Human enterovirus 71
6877.	ADB77827.1	Human enterovirus 71
6878.	ADG84880.1	Human coxsackievirus A9
6879.	ACZ16009.1	Human enterovirus 71
6880.	ACZ16008.1	Human enterovirus 71
6881.	ACZ16007.1	Human enterovirus 71
6882.	ACZ16006.1	Human enterovirus 71
6883.	ACZ16005.1	Human enterovirus 71
6884.	ACZ16004.1	Human enterovirus 71
6885.	ACZ16003.1	Human enterovirus 71
6886.	BAA00168.1|1095-1416	Human rhinovirus 1B
6887.	CAA26181.1|1088-1409	Human rhinovirus 2
6888.	NP_041009.1|1100-1429	Human rhinovirus 14
6889.	BAA00168.1|1698-2157	Human rhinovirus 1B
6890.	CAA26181.1|1691-2150	Human rhinovirus 2
6891.	NP_041009.1|1720-2179	Human rhinovirus 14
6892.	1R1A_2	Human Rhinovirus Serotype 1a (Hrv1a)
6893.	BAA00168.1|70-332	Human rhinovirus 1B
6894.	CAA26181.1|70-330	Human rhinovirus 2
6895.	ACK37432.1|70-334	Human rhinovirus 7
6896.	ACK37369.1|70-330	Human rhinovirus 8
6897.	ACK37433.1|70-331	Human rhinovirus 9

6898.	ACK37434.1|70-334	Human rhinovirus 10
6899.	ACK37372.1|70-332	Human rhinovirus 13
6900.	ABF51187.1|70-331	Human rhinovirus 15
6901.	1QJY_2	Human Rhinovirus 16
6902.	ACK37374.1|70-328	Human rhinovirus 18
6903.	ACK37375.1|70-330	Human rhinovirus 19
6904.	ACK37376.1|70-331	Human rhinovirus 20
6905.	ACK37377.1|70-329	Human rhinovirus 21
6906.	ACK37378.1|70-330	Human rhinovirus 22
6907.	ABF51191.1|70-330	Human rhinovirus 23
6908.	ACK37446.1|70-331	Human rhinovirus 24
6909.	ACK37379.1|70-335	Human rhinovirus 25
6910.	ABF51202.1|70-332	Human rhinovirus 28
6911.	ACK37381.1|70-335	Human rhinovirus 29
6912.	ACK37435.1|70-330	Human rhinovirus 30
6913.	ACK37382.1|70-334	Human rhinovirus 31
6914.	ACK37383.1|70-331	Human rhinovirus 32
6915.	ACK37384.1|70-331	Human rhinovirus 33
6916.	ACK37445.1|70-328	Human rhinovirus 34
6917.	ABF51199.1|70-336	Human rhinovirus 36
6918.	ACK37436.1|70-331	Human rhinovirus 38
6919.	ACK37385.1|70-335	Human rhinovirus 40
6920.	ABF51185.1|70-332	Human rhinovirus 41
6921.	ACK37387.1|70-331	Human rhinovirus 43
6922.	ABF51193.1|70-335	Human rhinovirus 44
6923.	ACK37388.1|70-329	Human rhinovirus 45
6924.	ABF51200.1|70-333	Human rhinovirus 46
6925.	ACK37389.1|70-334	Human rhinovirus 47
6926.	ACK37390.1|70-330	Human rhinovirus 49
6927.	ACK37391.1|70-328	Human rhinovirus 50
6928.	ACK37392.1|70-331	Human rhinovirus 51
6929.	ABF51201.1|70-332	Human rhinovirus 53
6930.	ACK37395.1|70-334	Human rhinovirus 54
6931.	ACK37396.1|70-334	Human rhinovirus 56
6932.	ACK37397.1|70-331	Human rhinovirus 57
6933.	ACK37398.1|70-334	Human rhinovirus 58
6934.	ABF51194.1|70-334	Human rhinovirus 59
6935.	ACK37399.1|70-331	Human rhinovirus 60
6936.	ACK37400.1|70-330	Human rhinovirus 61
6937.	ACK37401.1|70-336	Human rhinovirus 62
6938.	ACK37402.1|70-334	Human rhinovirus 63
6939.	ACK37437.1|70-330	Human rhinovirus 64
6940.	ACK37403.1|70-331	Human rhinovirus 65
6941.	ACK37404.1|70-332	Human rhinovirus 66

6942.	ACK37405.1|70-331	Human rhinovirus 67
6943.	ACK37406.1|70-331	Human rhinovirus 68
6944.	ACK37408.1|70-331	Human rhinovirus 71
6945.	ABF51186.1|70-332	Human rhinovirus 73
6946.	ABF51188.1|70-331	Human rhinovirus 74
6947.	ABF51204.1|70-331	Human rhinovirus 75
6948.	ACK37438.1|70-331	Human rhinovirus 76
6949.	ACK37410.1|70-332	Human rhinovirus 77
6950.	ACK37439.1|70-333	Human rhinovirus 78
6951.	ACK37412.1|70-331	Human rhinovirus 80
6952.	ACK37415.1|70-330	Human rhinovirus 81
6953.	ACK37416.1|70-329	Human rhinovirus 82
6954.	ACK37419.1|70-335	Human rhinovirus 85
6955.	ABF51198.1|70-334	Human rhinovirus 88
6956.	ACK37440.1|70-336	Human rhinovirus 89
6957.	ACK37423.1|70-330	Human rhinovirus 90
6958.	ACK37441.1|70-330	Human rhinovirus 94
6959.	ACK37426.1|70-330	Human rhinovirus 95
6960.	ACK37427.1|70-330	Human rhinovirus 96
6961.	ACK37429.1|70-334	Human rhinovirus 98
6962.	ACK37431.1|70-334	Human rhinovirus 100
6963.	ACK37430.1|70-329	Human rhinovirus 99
6964.	ACK37428.1|70-330	Human rhinovirus 97
6965.	ACK37425.1|70-335	Human rhinovirus 92
6966.	ACK37424.1|70-331	Human rhinovirus 91
6967.	ACK37420.1|70-331	Human rhinovirus 86
6968.	ACK37418.1|70-330	Human rhinovirus 84
6969.	ACK37417.1|70-335	Human rhinovirus 83
6970.	ACK37411.1|70-335	Human rhinovirus 79
6971.	ACK37409.1|70-331	Human rhinovirus 72
6972.	ABF51183.1|70-331	Human rhinovirus 70
6973.	ACK37407.1|70-331	Human rhinovirus 69
6974.	ACK37393.1|70-331	Human rhinovirus 52
6975.	ABF51182.1|70-331	Human rhinovirus 48
6976.	ACK37386.1|70-329	Human rhinovirus 42
6977.	ACK37443.1|70-335	Human rhinovirus 35
6978.	ACK37442.1|70-331	Human rhinovirus 27
6979.	ACK37380.1|70-329	Human rhinovirus 26
6980.	AAA45756.1|70-331	Human rhinovirus 14
6981.	ABF51180.1|70-331	Human rhinovirus 6
6982.	ACK37368.1|70-329	Human rhinovirus 5
6983.	ABF51184.1|70-330	Human rhinovirus 4
6984.	ABF51179.1|70-331	Human rhinovirus 3
6985.	1R1A_3	Human Rhinovirus Serotype 1a (Hrv1a)

6986.	BAA00168.1|333-570	Human rhinovirus 1B
6987.	CAA26181.1|331-567	Human rhinovirus 2
6988.	ACK37432.1|335-573	Human rhinovirus 7
6989.	ACK37369.1|331-568	Human rhinovirus 8
6990.	ACK37433.1|332-569	Human rhinovirus 9
6991.	ACK37434.1|335-572	Human rhinovirus 10
6992.	ACK37372.1|333-570	Human rhinovirus 13
6993.	ABF51187.1|332-569	Human rhinovirus 15
6994.	1QJY_3	Human Rhinovirus 16
6995.	ACK37374.1|329-566	Human rhinovirus 18
6996.	ACK37375.1|331-568	Human rhinovirus 19
6997.	ACK37376.1|332-569	Human rhinovirus 20
6998.	ACK37377.1|330-570	Human rhinovirus 21
6999.	ACK37378.1|331-567	Human rhinovirus 22
7000.	ABF51191.1|331-566	Human rhinovirus 23
7001.	ACK37446.1|332-569	Human rhinovirus 24
7002.	ACK37379.1|336-573	Human rhinovirus 25
7003.	ABF51202.1|333-570	Human rhinovirus 28
7004.	ACK37381.1|336-573	Human rhinovirus 29
7005.	ACK37435.1|331-566	Human rhinovirus 30
7006.	ACK37382.1|335-572	Human rhinovirus 31
7007.	ACK37383.1|332-569	Human rhinovirus 32
7008.	ACK37384.1|332-569	Human rhinovirus 33
7009.	ACK37445.1|329-566	Human rhinovirus 34
7010.	ABF51199.1|337-574	Human rhinovirus 36
7011.	ACK37436.1|332-569	Human rhinovirus 38
7012.	ACK37385.1|336-573	Human rhinovirus 40
7013.	ABF51185.1|333-570	Human rhinovirus 41
7014.	ACK37387.1|332-569	Human rhinovirus 43
7015.	ABF51193.1|336-573	Human rhinovirus 44
7016.	ACK37388.1|330-567	Human rhinovirus 45
7017.	ABF51200.1|334-571	Human rhinovirus 46
7018.	ACK37389.1|335-572	Human rhinovirus 47
7019.	ACK37390.1|331-567	Human rhinovirus 49
7020.	ACK37391.1|329-566	Human rhinovirus 50
7021.	ACK37392.1|332-569	Human rhinovirus 51
7022.	ABF51201.1|333-570	Human rhinovirus 53
7023.	ACK37395.1|335-572	Human rhinovirus 54
7024.	ACK37396.1|335-572	Human rhinovirus 56
7025.	ACK37397.1|332-569	Human rhinovirus 57
7026.	ACK37398.1|335-572	Human rhinovirus 58
7027.	ABF51194.1|335-572	Human rhinovirus 59
7028.	ACK37399.1|332-569	Human rhinovirus 60
7029.	ACK37400.1|331-568	Human rhinovirus 61

7030.	ACK37401.1|337-574	Human rhinovirus 62
7031.	ACK37402.1|335-572	Human rhinovirus 63
7032.	ACK37437.1|331-567	Human rhinovirus 64
7033.	ACK37403.1|332-569	Human rhinovirus 65
7034.	ACK37404.1|333-570	Human rhinovirus 66
7035.	ACK37405.1|332-569	Human rhinovirus 67
7036.	ACK37406.1|332-569	Human rhinovirus 68
7037.	ACK37408.1|332-569	Human rhinovirus 71
7038.	ABF51186.1|333-570	Human rhinovirus 73
7039.	ABF51188.1|332-569	Human rhinovirus 74
7040.	ABF51204.1|332-569	Human rhinovirus 75
7041.	ACK37438.1|332-569	Human rhinovirus 76
7042.	ACK37410.1|333-570	Human rhinovirus 77
7043.	ACK37439.1|334-571	Human rhinovirus 78
7044.	ACK37412.1|332-569	Human rhinovirus 80
7045.	ACK37415.1|331-568	Human rhinovirus 81
7046.	ACK37416.1|330-566	Human rhinovirus 82
7047.	ACK37419.1|336-573	Human rhinovirus 85
7048.	ABF51198.1|335-572	Human rhinovirus 88
7049.	ACK37440.1|337-574	Human rhinovirus 89
7050.	ACK37423.1|331-568	Human rhinovirus 90
7051.	ACK37441.1|331-567	Human rhinovirus 94
7052.	ACK37426.1|331-568	Human rhinovirus 95
7053.	ACK37427.1|331-568	Human rhinovirus 96
7054.	ACK37429.1|335-572	Human rhinovirus 98
7055.	ACK37431.1|335-572	Human rhinovirus 100
7056.	ACK37430.1|330-565	Human rhinovirus 99
7057.	ACK37428.1|331-566	Human rhinovirus 97
7058.	ACK37425.1|336-571	Human rhinovirus 92
7059.	ACK37424.1|332-567	Human rhinovirus 91
7060.	ACK37420.1|332-567	Human rhinovirus 86
7061.	ACK37418.1|331-566	Human rhinovirus 84
7062.	ACK37417.1|336-571	Human rhinovirus 83
7063.	ACK37411.1|336-571	Human rhinovirus 79
7064.	ACK37409.1|332-567	Human rhinovirus 72
7065.	ABF51183.1|332-567	Human rhinovirus 70
7066.	ACK37407.1|332-567	Human rhinovirus 69
7067.	ACK37393.1|332-567	Human rhinovirus 52
7068.	ABF51182.1|332-567	Human rhinovirus 48
7069.	ACK37386.1|330-565	Human rhinovirus 42
7070.	ABF51181.1|336-571	Human rhinovirus 35
7071.	ACK37442.1|332-567	Human rhinovirus 27
7072.	ACK37380.1|330-565	Human rhinovirus 26
7073.	AAA45756.1|332-567	Human rhinovirus 14

7074.	ABF51180.1|332-567	Human rhinovirus 6
7075.	ACK37368.1 |330-565	Human rhinovirus 5
7076.	ABF51184.1|330-565	Human rhinovirus 4
7077.	ABF51179.1|332-567	Human rhinovirus 3
7078.	NP_042242.1|70-323	Human enterovirus A
7079.	NP_042242.1|324-565	Human enterovirus A
7080.	NP_042242.1|1112-1440	Human enterovirus A
7081.	NP_042242.1|1732-2193	Human enterovirus A
7082.	YP_003104774.1|70-329	Human enterovirus 107
7083.	YP_003104774.1|330-567	Human enterovirus 107
7084.	YP_003104774.1|1108-1436	Human enterovirus 107
7085.	YP_003104774.1|1731-2192	Human enterovirus 107
7086.	NP_040759.1|70-341	Human enterovirus C
7087.	NP_040759.1|342-581	Human enterovirus C
7088.	NP_040759.1|1126-1453	Human enterovirus C
7089.	NP_040759.1|1746-2206	Human enterovirus C
7090.	NP_040760.1|70-319	Human enterovirus D
7091.	NP_040760.1|320-561	Human enterovirus D
7092.	NP_040760.1|320-561	Human enterovirus D
7093.	NP_040760.1|1738-2194	Human enterovirus D
7094.	NP_758520.1|70-315	Porcine enterovirus B
7095.	NP_758520.1|316-553	Porcine enterovirus B
7096.	NP_758520.1|1085-1413	Porcine enterovirus B
7097.	NP_758520.1|1708-2168	Porcine enterovirus B
7098.	NP_653149.1|70-319	Simian enterovirus A
7099.	NP_653149.1|320-558	Simian enterovirus A
7100.	NP_653149.1|1118-1446	Simian enterovirus A
7101.	NP_653149.1|1739-2197	Simian enterovirus A
